# Supplementary material for: Diverse redox-mediated transformations to realize the para-quinoid, σ-bond, and ortho-diphenoquinoid forms
Source: Nat Commun. 2025 May 8;16:4088. doi: 10.1038/s41467-025-59317-w (PMC12062416; doi:10.1038/s41467-025-59317-w)
Supplement: Supplementary file 1 — Supplementary Information [file 41467_2025_59317_MOESM1_ESM.pdf]

Supplementary Information for

Diverse Redox-Mediated Transformations to Realize the *Para*-Quinoid, σ-Bond, and *Ortho*-Diphenoquinoid Forms

Takashi Harimoto,[1,2]* Moto Kikuchi,[1] Takanori Suzuki,[1] and Yusuke Ishigaki[1]*

[1]*Department of Chemistry, Faculty of Science, Hokkaido University, Sapporo 060-0810, Japan*

[2]*Present address: Institute for Molecular Science, Myodaiji, Okazaki 444-8787, Japan*

*Correspondence to: yishigaki@sci.hokudai.ac.jp, t-harimoto@ims.ac.jp

Table of Contents

Experimental Section **S2**

NMR spectra of new compounds (Figures S1-S14) **S16**

Redox Properties **S28**

Differential pulse voltammetry (Figure S15) **S28**

Cyclic voltammetry (Figures S16 and S17) **S29**

Spectroscopic Investigation **S31**

UV−vis−NIR spectroscopy (Figure S18) **S31**

VT-^{1}H NMR spectroscopy (Figures S19-S21) **S32**

X-ray Analysis (Figures S22-S25 and Tables S1-S12) **S35**

Theoretical Study **S51**

DFT calculations (Figures S26-S44 and Tables S13 and S14) **S51**

TD-DFT calculations (Figures S45-S47) **S73**

CASSCF calculations (Figure S48) **S76**

ACID plots (Figure S49) **S78**

NICS calculations (Figure S50) **S79**

TD-DFT Excitation Energies **S80**

References **S155**

Experimental Section

Synthetic Procedures

5,7,12,14-Tetrakis(dibromomethylene)-5,7,12,14-tetrahydrodibenzo[*b*,*i*]thianthrene 6-oxide 3

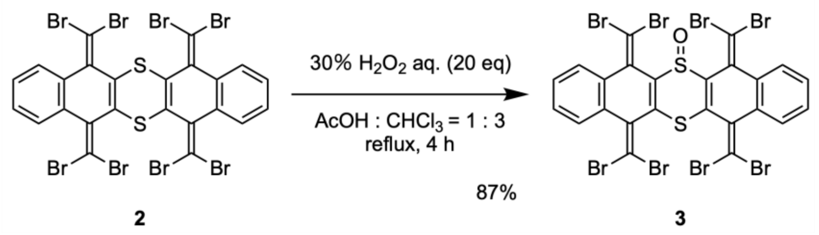

A mixture of 5,7,12,14-tetrakis(dibromomethylene)-5,7,12,14-tetrahydrodibenzo[*b*,*i*]thianthrene **2**[1] (5.01 g, 5.01 mmol) and 30% H_2O_2 aq. (11.4 mL, 100 mmol) in $CHCl_3$ (75 mL) and AcOH (25 mL) was heated at reflux for 4 h. After cooling to 25 °C, the mixture was filtered and washed with water. The solvent was dried under reduced pressure to give **3** (4.41 g) as a white solid in 87 % yield.

3; Mp: 203-207 °C (decomp.) ^{1}H NMR (400 MHz, $CDCl_3$): δ/ppm 7.97 (1H, dd, J = 1.2, 7.6 Hz), 7.88-7.81 (1H, m), 7.71 (1H, dd, J = 1.2, 7.6 Hz), 7.71-7.73 (1H, m), 7.40-7.30 (4H, m); ^{13}C NMR (100 MHz, $CDCl_3$): δ/ppm 139.78, 139.59, 139.33, 139.26, 138.18, 136.65, 136.65, 135.89, 135.55, 135.53, 134.08, 134.02, 128.58, 127.89, 127.72, 127.70, 127.48, 127.30, 127.30, 126.92, 99.80, 96.44, 92.74, 92.14; IR (ATR): ν/cm^{-1} 3058, 2991, 1700, 1595, 1586, 1549, 1533, 1506, 1489, 1451, 1294, 1234, 1155, 1089, 1054, 910, 895, 853, 764, 755, 745, 664, 647, 637, 627, 609, 572, 541, 527, 452, 420; LR-MS (FD) m/z (%):1021.26 (3), 1020.26 (3), 1019.27 (7), 1018.28 (6), 1017.27 (16), 1016.27 (7), 1015.28 (16, M$^+$, $C_{24}H_8{}^{79}Br_4{}^{81}Br_4OS_2$), 1014.28 (6), 1013.27 (12), 1012.28 (3), 1011.28 (7), 971.30 (5), 970.32 (2), 969.30 (9), 968.32 (4), 967.31 (13, [M−SO]$^+$, $C_{24}H_8{}^{79}Br_4{}^{81}Br_4S$), 966.30 (2), 965.31 (10), 964.31 (1), 963.31 (4), 940.36 (27), 939.36 (20), 938.36 (67), 937.37 (31), 936.36 (bp, [M−Br]$^+$, $C_{24}H_8{}^{79}Br_3{}^{81}Br_4OS_2$), 935.37 (29), 934.36 (99), 933.37 (16), 932.36 (59), 931.37 (6), 930.36 (18); HR-MS (FD) Calcd. for $C_{24}H_8{}^{79}Br_4{}^{81}Br_4OS_2$: 1015.34027; Found: 1015.34102 (MS error 0.74 ppm).

5,7,12,14-Tetrakis[bis(2-fluoro-4-methoxyphenyl)methylene]-5,7,12,14-tetrahydrodibenzo[*b,i*]thianthrene 6-oxide (4a)

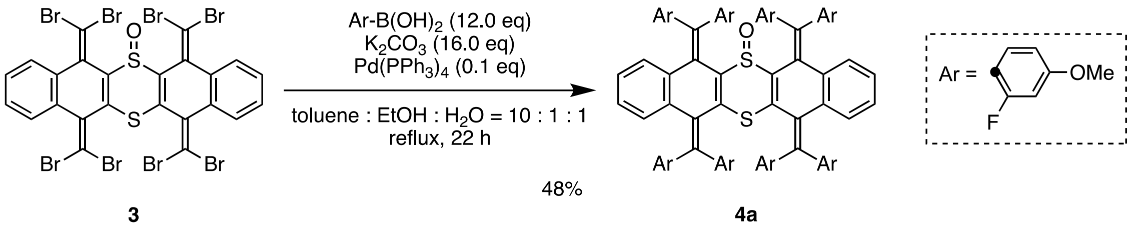

A mixture of **3** (1.57 g, 1.55 mmol), 2-fluoro-4-methoxyphenylboronic acid (3.16 g, 18.6 mmol), K_2CO_3 (3.43 g, 24.8 mmol), and $Pd(PPh_3)_4$ (179 mg, 155 μmol) in toluene (31 mL), EtOH (3.1 mL), and H_2O (3.1 mL) was heated at reflux for 22 h. After cooling to 25 °C, the mixture was diluted with water and extracted with EtOAc five times. The combined organic layers were washed with water and brine, and dried over anhydrous Na_2SO_4. After filtration, the solvent was concentrated under reduced pressure. The crude product was purified by column chromatography on silica gel (hexane/EtOAc = 3) to give **4a** (1.02 g) as a yellow solid in 48 % yield.

4a; Mp: 216-220 °C (decomp.); ^{1}H NMR (400 MHz, $CDCl_3$, 323 K): δ/ppm 7.35 (4H, brs), 7.19-6.00 (28H, m), 3.95-3.80 (6H, m), 3.80-3.60 (18H, m); ^{13}C NMR could not be recorded due to inevitable broadening of the signals caused by the existence of multiple conformers.; IR (ATR): *ν*/cm^{-1} 3066, 3000, 2953, 2936, 2907, 2836, 1617, 1570, 1503, 1465, 1442, 1427, 1316, 1287, 1268, 1245, 1228, 1193, 1153, 1115, 1080, 1028, 953, 905, 832, 795, 765, 745, 727, 715, 694, 628, 576; LR-MS (FD) m/z (%): 1380.39 (9), 1379.40 (22), 1378.39 (48), 1377.39 (89), 1376.38 (M^+, bp), 1331.41 (7), 1330.42 (19), 1329.42 (36), 1328.41 ($[M{-}SO]^+$, 39), 689.70 (5), 689.20 (19), 688.69 (19), 688.20 (M^{2+}, 22); HR-MS (FD) Calcd. for $C_{80}H_{56}F_8O_9S_2$: 1376.32380; Found: 1376.32548 (MS error 1.22 ppm); Elemental Analysis Calcd. (%) for $C_{80}H_{56}F_8O_9S_2$: C 69.76, H 4.10; Found: C 69.75, H 4.17.

5,7,12,14-Tetrakis[bis(4-methoxyphenyl)methylene]-5,7,12,14-tetrahydrodibenzo[*b*,*i*]thianthrene 6-oxide (4b)

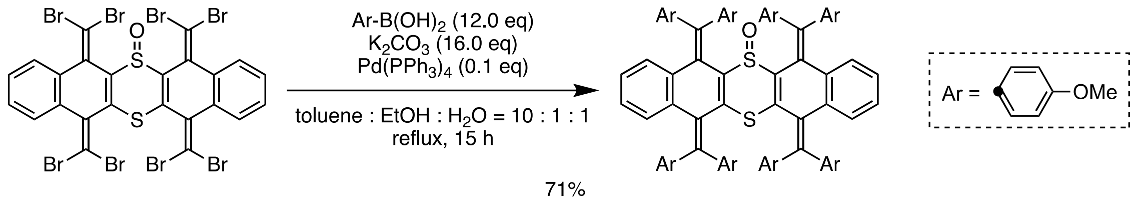

A mixture of **3** (260 mg, 256 μmol), 4-methoxyphenylboronic acid (467 mg, 3.07 mmol), K_2CO_3 (567 mg, 4.10 mmol), and $Pd(PPh_3)_4$ (29.6 mg, 25.6 μmol) in toluene (5.0 mL), EtOH (0.50 mL), and H_2O (0.50 mL) was heated at reflux for 15 h. After cooling to 25 °C, the mixture was diluted with water and extracted with CH_2Cl_2 five times. The combined organic layers were washed with water and brine, and dried over anhydrous Na_2SO_4. After filtration, the solvent was concentrated under reduced pressure. The crude product was purified by column chromatography on silica gel (hexane/EtOAc = 3) to give **4b** (225 mg) as a yellow solid in 71 % yield.

4b; Mp: 246-250 °C (decomp.); ^{1}H NMR (400 MHz, $CDCl_3$): δ/ppm 7.19 (4H, d, *J* = 8.0 Hz), 7.10 (4H, d, *J* = 8.4 Hz), 7.07 (4H, d, *J* = 7.6 Hz), 7.05 (4H, d, *J* = 8.4 Hz), 6.90-6.84 (2H, m), 6.84-6.84-6.81 (2H, m), 6.78 (8H, d, *J* = 8.0 Hz), 6.81-6.71 (8H, m), 6.67 (4H, d, *J* = 7.6 Hz), 3.81 (12H, s), 3.77 (12H, s); ^{13}C NMR (100 MHz, $CDCl_3$): δ/ppm 159.02, 158.72, 158.05, 158.34, 139.24, 137.15, 136.25, 136.08, 135.21, 134.45, 134.25, 133.81, 132.59, 131.33, 131.00, 130.65, 130.55, 130.35, 128.16, 127.77, 124.81, 124.72, 113.95, 113.65, 55.22, 55.18; IR (ATR): ν/cm^{-1} 3060, 3031, 2997, 2953, 2931, 2903, 2833, 1603, 1570, 1505, 1462., 1441, 1286, 1241, 1172, 1109, 1030, 950, 916, 849, 824, 763, 744, 693, 633, 587, 554; LR-MS (FD) m/z (%): 1236.32 (10), 1235.32 (23), 1234.32 (53), 1233.32 (91), 1232.32 (M$^+$, bp), 1187.36 (5), 1186.36 (11), 1185.36 (20), 1184.36 ([M−SO]$^+$, 23); HR-MS (FD) Calcd. for $C_{80}H_{64}O_9S_2$: 1232.39766; Found: 1232.39917 (MS error 1.22 ppm).

5,7,12,14-Tetrakis[bis(4-methoxy-2-methylphenyl)methylene]-5,7,12,14-tetrahydrodibenzo[*b*,*i*]thianthrene 6-oxide (4c)

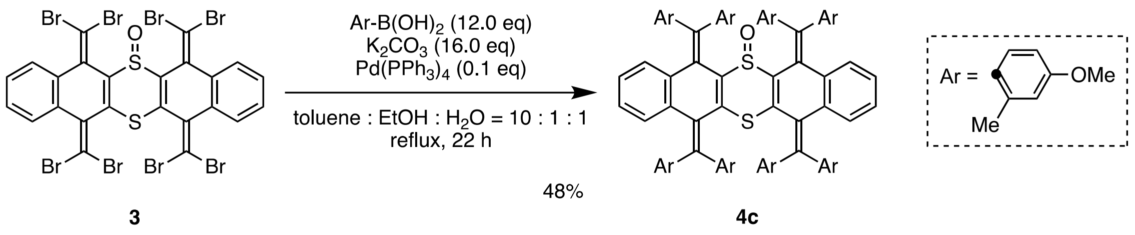

A mixture of **3** (1.32 g, 1.30 mmol), 4-methoxy-2-methylphenylboronic acid (2.58 g, 15.5 mmol), K_2CO_3 (2.87 g, 20.7 mmol), and $Pd(PPh_3)_4$ (150 mg, 130 μmol) in toluene (26 mL), EtOH (2.6 mL), and H_2O (2.6 mL) was heated at reflux for 22 h. After cooling to 25 °C, the mixture was diluted with water and extracted with EtOAc five times. The combined organic layers were washed with water and brine, and dried over anhydrous Na_2SO_4. After filtration, the solvent was concentrated under reduced pressure. The crude product was purified by column chromatography on silica gel (hexane/EtOAc = 3) to give **4c** (842 mg) as a yellow solid in 48 % yield.

4c; Mp: 298-300 °C (decomp.); ^{1}H NMR (400 MHz, DMSO-d_6, 383 K): δ/ppm 7.15 (4H, brs), 7.30-6.94 (4H, m), 6.91 (2H, br), 6.89-6.60 (18H, m), 6.58 (2H, br), 6.41 (2H, br), 3.82 (6H, s), 3.75 (6H, s) 3.71 (6H, s) 3.70 (6H, s), 2.22 (6H, s), 2.03 (6H, s), 1.97 (6H, s), 1.93 (6H, s); ^{13}C NMR could not be recorded due to inevitable broadening of the signals caused by the existence of multiple conformers.; IR (ATR): *ν*/cm^{-1} 3054, 2996, 2937, 2915, 2833, 1603, 1566, 1495, 1464, 1451, 1442, 1375, 1307, 1290, 1230, 1196, 1161, 1116, 1079, 1041, 938, 861, 845, 808, 792, 761, 729, 691, 605, 565, 447; LR-MS (FD) m/z (%): 1348.60 (14), 1347.60 (32), 1346.60 (61), 1345.61 (99), 1344.59 (M^+, bp), 1299.66 (18), 1298.63 (33), 1297.64 (62), 1296.63 ($[M-SO]^+$, 68); HR-MS (FD) Calcd. for $C_{88}H_{80}O_9S_2$: 1344.52437; Found: 1344.52686 (MS error 1.85 ppm).

5,7,12,13-Tetrakis[bis(2-fluoro-4-methoxyphenyl)methylene]-5,7,12,13-tetrahydrodinaphtho[2,3-*b*:2',3'-*d*]thiophene (1a)

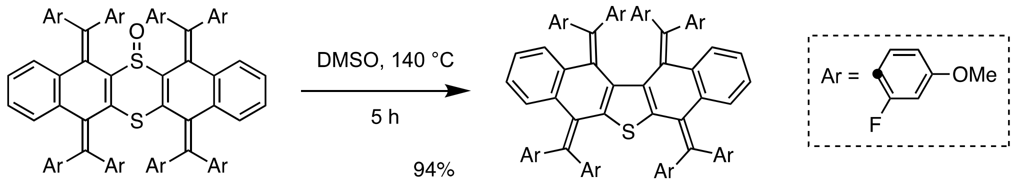

4a **1a**

A solution of **4a** (117 mg, 84.9 μmol) in DMSO (3 mL) was heated at 140 °C for 5 h. After cooling to 25 °C, the mixture was diluted with water and extracted with Et_2O five times. The combined organic layers were washed with water and brine, and dried over anhydrous Na_2SO_4. After filtration, the solvent was concentrated under reduced pressure. The crude product was purified by column chromatography on silica gel (hexane/EtOAc = 3) to give **1a** (106 mg) as a yellow solid in 94 % yield.

1a; Mp: 288-293 °C (decomp.); ^{1}H NMR (400 MHz, DMSO-d_6, 413 K): δ/ppm 7.46 (2H, brs), 7.07 (2H, brs), 7.02-6.90 (4H, m), 6.85-6.70 (8H, m), 6.70-6.46 (12H, m), 6.25-6.06 (4H, m), 3.95 (6H, s), 3.73 (6H, s), 3.69 (6H, s), 3.38 (6H, s); ^{13}C NMR could not be recorded due to inevitable broadening of the signals caused by the existence of multiple conformers.; IR (ATR): *ν*/cm^{-1} 3067, 3006, 2956, 2931, 2907, 2835, 1616, 1571, 1504, 1463, 1454, 1440, 1426, 1319, 1288, 1241, 1227, 1190, 1151, 1115, 1085, 1028, 946, 830, 808, 789, 760, 745, 728, 681, 628, 587, 541; LR-MS (FD) m/z (%): 1332.49 (6), 1331.49 (17), 1330.48 (48), 1329.48 (91), 1328.48 (M^+, bp); HR-MS (FD) Calcd. for $C_{80}H_{56}F_8O_8S$: 1328.35681; Found: 1328.35919 (MS error 1.79 ppm); Elemental Analysis Calcd. (%) for $C_{80}H_{56}F_8O_8S$: C 72.28, H 4.25; Found: C 72.01, H 4.30; UV−vis (CH_2Cl_2): λ_{max}/nm (ε/L mol^{-1} cm^{-1}) 345 (34800).

5,7,12,13-Tetrakis[bis(4-methoxyphenyl)methylene]-5,7,12,13-tetrahydrodinaphtho[2,3-*b*:2',3'-*d*]thiophene (1b)

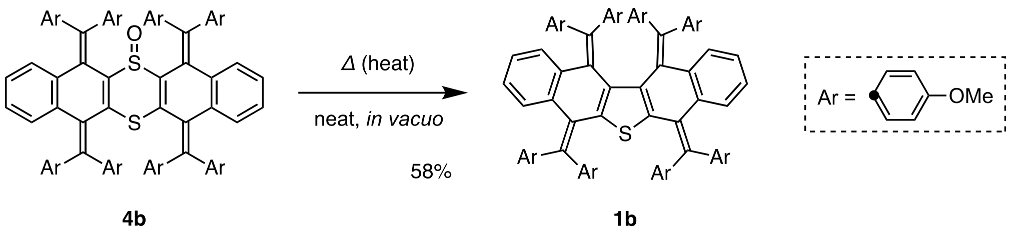

A solid of **4b** (125 mg, 101 μmol) in a pyrex glass tube was heated by a heat gun under reduced pressure for 5 min. After cooling to 25 °C, the crude product was purified by column chromatography on silica gel (hexane/EtOAc = 3) to give **1b** (68.8 mg) as a yellow solid in 58 % yield.

1b; Mp: > 300 °C; ^{1}H NMR (400 MHz, $CDCl_3$): δ/ppm 7.18 (4H, d, *J* = 8.2 Hz), 7.15 (4H, d, *J* = 8.2 Hz), 7.05 (4H, d, *J* = 8.8 Hz), 6.96 (4H, d, *J* = 8.8 Hz), 6.87 (4H, d, *J* = 8.8 Hz), 6.86 (4H, d, *J* = 8.8 Hz), 6.82 (2H, ddd, *J* = 1.2, 7.2, 7.6 Hz), 6.58 (4H, d, *J* = 8.8 Hz), 6.51 (2H, ddd, *J* = 1.2, 7.2, 7.6 Hz), 6.38 (2H, td, *J* = 1.2, 7.6 Hz), 6.25 (4H, d, *J* = 8.8 Hz), 6.09 (2H, dd, *J* = 1.2, 7.6 Hz), 4.04 (6H, s), 3.82 (6H, s), 3.69 (6H, s), 3.34 (6H, s); ^{13}C NMR (100 MHz, $CDCl_3$): δ/ppm 159.02, 158.35, 157.95, 157.83, 138.81, 138.23, 137.15, 136.78, 136.73, 135.83, 135.81, 135.32, 135.04, 134.17, 132.15, 131.35, 130.96, 130.46, 129.98, 129.84, 128.62, 128.30, 123.77, 123.48, 114.38, 113.41, 113.20, 112.85, 55.44, 55.19, 55.11, 54.64; IR (ATR): *ν*/cm^{-1} 3062, 3028, 2996, 2947, 2930, 2903, 2833, 1603, 1506, 1457, 1437, 1285, 1240, 1171, 1103, 1030, 950, 827, 814 , 760, 732, 702, 651, 635, 608, 587, 550, 517; LR-MS (FD) m/z (%): 1188.34 (9), 1187.34 (18), 1186.34 (47), 1185.34 (90), 1184.34 (M^+, bp), 592.68 (6), 592.18 (M^{2+}, 6); HR-MS (FD) Calcd. for $C_{80}H_{64}O_8S$: 1184.43219; Found: 1184.43334 (MS error 0.97 ppm); UV−vis (CH_2Cl_2): λ_{max}/nm (ε/L mol^{-1} cm^{-1}) 366 (37700).

5,7,12,13-Tetrakis[bis(4-methoxy-2-methylphenyl)methylene]-5,7,12,13-tetrahydrodinaphtho[2,3-*b*:2',3'-*d*]thiophene (1c)

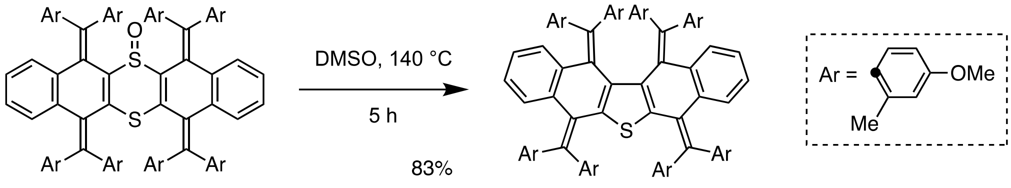

4c **1c**

A solution of **4c** (32.8 mg, 84.9 μmol) in DMSO (3 mL) was heated at 140 °C for 5 h. After cooling to 25 °C, the mixture was diluted with water and extracted with Et_2O five times. The combined organic layers were washed with water and brine, and dried over anhydrous Na_2SO_4. After filtration, the solvent was concentrated under reduced pressure. The crude product was purified by column chromatography on silica gel (hexane/EtOAc = 3) to give **1c** (26.1 mg) as a yellow solid in 83 % yield.

1c; Mp: > 300 °C; ^{1}H NMR (400 MHz, DMSO-d_6, 411 K): δ/ppm 7.33 (2H, brs), 7.10-6.20 (28H, m), 5.80 (2H, brs), 3.95 (6H, s), 3.74 (6H, s), 3.63 (6H, s), 3.34 (6H, s), 2.40-1.40 (24H, m); ^{13}C NMR could not be recorded due to inevitable broadening of the signals caused by the existence of multiple conformers.; IR (ATR): ν/cm^{-1} 3077, 3059, 3022, 2998, 2952, 2937, 2917, 2833, 1605, 1565, 1494, 1465, 1450, 1441, 1374, 1290, 1245, 1230, 1163, 1123, 1111, 1086, 1048 , 990 , 933, 856, 842, 802, 787, 763, 745, 726, 705, 648, 636, 604, 588, 566, 507, 474; LR-MS (FD) m/z (%): 1300.70 (7), 1299.70 (22), 1298.70 (53), 1297.69 (98), 1296.69 (M^+, bp); HR-MS (FD) Calcd. for $C_{88}H_{80}O_8S$: 1296.55739; Found: 1296.55937 (MS error 1.53 ppm); Elemental Analysis Calcd. (%) for $C_{88}H_{80}O_8S$: C 81.46, H 6.21; Found: C 81.17, H 6.25; UV−vis (CH_2Cl_2): λ_{max}/nm (ε/L mol^{-1} cm^{-1}) 364 (37000).

7,12-Bis[bis(2-fluoro-4-methoxyphenyl)methylene]-7,12-dihydrodinaphtho[2,3-*b*:2',3'-*d*]thiophene-5,13-diyl-bis[bis(2-fluoro-4-methoxyphenyl)methylium] bis(hexachloroantimonate) [1a^{2+}(SbCl$_6^-$)$_2$]

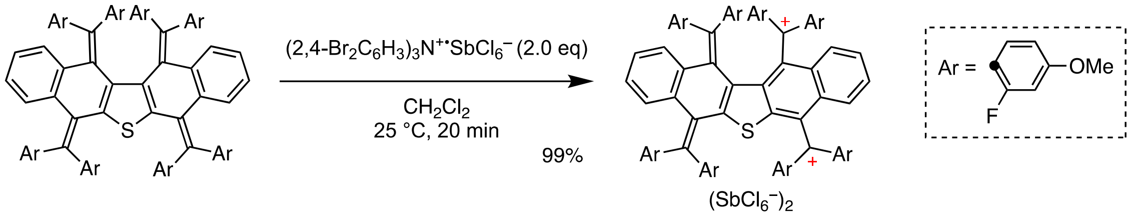

1a 1**a**$^{2+}$(SbCl$_6^-$)$_2$

To a solution of **1a** (65.0 mg, 48.9 µmol) in dry CH_2Cl_2 (2 mL) was added tris(2,4-dibromophenyl)aminium hexachloroantimonate (103 mg, 97.8 µmol) at 25 °C to generate a deep red solution, and the mixture was stirred at 25 °C for 20 min. The addition of dry hexane led to precipitation of the dication salt. The solvent was decantated and the resulting precipitates were washed with dry hexane five times, and collected by filtration to give **1a**$^{2+}$(SbCl$_6^-$)$_2$ (97.1 mg) as a dark red powder in 99% yield.

1a^{2+}(SbCl$_6^-$)$_2$; Mp: 179-185 °C (decomp.); ^{1}H NMR (400 MHz, CD_3CN): δ/ppm 7.33 (4H, brs), 7.20 (4H, brs), 7.15-7.02 (6H, m), 7.01-6.81 (12H, m), 6.71 (4H, brs), 6.40 (2H, brs), 4.05 (12H, brs), 3.98 (6H, s), 3.84 (6H, brs); ^{13}C NMR could not be recorded due to inevitable broadening of the signals caused by the existence of multiple conformers.; IR (ATR): ν/cm^{-1} 3085, 2934, 2840, 1616, 1590, 1503, 1441, 1363, 1286, 1208, 1188, 1153, 1105, 1008, 948, 931, 842, 811, 759, 712, 632, 556, 505; LR-MS (FD) m/z (%): 1668.14 (7), 1667.14 (12), 1666.14 (16), 1665.14 (22), 1664.15 (22), 1663.14 (30), 1662.14 (17), 1661.14 (21), 1660.15 (6), 1659.14 (7, M^{2+}+SbCl$_6^-$), 1331.43 (16), 1330.42 (39), 1329.43 (75), 1328.42 (85, $M^{2+}+e^-$), 1316.40 (7), 1315.39 (21), 1314.39 (41), 1313.39 (44, $[M-CH_3]^+$), 666.20 (5), 665.70 (19), 665.20 (50), 664.70 (92), 664.20 (bp, M^{2+}); HR-MS (FD) Calcd. for $C_{80}H_{56}F_8O_8S$: 1328.35681; Found: 1328.35684 (MS error 0.02 ppm); Elemental Analysis Calcd. (%) for $C_{80}H_{56}F_8O_8S{\bullet}Sb_2Cl_{12}$: C 48.09, H 2.82; Found: C 48.25, H 2.94; UV−vis−NIR (CH_2Cl_2): λ_{max}/nm (ε/L mol^{-1} cm^{-1}) 970 (4500), 715 (7400), 562 (75600), 367 (29600), 272 (77800).

12,12,13,13-Tetrakis(4-methoxyphenyl)-12,13-dihydropiceno[6,7-*bcd*]thiophene-5,7-diyl-bis[bis(4-methoxyphenyl)methylium] bis(hexachloroantimonate) [1b^{2+}(SbCl$_6^-$)$_2$]

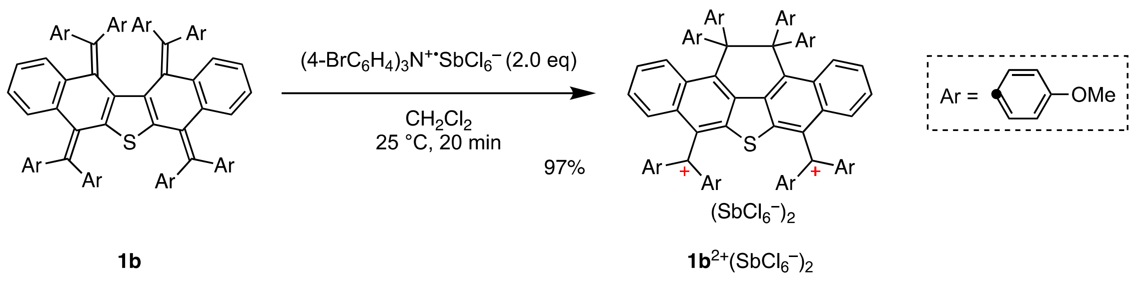

To a solution of **1b** (120 mg, 101 μmol) in dry CH_2Cl_2 (2 mL) was added tris(4-bromophenyl)aminium hexachloroantimonate (165 mg, 202 μmol) at 25 °C to generate a deep red solution, and the mixture was stirred at 25 °C for 20 min. The addition of dry hexane led to precipitation of the dication salt. The solvent was decantated and the resulting precipitates were washed with dry hexane five times, and collected by filtration to give **1b**$^{2+}$(SbCl$_6^-$)$_2$ (182 mg) as a dark red powder in 97% yield.

1b^{2+}(SbCl$_6^-$)$_2$; Mp: 191-196 °C (decomp.); ^{1}H NMR (400 MHz, CD_3CN): δ/ppm 7.69 (8H, brd, *J* = 8.0 Hz), 7.64 (2H, dd, *J* = 0.8, 8.8 Hz), 7.53 (8H, brs), 7.30 (2H, dd, *J* = 0.8, 8.8 Hz), 7.25 (8H, brd, *J* = 8.0 Hz), 7.15 (2H, ddd, *J* = 0.8, 6.8, 8.8 Hz), 7.12 (2H, ddd, *J* = 0.8, 6.8, 8.8 Hz), 6.54 (8H, brd, *J* = 8.0 Hz), 4.16 (12H, s), 3.68 (12H, s); ^{13}C NMR (100 MHz, CD_3CN): δ/ppm 190.18, 173.89, 158.54, 147.54, 146.58, 144.40, 137.51, 134.42, 134.28, 133.63, 131.75, 130.67, 130.59, 130.33, 128.26, 127.56, 125.23, 118.38, 112.39, 70.31, 58.34, 55.44; IR (ATR): ν/cm^{-1} 3101, 3069, 3039, 3003, 2935, 2842, 1606, 1576, 1505, 1448, 1371, 1321, 1279, 1253, 1183, 1152, 1121, 1030, 1001, 920, 870, 862, 845, 830, 803, 792, 769, 760, 749, 727, 714, 705, 685, 669, 655, 640, 623, 583, 559, 534, 516, 471; LR-MS (FD) m/z (%): 1525.24 (8), 1524.20 (11), 1523.20 (18), 1522.21 (27), 1521.20 (49), 1520.22 (40), 1519.21 (58), 1518.21 (30), 1517.20 (39), 1516.19 (8), 1515.22 (14, M^{2+}+SbCl$_6^-$), 1188,46 (5), 1187.47 (10), 1186.46 (13), 1185.48 (22), 1184.48 (17, M^{2+}+e^-), 1173.49 (7), 1172.46 (18), 1171.45 (47), 1170.46 (97), 1169.45 (97, [M−CH_3]$^+$), 1156.44 (9), 1155.43 (12), 1154.42 (19, [M−2CH_3]$^+$), 594.24 (8), 593.74 (20), 593.24 (52), 592.73 (90), 592.24 (bp, M^{2+}); HR-MS (FD) Calcd. for $C_{80}H_{64}O_8S$: 1184.43219; Found: 1184.43118 (MS error 0.85 ppm); UV−vis−NIR (CH_2Cl_2): λ_{max}/nm (ε/L mol^{-1} cm^{-1}) 710 (7800), 523 (169500), 430 (26200), 356 (49400), 321 (52100), 273 (114700).

7,12-Bis[bis(4-methoxy-2-methylphenyl)methylene]-7,12-dihydrodinaphtho[2,3-*b*:2',3'-*d*]thiophene-5,13-diyl-bis[bis(4-methoxy-2-methylphenyl)methylium] bis(hexachloroantimonate) [1c^{2+}($SbCl_6^-$)$_2$]

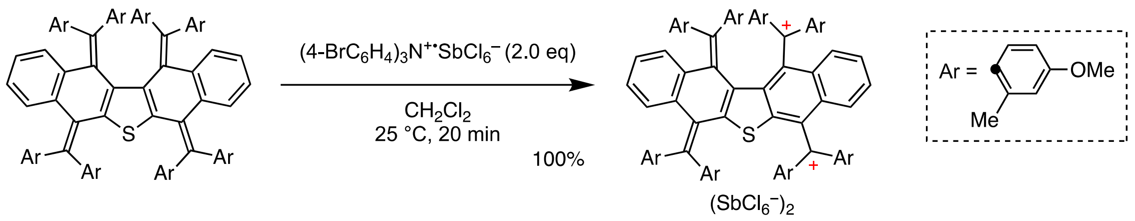

1c 1c^{2+}($SbCl_6^-$)$_2$

To a solution of **1c** (80.9 mg, 62.3 μmol) in dry CH_2Cl_2 (2 mL) was added tris(4-bromophenyl)aminium hexachloroantimonate (102 mg, 125 μmol) at 25 °C to generate a deep blue solution, and the mixture was stirred at 25 °C for 20 min. The addition of dry hexane led to precipitation of the dication salt. The solvent was decantated and the resulting precipitates were washed with dry hexane five times, and collected by filtration to give **1c**$^{2+}$($SbCl_6^-$)$_2$ (123 mg) as a dark red powder in 100% yield.

1c^{2+}($SbCl_6^-$)$_2$; Mp: 169-181 °C (decomp.); ^{1}H NMR (400 MHz, 1,1,2,2-tetrachloroethane-*d*$_2$, 293 K): δ/ppm 8.34-6.10 (32H, m), 4.23 (6H, brs), 4.09 (6H, brs), 3.89 (6H, brs), 3.74 (6H, brs), 2.00 (18H, m), 1.47 (6H, brs); ^{13}C NMR could not be recorded due to inevitable broadening of the signals caused by the existence of multiple conformers.; IR (ATR): *ν*/cm^{-1} 3064, 2941, 2836, 1599, 1581, 1534, 1493, 1451, 1438, 1344, 1295, 1219, 1186, 1163, 1102, 1029, 988, 935, 857, 811, 762, 705, 637, 609, 560, 510; LR-MS (FD) m/z (%): 1635.34 (5), 1634.34 (7), 1633.34 (11), 1632.34 (11), 1631.34 (13), 1630.35 (9), 1629.35 (11), 1628.33 (5), 1627.34 (4, M^{2+}+$SbCl_6^-$), 1300.61 (6), 1299.63 (19), 1298.62 (49), 1297.62 (92), 1296.62 (bp, M^{2+}+e^-), 1295.61 (19), 1294.60 (12), 1293.59 (8), 649.81 (9), 649.31 (22), 648.80 (39), 648.31 (42, M^{2+}), 647.80 (5); HR-MS (FD) Calcd. for $C_{88}H_{80}O_8S$: 1296.55739; Found: 1296.55628 (MS error 0.86 ppm); Elemental Analysis Calcd. (%) for $C_{88}H_{80}O_8S$•Sb_2Cl_{12}•0.5C_6H_{14}: C 54.39, H 4.36; Found: C 54.25, H 4.26; UV−vis−NIR (CH_2Cl_2): λ_{max}/nm (ε/L mol^{-1} cm^{-1}) 1190 (5000), 720 (sh, 12800), 583 (46600), 454 (26400), 378 (26200), 272 (75200).

5,7-Bis[bis(4-methoxyphenyl)methyl]-12,12,13,13-tetrakis(4-methoxyphenyl)-12,13-dihydropiceno[6,7-*bcd*]thiophene (1b-2H)

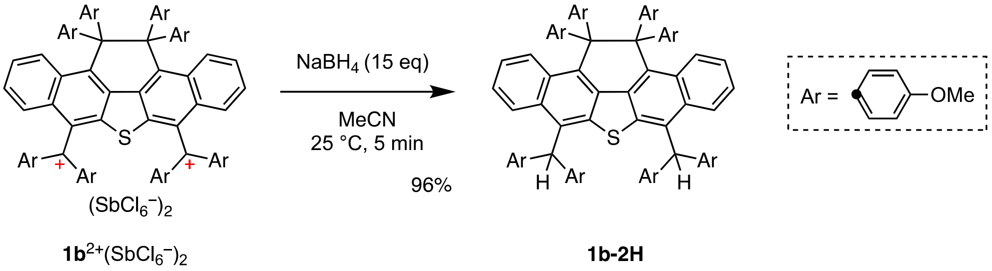

To a solution of **1b**$^{2+}(SbCl_6^-)_2$ (72.2 mg, 38.9 μmol) in dry MeCN (2.0 mL) was added sodium borohydride ($NaBH_4$) (22.1 mg, 582 μmol). The mixture was stirred at 25 °C for 5 min, and then diluted with water. The whole mixture was extracted with CH_2Cl_2 five times. The combined organic layers were washed with water and brine, and dried over anhydrous Na_2SO_4. After filtration through silica gel, the solvent was concentrated under reduced pressure to give **1b-2H** (44.5 mg) as a pale-yellow solid in 96% yield.

1b-2H; Mp: 152-162 °C (decomp.); ^{1}H NMR (400 MHz, $CDCl_3$): δ/ppm 7.94 (2H, brd, *J* = 8.6 Hz), 7.48 (2H, brd, *J* = 8.6 Hz), 7.28 (8H, d, *J* = 8.8 Hz), 7.14 (8H, d, *J* = 8.8 Hz), 7.03 (2H, ddd, *J* = 1.2, 6.8, 8.6 Hz), 6.84 (8H, d, *J* = 8.8 Hz), 6.74 (2H, ddd, *J* = 1.2, 6.8, 8.6 Hz), 6.34 (8H, d, *J* = 8.8 Hz), 6.29 (2H, s), 3.80 (12H, s), 3.62 (12H, s); ^{13}C NMR (100 MHz, $CDCl_3$): δ/ppm 158.33, 157.06, 137.91, 137.22, 135.58, 134.29, 133.47, 133.39, 132.26, 131.74, 130.76, 130.08, 129.85, 124.91, 124.44, 122.13, 113.81, 111.18, 69.05, 55.21, 55.00, 52.75; IR (ATR): *ν*/cm^{-1} 3076, 3062, 2999, 2949, 2927, 2899, 2826, 1608, 1580, 1507, 1458, 1437, 1361, 1303, 1278, 1253, 1177, 1109, 1038, 932, 893, 866, 837, 822, 804, 782, 752, 685, 669, 656, 583, 531; LR-MS (FD) m/z (%): 1190.48 (6), 1189.48 (18), 1188.47 (46), 1187.47 (89), 1186.47 (M^+, bp), 594.24 (5), 593.74 (10), 593.24 (M^{2+}, 11); HR-MS (FD) Calcd. for $C_{80}H_{66}O_8S$: 1186.44784; Found: 1186.44890 (MS error 0.89 ppm); UV−vis (CH_2Cl_2): λ_{max}/nm (ε/L mol^{-1} cm^{-1}) 425 (4500), 366 (9100), 329 (44700), 277 (77000).

5,7-Bis[bis(4-methoxy-2-methylphenyl)methyl]-12,13-bis[bis(4-methoxy-2-methylphenyl)methylene]-12,13-dihydrodinaphtho[2,3-b:2',3'-d]thiophene (1c-2H)

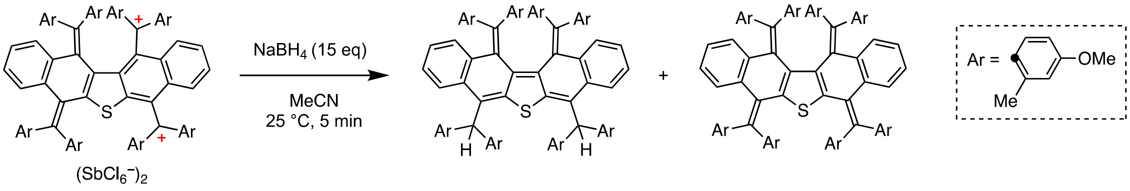

1c^{2+}($SbCl_6^-$)$_2$ **1c-2H** (38%) **1c** (60%)

To a solution of **1c**$^{2+}$($SbCl_6^-$)$_2$ (39.3 mg, 20.0 μmol) in dry MeCN (2.0 mL) was added sodium borohydride ($NaBH_4$) (11.4 mg, 301 μmol). The mixture was stirred at 25 °C for 5 min, and then diluted with water. The whole mixture was extracted with CH_2Cl_2 five times. The combined organic layers were washed with water and brine, and dried over anhydrous Na_2SO_4. After filtration, the solvent was concentrated under reduced pressure. The crude product was purified by column chromatography on silica gel (hexane/EtOAc = 3) to give **1c-2H** (10.0 mg) as a purple solid in 38 % yield. In this reaction, **1c** (15.6 mg) was also obtained in 60% yield upon chromatographic separation.

1c-2H; Mp: 290-292 °C; ^{1}H NMR (400 MHz, DMSO-d_6, 398 K): δ/ppm 8.01-7.55 (2H, m), 7.12-6.73 (4H, m), 6.80 (4H, brd, J = 8.4 Hz), 6.68 (2H, brs), 6.66 (2H, brd, J = 8.4 Hz), 6.61 (4H, brd, J = 8.4 Hz), 6.55 (4H, brs), 6.58-6.20 (2H, m), 6.42 (4H, brs), 6.35 (2H, s), 5.94 (2H, s), 5.22 (2H, s), 3.75 (6H, s), 3.72 (6H, brs), 3.66 (6H, s), 3.16 (6H, s), 2.06 (6H, s), 2.05 (6H, s), 1.92 (6H, s) 1.49 (6H, s); ^{13}C NMR could not be recorded due to inevitable broadening of the signals caused by the existence of multiple conformers.; IR (ATR): ν/cm^{-1} 3050, 2994, 2950, 2940, 2909, 2835, 1603, 1576, 1493, 1464, 1456, 1442, 1378, 1302, 1289, 1228, 1197, 1160, 1104, 1042, 996, 935, 866, 856, 844, 823, 812, 790, 756, 725, 675, 621, 604, 584, 556, 523, 474; LR-MS (FD) m/z (%): 1302.60 (7), 1301.60 (23), 1300.60 (53), 1299.59 (98), 1298.59 (M$^+$, bp); HR-MS (FD) Calcd. for $C_{88}H_{82}O_8S$: 1298.57304; Found: 1298.57174 (MS error 1.00 ppm); Elemental Analysis Calcd. (%) for $C_{88}H_{82}O_8S$: C 81.33, H 6.36; Found: C 80.94, H 6.59; UV−vis (CH_2Cl_2): λ_{max}/nm (ε/L mol^{-1} cm^{-1}) 583 (14900), 329 (21800), 278 (39700).

11,11,12,12-Tetrakis(2-fluoro-4-methoxyphenyl)-9,10-anthraquinodimethane (I-a)

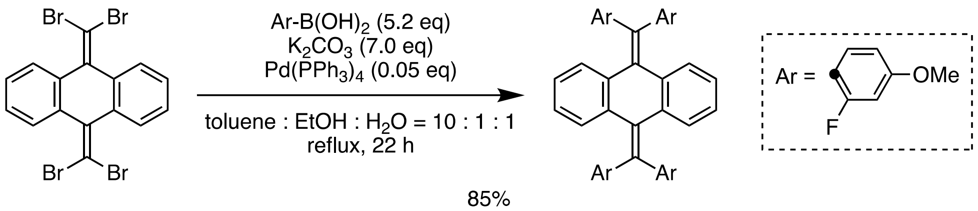

I-a

A mixture of 11,11,12,12-tetrabromo-9,10-anthraquinodimethane[2] (621 mg, 1.19 mmol), 2-fluoro-4-methoxyphenylboronic acid (1.06 g, 6.25 mmol), K_2CO_3 (1.16 g, 8.40 mmol) and $Pd(PPh_3)_4$ (69.0 mg, 59.7 µmol) in toluene (12 mL), EtOH (1.2 mL), and H_2O (1.2 mL) was heated at reflux for 22 h. After cooling to 25 °C, the mixture was diluted with water and extracted with EtOAc five times. The combined organic layers were washed with water and brine, and dried over anhydrous Na_2SO_4. After filtration, the solvent was concentrated under reduced pressure. The crude product was purified by column chromatography on silica gel (hexane/EtOAc = 2) to give **I-a** (713 mg) as a white solid in 85 % yield.

I-a; Mp: 288-293 °C (decomp.); ^{1}H NMR (400 MHz, $CDCl_3$, 353 K): δ/ppm 7.35 (4H, brs), 7.09 (4H, dd, J = 3.4, 5.8 Hz), 6.79 (4H, dd, J = 3.4, 5.8 Hz), 6.61 (4H, d, J = 8.4 Hz), 6.54 (4H, dd, J = 8.4 Hz), 3.73 (12H, s); ^{13}C NMR (100 MHz, $CDCl_3$, 353 K): δ/ppm 160.25 (d, $J_{C\text{-}F}$ = 240.4 Hz), 160.11 (d, $J_{C\text{-}F}$ = 10.8 Hz), 140.08, 137.14, 132.21, 126.71, 126.25, 125.61, 122.43 (d, $J_{C\text{-}F}$ = 17.3 Hz), 109.99, 101.65 (d, $J_{C\text{-}F}$ = 26.3 Hz), 55.42; IR (ATR): ν/cm^{-1} 3071, 3006, 2965, 2936, 2911, 2836, 1619, 1573, 1505, 1457, 1441, 1427, 1311, 1288, 1263, 1244, 1229, 1191, 1152, 1116, 1102, 1030, 946, 933, 854, 839, 805, 769, 726, 718, 668, 658, 631, 577, 542, 501; LR-MS(FD) m/z (%): 702.26 (13), 701.26 (49), 700.25 (M^+, bp), 350.63 (8), 350.12 (M^{2+}, 16); HR-MS (FD) Calcd. for $C_{44}H_{32}F_4O_4$: 700.22367; Found: 700.22293 (MS error 1.06 ppm); UV−vis (CH_2Cl_2): λ_{max}/nm (ε/L mol^{-1} cm^{-1}) 310 (sh, 17800), 283 (20500).

Anthracene-9,10-diyl-bis[bis(2-fluoro-4-methoxyphenyl)methylium] bis(hexachloroantimonate) [I-a^{2+}($SbCl_6^-$)$_2$]

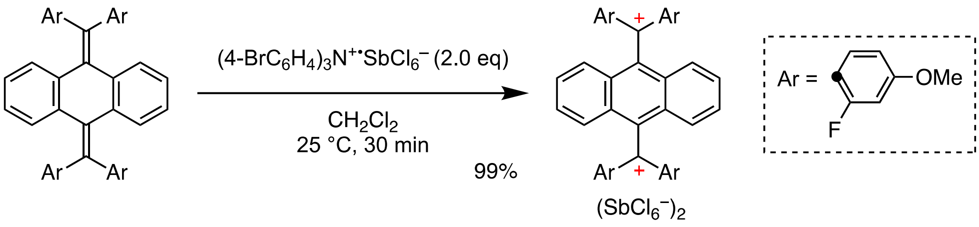

I-a

I-a^{2+}($SbCl_6^-$)$_2$

To a solution of **I-a** (98.0 mg, 140 μmol) in dry CH_2Cl_2 (2.8 mL) was added tris(4-bromophenyl)aminium hexachloroantimonate (228 mg, 279 μmol) at 25 ºC to generate a deep red solution, and the mixture was stirred at 25 ºC for 30 min. The addition of dry ether led to precipitation of the dication salt. The solvent was decantated and the resulting precipitates were washed with dry ether five times, and collected by filtration to give **I-a**$^{2+}$($SbCl_6^-$)$_2$ (190 mg) as a dark red powder in 99% yield.

I-a^{2+}($SbCl_6^-$)$_2$; Mp: 198-208 ºC (decomp.); ^{1}H NMR (400 MHz, CD_3CN): δ/ppm 7.68 (4H, dd, J = 3.4, 6.8 Hz), 7.57 (4H, dd, J = 3.4, 6.8 Hz), 7.53-7.43 (4H, m), 7.15 (4H, dd, J = 2.4, 8.4 Hz), 7.02 (4H, dd, J = 2.4, 13.2 Hz), 4.17 (12H, s); ^{13}C NMR (100 MHz, CD_3CN): δ/ppm 181.91, 178.10 (d, $J_{C\text{-}F}$ = 15.0 Hz), 167.98 (d, $J_{C\text{-}F}$ = 271.8 Hz), 143.16, 139.50, 131.31, 129.87, 126.42, 125.88 (d, $J_{C\text{-}F}$ = 10.9 Hz), 116.42, 105.68 (d, $J_{C\text{-}F}$ = 26.1 Hz), 59.61; IR (ATR): ν/cm^{-1} 3077 , 3037, 3000, 2941, 2887, 2848, 1592, 1534, 1482, 1450, 1439, 1377, 1342, 1320, 1291, 1242, 1210, 1188, 1170, 1110, 1062, 1013, 1001, 948, 933, 853, 846, 817, 789, 764, 741, 736, 712, 680, 650, 626, 607, 592, 563, 551, 527, 508, 470, 453; LR-MS(FD) m/z (%): 702.22 (13), 701.22 (50), 700.21 (bp, [M^{2+}+e^-]$^+$), 350.61 (12), 350.11 (12, M^{2+}); HR-MS (FD) Calcd. for $C_{44}H_{32}F_4O_4$: 700.22367; Found: 700.22558 (MS error 2.73 ppm); UV−vis−NIR (CH_2Cl_2): λ_{max}/nm (ε/L mol^{-1} cm^{-1}) 732 (12400), 548 (116600), 260 (96000).

NMR spectra of new compounds (Figures S1-S14)

(a)

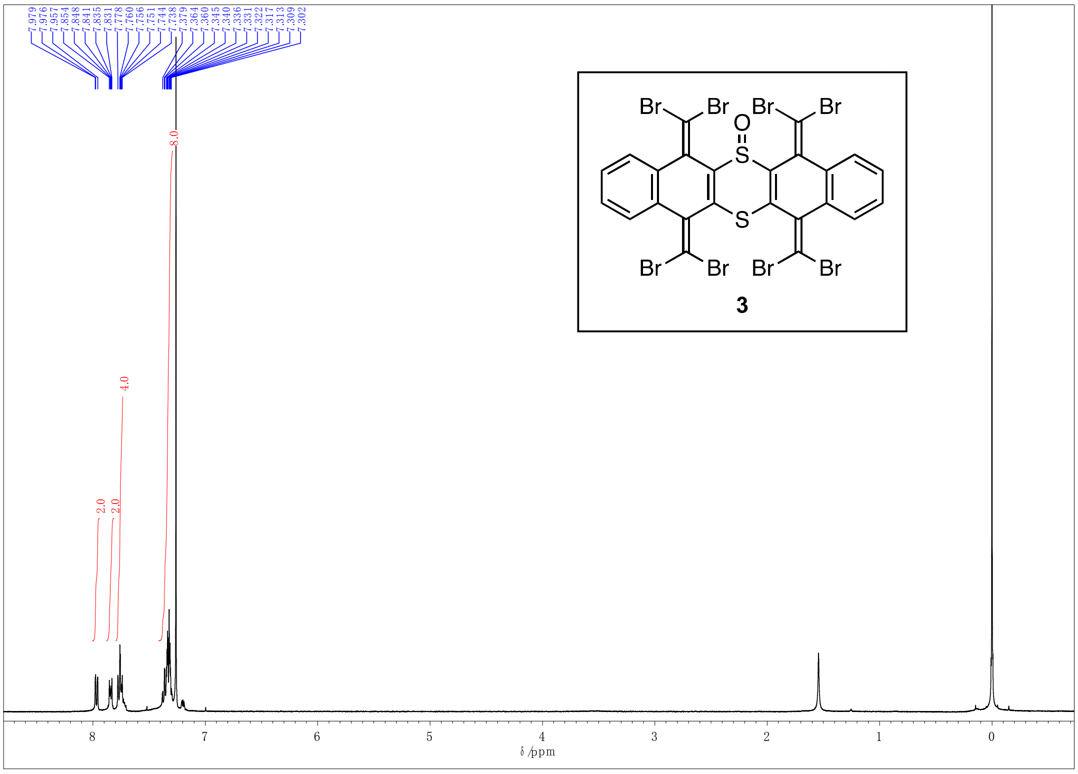

(b)

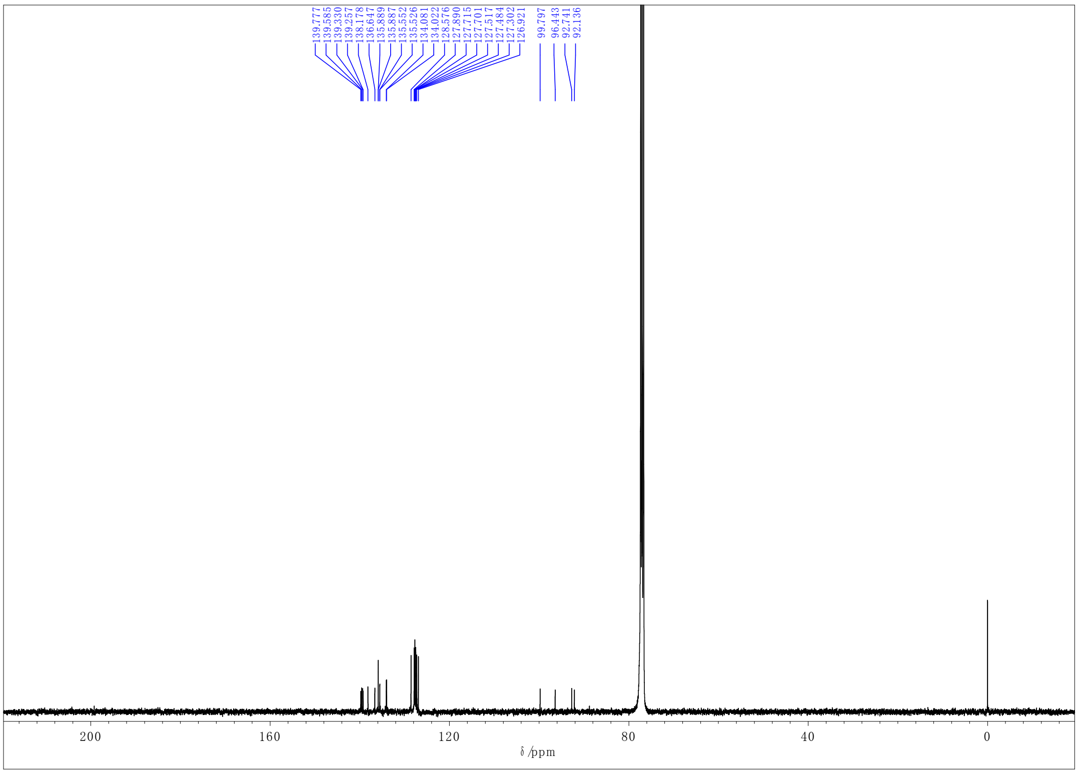

Figure S1 | NMR charts of 3. (a) ^{1}H NMR (400 MHz) and (b) ^{13}C NMR (100 MHz) spectra in $CDCl_3$.

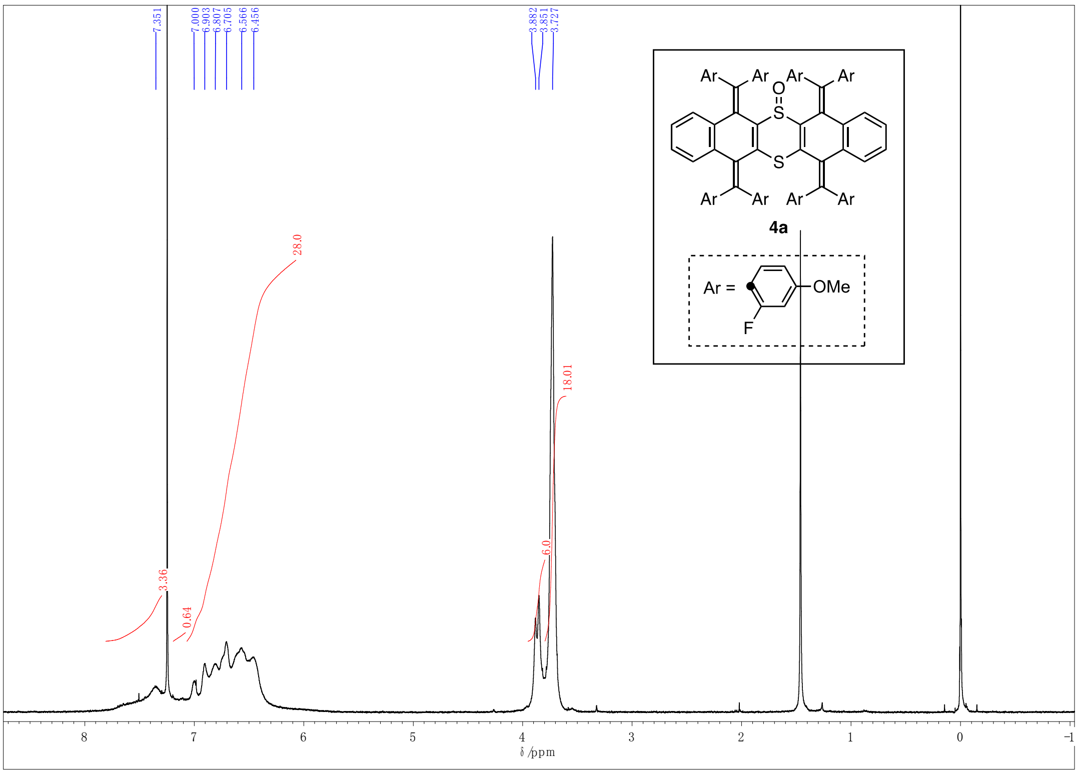

Figure S2 | NMR chart of 4a. ^{1}H NMR (400 MHz) spectrum in $CDCl_3$ at 323 K.

(a)

(b)

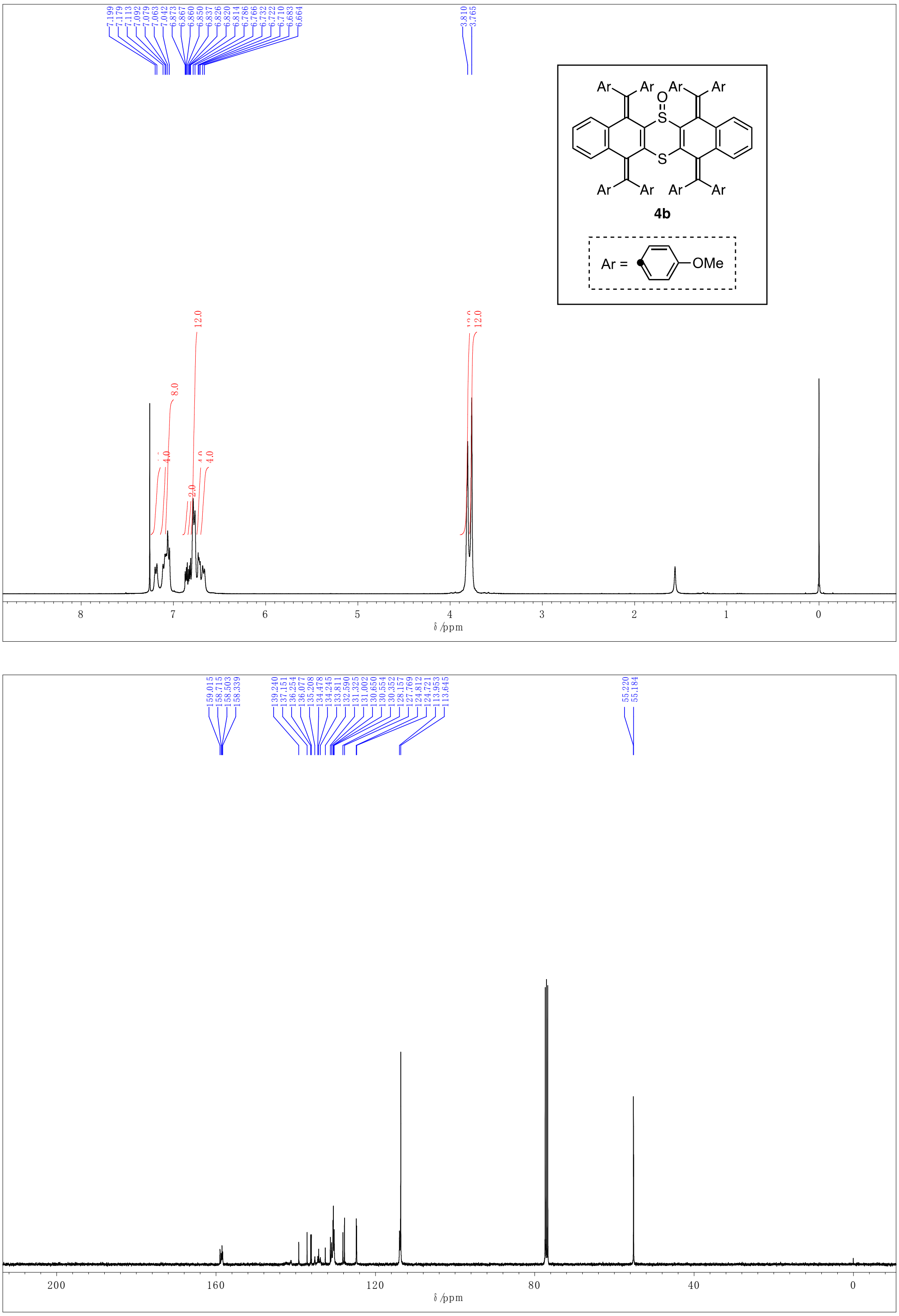

Figure S3 | NMR charts of 4b. (a) ^{1}H NMR (400 MHz) and (b) ^{13}C NMR (100 MHz) spectra in $CDCl_3$.

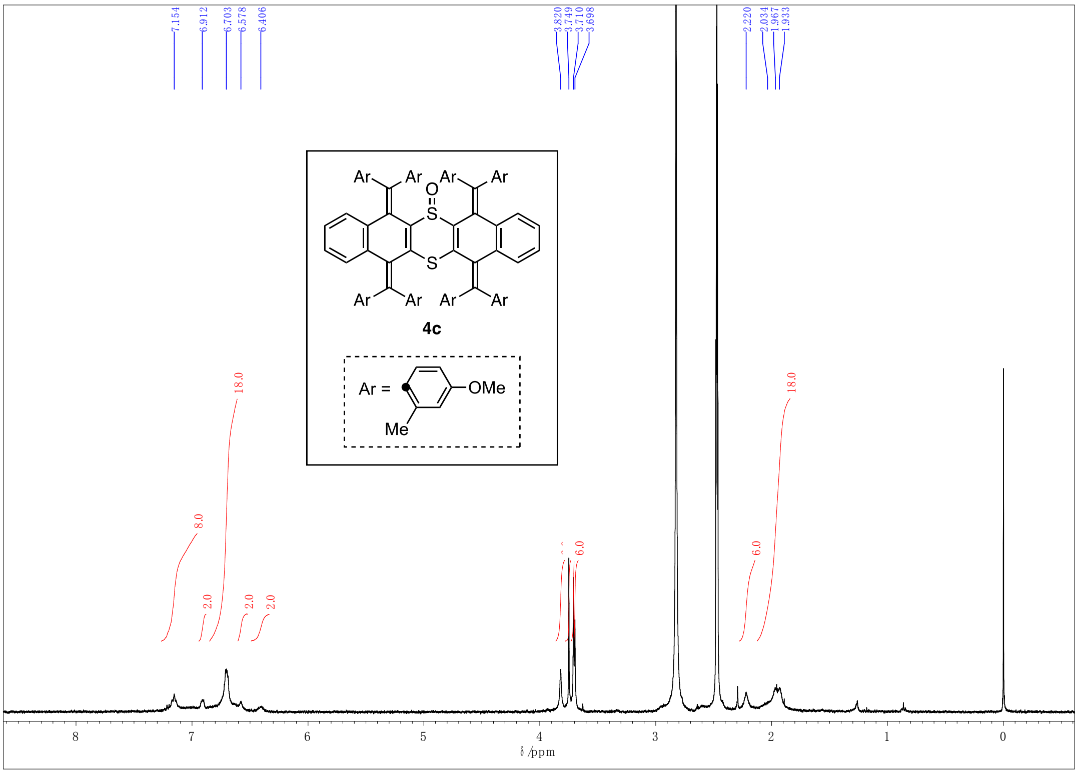

Figure S4 | NMR chart of 4c. ^{1}H NMR (400 MHz) spectrum in DMSO-d_6 at 383 K.

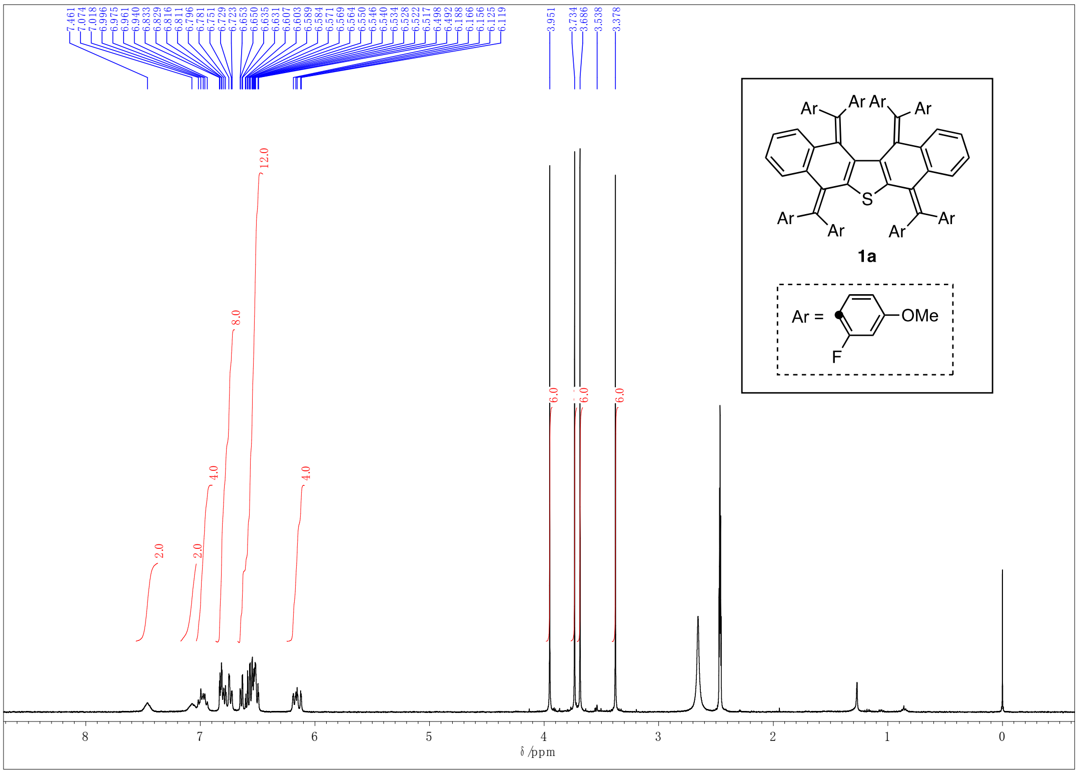

Figure S5 | NMR chart of 1a. ^{1}H NMR (400 MHz) spectrum in DMSO-d_6 at 413 K.

(a)

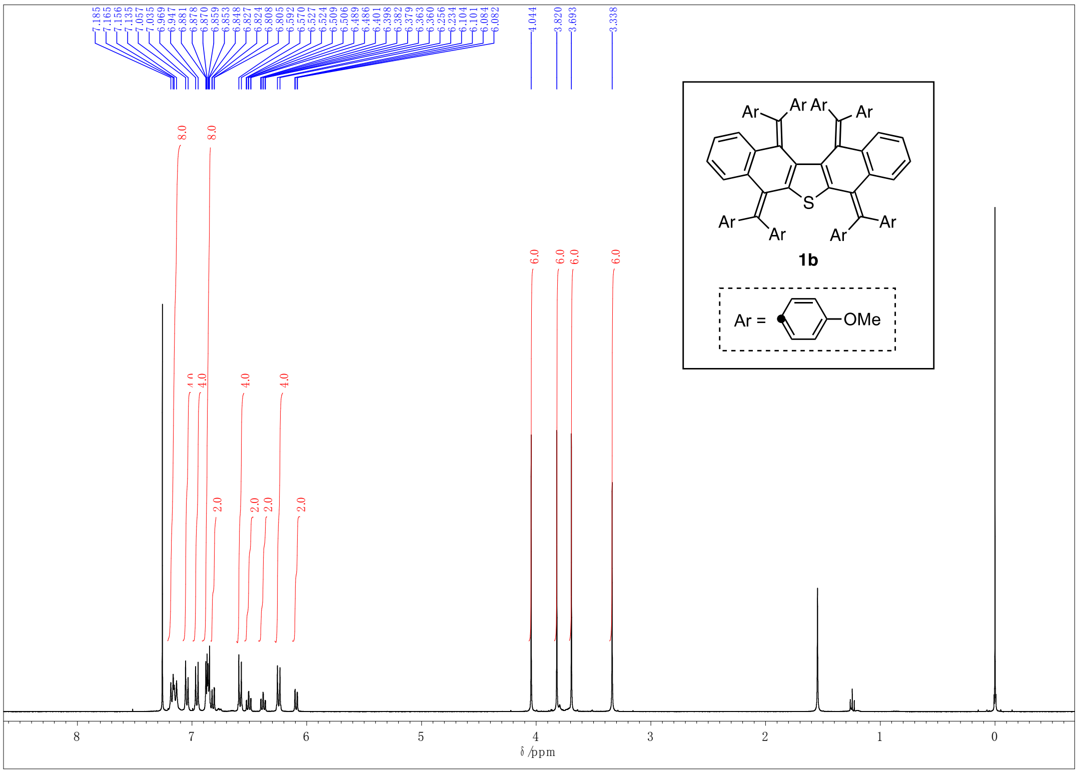

(b)

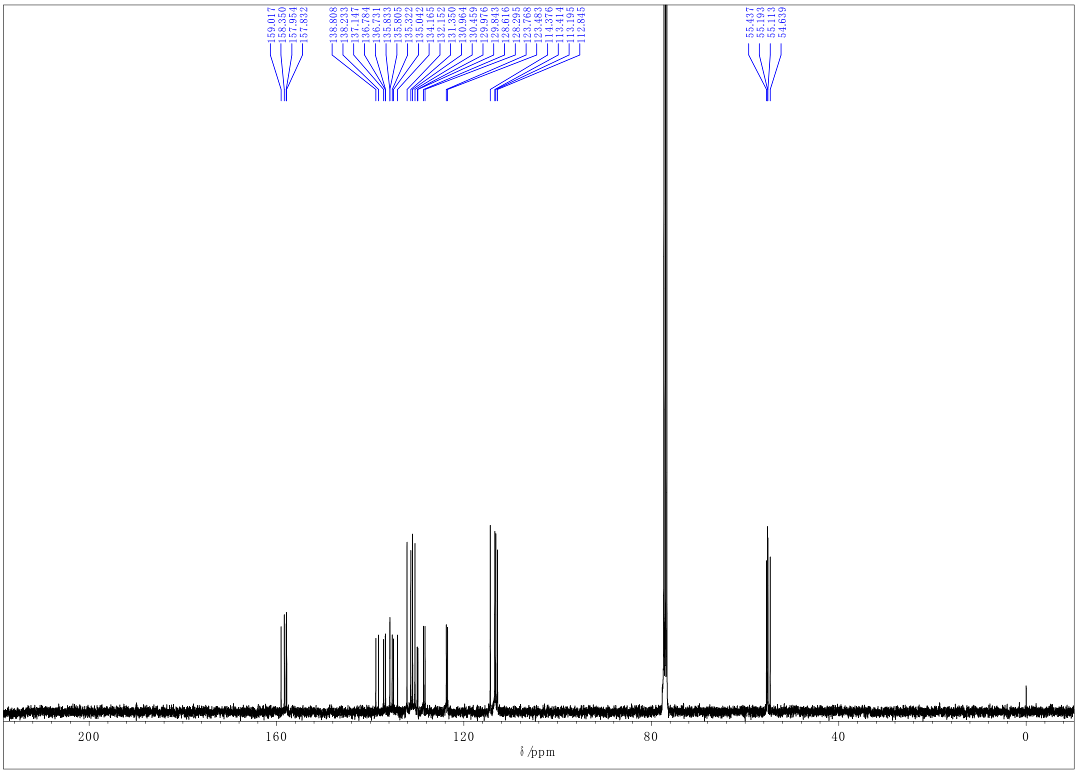

Figure S6 | NMR charts of 1b. (a) ^{1}H NMR (400 MHz) and (b) ^{13}C NMR (100 MHz) spectra in $CDCl_3$.

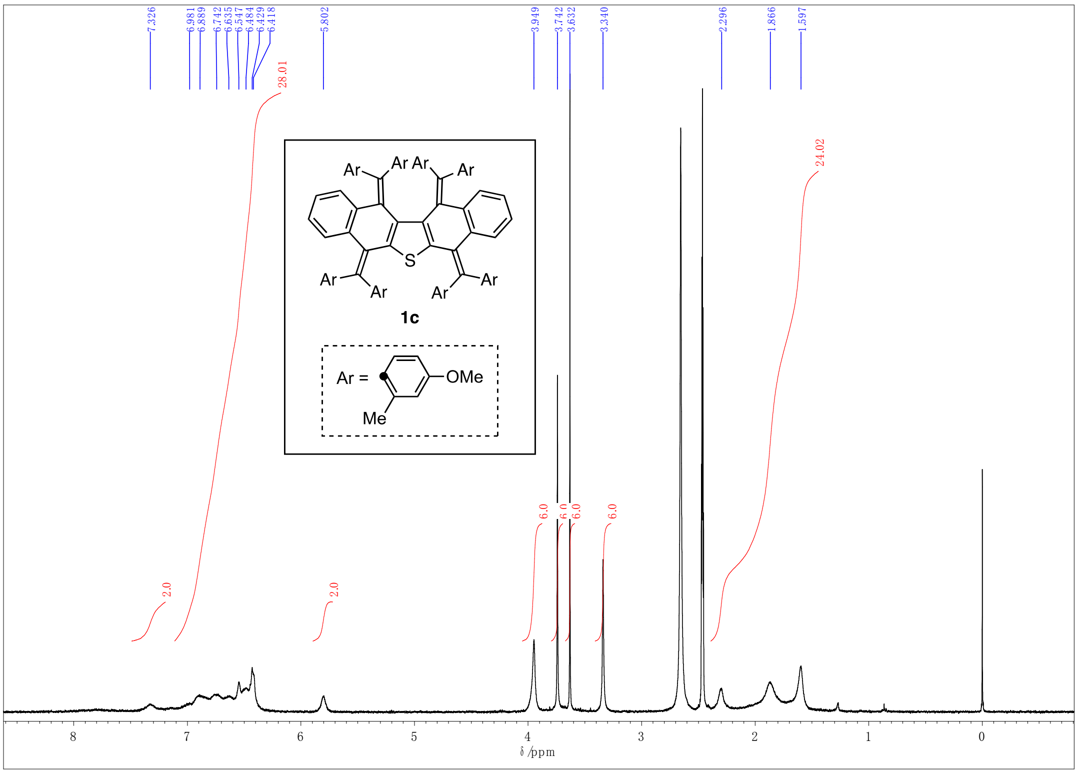

Figure S7 | NMR chart of 1c. ^{1}H NMR (400 MHz) spectrum in DMSO-d_6 at 411 K.

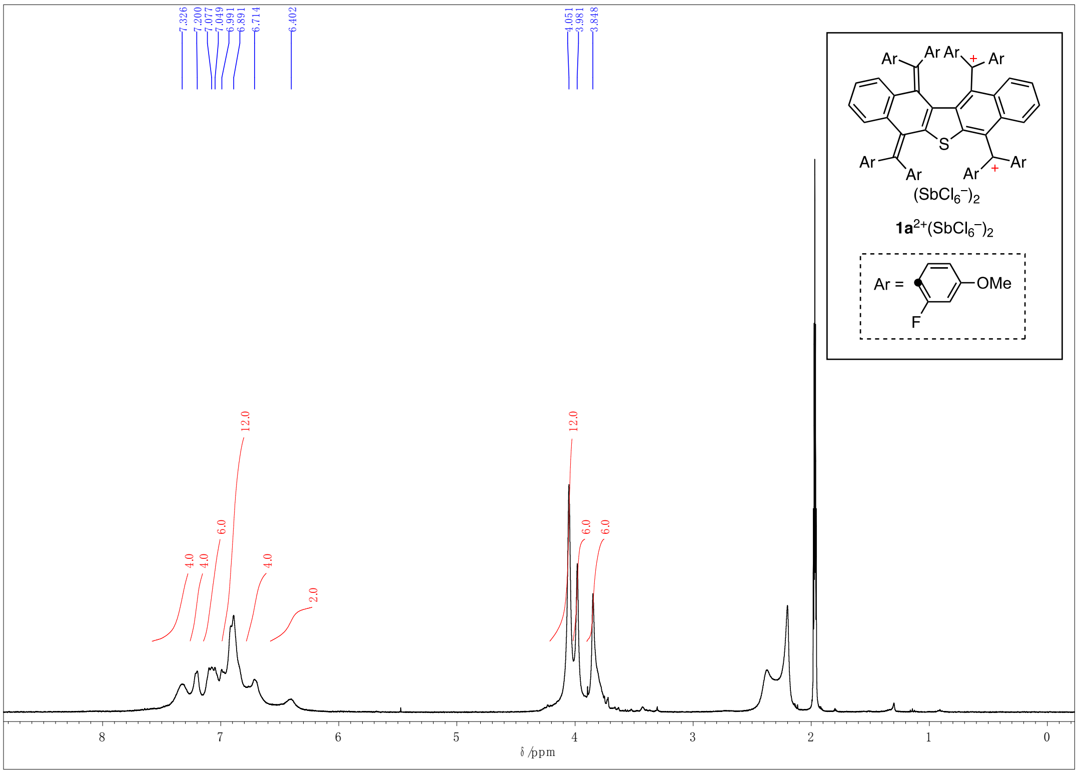

Figure S8 | NMR chart of $1a^{2+}(SbCl_6^{-})_2$. ^{1}H NMR (400 MHz) spectrum in CD_3CN.

(a)

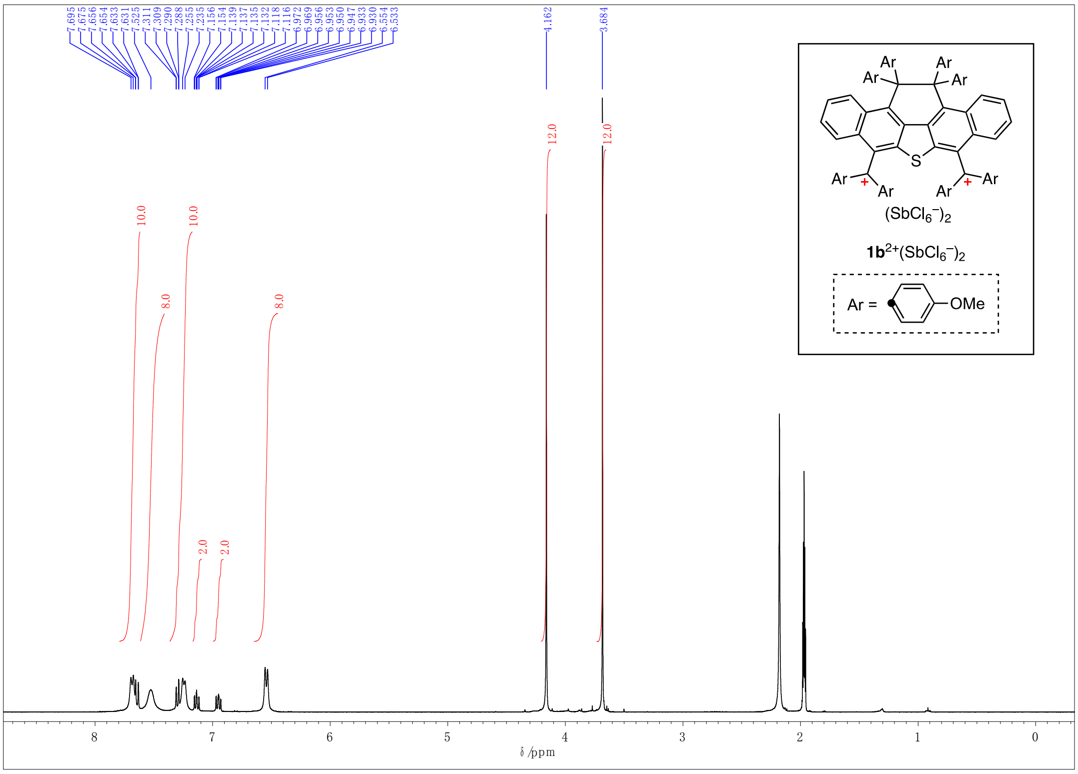

(b)

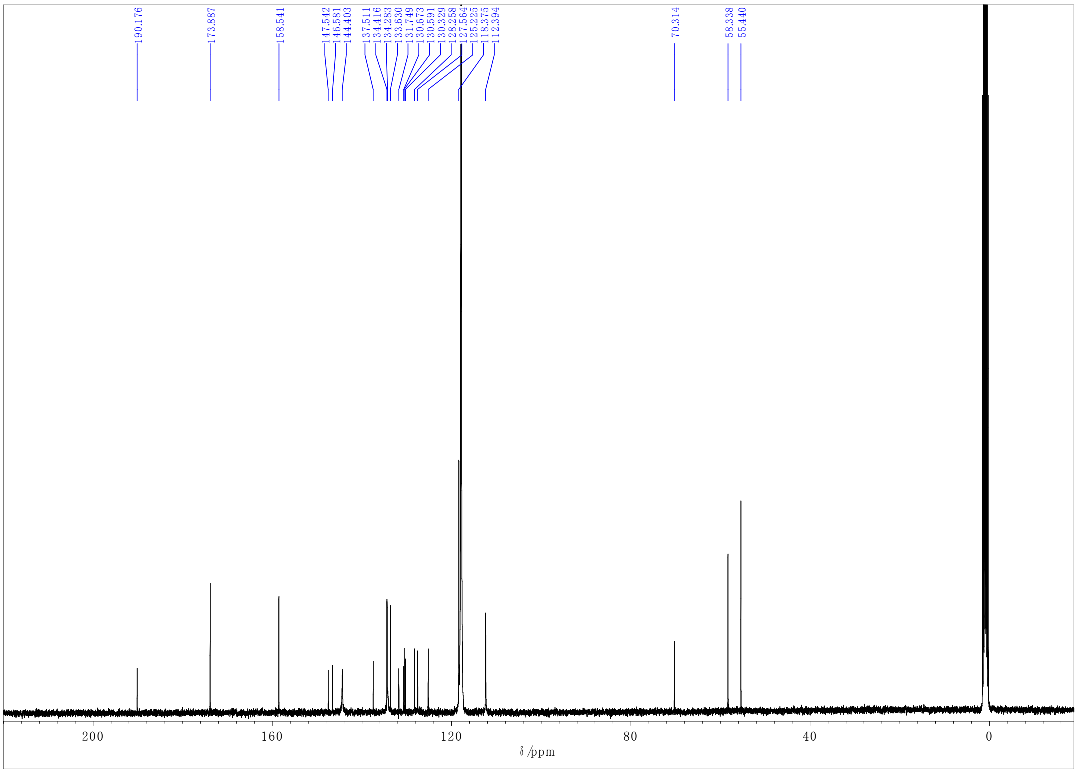

Figure S9 | NMR charts of $\mathbf{1b^{2+}(SbCl_6^-)_2}$. (a) ^{1}H NMR (400 MHz) and (b) ^{13}C NMR (100 MHz) spectra in CD_3CN.

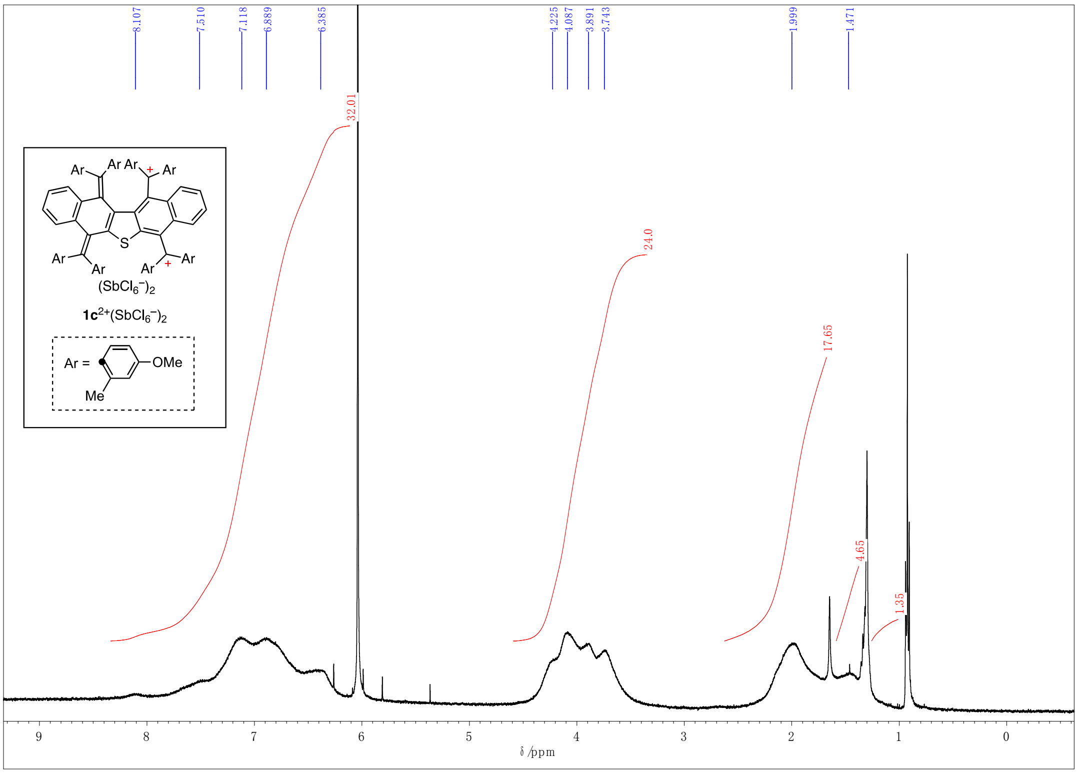

Figure S10 | NMR chart of $1c^{2+}(SbCl_6^-)_2$. ^{1}H NMR (400 MHz) spectrum in 1,1,2,2-tetrachloroethane-d_2 at 293 K.

(a)

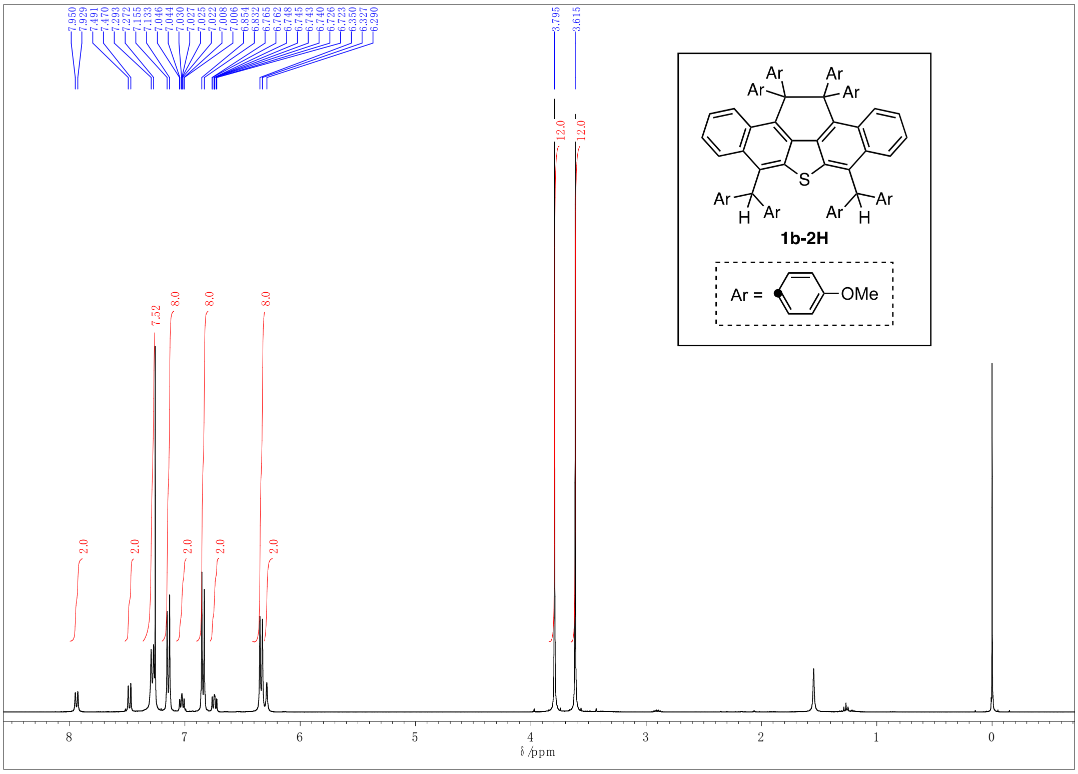

(b)

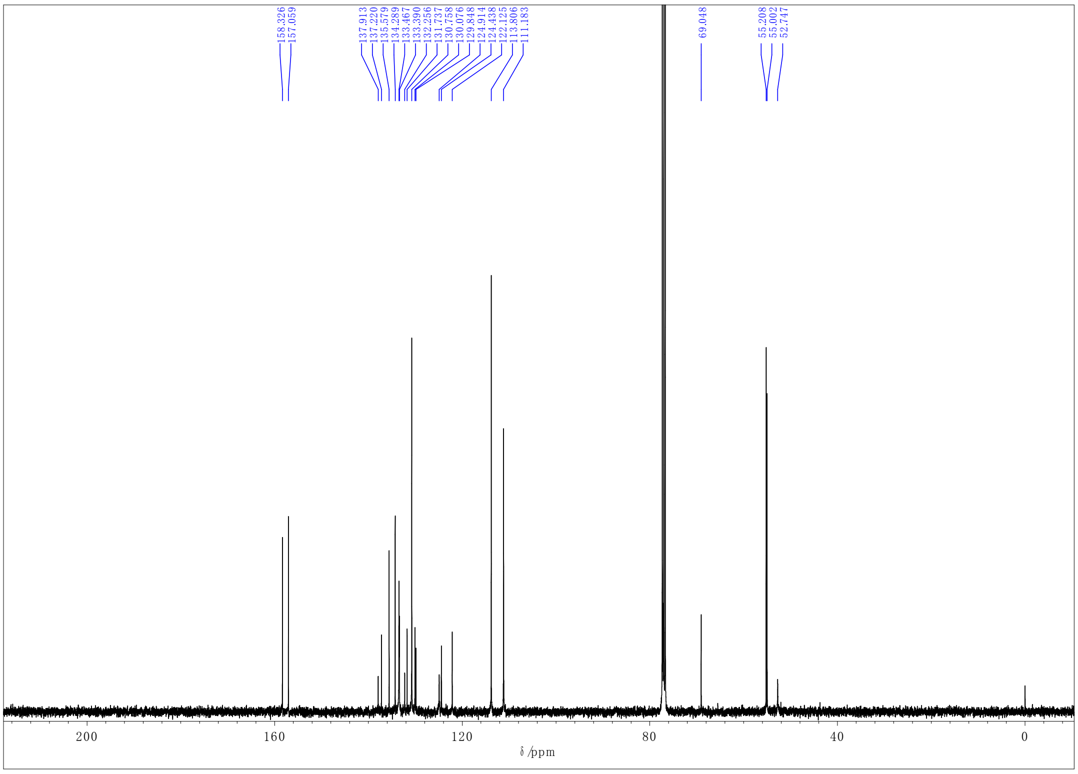

Figure S11 | NMR charts of 1b-2H. (a) ^{1}H NMR (400 MHz) and (b) ^{13}C (100 MHz) NMR spectra in $CDCl_3$.

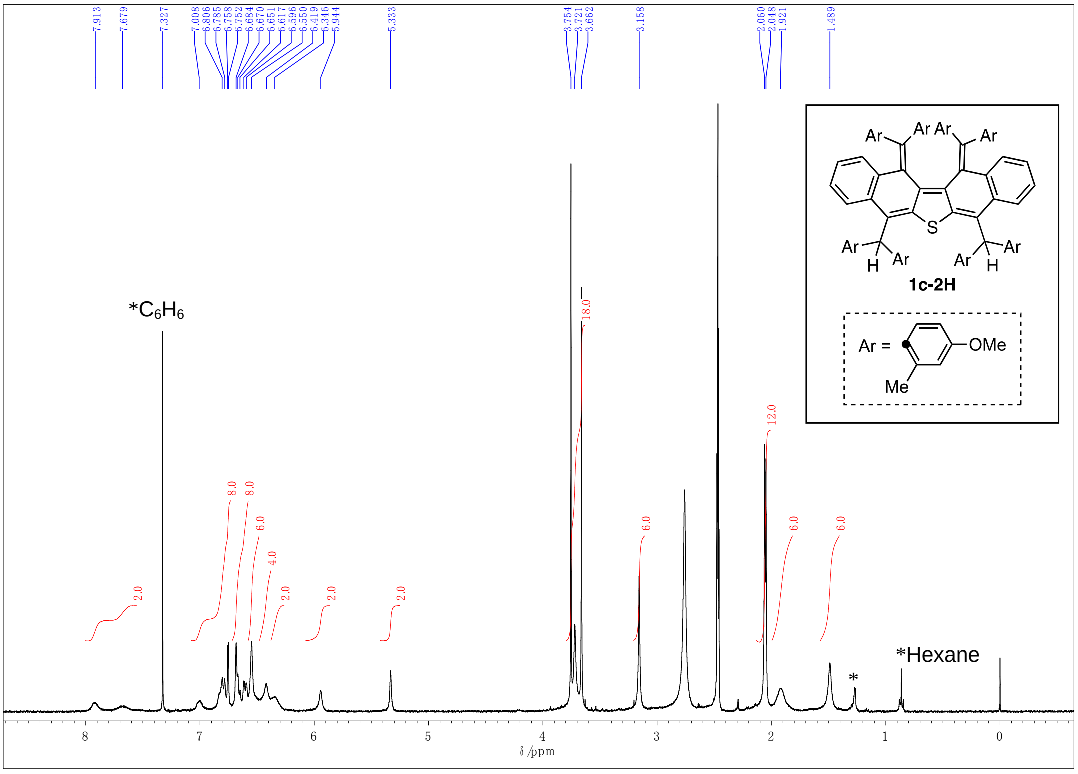

Figure S12 | NMR chart of 1c-2H. ^{1}H NMR (400 MHz) spectrum in DMSO-d_6 at 398 K.

(a)

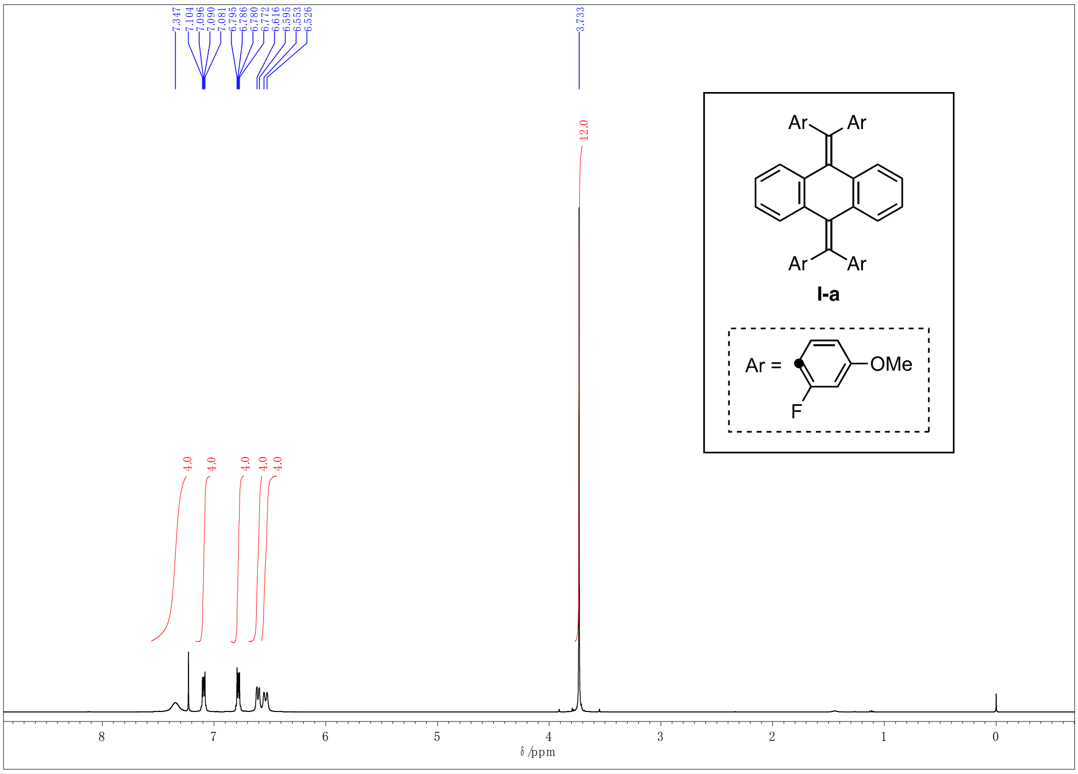

(b)

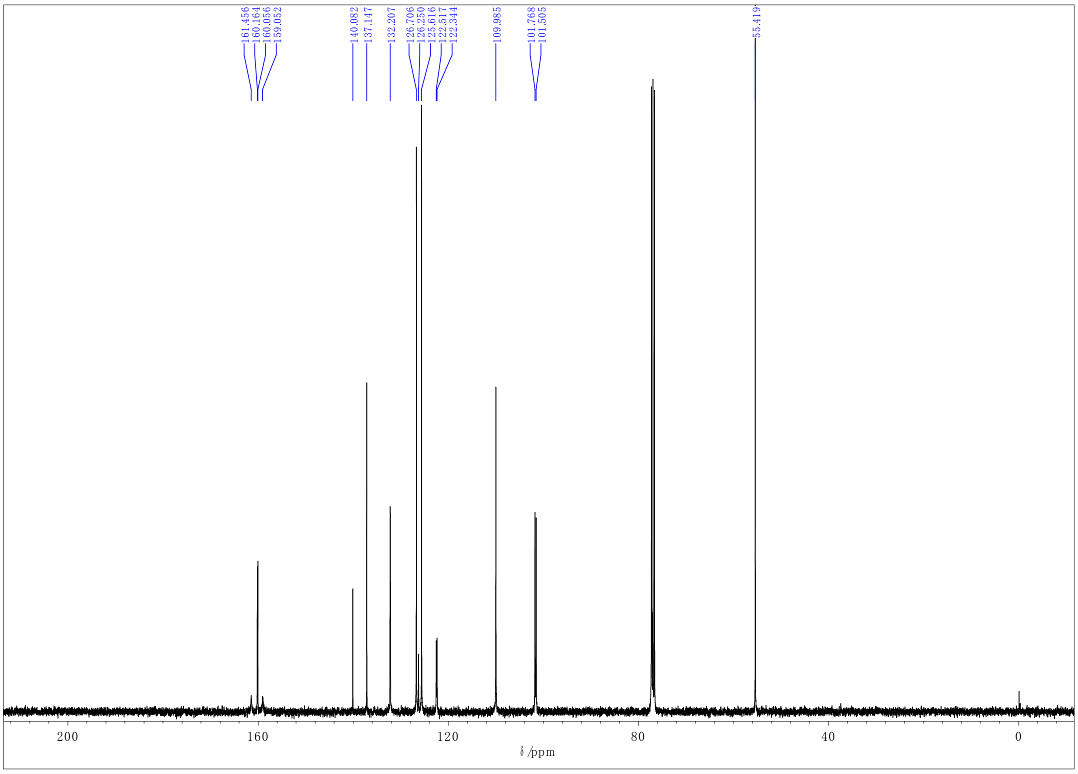

Figure S13 | NMR charts of I-a. (a) ^{1}H NMR (400 MHz) and (b) ^{13}C NMR (100 MHz) spectra in $CDCl_3$ at 353 K.

(a)

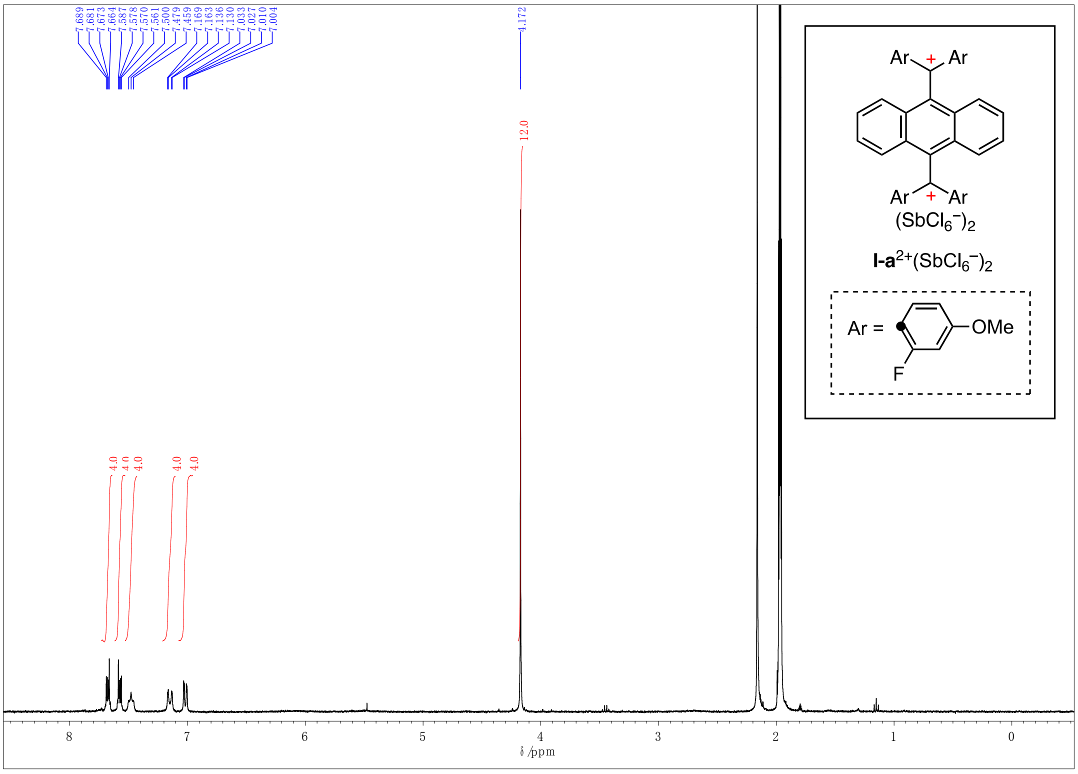

(b)

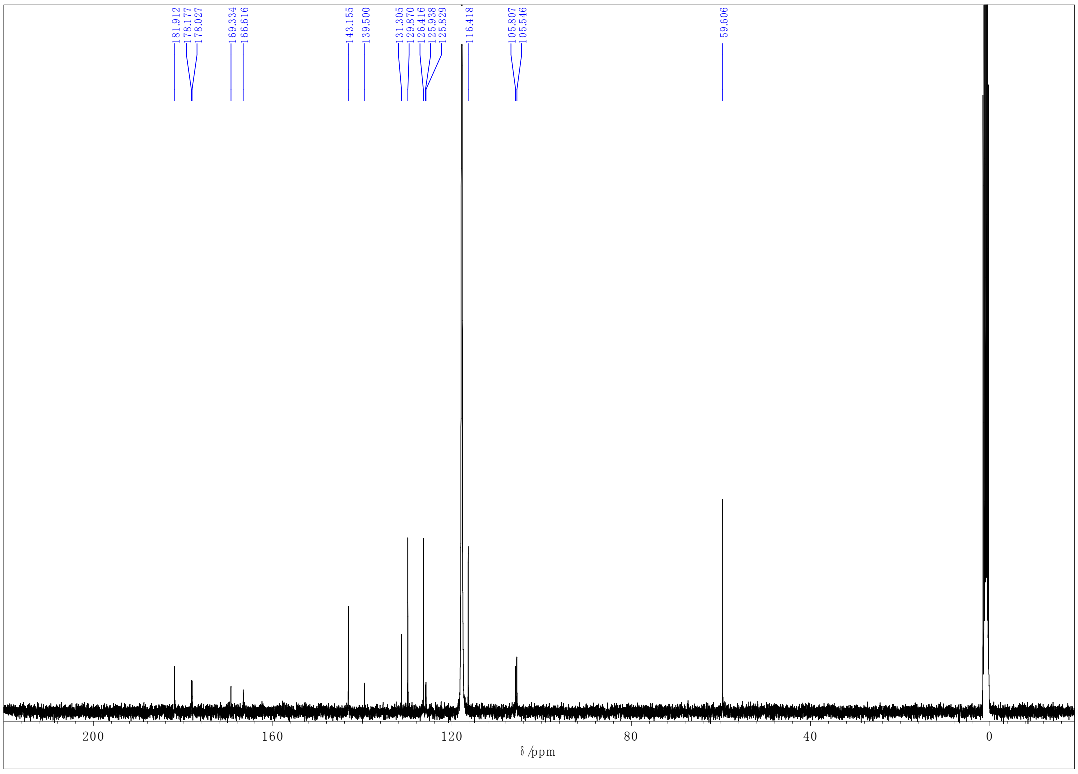

Figure S14 | NMR charts of I-a^{2+}(SbCl$_6^-$)$_2$. (a) ^{1}H NMR (400 MHz) and (b) ^{13}C NMR (100 MHz) spectra in CD_3CN.

Redox Properties

Differential pulse voltammetry (Figure S15)

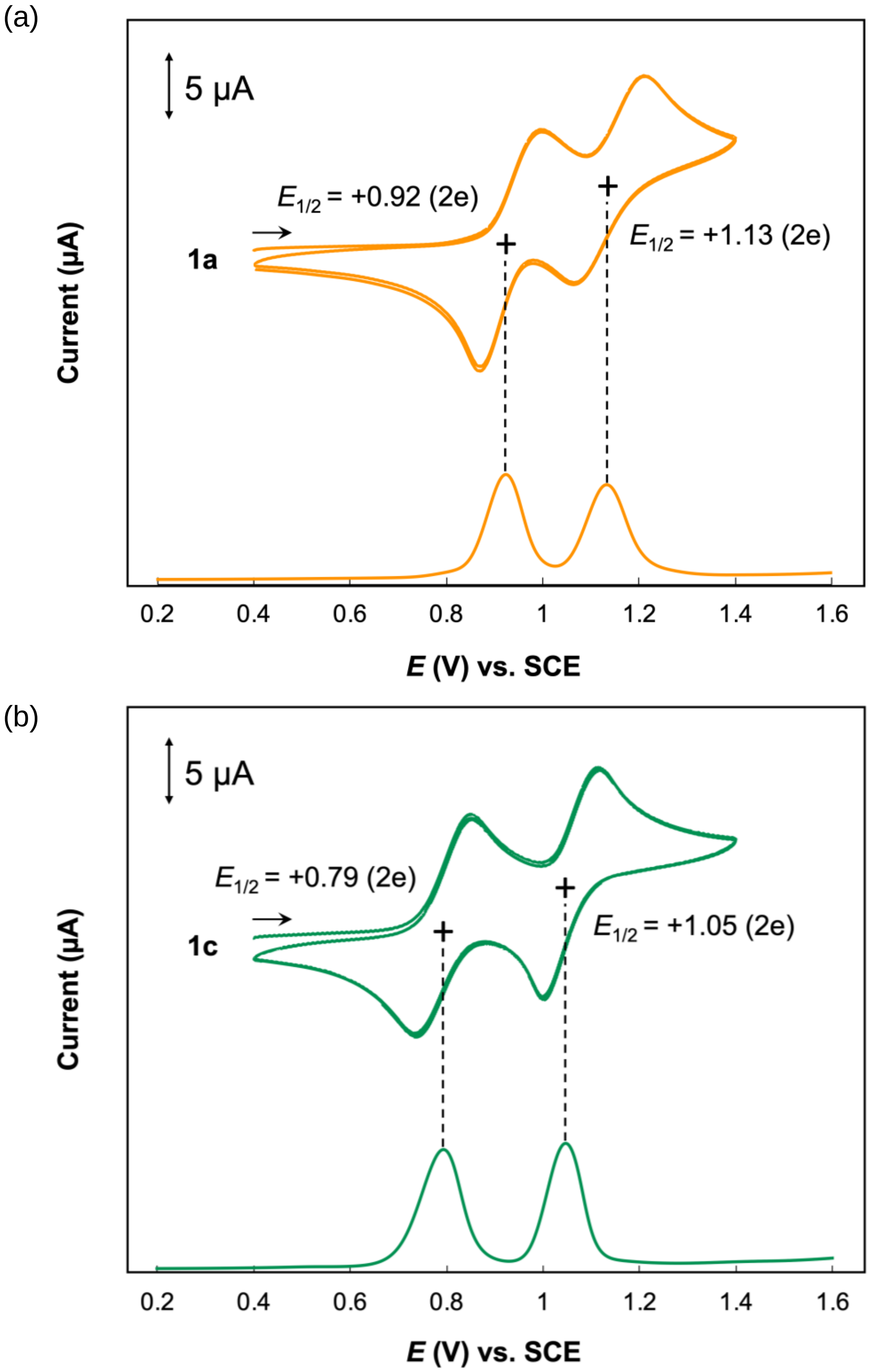

Figure S15 | Voltammetric analyses. Cyclic and differential pulse voltammograms of (a) **1a** and (b) **1c** in CH_2Cl_2 containing 0.1 M Bu_4NBF_4 as a supporting electrolyte (Pt electrode, 298 K). [**1a**: Ar= 2-F-4-$MeOC_6H_3$, **1c**: Ar= 4-MeO-2-MeC_6H_3]

Cyclic voltammetry (Figures S16 and S17)

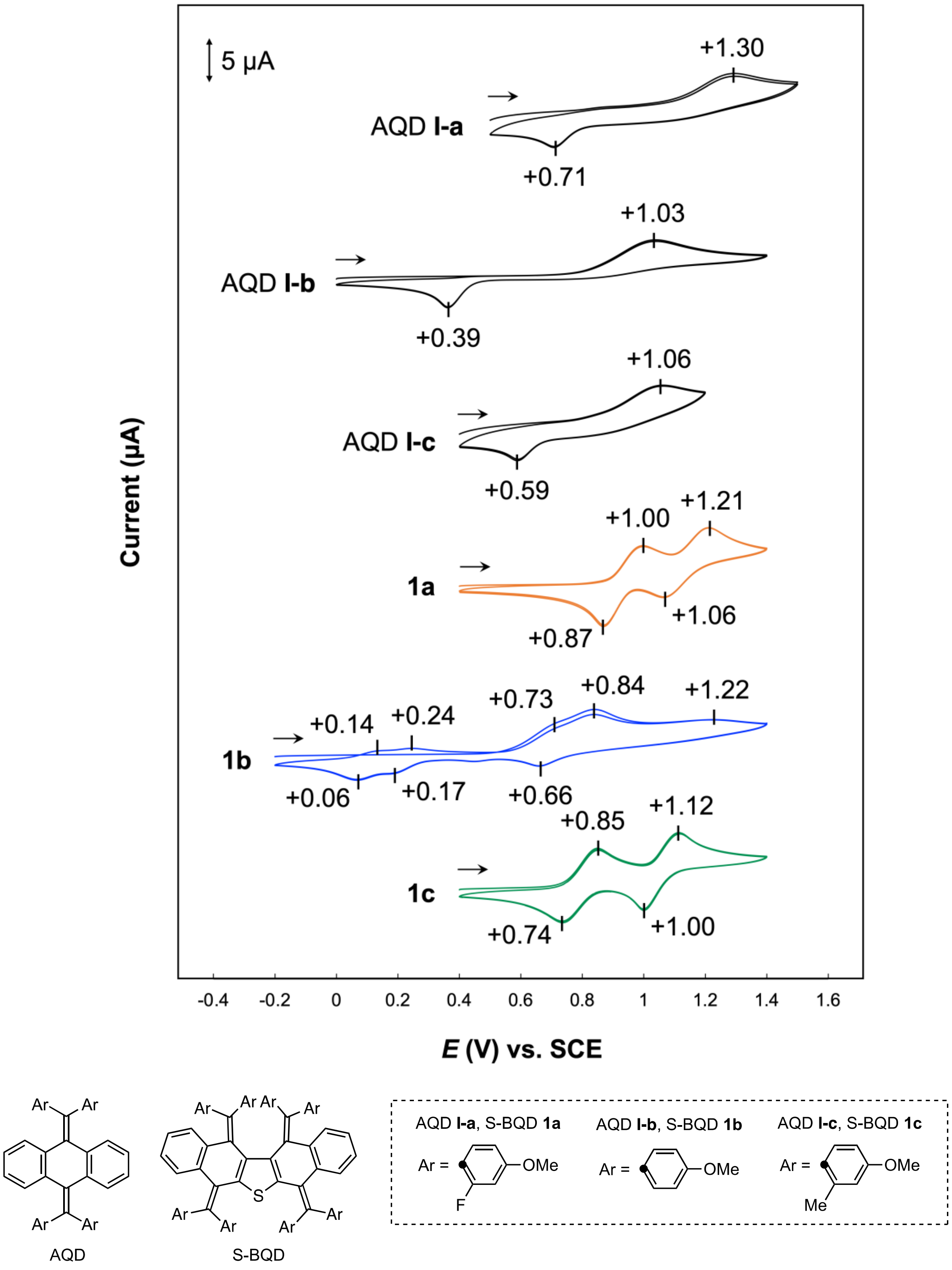

Figure S16 | Redox behavior of AQD and BQD derivatives. Cyclic voltammograms of neutral donors **I-a**, **I-b**, **I-c**, **1a**, **1b**, and **1c** measured in CH_2Cl_2 containing 0.1 M Bu_4NBF_4 as a supporting electrolyte (scan rate 100 mV s^{-1}, Pt electrode, 298 K).

Steric effects of *ortho* substituents on the HOMO levels of neutral donors

The HOMOs of the neutral donor **1a-1c** are mainly distributed in the diarylmethylene moieties, and the HOMO levels are affected by both steric and electronic effects of the *ortho* substituent. Since the steric repulsion of the *ortho* substituent is more pronounced in **1a** with F atoms and **1c** with Me groups than in **1b** with H atoms, the dihedral angles β around the aryl group and methylene moiety become larger, resulting in lower co-planarity (β_{ave}^{calc} **1a**: 52.5° and **1c**: 52.6° vs **1a**: 39.6°). The co-planarity is effective for the π conjugation between the methylene moiety and the methoxyphenyl groups, resulting in the elevated HOMO level in **1b** (Fig. S41). This is consistent with the results of the CV measurements, which showed that **1b** was most easily oxidized due to the highest HOMO level among **1a-1c** (E_{peak}^{ox1}: +0.73 V vs SCE). Neutral donor **1c** has weak electron-donating Me groups at the *ortho*-position on the aryl group, which could electronically contribute to the increase in the HOMO level, but the conjugation between the aryl groups and the exomethylene moieties is not very effective due to the lower co-planarity, resulting in the slightly more positive oxidation potential (E_{peak}^{ox1}: +0.85 V vs SCE for **1c**) than that for **1b**. Compared to these two derivatives **1b** and **1c**, in the case of fluorinated donor **1a**, both the steric and the electronic effects of electron-withdrawing F atoms are dominant, inducing a significant decrease in the HOMO level, and thus the oxidation potential was observed in the most anodic region (E_{peak}^{ox1}: +1.00 V vs SCE). In summary, it is suggested that the oxidation potentials (i.e., HOMO levels) of the neutral donor are affected by both steric and electronic effects of *ortho* substituents, which is in common with our previous study for the reference monomeric AQDs **I**.[3]

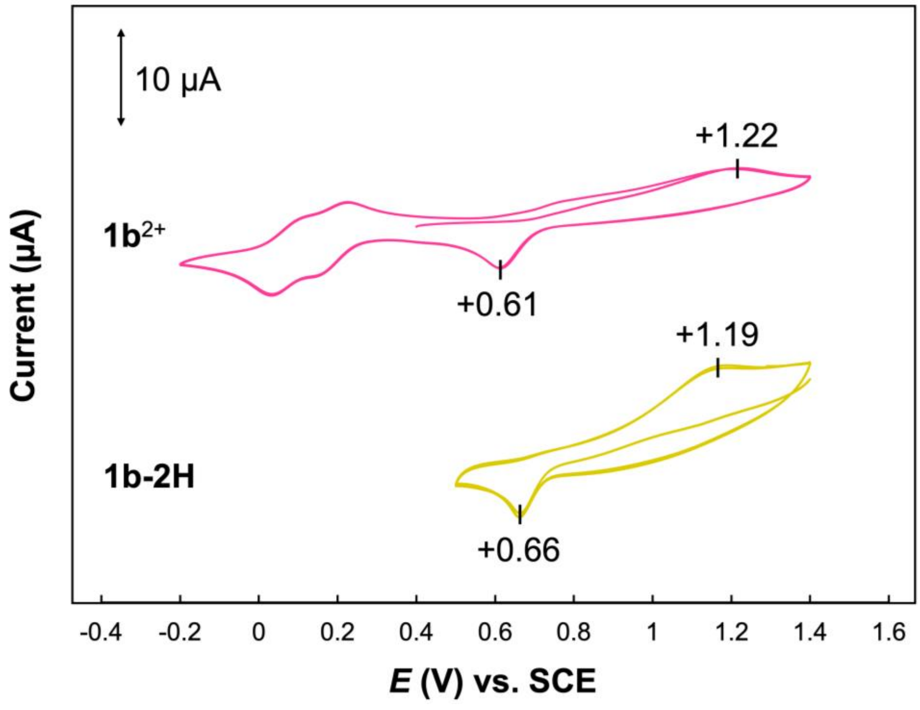

Figure S17 | Redox behavior of σ-bonded species. Cyclic voltammograms of dication $\mathbf{1b}^{2+}(SbCl_6^-)_2$, and hydrid adduct **1b-2H** measured in CH_2Cl_2 containing 0.1 M Bu_4NBF_4 as a supporting electrolyte (scan rate 100 mV s^{-1}, Pt electrode, 298 K).

Spectroscopic Investigation

UV−vis−NIR spectroscopy (Figure S18)

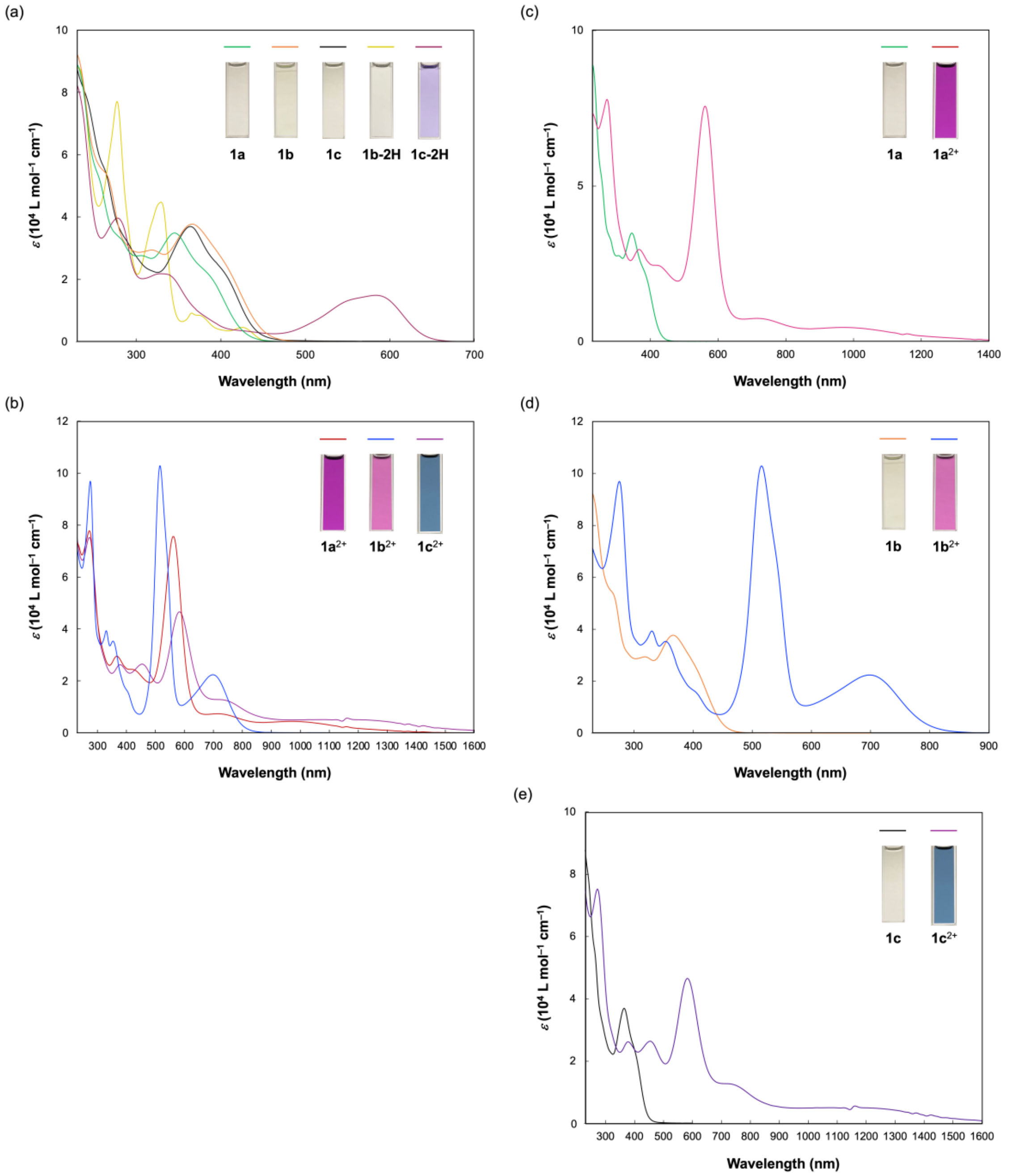

Figure S18 | Electronic absorptions. UV−vis(−NIR) spectra of $(Ar_4QD)_2S$, hydride adducts, and corresponding cationic species in CH_2Cl_2. [**1a**: Ar= 2-F-4-MeOC_6H_3; **1b**: Ar= 4-MeOC_6H_4; **1c**: Ar= 4-MeO-2-MeC_6H_3; Counterion **X**$^-$ = $SbCl_6^-$]

VT-¹H NMR spectroscopy (Figures S19-S21)

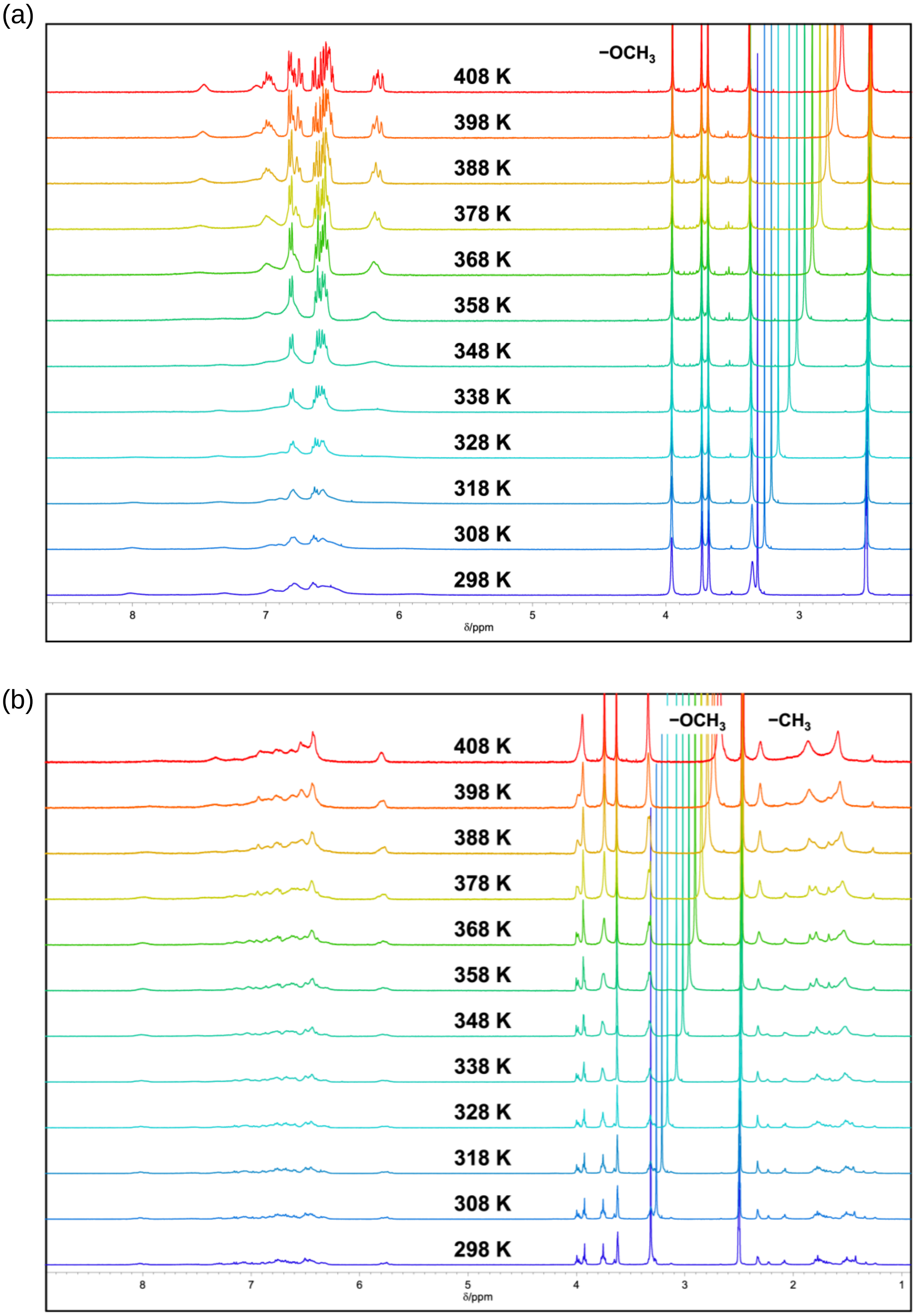

Figure S19 | VT-NMR charts. Changes in ¹H NMR spectra of (a) **1a** and (b) **1c** in DMSO-d_6 (298–408 K).

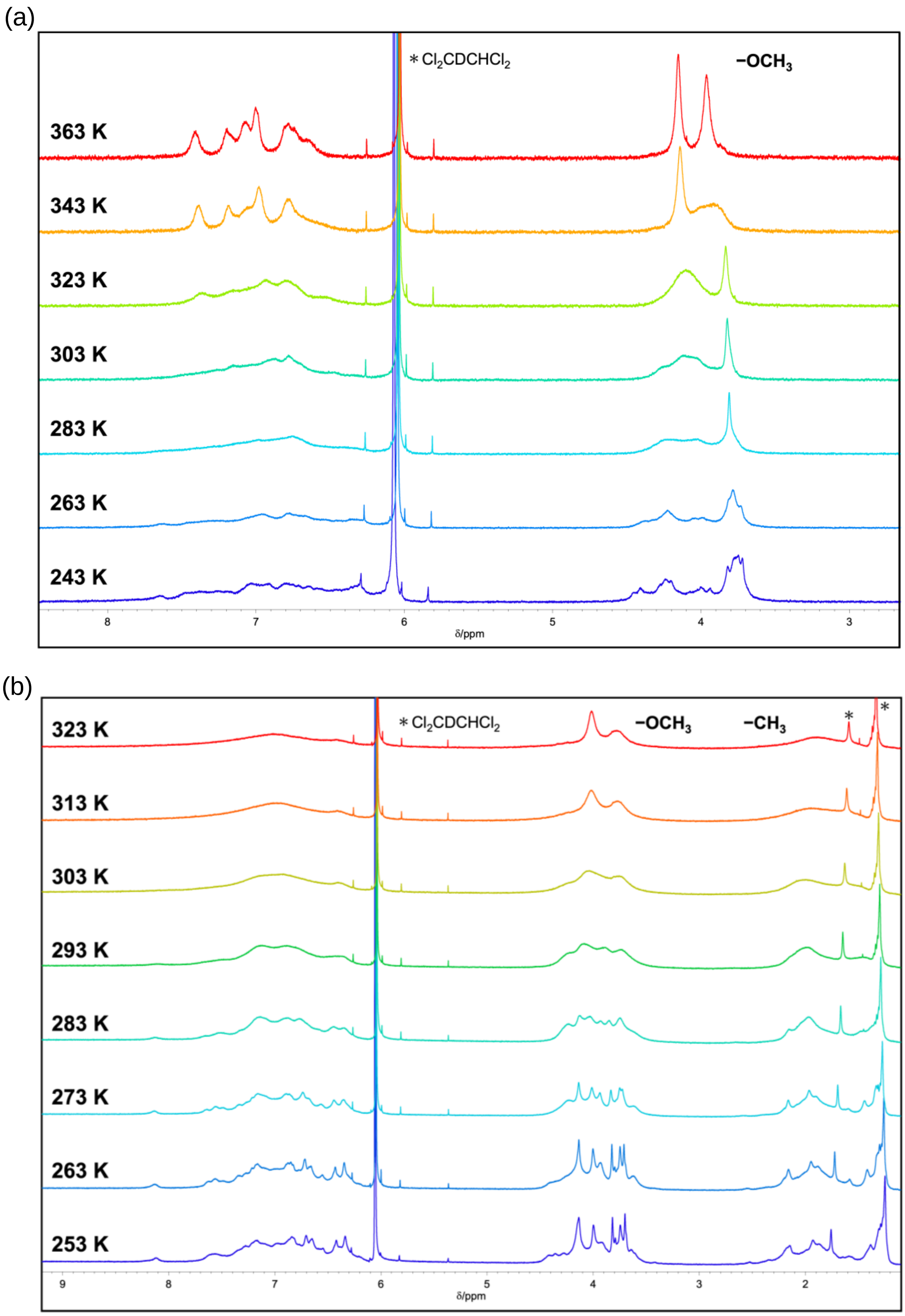

Figure S20 | VT-NMR charts. Changes in ^{1}H NMR spectra of (a) **1a**$^{2+}$ $(SbCl_6^-)_2$ (243–363 K) and (b) **1c**$^{2+}$ $(SbCl_6^-)_2$ (253–323 K) and in 1,1,2,2-tetrachloroethane-d_2.

(a)

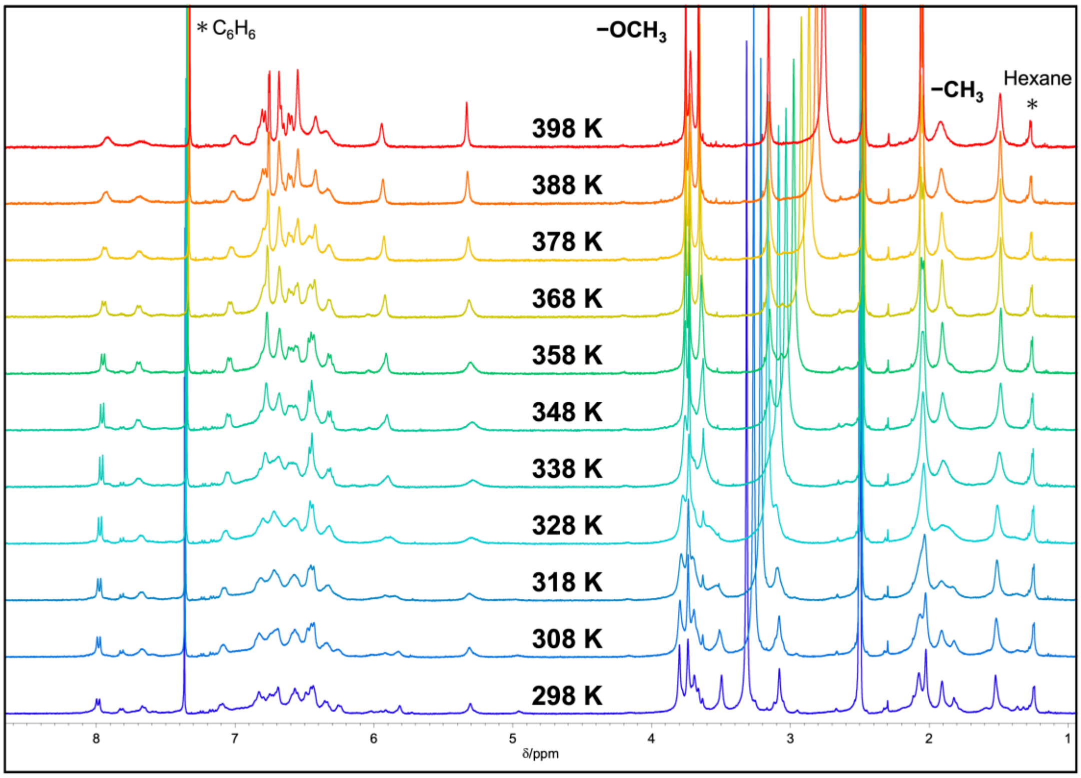

(b)

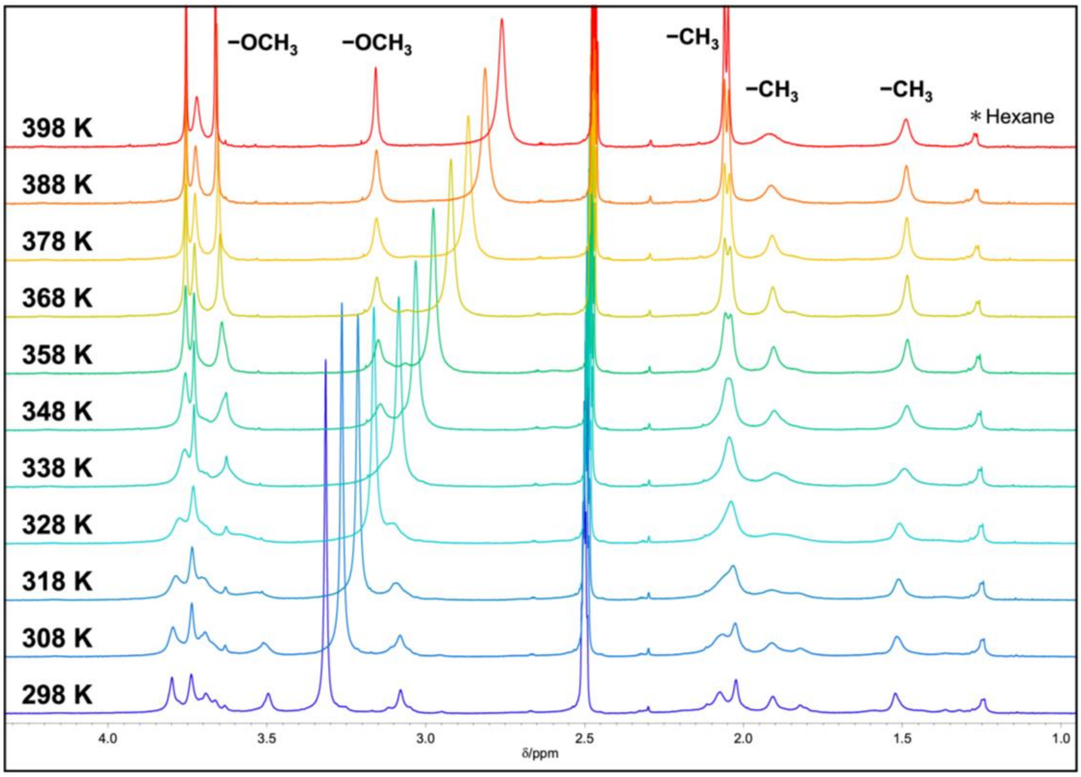

Figure S21 | VT-NMR charts. Changes in ^{1}H NMR spectra [(a) whole region and (b) aliphatic region] of **1c-2H** in DMSO-d_6 (298–398 K).

X-ray Analysis (*Figures S22-S25 and Tables S1-S12*)

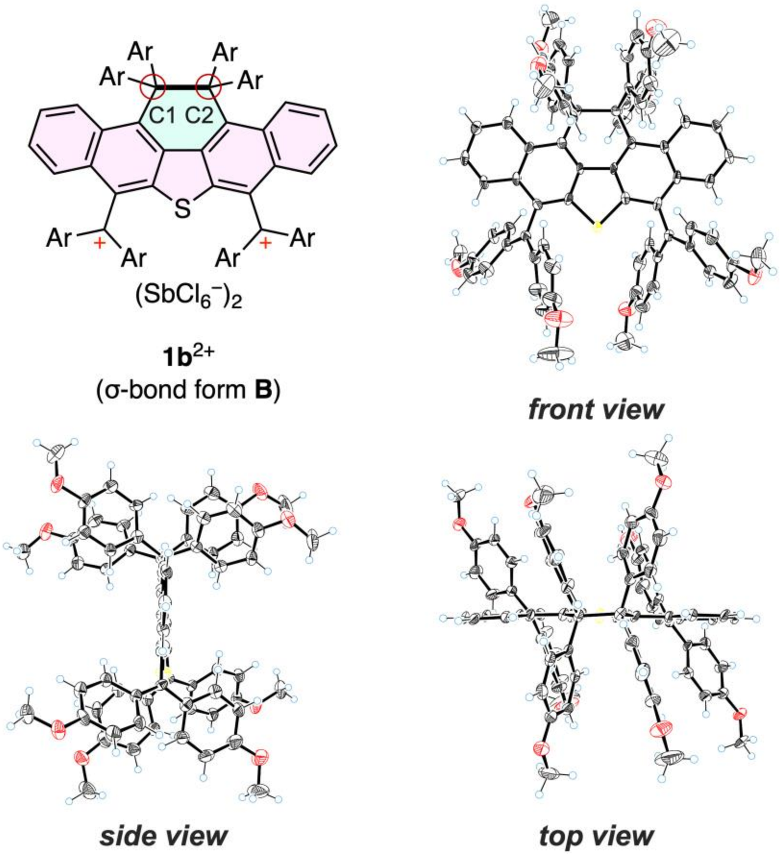

Figure S22 | Single-crystal X-ray diffraction analysis. X-ray crystal structures (ORTEP drawings) of **1b**$^{2+}$($SbCl_6^-$)$_2$ determined at 100 K (mol-1: one of the two crystallographically independent molecules). The counterions, solvent molecules, and disordered atoms are omitted for clarity. Thermal ellipsoids are shown at 50% probability.

Preparation of single crystals of dication salt 1c-2H^{2+}($SbCl_6^{-}$)$_2$

To a solution of **1c-2H** (3.28 mg, 2.52 μmol) in dry CH_2Cl_2 (2 mL) was added tris(4-bromophenyl)aminium hexachloroantimonate (4.08 mg, 5.00 μmol) at 25 °C to generate a deep blue solution, and the mixture was stirred at 25 °C for 40 min. The addition of dry hexane led to precipitation of the dication salt. The solvent was decantated and the resulting precipitates were washed with dry hexane five times, and dried in vacuo to give **1c-2H**$^{2+}$($SbCl_6^{-}$)$_2$ (5.0 mg) as a dark purple powder quantitatively. Single crystals of **1c-2H**$^{2+}$($SbCl_6^{-}$)$_2$ suitable for X-ray analysis were obtained as a dark green plate by recrystallization from CH_2Cl_2/^{t}BuOMe.

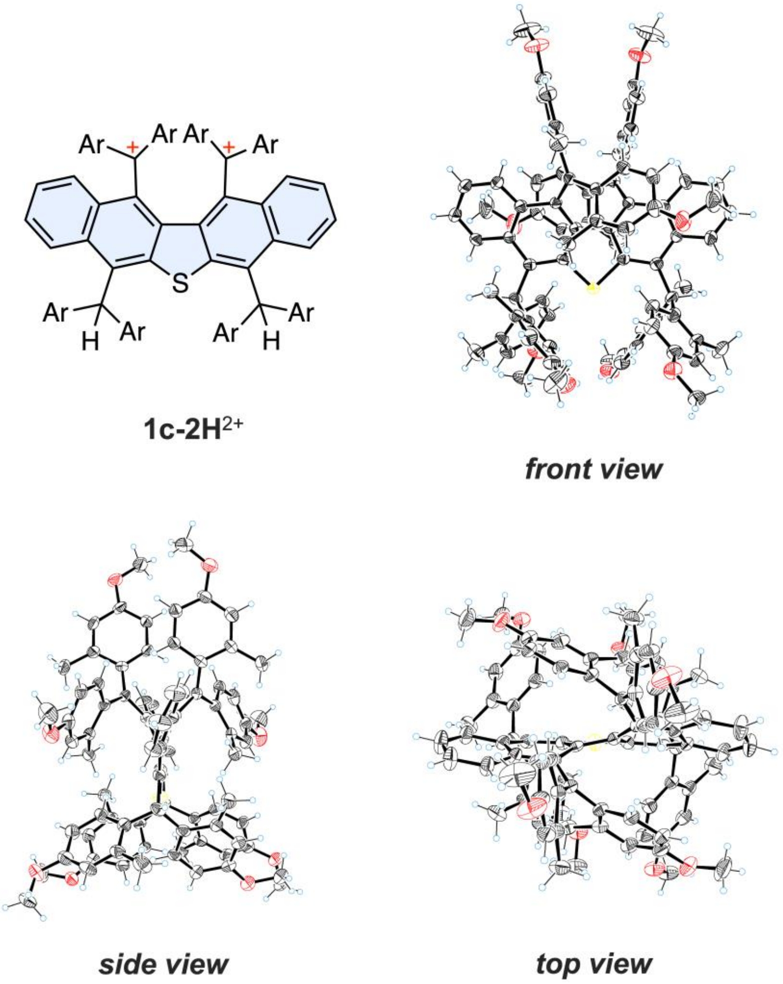

Figure S23 | Single-crystal X-ray diffraction analysis. X-ray crystal structures (ORTEP drawings) of **1c-2H**$^{2+}$($SbCl_6^{-}$)$_2$ determined at 150 K (mol-1: one of the two crystallographically independent molecules). The counterions are omitted for clarity. Thermal ellipsoids are shown at 30% probability.

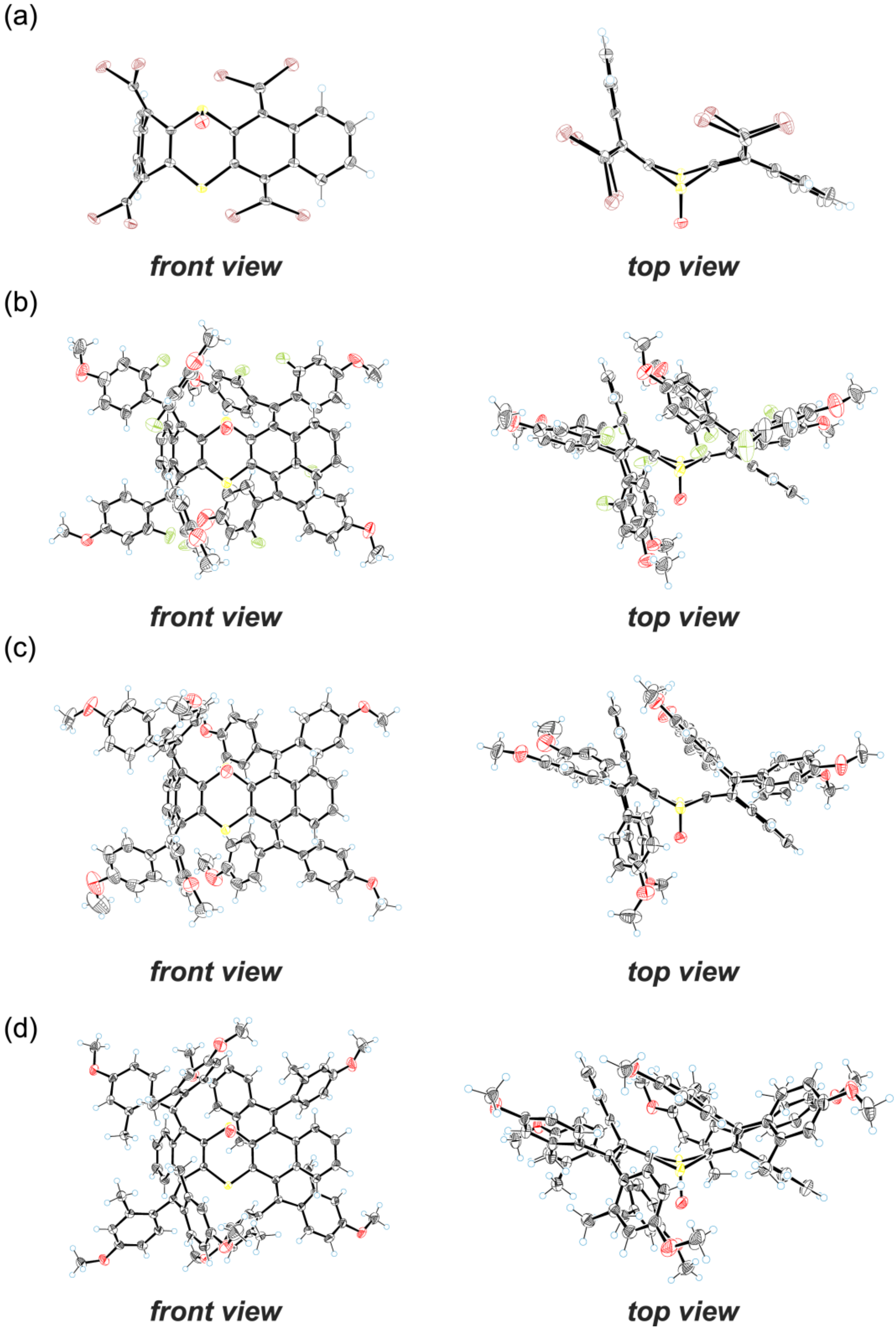

Figure S24 | Single-crystal X-ray diffraction analysis. X-ray crystal structures (ORTEP drawings) of (a) **3**, (b) **4a**, (c) **4b**, and (d) **4c** determined at 150 K. The solvent molecules and disordered atoms are omitted for clarity. Thermal ellipsoids are shown at 50% probability.

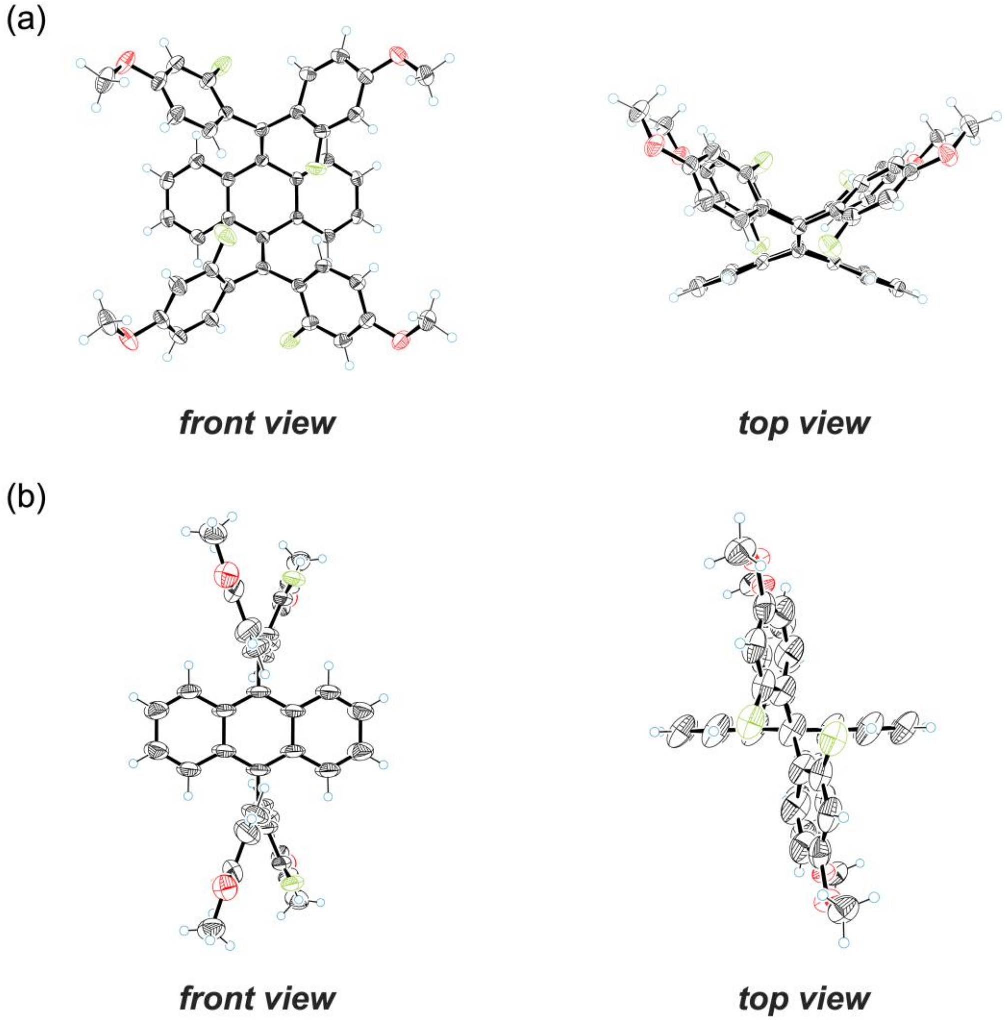

Figure S25 | Single-crystal X-ray diffraction analysis. X-ray crystal structures (ORTEP drawings) of (a) **I-a** and (b) **I-a**$^{2+}$($SbCl_6^-$)$_2$ determined at 150 K. The solvent molecules, disordered atoms, and counterions are omitted for clarity. Thermal ellipsoids are shown at 50% probability.

Table S1 | Summary of bond lengths and dihedral angles. Structural data of **1a** determined by X-ray analysis at 150 K and predicted by DFT calculations (CAM-B3LYP-D3/6-31G*).

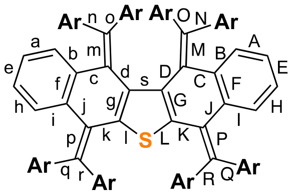

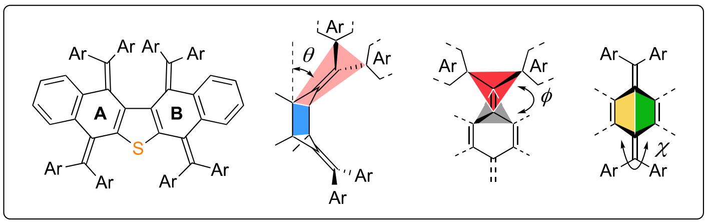

**1a** (Ar = 2-F-4-MeOC$_6$H$_3$)					
bond length (Å)			dihedral angle (°)		
	Expt.	*Calcd.*		Expt.	*Calcd.*
**a**	1.380(2)	*1.388*	θ_A	41.17(9)	*41.048*
**b**	1.393(2)	*1.394*		33.28(9)	*33.076*
**c**	1.491(2)	*1.490*	ϕ_A	11.56(7)	*8.038*
**d**	1.477(2)	*1.475*		9.51(7)	*9.209*
**e**	1.385(3)	*1.390*	χ_A	41.13(11)	*40.790*
**f**	1.409(2)	*1.407*			
**g**	1.3758(19)	*1.375*	θ_B	$=\theta_A$	*41.049*
**h**	1.381(2)	*1.387*			*33.078*
**i**	1.397(3)	*1.394*	ϕ_B	$=\phi_A$	*8.041*
**j**	1.495(2)	*1.489*			*9.208*
**k**	1.470(2)	*1.468*	χ_B	$=\chi_A$	*40.787*
**l**	1.7219(15)	*1.721*			
**m**	1.348(2)	*1.348*			
**n**	1.502(2)	*1.491*			
**o**	1.495(2)	*1.486*			
**p**	1.354(2)	*1.351*			
**q**	1.487(2)	*1.483*			
**r**	1.493(2)	*1.491*			
**s**	1.429(3)	*1.430*			
**A**	=a	*1.388*			
**B**	=b	*1.394*			
**C**	=c	*1.490*			
**D**	=d	*1.475*			
**E**	=e	*1.390*			
**F**	=f	*1.407*			
**G**	=g	*1.375*			
**H**	=h	*1.387*			
**I**	=i	*1.394*			
**J**	=j	*1.489*			
**K**	=k	*1.468*			
**L**	=l	*1.721*			
**M**	=m	*1.347*			
**N**	=n	*1.491*			
**O**	=o	*1.486*			
**P**	=p	*1.351*			
**Q**	=q	*1.483*			
**R**	=r	*1.491*			

Table S2 | Summary of bond lengths and dihedral angles. Structural data of **1b** determined by X-ray analysis at 150 K and predicted by DFT calculations (CAM-B3LYP-D3/6-31G*).

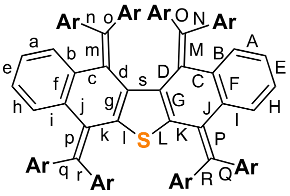

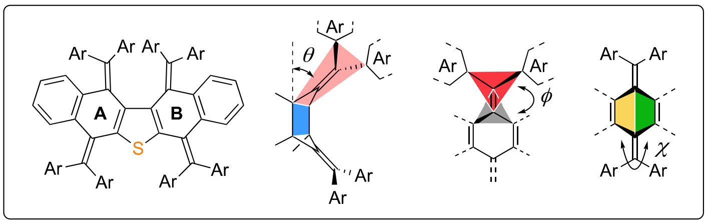

1b (Ar = 4-MeOC_6H_3)					
	bond length (Å)		dihedral angle (°)		
	Expt.	*Calcd.*		Expt.	*Calcd.*
**a**	1.387(3)	*1.387*	θ_A	44.08(10)	*38.517*
**b**	1.393(3)	*1.395*		33.91(10)	*28.328*
**c**	1.493(3)	*1.488*	ϕ_A	11.52(8)	*8.354*
**d**	1.477(2)	*1.478*		11.87(9)	*16.513*
**e**	1.387(3)	*1.391*	χ_A	42.78(12)	*38.938*
**f**	1.414(3)	*1.407*			
**g**	1.377(3)	*1.374*	θ_B	36.39(10)	*39.149*
**h**	1.381(3)	*1.386*		33.12(11)	*28.528*
**i**	1.397(3)	*1.397*	ϕ_B	12.44(8)	*8.178*
**j**	1.492(3)	*1.493*		7.34(10)	*17.064*
**k**	1.466(3)	*1.471*	χ_B	41.22(13)	*39.169*
**l**	1.7321(19)	*1.740*			
**m**	1.356(3)	*1.352*			
**n**	1.500(3)	*1.495*			
**o**	1.491(3)	*1.489*			
**p**	1.356(3)	*1.357*			
**q**	1.489(3)	*1.484*			
**r**	1.547(7)	*1.489*			
**s**	1.425(2)	*1.432*			
**A**	1.385(3)	*1.387*			
**B**	1.394(3)	*1.395*			
**C**	1.492(3)	*1.488*			
**D**	1.480(2)	*1.479*			
**E**	1.383(3)	*1.390*			
**F**	1.413(3)	*1.407*			
**G**	1.381(2)	*1.374*			
**H**	1.387(3)	*1.385*			
**I**	1.392(3)	*1.397*			
**J**	1.495(3)	*1.493*			
**K**	1.469(3)	*1.471*			
**L**	1.7243(19)	*1.738*			
**M**	1.357(3)	*1.353*			
**N**	1.499(3)	*1.495*			
**O**	1.495(3)	*1.488*			
**P**	1.356(3)	*1.357*			
**Q**	1.487(3)	*1.485*			
**R**	1.490(3)	*1.490*			

Table S3 | Summary of bond lengths and dihedral angles. Structural data of **1c** determined by X-ray analysis at 100 K and predicted by DFT calculations (CAM-B3LYP-D3/6-31G*).

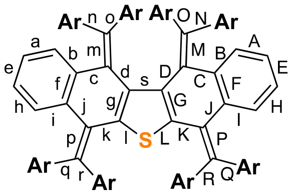

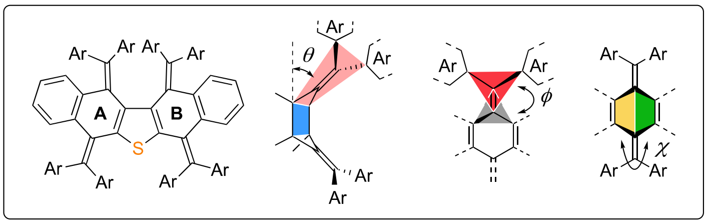

**1c** (Ar = 4-MeO-2-MeC_6H_3)					
bond length (Å)			dihedral angle (°)		
	Expt.	*Calcd.*		Expt.	*Calcd.*
**a**	1.393(6)	*1.387*	θ_A	39.9(2)	*38.552*
**b**	1.394(5)	*1.396*		26.7(2)	*26.132*
**c**	1.501(5)	*1.494*	ϕ_A	2.8(2)	*8.361*
**d**	1.484(5)	*1.482*		12.41(4)	*9.103*
**e**	1.377(6)	*1.388*	χ_A	38.6(3)	*37.493*
**f**	1.406(5)	*1.412*			
**g**	1.374(5)	*1.377*	θ_B	$=\theta_A$	*38.555*
**h**	1.373(6)	*1.386*			*26.140*
**i**	1.401(5)	*1.396*	ϕ_B	$=\phi_A$	*8.355*
**j**	1.485(5)	*1.490*			*9.096*
**k**	1.479(5)	*1.470*	χ_B	$=\chi_A$	*37.499*
**l**	1.723(4)	*1.735*			
**m**	1.348(5)	*1.357*			
**n**	1.514(6)	*1.499*			
**o**	1.542(7)	*1.499*			
**p**	1.349(5)	*1.356*			
**q**	1.494(7)	*1.494*			
**r**	1.511(5)	*1.494*			
**s**	1.433(7)	*1.435*			
**A**	=a	*1.387*			
**B**	=b	*1.396*			
**C**	=c	*1.494*			
**D**	=d	*1.482*			
**E**	=e	*1.388*			
**F**	=f	*1.412*			
**G**	=g	*1.377*			
**H**	=h	*1.385*			
**I**	=i	*1.397*			
**J**	=j	*1.490*			
**K**	=k	*1.470*			
**L**	=l	*1.735*			
**M**	=m	*1.356*			
**N**	=n	*1.499*			
**O**	=o	*1.499*			
**P**	=p	*1.356*			
**Q**	=q	*1.494*			
**R**	=r	*1.494*			

Table S4 | Summary of bond lengths and dihedral angles. Structural data of **1a**$^{2+}$($SbCl_6^-$)$_2$ determined by X-ray analysis at 100 K and predicted by DFT calculations (CAM-B3LYP-D3/6-31G*).

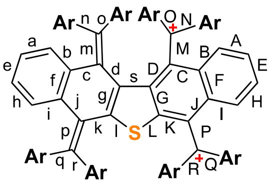

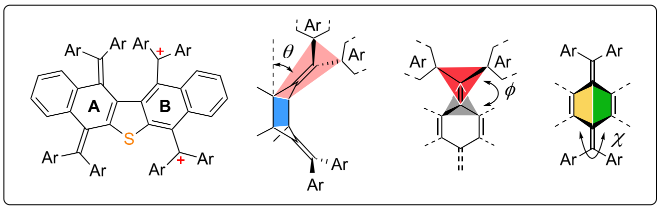

**1a**$^{2+}$($SbCl_6^-$)$_2$ (Ar = 2-F-4-MeOC$_6$H$_3$)					
bond length (Å)			dihedral angle (°)		
	Expt.	*Calcd.*		Expt.	*Calcd.*
**a**	1.393(5)	*1.391*	θ_A	51.44(19)	*49.700*
**b**	1.396(5)	*1.391*		29.5(2)	*32.128*
**c**	1.489(5)	*1.487*	ϕ_A	11.47(14)	*20.226*
**d**	1.499(4)	*1.488*		16.73(16)	*14.744*
**e**	1.386(5)	*1.390*	χ_A	42.3(2)	*40.839*
**f**	1.407(5)	*1.411*			
**g**	1.372(4)	*1.365*	θ_B	5.4(2)	*9.870*
**h**	1.383(5)	*1.389*		3.55(19)	*2.902*
**i**	1.397(5)	*1.393*	ϕ_B	48.19(15)	*57.132*
**j**	1.487(5)	*1.493*		72.65(16)	*62.301*
**k**	1.470(4)	*1.463*	χ_B	1.03(15)	*3.010*
**l**	1.740(4)	*1.754*			
**m**	1.345(5)	*1.356*			
**n**	1.500(4)	*1.483*			
**o**	1.492(5)	*1.486*			
**p**	1.363(5)	*1.357*			
**q**	1.482(4)	*1.475*			
**r**	1.486(5)	*1.489*			
**s**	1.449(4)	*1.449*			
**A**	1.370(5)	*1.367*			
**B**	1.418(5)	*1.422*			
**C**	1.448(4)	*1.436*			
**D**	1.416(4)	*1.398*			
**E**	1.404(6)	*1.410*			
**F**	1.433(5)	*1.428*			
**G**	1.430(4)	*1.424*			
**H**	1.360(5)	*1.366*			
**I**	1.423(5)	*1.420*			
**J**	1.418(5)	*1.425*			
**K**	1.375(4)	*1.377*			
**L**	1.736(3)	*1.749*			
**M**	1.465(4)	*1.478*			
**N**	1.439(5)	*1.441*			
**O**	1.428(5)	*1.414*			
**P**	1.500(4)	*1.481*			
**Q**	1.395(5)	*1.419*			
**R**	1.438(5)	*1.422*			

Table S5 | Summary of bond lengths and dihedral angles. Structural data of **1b**$^{2+}$($SbCl_6^-$)$_2$ determined by X-ray analysis at 100 K and predicted by DFT calculations (CAM-B3LYP-D3/6-31G*).

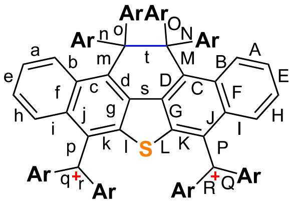

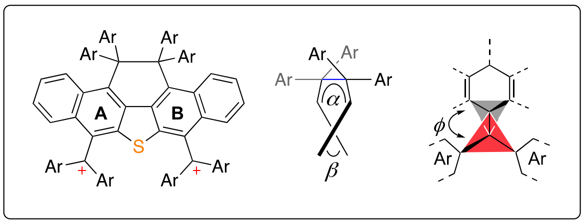

**1b**$^{2+}$($SbCl_6^-$)$_2$ (Ar = 4-MeOC$_6$H$_4$)							
bond length (Å)				dihedral angle (°)			
	Expt. (mol1)	Expt. (mol2)	*Calcd.*		Expt. (mol1)	Expt. (mol2)	*Calcd.*
**a**	1.361(11)	1.373(11)	*1.368*	α	10.6(9)	13.9(9)	*27.518*
**b**	1.44(1)	1.432(10)	*1.422*				
**c**	1.433(10)	1.434(10)	*1.443*	β	2.5(2)	3.7(9)	*1.533*
**d**	1.368(10)	1.368(10)	*1.379*				
**e**	1.398(12)	1.395(12)	*1.405*	ϕ_A	55.9(4)	43.86(13)	*53.170*
**f**	1.461(11)	1.449(10)	*1.429*	ϕ_B	57.9(3)	43.8(4)	*53.169*
**g**	1.415(10)	1.418(10)	*1.413*				
**h**	1.350(12)	1.367(11)	*1.368*				
**i**	1.424(10)	1.429(9)	*1.417*				
**j**	1.435(11)	1.432(10)	*1.442*				
**k**	1.387(11)	1.399(10)	*1.386*				
**l**	1.745(7)	1.748(7)	*1.764*				
**m**	1.558(10)	1.553(10)	*1.544*				
**n**	1.552(10)	1.573(9)	*1.560*				
**o**	1.541(10)	1.544(9)	*1.539*				
**p**	1.478(10)	1.475(10)	*1.459*				
**q**	1.431(11)	1.437(11)	*1.428*				
**r**	1.421(11)	1.427(11)	*1.434*				
**s**	1.455(9)	1.455(9)	*1.453*				
**t**	1.731(11)	1.708(10)	*1.680*				
**A**	1.384(11)	1.386(10)	*1.368*				
**B**	1.438(10)	1.432(10)	*1.422*				
**C**	1.441(10)	1.446(9)	*1.443*				
**D**	1.369(10)	1.372(10)	*1.379*				
**E**	1.397(12)	1.392(11)	*1.405*				
**F**	1.438(11)	1.428(10)	*1.429*				
**G**	1.39(1)	1.411(10)	*1.413*				
**H**	1.370(11)	1.358(11)	*1.368*				
**I**	1.423(10)	1.426(9)	*1.417*				
**J**	1.431(11)	1.447(10)	*1.442*				
**K**	1.42(1)	1.416(10)	*1.386*				
**L**	1.747(8)	1.746(7)	*1.764*				
**M**	1.547(10)	1.539(9)	*1.544*				
**N**	1.556(10)	1.546(9)	*1.540*				
**O**	1.528(11)	1.571(10)	*1.560*				
**P**	1.461(10)	1.452(11)	*1.459*				
**Q**	1.424(10)	1.439(11)	*1.434*				
**R**	1.436(11)	1.444(12)	*1.428*				

Table S6 | Summary of bond lengths and dihedral angles. Structural data of **1b-2H** determined by X-ray analysis at 200 K and predicted by DFT calculations (CAM-B3LYP-D3/6-31G*).

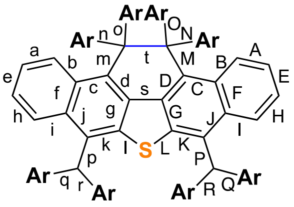

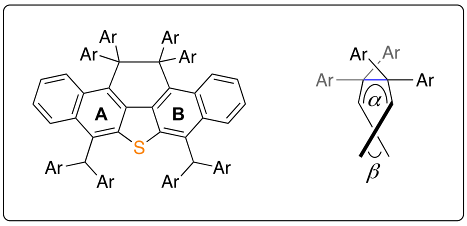

**1b-2H** (Ar = 4-MeOC$_6$H$_4$)					
bond length (Å)			dihedral angle (°)		
	Expt.	*Calcd.*		Expt.	*Calcd.*
**a**	1.3698(18)	*1.368*	***α***	27.74(18)	*29.749*
**b**	1.4176(18)	*1.422*			
**c**	1.4449(16)	*1.440*	***β***	6.45(5)	*8.513*
**d**	1.3709(17)	*1.369*			
**e**	1.401(2)	*1.406*			
**f**	1.4413(18)	*1.435*			
**g**	1.4234(17)	*1.422*			
**h**	1.356(2)	*1.367*			
**i**	1.4297(17)	*1.422*			
**j**	1.4363(18)	*1.433*			
**k**	1.3766(17)	*1.371*			
**l**	1.7609(13)	*1.763*			
**m**	1.5555(17)	*1.549*			
**n**	1.5408(17)	*1.541*			
**o**	1.5594(16)	*1.559*			
**p**	1.5315(17)	*1.530*			
**q**	1.5279(18)	*1.530*			
**r**	1.5193(17)	*1.521*			
**s**	1.452(2)	*1.453*			
**t**	1.709(2)	*1.689*			
**A**	=a	*1.368*			
**B**	=b	*1.422*			
**C**	=c	*1.440*			
**D**	=d	*1.369*			
**E**	=e	*1.406*			
**F**	=f	*1.435*			
**G**	=g	*1.422*			
**H**	=h	*1.367*			
**I**	=i	*1.422*			
**J**	=j	*1.433*			
**K**	=k	*1.371*			
**L**	=l	*1.763*			
**M**	=m	*1.549*			
**N**	=n	*1.559*			
**O**	=o	*1.541*			
**P**	=p	*1.530*			
**Q**	=q	*1.521*			
**R**	=r	*1.530*			

Table S7 | Summary of bond lengths and dihedral angles. Structural data of **1c-2H** determined by X-ray analysis at 150 K and predicted by DFT calculations (CAM-B3LYP-D3/6-31G*).

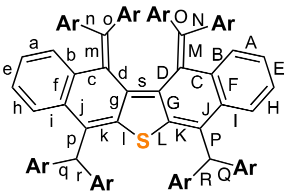

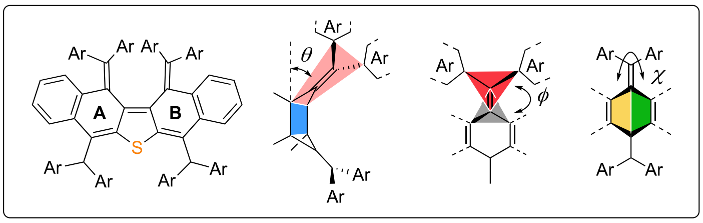

**1c-2H** (Ar = 4-MeO-2-MeC_6H_3)					
bond length (Å)			dihedral angle (°)		
	Expt.	*Calcd.*		Expt.	*Calcd.*
**a**	1.391(2)	*1.388*	θ_A	37.32(7)	*36.721*
**b**	1.3960(18)	*1.395*			
**c**	1.4912(17)	*1.491*	ϕ_A	10.77(6)	*10.354*
**d**	1.4740(17)	*1.472*			
**e**	1.385(2)	*1.387*	χ_A	25.68(10)	*25.361*
**f**	1.4184(18)	*1.419*			
**g**	1.4497(17)	*1.454*	θ_B	37.46(8)	*36.552*
**h**	1.383(2)	*1.386*			
**i**	1.4116(18)	*1.402*	ϕ_B	7.86(6)	*9.533*
**j**	1.4709(17)	*1.472*			
**k**	1.3623(18)	*1.355*	χ_B	24.49(10)	*24.928*
**l**	1.7645(12)	*1.764*			
**m**	1.3608(18)	*1.359*			
**n**	1.5061(17)	*1.496*			
**o**	1.5030(18)	*1.496*			
**p**	1.5269(17)	*1.528*			
**q**	1.5292(18)	*1.533*			
**r**	1.5229(19)	*1.528*			
**s**	1.3697(19)	*1.363*			
**A**	1.388(2)	*1.388*			
**B**	1.3999(19)	*1.395*			
**C**	1.4918(18)	*1.491*			
**D**	1.4718(17)	*1.472*			
**E**	1.384(2)	*1.387*			
**F**	1.4229(19)	*1.419*			
**G**	1.4490(17)	*1.454*			
**H**	1.384(2)	*1.386*			
**I**	1.4073(19)	*1.402*			
**J**	1.4693(19)	*1.472*			
**K**	1.3659(18)	*1.354*			
**L**	1.7611(13)	*1.764*			
**M**	1.3585(18)	*1.359*			
**N**	1.5091(18)	*1.496*			
**O**	1.5030(19)	*1.496*			
**P**	1.5305(17)	*1.528*			
**Q**	1.528(2)	*1.533*			
**R**	1.527(2)	*1.527*			

Table S8 | Summary of bond lengths and dihedral angles. Structural data of **1c-2H**$^{\bullet+}$ and **1c-2H**$^{2+}$ obtained by DFT calculations ((U)CAM-B3LYP-D3/6-31G*) and/or by X-ray analysis at 150 K.

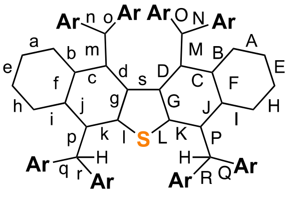

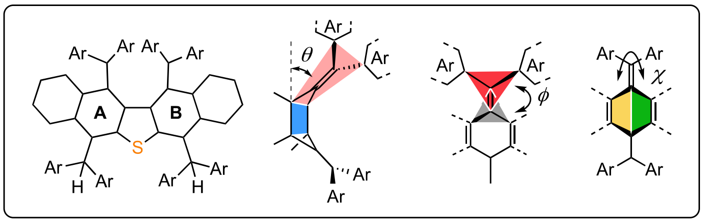

**1c-2H**$^{\bullet+}$ (folded form)				**1c-2H**$^{2+}$ (twisted form)							
bond length (Å)		dihedral angle (°)		bond length (Å)				dihedral angle (°)			
	Calcd.		*Calcd.*		Expt. (mol1)	Expt. (mol2)	*Calcd.*		Expt. (mol1)	Expt. (mol2)	*Calcd.*
**a**	*1.384*	θ_A	*34.252*	**a**	1.365(14)	1.364(14)	*1.369*	θ_A	23.6(5)	23.6(5)	*20.798*
**b**	*1.398*			**b**	1.439(13)	1.412(13)	*1.421*				
**c**	*1.481*	ϕ_A	*13.193*	**c**	1.466(12)	1.464(12)	*1.447*	ϕ_A	37.2(4)	37.2(4)	*44.020*
**d**	*1.461*			**d**	1.399(11)	1.396(11)	*1.407*				
**e**	*1.394*	χ_A	*23.300*	**e**	1.398(16)	1.388(16)	*1.408*	χ_A	14.5(7)	14.2(7)	*13.404*
**f**	*1.426*			**f**	1.430(12)	1.425(12)	*1.437*				
**g**	*1.424*	θ_B	*34.231*	**g**	1.403(11)	1.416(11)	*1.419*	θ_B	22.9(5)	24.4(6)	*25.354*
**h**	*1.378*			**h**	1.359(15)	1.349(15)	*1.367*				
**i**	*1.410*	ϕ_B	*12.563*	**i**	1.421(12)	1.436(12)	*1.421*	ϕ_B	36.3(4)	35.8(4)	*35.449*
**j**	*1.450*			**j**	1.438(12)	1.411(12)	*1.432*				
**k**	*1.382*	χ_B	*23.098*	**k**	1.390(12)	1.400(12)	*1.381*	χ_B	12.5(7)	14.2(7)	*15.209*
**l**	*1.750*			**l**	1.743(9)	1.753(9)	*1.761*				
**m**	*1.372*			**m**	1.463(11)	1.458(11)	*1.470*				
**n**	*1.491*			**n**	1.423(12)	1.425(12)	*1.435*				
**o**	*1.487*			**o**	1.456(11)	1.452(11)	*1.444*				
**p**	*1.528*			**p**	1.541(12)	1.539(12)	*1.534*				
**q**	*1.532*			**q**	1.539(14)	1.494(14)	*1.539*				
**r**	*1.528*			**r**	1.542(14)	1.555(14)	*1.526*				
**s**	*1.397*			**s**	1.451(11)	1.456(11)	*1.454*				
**A**	*1.385*			**A**	1.354(14)	1.376(14)	*1.373*				
**B**	*1.398*			**B**	1.397(13)	1.395(13)	*1.414*				
**C**	*1.481*			**C**	1.457(11)	1.458(11)	*1.457*				
**D**	*1.461*			**D**	1.412(11)	1.405(11)	*1.418*				
**E**	*1.394*			**E**	1.410(16)	1.391(16)	*1.405*				
**F**	*1.426*			**F**	1.460(11)	1.455(11)	*1.436*				
**G**	*1.423*			**G**	1.403(11)	1.408(11)	*1.411*				
**H**	*1.378*			**H**	1.373(14)	1.347(14)	*1.369*				
**I**	*1.410*			**I**	1.417(12)	1.421(12)	*1.418*				
**J**	*1.450*			**J**	1.418(11)	1.424(11)	*1.435*				
**K**	*1.382*			**K**	1.391(11)	1.415(11)	*1.381*				
**L**	*1.751*			**L**	1.734(8)	1.738(8)	*1.763*				
**M**	*1.371*			**M**	1.452(11)	1.452(11)	*1.444*				
**N**	*1.491*			**N**	1.422(12)	1.422(12)	*1.447*				
**O**	*1.488*			**O**	1.465(11)	1.465(11)	*1.453*				
**P**	*1.526*			**P**	1.538(11)	1.513(12)	*1.531*				
**Q**	*1.532*			**Q**	1.536(12)	1.550(13)	*1.531*				
**R**	*1.527*			**R**	1.536(13)	1.530(15)	*1.526*				

Table S9 | Summary of the results of X-ray analysis. Crystal data of **1a**, **1b**, **1c**, and **1a**$^{2+}$($SbCl_6^-$)$_2$.

**Compounds**	**1a**	**1b**	**1c**	**1a**$^{2+}$($SbCl_6^-$)$_2$
Solvent system	DMSO/MeOH	$CHCl_3$/hexane	DMSO/H_2O	MeCN/tBuOMe
Empirical formula	$C_{80}H_{56}F_8O_8S$	$C_{80}H_{64}O_8S$	$C_{88}H_{80}O_2S$	$C_{80}H_{56}Cl_{12}F_8O_8SSb_2$ • $1.5CH_3CN$ • $(CH_3)_3COCH_3$
Formula weight	1329.30	1158.37	1297.58	2147.93
Temperature/K	150	150	100	100
Crystal system	monoclinic	monoclinic	monoclinic	triclinic
Space group	*C*2/*c*	*P*2$_1$/*n*	*P*2/*c*	*P*-1
a/Å	14.64488(14)	14.3661(2)	10.8291(2)	14.42709(12)
b/Å	21.00585(18)	20.9256(4)	14.3092(7)	17.98609(14)
c/Å	21.2786(2)	21.2833(3)	22.1848(8)	18.76318(14)
α/°	90	90	90	92.2148(6)
β/°	105.6985(10)	104.2114(16)	96.833(3)	99.3480(7)
γ/°	90	90	90	110.8385(7)
Volume/Å^3	6301.72(11)	6202.37(19)	3413.2(2)	4465.27(6)
Z	4	4	2	2
ρ_{calc} g/cm^3	1.401	1.269	1.263	1.598
μ/mm^{-1}	1.182	0.944	0.900	8.949
Color and shape	light yellow block	yellow block	yellow block	dark violet block
Crystal size/mm^3	0.095 × 0.069 × 0.039	0.25 × 0.20 × 0.20	0.042 × 0.036 × 0.031	0.14 × 0.095 × 0.043
Reflections collected	31132	20157	23896	84987
R_{int}	R_{int} = 0.0272	R_{int} = 0.0481	R_{int} = 0.0615	R_{int} = 0.0354
Data/restraints/ parameters	6501/4/483	20157/0/918	6701/478/636	18203/148/1168
GOF	1.041	1.024	1.083	1.024
R_1 [I>=2σ(I)]	0.0427	0.0492	0.0836	0.0511
wR_2 [I>=2σ(I)]	0.1109	0.1338	0.1848	0.1327
R_1 [all data]	0.0502	0.0536	0.1589	0.0563
wR_2 [all data]	0.1159	0.1371	0.2215	0.1326
Largest diff.peak /hole/e Å^{-3}	0.23/-0.21	0.29/-0.29	0.34/-0.29	1.60/-1.27
Solvent mask	None	None	None	Used for the analysis
CCDC No.	2342072	2342073	2342074	2342075

Table S10 | Summary of the results of X-ray analysis. Crystal data of **1b**$^{2+}$($SbCl_6^-$)$_2$, **1b-2H**, **1c-2H**, and **1c-2H**$^{2+}$($SbCl_6^-$)$_2$.

**Compounds**	**1b**$^{2+}$($SbCl_6^-$)$_2$ [a]	**1b-2H**	**1c-2H**	**1c-2H**$^{2+}$($SbCl_6^-$)$_2$
Solvent system	MeCN/tBuOMe	1,4-dioxane/hexane	benzene/hexane	MeCN/^{t}BuOMe
Empirical formula	$C_{80}H_{64}Cl_{12}O_8SSb_2$ • 0.5 CH_3CN	$C_{80}H_{66}O_8S$ • $2C_4H_8O_2$	$C_{88}H_{82}O_8S$ • C_6H_{14}	$C_{88}H_{82}Cl_{12}O_8SSb_2$
Formula weight	1874.80	1363.59	1385.76	1968.49
Temperature/K	100	200	150	150
Crystal system	monoclinic	monoclinic	triclinic	triclinic
Space group	$P2_1/n$	$I2/a$	P-1	P-1
a/Å	21.4934(3)	18.2089(4)	13.61478(14)	17.0521(2)
b/Å	31.8232(4)	16.6193(4)	16.69552(16)	18.1682(3)
c/Å	23.3850(3)	23.3793(6)	18.01854(19)	29.3025(5)
α/°	90	90	102.4239(9)	103.4350(10)
β/°	97.9214(12)	98.673(2)	105.4513(9)	97.3400(10)
γ/°	90	90	98.2256(8)	90.0380(10)
Volume/Å^3	15842.4(3)	6994.1(3)	3766.87(7)	8753.0(2)
Z	8	4	2	4
ρ_{calc} g/cm^3	1.572	1.295	1.222	1.494
μ/mm^{-1}	9.832	0.948	0.845	8.923
Color and shape	dark red plate	colourless needle	dark red block	dark green plate
Crystal size/mm^3	0.15 × 0.15 × 0.02	0.254 × 0.035 × 0.024	0.251 × 0.193 × 0.071	0.468 × 0.111 × 0.018
Reflections collected	33585	31991	56374	113966
R_{int}	R_{int} = 0.0948	R_{int} = 0.0380	R_{int} = 0.0329	R_{int} = 0.1248
Data/restraints/ parameters	33585/0/1900	7132/0/406	15305/0/891	35085/28/2068
GOF	1.658	1.061	1.050	1.023
R_1 [I>=2σ(I)]	0.1283	0.0405	0.0445	0.1125
wR_2 [I>=2σ(I)]	0.3668	0.1092	0.1242	0.2896
R_1 [all data]	0.1419	0.0471	0.0492	0.1772
wR_2 [all data]	0.3819	0.1129	0.1281	0.3403
Largest diff.peak /hole/e Å^{-3}	7.96/-5.14	0.23/-0.32	0.71/-0.23	2.54/-1.47
Solvent mask	Used for the analysis	Used for the analysis	Used for the analysis	None
CCDC No.	2342076	2342077	2342079	2400963

[a] Some B-level alerts were found in the checkCIF file.

Authors response: These alerts are a consequence of two crystallographically independent molecules and disordered solvent molecules as well as a crystal twinning for **1b**$^{2+}$($SbCl_6^-$)$_2$.

Table S11 | Summary of the results of X-ray analysis. Crystal data of **3**, **4a**, **4b**, and **4c**.

**Compounds**	**3** [a]	**4a** [b]	**4b** [b]	**4c**
Solvent system	$CHCl_3$/hexane	1,2-dichloroethane/hexane	EtOAc/MeOH	EtOAc/MeOH
Empirical formula	$C_{24}H_8Br_8OS_2 \cdot CHCl_3$	$C_{80}H_{56}O_9S_2 \cdot C_2H_4Cl_2$	$C_{80}H_{64}O_9S_2$	$C_{88}H_{80}O_9S_2$ $\cdot C_4H_{12}O_2 \cdot CH_3OH$
Formula weight	1135.07	1476.32	1233.43	1465.78
Temperature/K	150	150	150	150
Crystal system	triclinic	triclinic	triclinic	triclinic
Space group	*P*-1	*P*-1	*P*-1	*P*-1
a/Å	10.25295(15)	12.05383(11)	16.2315(5)	13.8566(2)
b/Å	12.6453(3)	16.27991(18)	16.2849(4)	14.7777(2)
c/Å	13.7864(3)	18.2257(2)	17.2420(5)	21.0135(3)
α/°	63.345(2)	98.6754(10)	67.194(3)	101.0943(13)
β/°	81.7502(14)	96.7394(9)	62.533(3)	96.1875(12)
γ/°	85.6332(14)	96.7056(8)	70.350(3)	111.6646(14)
Volume/Å^3	1580.80(6)	3478.08(7)	3657.9(2)	3848.03(11)
Z	2	2	2	2
ρ_{calc} g/cm^3	2.385	1.410	1.120	1.265
μ/mm^{-1}	15.893	2.102	1.089	1.144
Color and shape	colorless plate	colorless plate	colorless block	yellow plate
Crystal size/mm^3	0.30 × 0.20 × 0.04	0.174 × 0.132 × 0.039	0.15 × 0.15 × 0.05	0.155 × 0.13 × 0.033
Reflections collected	23515	66890	51879	67235
R_{int}	R_{int} = 0.0568	R_{int} = 0.0346	R_{int} = 0.0484	R_{int} = 0.0331
Data/restraints/ parameters	6449/0/352	14256/9/990	14752/0/839	15634/2/931
GOF	1.056	1.043	1.059	1.027
R_1 [I>=2σ(I)]	0.0469	0.0586	0.0436	0.0401
wR_2 [I>=2σ(I)]	0.1299	0.1552	0.1277	0.1037
R_1 [all data]	0.0494	0.0660	0.0504	0.0454
wR_2 [all data]	0.1325	0.1608	0.1327	0.1066
Largest diff.peak /hole/e Å^{-3}	2.45/-1.62	1.00/-0.45	0.48/-0.41	0.54/-0.37
Solvent mask	None	Used for the analysis	Used for the analysis	Used for the analysis
CCDC No.	2342080	2342081	2342082	2342083

[a] A-level alert was found in the checkCIF file.

Authors response: This alert is due to a halogen bonding between Br and O atoms for **3**.

[b] Some B-level alerts were found in the checkCIF file.

Authors response: These alerts are a consequence of disordered atoms for **4a** and **4b**.

Table S12 | Summary of the results of X-ray analysis. Crystal data of **I-a** and **I-a**$^{2+}$($SbCl_6^-$)$_2$.

**Compounds**	**I-a**	**I-a**$^{2+}$($SbCl_6^-$)$_2$ [a]
Solvent system	$CHCl_3$/hexane	MeCN/benzene
Empirical formula	$C_{44}H_{32}F_4O_4$ • $CHCl_3$	$C_{44}H_{32}Cl_{12}F_4O_4Sb_2$
Formula weight	820.06	1369.59
Temperature/K	150	150
Crystal system	orthorhombic	tetragonal
Space group	*Pnma*	*P*4/*m*
a/Å	32.1306(5)	12.84729(19)
b/Å	17.2854(3)	12.84729(19)
c/Å	8.31283(14)	17.7485(4)
α/°	90	90
β/°	90	90
γ/°	90	90
Volume/Å^3	4616.86(12)	2929.43(11)
Z	4	2
ρ_{calc} g/cm^3	1.180	1.553
μ/mm^{-1}	2.252	12.771
Color and shape	colorless block	dark red plate
Crystal size/mm^3	0.2 × 0.06 × 0.06	0.1 × 0.1 × 0.01
Reflections collected	15778	11957
R_{int}	R_{int} = 0.0301	R_{int} = 0.0277
Data/restraints/ parameters	4842/0/279	3121/0/180
GOF	1.073	1.145
R_1 [I>=2σ(I)]	0.0777	0.0638
wR_2 [I>=2σ(I)]	0.2247	0.1874
R_1 [all data]	0.0866	0.0715
wR_2 [all data]	0.2326	0.1941
Largest diff.peak /hole/e Å^{-3}	1.26/-1.19	2.28/-2.57
Solvent mask	Used for the analysis	Used for the analysis
CCDC No.	2342084	2342085

[a] Some B-level alerts were found in the checkCIF file.

Authors response: These alerts are a consequence of disordered atoms and counterions for **I-a**$^{2+}$($SbCl_6^-$)$_2$.

Theoretical Study

DFT calculations (Figures S26-S44 and Tables S13 and S14)

Molecular design using DFT calculations

Before starting the synthetic study, we conducted preliminary theoretical calculations of reference compounds. As shown in Table S13, DFT calculations have suggested that the conventional *p*-QD form **A** is the most stable dicationic structure for a QD derivative, consisting of two QD units linked just by one C-C covalent bond. Therefore, we planned a further refinement of this molecular design to realize structurally diverse QD-based systems. We decided that the co-planarization of the two QD units and their precise proximity are crucial for providing form **B** with an energy gain upon formation of the σ-bond. Furthermore, form **C**, an isomer of the diphenoquinoid structure that has not yet been isolated, could be obtained by proper molecular design.

Discussion for the conformational preferences of the cationic states of **1c-2H**

DFT calculations at the (U)CAM-B3LYP-D3/6-31G* level were conducted to obtain the optimized structures of radical cation **1c-2H**$^{\bullet+}$ and dication **1c-2H**$^{2+}$ (Figures S32 and S33, Table S13). A twisted form was calculated to be the most stable structure of dication **1c-2H**$^{2+}$, while a folded form is a metastable structure (E_{rel}: +15.9 kcal/mol). Indeed, a single-crystal X-ray diffraction analysis revealed that **1c-2H**$^{2+}$ adopts the twisted form, in which two diarylmethylium units are attached to the heteroacene core to avoid the steric repulsion of the *ortho* substituents (Figure S23, Table S8). On the other hand, for radical cation **1c-2H**$^{\bullet+}$, the relative stability of the two forms is reversed; a folded form was calculated to be by 23.1 kcal/mol more stable than a twisted form. In the case of previously reported arylated *p*-QD redox systems, the corresponding radical cations have been proven to be transient species and adopt only twisted conformations.[4] Despite the generation of a positive charge upon 1e oxidation, **1c-2H**$^{\bullet+}$ retains the folded structure as its most stable form. Such a unique structural preference can be explained by both the stabilization of the folded structure, in which the positive charge is effectively delocalized over the *o*-diphenoquinoid structure, and the destabilization of the twisted structure due to steric repulsion resulting from the bulky *ortho* substituents introduced onto the congested exomethylenes. The simulated bond lengths around the *o*-diphenoquinoid structure also suggest that the charge is effectively delocalized (Table S8).

Table S13 | Summary of the results of DFT calculations. HOMO and LUMO levels and relative energies for possible isomers of **1a**$^{2+}$, **1b**$^{2+}$, **1c**$^{2+}$, **1b-2H**, **1c-2H**, **1c-2H**$^{\bullet+}$, and **1c-2H**$^{2+}$ predicted by DFT calculations at the (U)CAM-B3LYP-D3/6-31G* level.

**1a**$^{2+}$ (Ar = 2-F-4-MeOC_6H_3)	*p*-quinoid form **A**	σ-bond form **B**	*o*-diphenoquinoid form **C**
LUMO (eV)	–7.01	–6.65	–7.15
HOMO (eV)	–9.90	–10.34	–10.07
E_{rel} (kcal mol^{-1})	0	+5.17	+16.06
**1b**$^{2+}$ (Ar = 4-MeOC_6H_4)	*p*-quinoid form **A**	σ-bond form **B**	*o*-diphenoquinoid form **C**
LUMO (eV)	–6.89	–6.60	–7.15
HOMO (eV)	–9.84	–10.16	–9.91
E_{rel} (kcal mol^{-1})	+1.94	0	+17.18
**1c**$^{2+}$ (Ar = 4-MeO-2-MeC_6H_3)	*p*-quinoid form **A**	σ-bond form **B**	*o*-diphenoquinoid form **C**
LUMO (eV)	–6.89	–6.68	–7.12
HOMO (eV)	–9.78	–9.96	–10.05
E_{rel} (kcal mol^{-1})	0	+25.49	+3.29
**1b-2H** (Ar = 4-MeOC_6H_4)	*p*-quinoid form **A**	σ-bond form **B**	*o*-diphenoquinoid form **C**
LUMO (eV)	–0.39	–0.27	–0.91
HOMO (eV)	–5.86	–6.10	–5.34
E_{rel} (kcal mol^{-1})	+7.84	0	+14.82
**1c-2H** (Ar = 4-MeO-2-MeC_6H_3)	*p*-quinoid form **A**	σ-bond form **B**	*o*-diphenoquinoid form **C**
LUMO (eV)	–0.28	–0.30	–0.95
HOMO (eV)	–5.90	–6.24	–5.48
E_{rel} (kcal mol^{-1})	+11.87	+40.38	0
**1c-2H**$^{\bullet+}$ (Ar = 4-MeO-2-MeC_6H_3)	Folded form	Twisted form	-
LUMO (eV)	–4.20(α), -5.07(β)	–4.56(α), -4.31(β)	-
HOMO (eV)	–8.24(α), -8.81(β)	–7.67(α), -8.25(β)	-
E_{rel} (kcal mol^{-1})	0	+23.09	-
**1c-2H**$^{2+}$ (Ar = 4-MeO-2-MeC_6H_3)	Folded form	Twisted form	-
LUMO (eV)	–7.74	–7.05	-
HOMO (eV)	–10.72	–10.62	-
E_{rel} (kcal mol^{-1})	+15.94	0	-

Table S14 | Summary of the results of DFT calculations. Relative energies for possible isomers of **1a**$^{2+}$, **1b**$^{2+}$, **1c**$^{2+}$, **1c-2H**, **1a'**$^{2+}$, **1b'**$^{2+}$, **1c'**$^{2+}$, **1a''**$^{2+}$, **1b''**$^{2+}$, **1c''**$^{2+}$, and **1c-2H''** predicted by DFT calculations at the (U)CAM-B3LYP-D3/6-31G* level.

**1a**$^{2+}$ (Ar = 2-F-4-MeOC_6H_3)	*p*-quinoid form **A**	σ-bond form **B**	*o*-diphenoquinoid form **C**	-
E_{rel} (kcal mol^{-1})	0	+5.17	+16.06	-
**1b**$^{2+}$ (Ar = 4-MeOC_6H_4)	*p*-quinoid form **A**	σ-bond form **B**	*o*-diphenoquinoid form **C**	-
E_{rel} (kcal mol^{-1})	+1.94	0	+17.18	-
**1c**$^{2+}$ (Ar = 4-MeO-2-MeC_6H_3)	*p*-quinoid form **A**	σ-bond form **B**	*o*-diphenoquinoid form **C**	-
E_{rel} (kcal mol^{-1})	0	+25.49	+3.29	-
**1c-2H** (Ar = 4-MeO-2-MeC_6H_3)	*p*-quinoid form **A**	σ-bond form **B**	*o*-diphenoquinoid form **C**	-
E_{rel} (kcal mol^{-1})	+11.87	+40.38	0	-
**1a'**$^{2+}$ (Ar = 4-MeO-2-MeC_6H_3) [a]	*p*-quinoid form **A**	σ-bond form **B**	*o*-diphenoquinoid form **C**	-
E_{rel} (kcal mol^{-1})	0	+4.94	+23.75	-
**1b'**$^{2+}$ (Ar = 4-MeOC_6H_4) [a]	*p*-quinoid form **A**	σ-bond form **B**	*o*-diphenoquinoid form **C**	-
E_{rel} (kcal mol^{-1})	0	+1.51	+20.05	-
**1c'**$^{2+}$ (Ar = 4-MeO-2-MeC_6H_3) [a]	*p*-quinoid form **A**	σ-bond form **B**	*o*-diphenoquinoid form **C**	-
E_{rel} (kcal mol^{-1})	0	+32.04	+14.65	-
**1a''**$^{2+}$ (Ar = 4-MeO-2-MeC_6H_3) [b]	*p*-quinoid form **A**	σ-bond form **B**	*o*-diphenoquinoid form **C**	singlet diradical
E_{rel} (kcal mol^{-1})	+11.17	0	- [c]	+5.70 [d]
**1b''**$^{2+}$ (Ar = 4-MeOC_6H_4) [b]	*p*-quinoid form **A**	σ-bond form **B**	*o*-diphenoquinoid form **C**	singlet diradical
E_{rel} (kcal mol^{-1})	+13.70	0	- [c]	+4.64 [d]
**1c''**$^{2+}$ (Ar = 4-MeO-2-MeC_6H_3) [b]	*p*-quinoid form **A**	σ-bond form **B**	*o*-diphenoquinoid form **C**	singlet diradical
E_{rel} (kcal mol^{-1})	+3.99	+22.93	- [c]	0 [d]
**1c-2H''** (Ar = 4-MeO-2-MeC_6H_3)	-	-	*o*-diphenoquinoid form **C**	singlet diradical
E_{rel} (kcal mol^{-1})	-	-	+4.58	0 [d]

[a] Reference compounds without sulfur bridge.

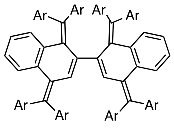

1a'–1c'

[b] Reference compounds without benzofusion.

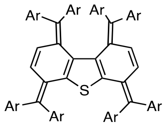

1a''–1c''

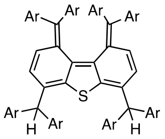

1c-2H''

[c] DFT calculations with *o*-diphenoquinoid form **C** as the initial structure instead yielded *p*-quinoid form **A** resulting from the equilibrium structure due to structural relaxation.

[d] Single-point calculations were performed using the optimized structure of triplet diradical species.

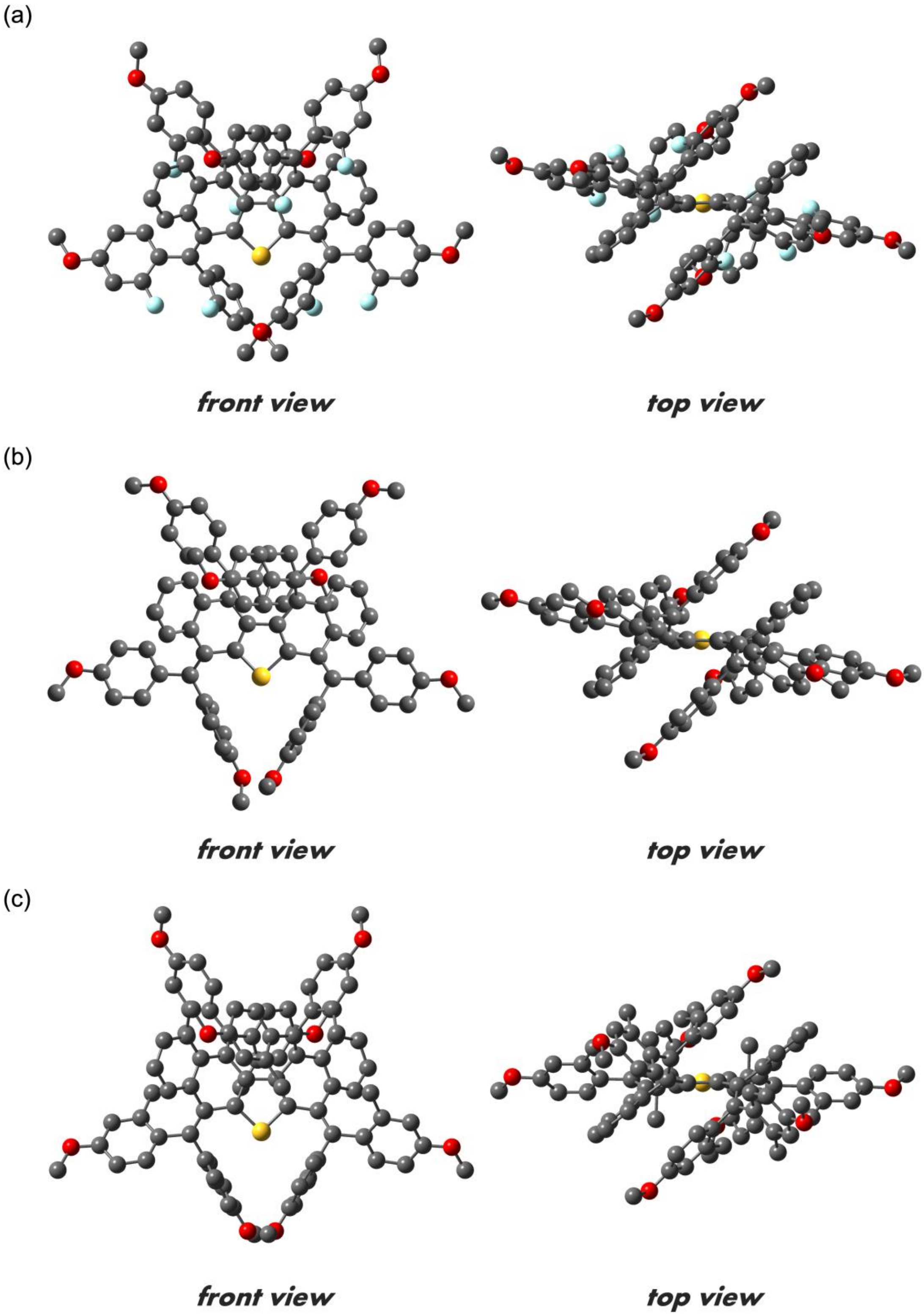

Figure S26 | Predicted geometries. Optimized structures of neutral donors (a) **1a**, (b) **1b**, and (c) **1c** obtained by DFT calculations at the CAM-B3LYP-D3/6-31G* level. Hydrogen atoms are omitted for clarity. [**1a**: Ar = 2-F-4-MeOC_6H_3, **1b**: Ar = 4-MeOC_6H_4, **1c**: Ar = 4-MeO-2-MeC_6H_3]

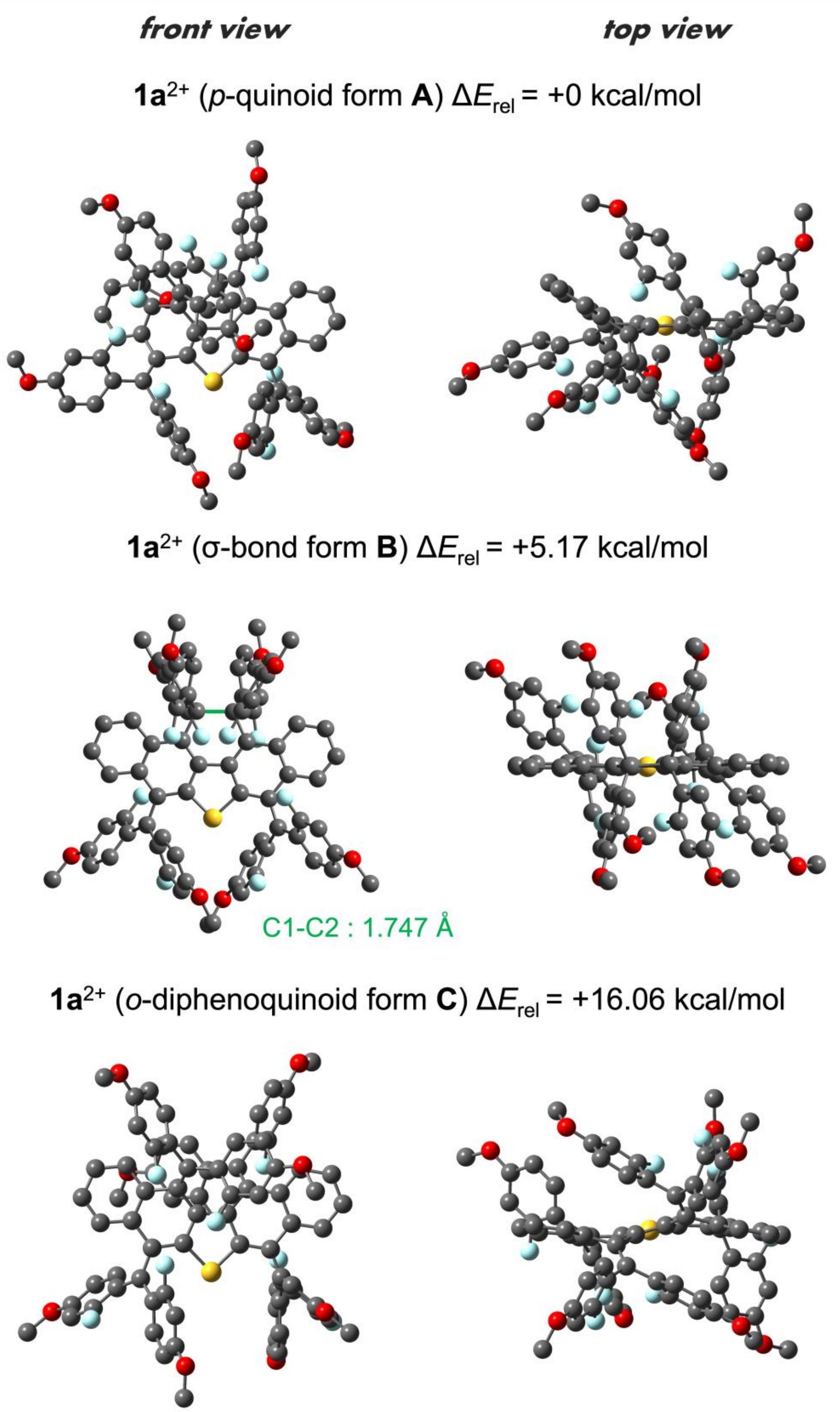

Figure S27 | Possible structures. Optimized structures and their relative energies of possible isomers for dication **1a**$^{2+}$ obtained by DFT calculations at the CAM-B3LYP-D3/6-31G* level (0 kcal/mol for *p*-quinoid form **A**). Hydrogen atoms are omitted for clarity. [**1a**$^{2+}$: Ar = 2-F-4-MeOC_6H_3]

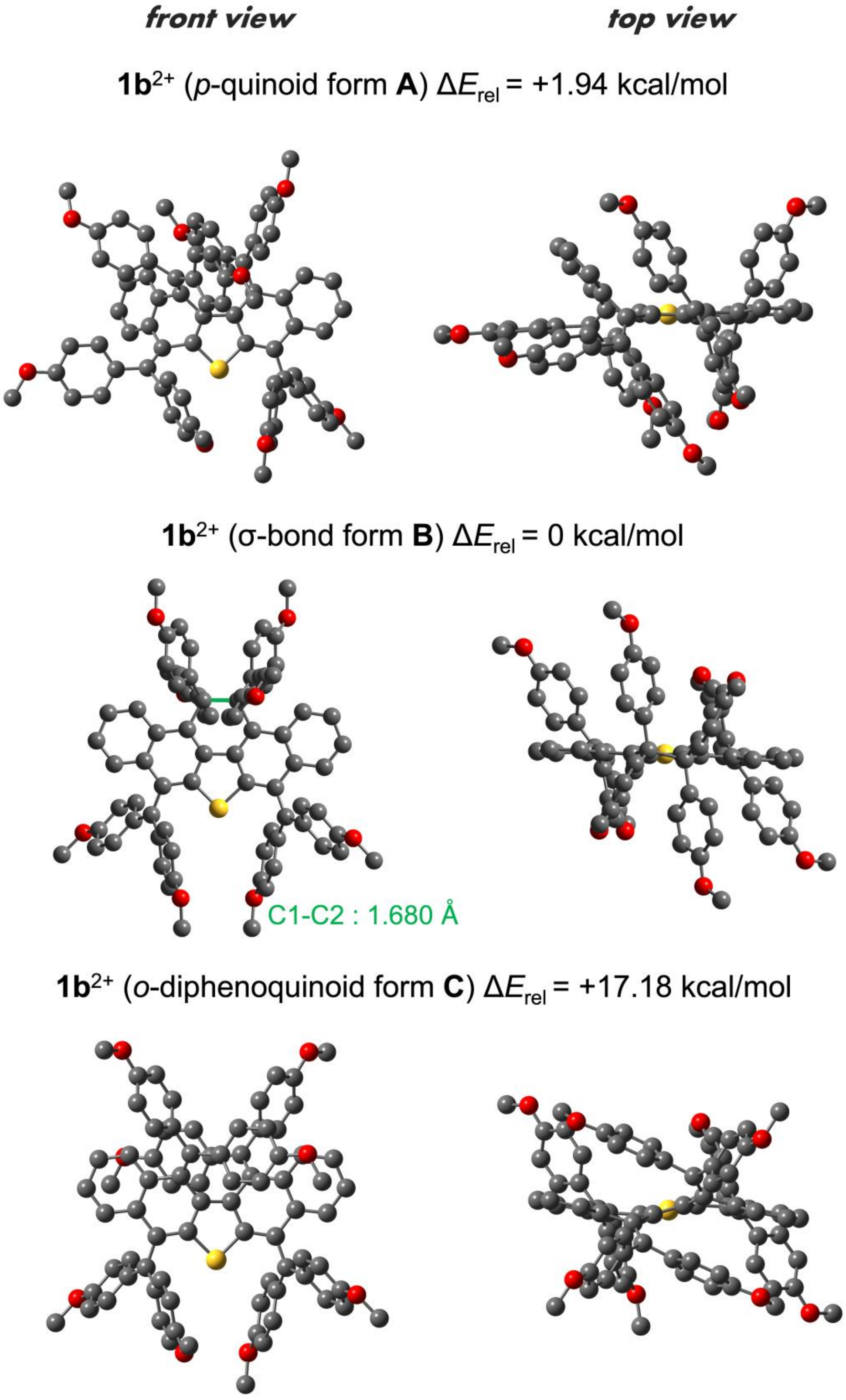

Figure S28 | Possible structures. Optimized structures and their relative energies of possible isomers for dication **1b**$^{2+}$ obtained by DFT calculations at the CAM-B3LYP-D3/6-31G* level (0 kcal/mol for σ-bond form **B**). Hydrogen atoms are omitted for clarity. [**1b**$^{2+}$: Ar = 4-MeOC_6H_4]

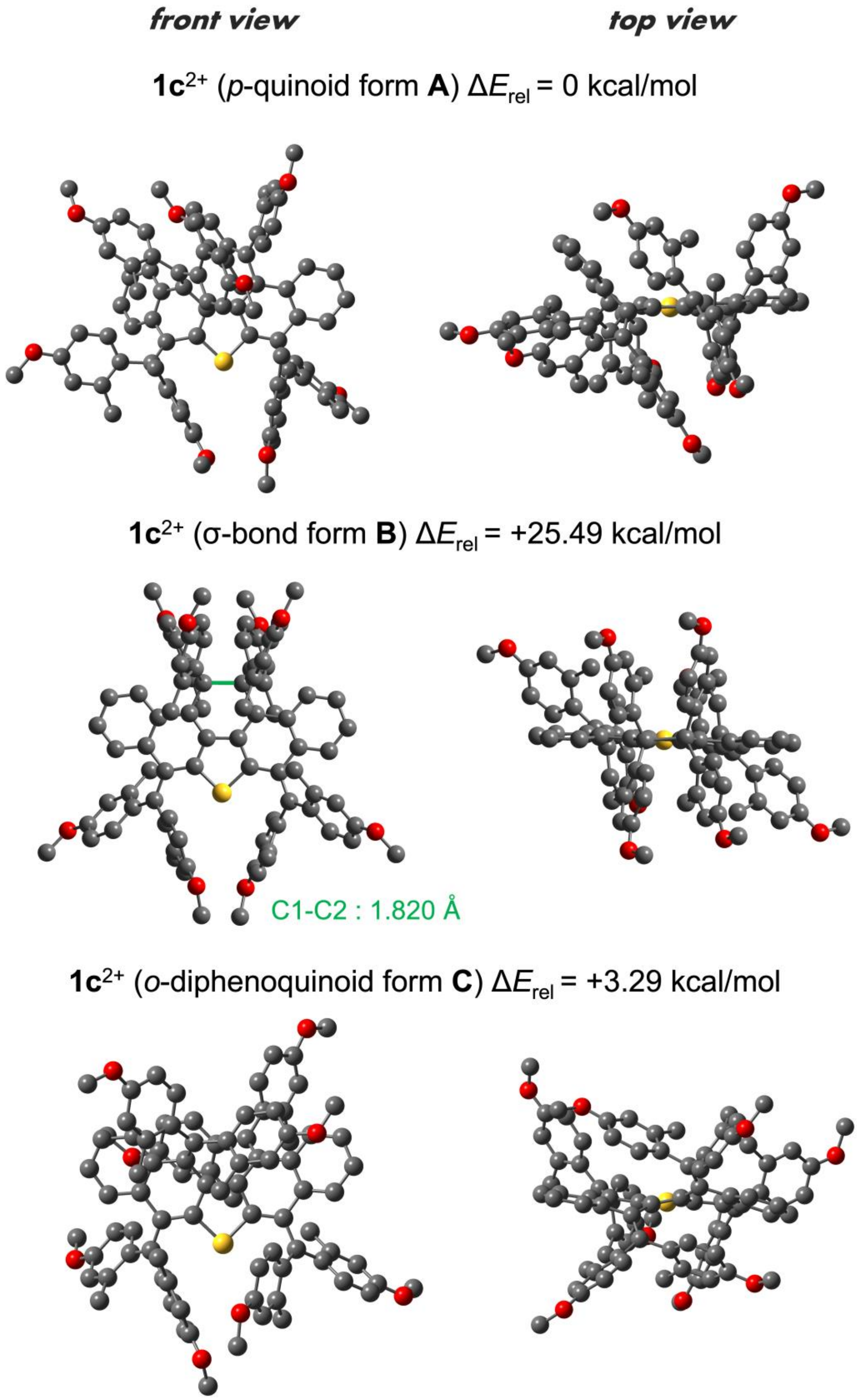

Figure S29 | Possible structures. Optimized structures and their relative energies of possible isomers for dication **1c**$^{2+}$ obtained by DFT calculations at the CAM-B3LYP-D3/6-31G* level (0 kcal/mol for *p*-quinoid form **A**). Hydrogen atoms are omitted for clarity. [**1c**$^{2+}$: Ar = 4-MeO-2-MeC_6H_3]

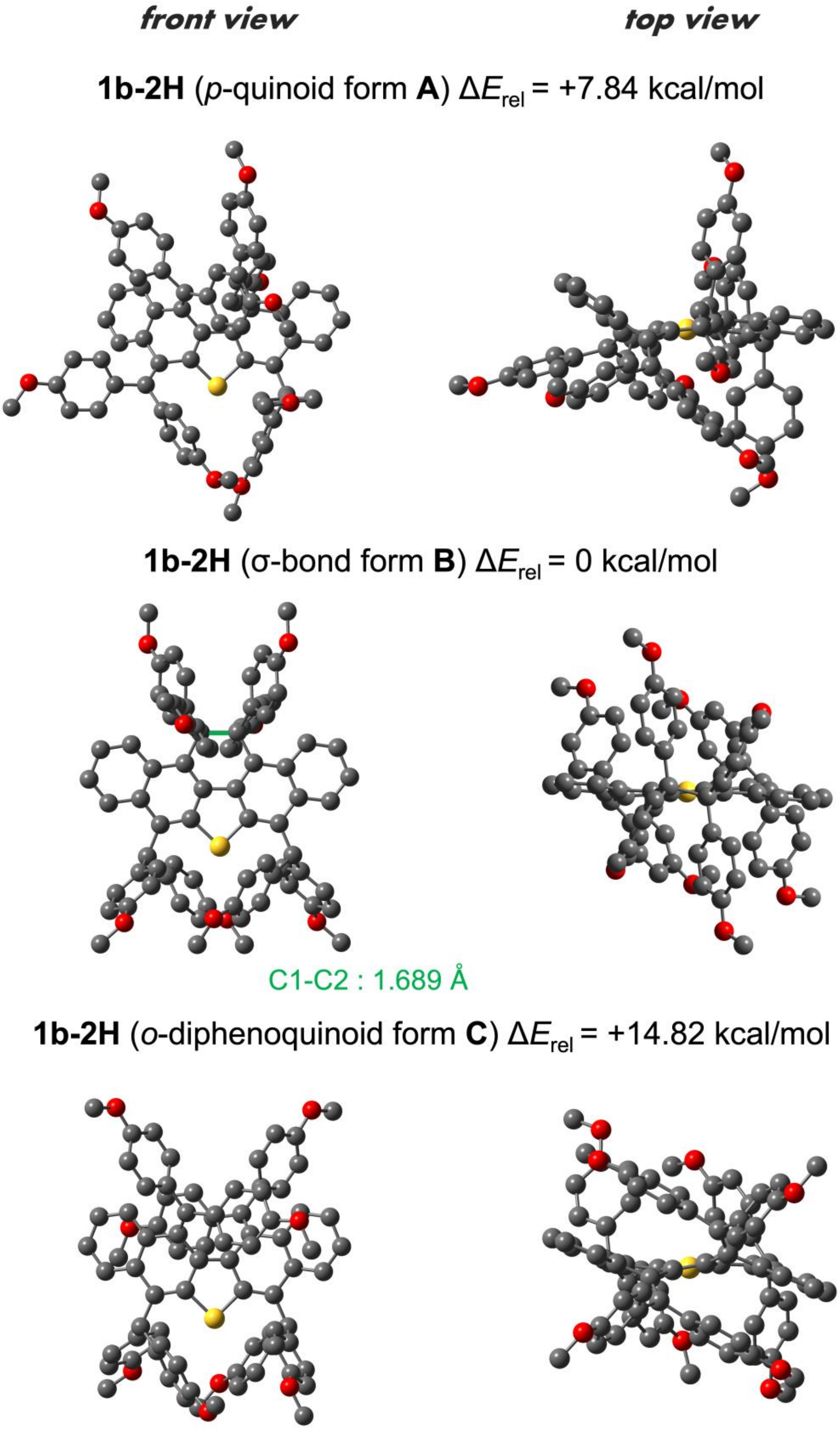

Figure S30 | Possible structures. Optimized structures and their relative energies of possible isomers for hydride adduct **1b-2H** obtained by DFT calculations at the CAM-B3LYP-D3/6-31G* level (0 kcal/mol for σ-bond form **B**). Hydrogen atoms are omitted for clarity. [**1b-2H**: Ar = 4-MeOC_6H_4]

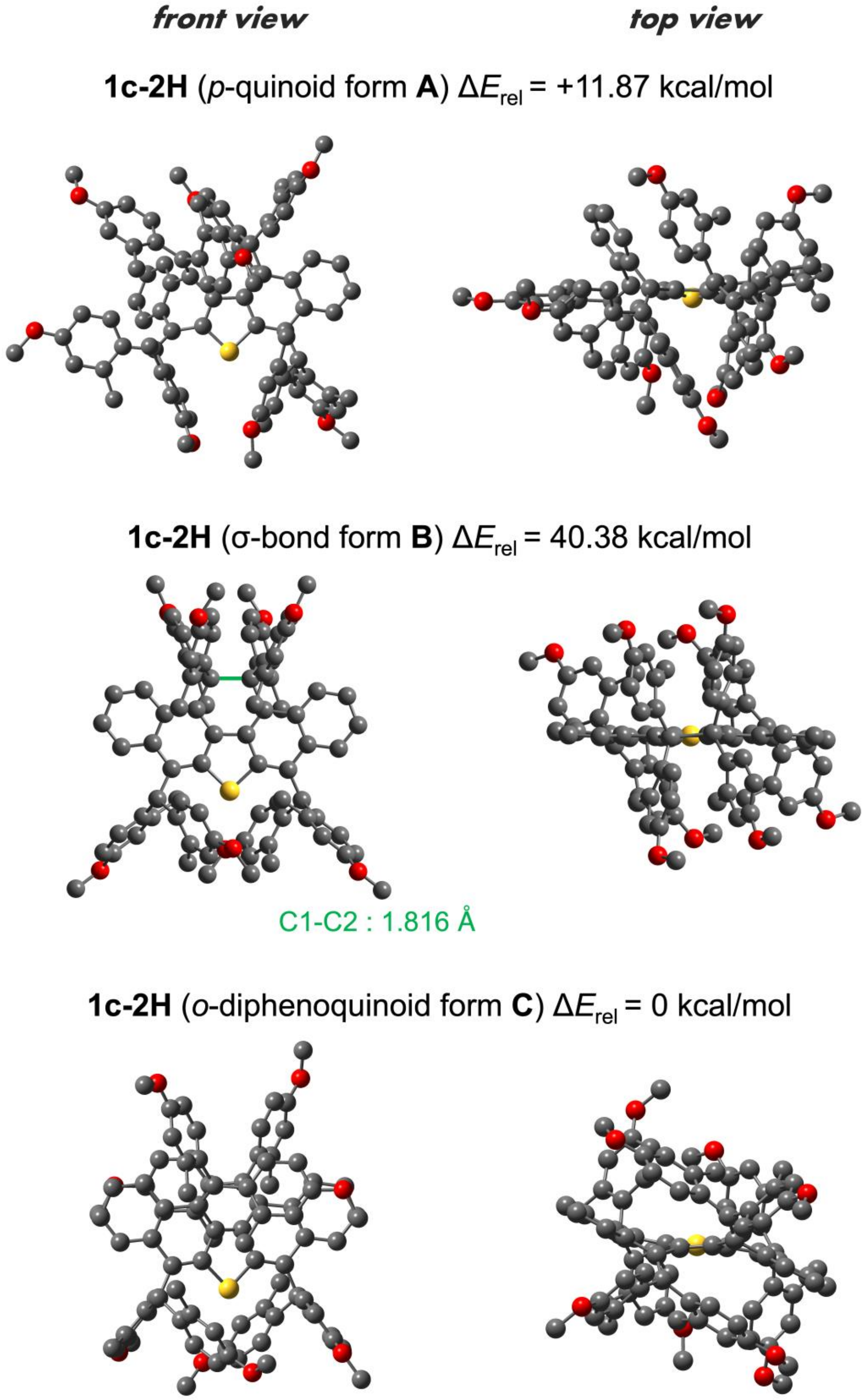

Figure S31 | Possible structures. Optimized structures and their relative energies of possible isomers for hydride adduct **1c-2H** obtained by DFT calculations at the CAM-B3LYP-D3/6-31G* level (0 kcal/mol for *o*-diphenoquinoid form **C**). Hydrogen atoms are omitted for clarity. [**1c-2H**: Ar = 4-MeO-2-MeC_6H_3]

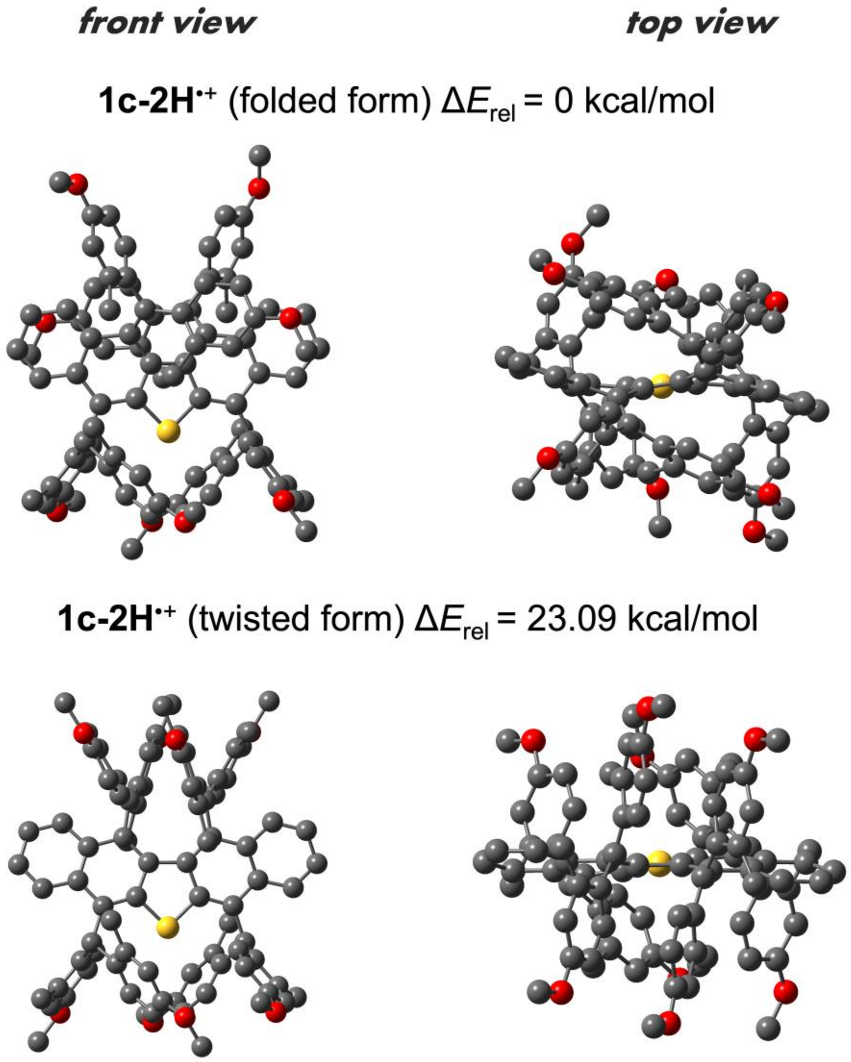

Figure S32 | Possible structures. Optimized structures and their relative energies of possible isomers for **1c-2H**$^{\bullet+}$ obtained by DFT calculations at the UCAM-B3LYP-D3/6-31G* level (0 kcal/mol for folded form). Hydrogen atoms are omitted for clarity. [**1c-2H**: Ar = 4-MeO-2-MeC_6H_3]

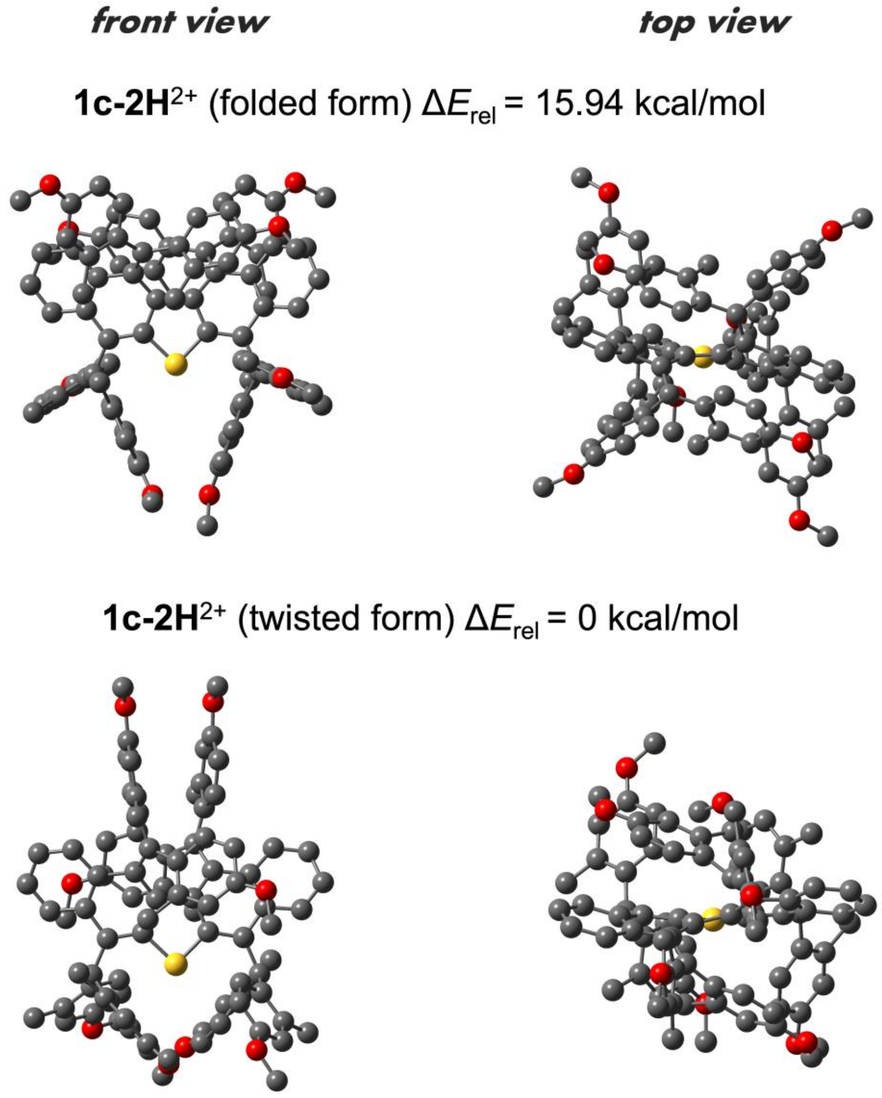

Figure S33 | Possible structures. Optimized structures and their relative energies of possible isomers for **1c-2H**$^{2+}$ obtained by DFT calculations at the CAM-B3LYP-D3/6-31G* level (0 kcal/mol for twisted form). Hydrogen atoms are omitted for clarity. [**1c-2H**: Ar = 4-MeO-2-MeC_6H_3]

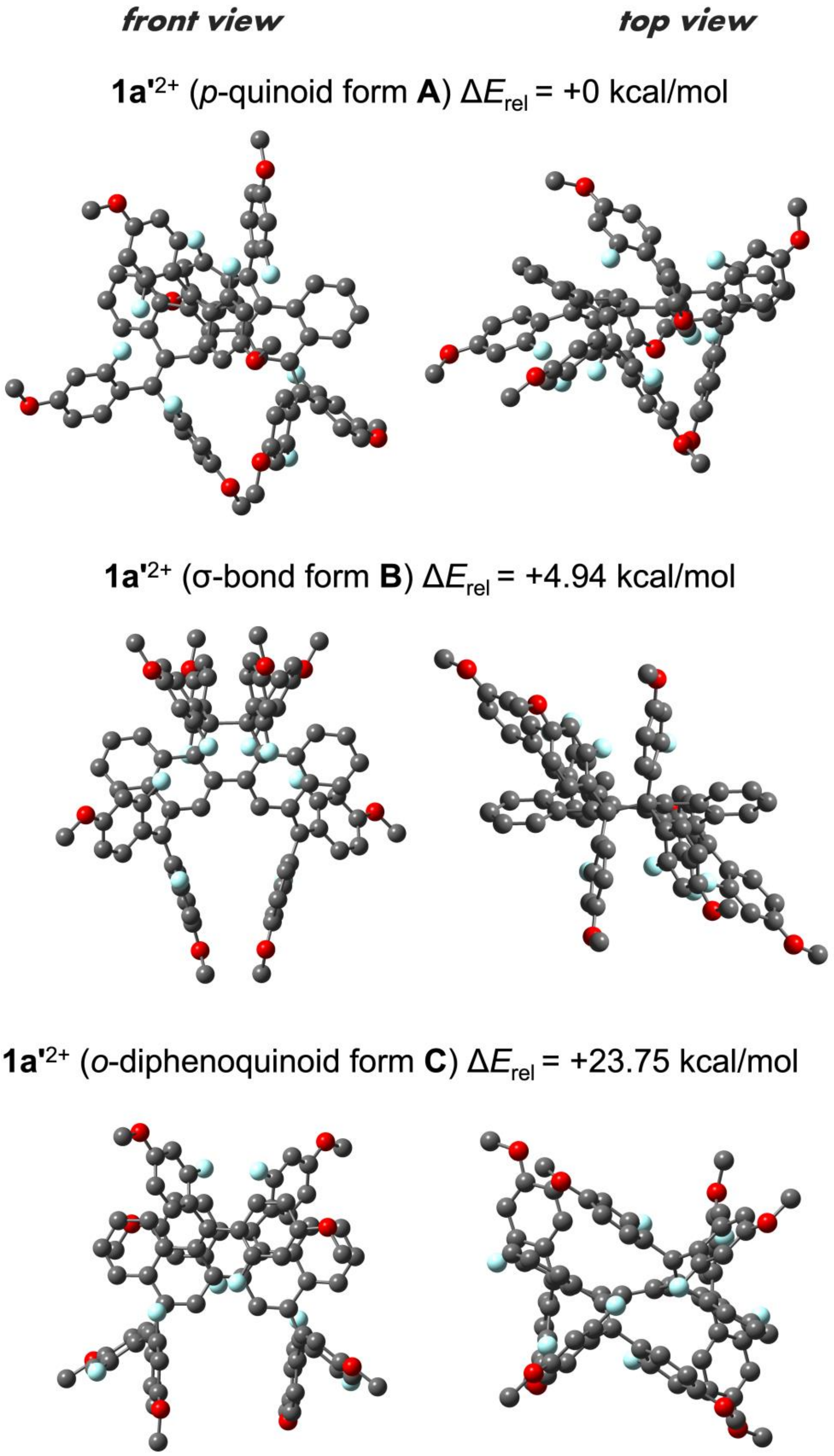

Figure S34 | Possible structures. Optimized structures and their relative energies of possible isomers for reference dication $\mathbf{1a'}^{2+}$ obtained by DFT calculations at the CAM-B3LYP-D3/6-31G* level (0 kcal/mol for *p*-quinoid form **A**). Hydrogen atoms are omitted for clarity. [$\mathbf{1a'}^{2+}$: Ar = 2-F-4-MeOC_6H_3]

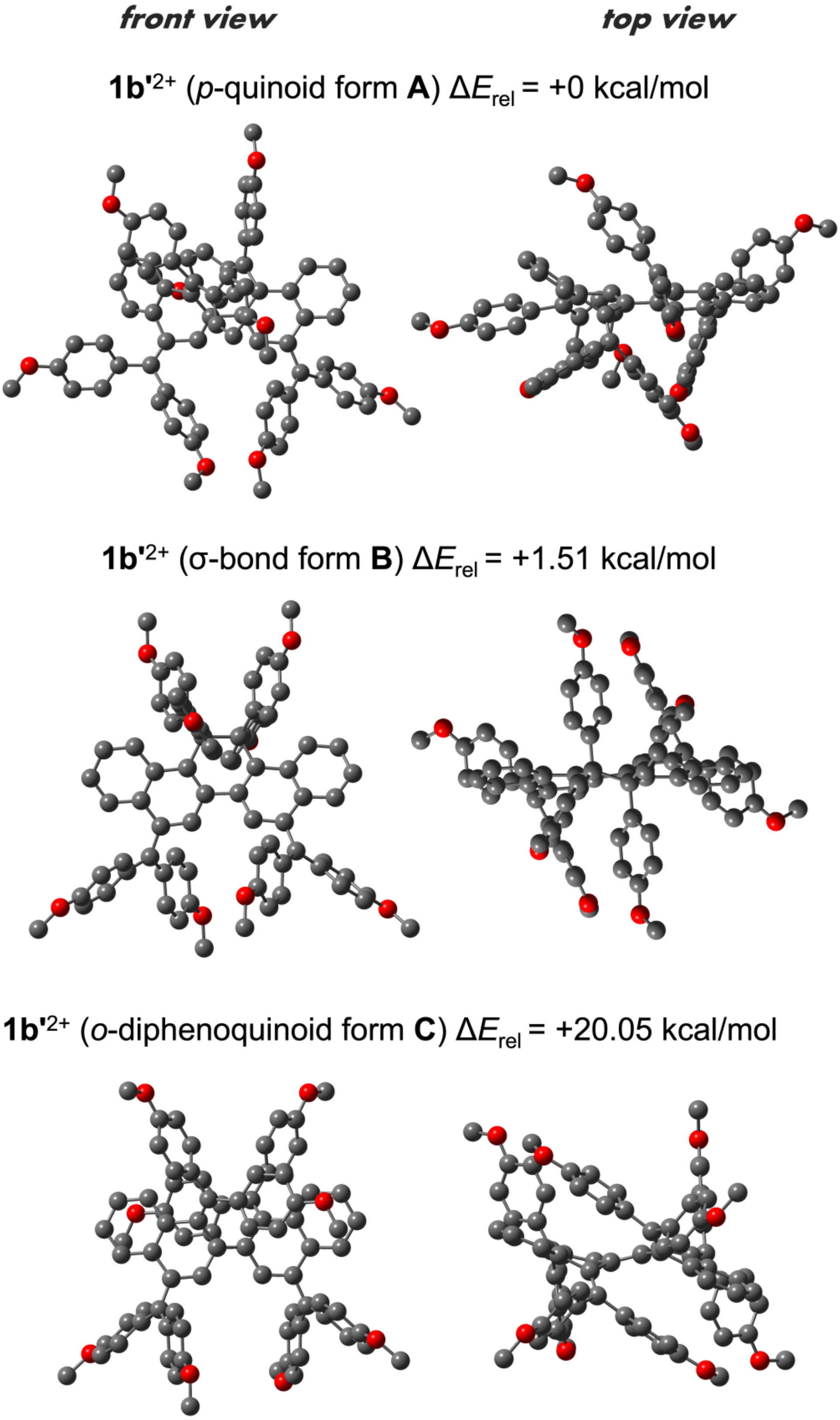

Figure S35 | Possible structures. Optimized structures and their relative energies of possible isomers for reference dication **1b**$'^{2+}$ obtained by DFT calculations at the CAM-B3LYP-D3/6-31G* level (0 kcal/mol for *p*-quinoid form **A**). Hydrogen atoms are omitted for clarity. [**1b**$'^{2+}$: Ar = 4-MeOC_6H_4]

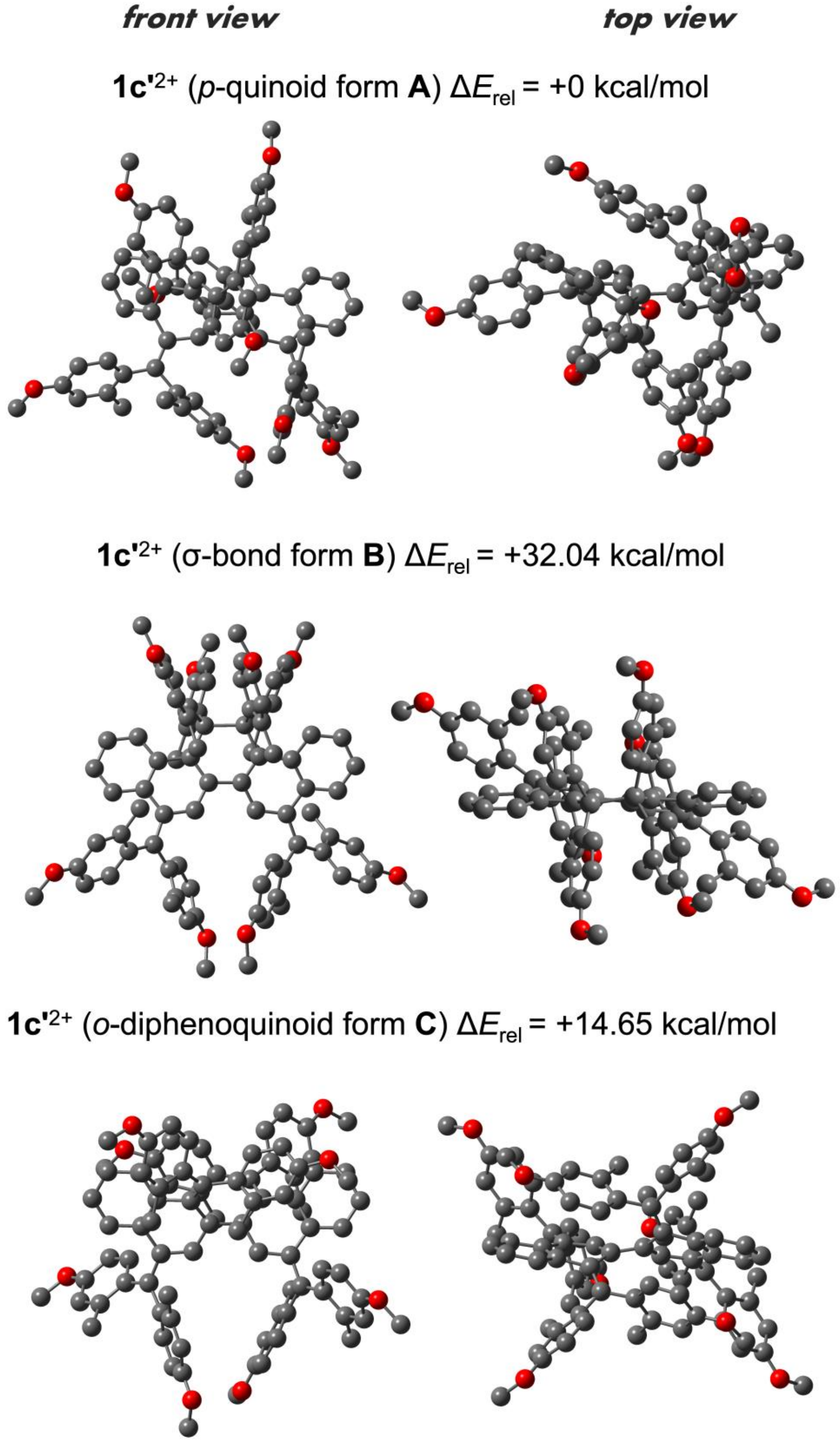

Figure S36 | Possible structures. Optimized structures and their relative energies of possible isomers for reference dication **1c**$'^{2+}$ obtained by DFT calculations at the CAM-B3LYP-D3/6-31G* level (0 kcal/mol for *p*-quinoid form **A**). Hydrogen atoms are omitted for clarity. [**1c**$'^{2+}$: Ar = 4-MeO-2-MeC_6H_3]

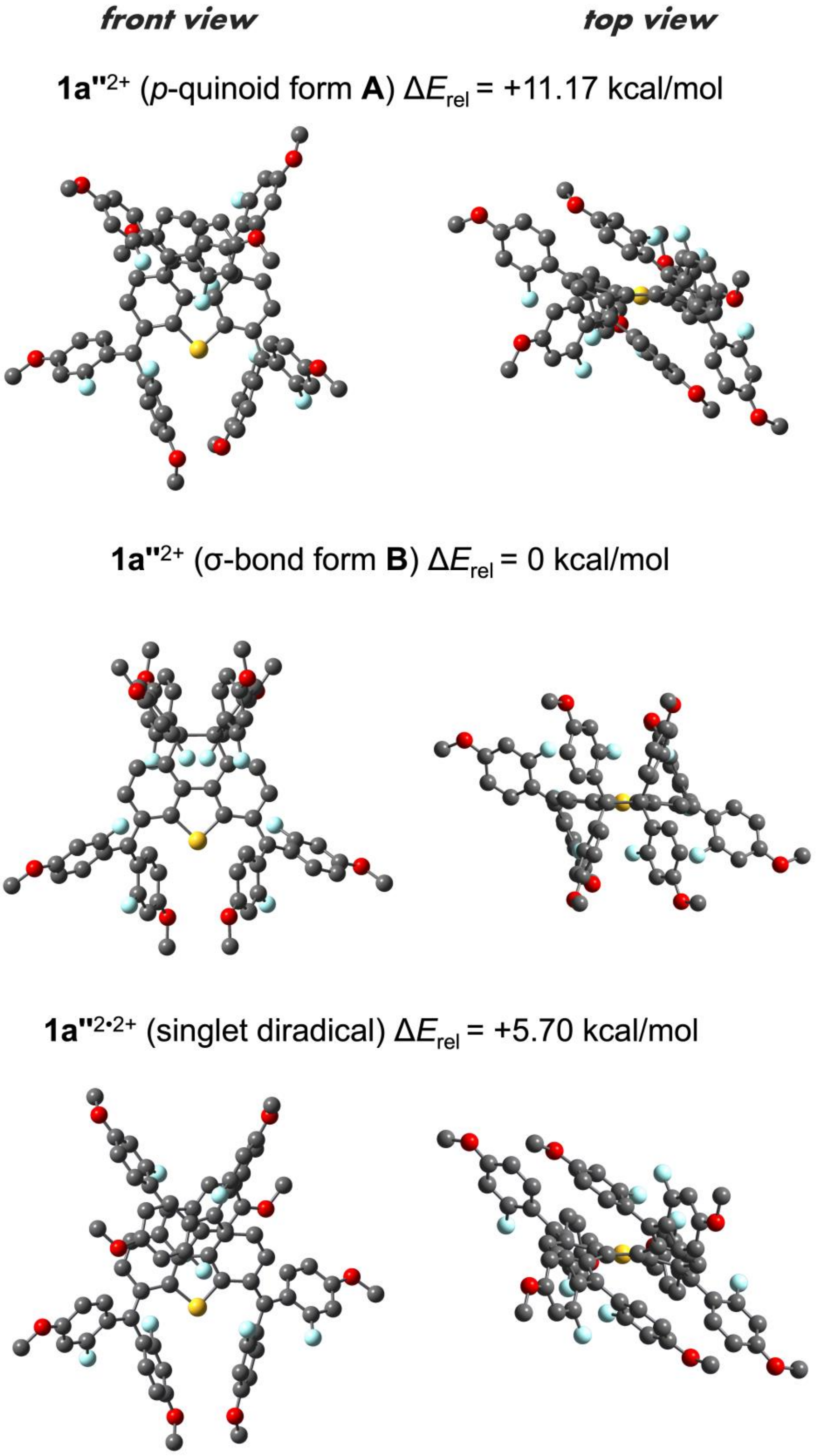

Figure S37 | Possible structures. Optimized structures and their relative energies of possible isomers for reference dication **1a**''$^{2+}$ obtained by DFT calculations at the (U)CAM-B3LYP-D3/6-31G* level (0 kcal/mol for σ-bond form **B**). Hydrogen atoms are omitted for clarity. [**1a**''$^{2+}$: Ar = 2-F-4-MeOC$_6$H$_3$]

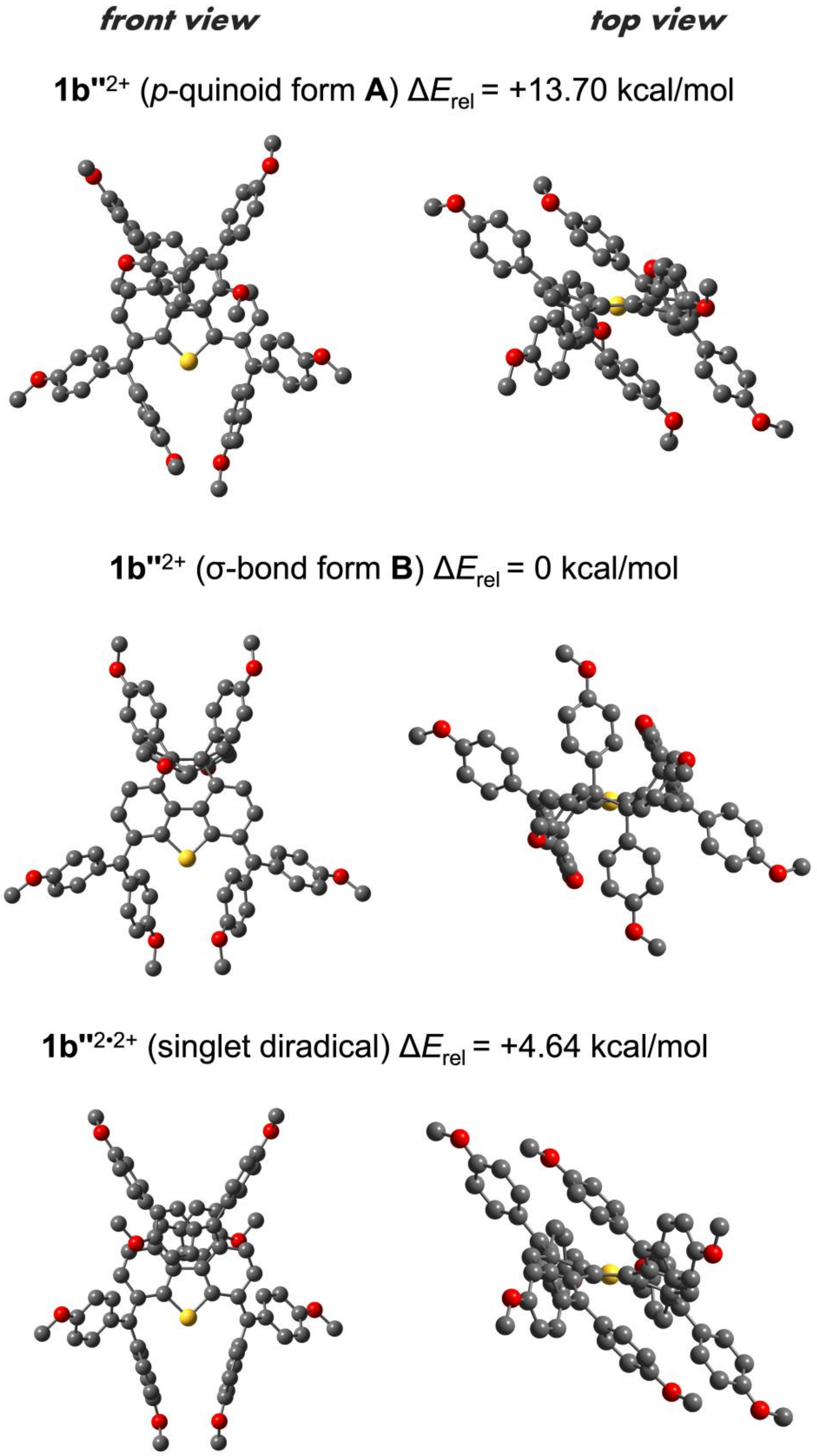

Figure S38 | Possible structures. Optimized structures and their relative energies of possible isomers for reference dication **1b**''$^{2+}$ obtained by DFT calculations at the (U)CAM-B3LYP-D3/6-31G* level (0 kcal/mol for σ-bond form **B**). Hydrogen atoms are omitted for clarity. [**1b**''$^{2+}$: Ar = 4-MeOC_6H_4]

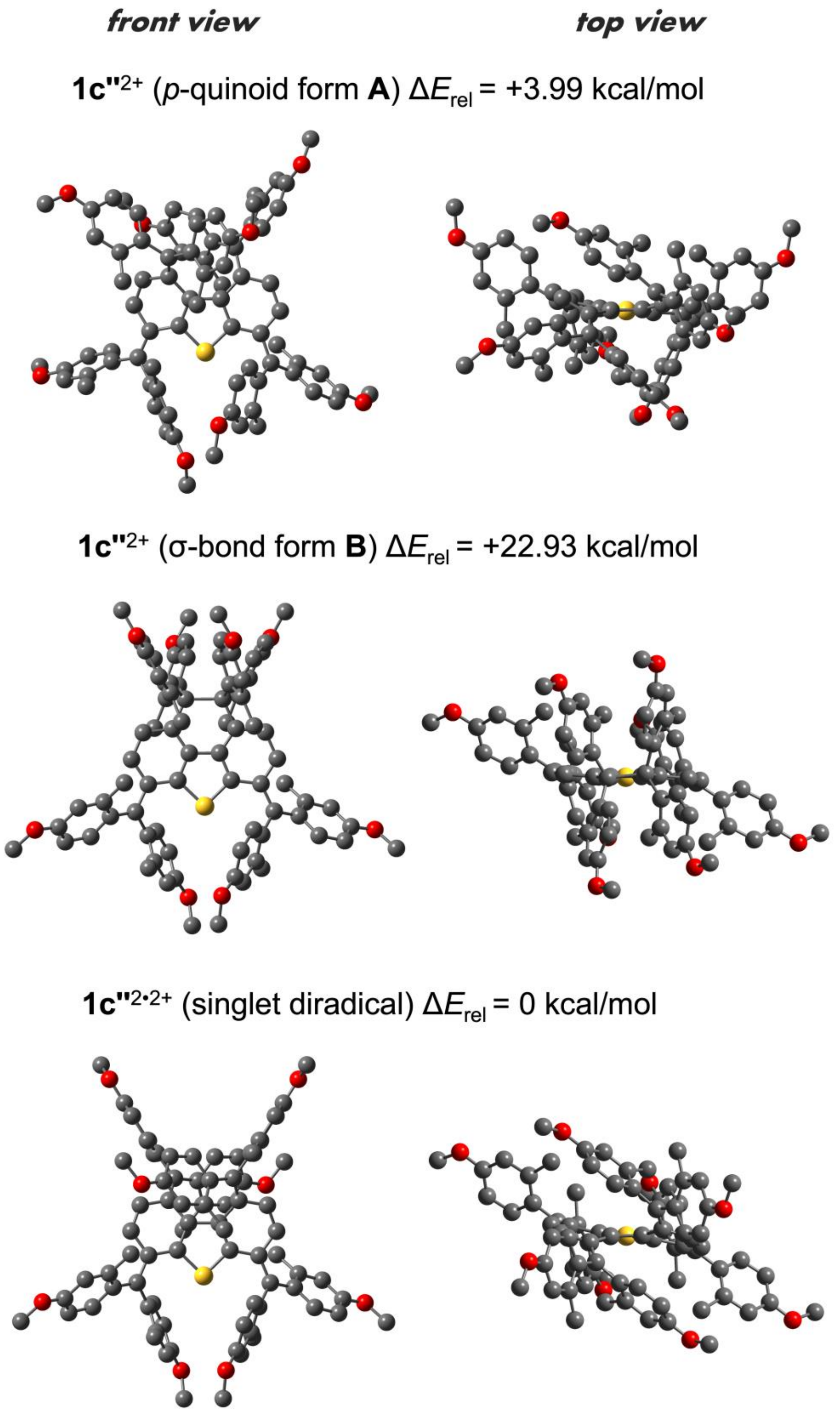

Figure S39 | Possible structures. Optimized structures and their relative energies of possible isomers for reference dication **1c**''$^{2+}$ obtained by DFT calculations at the (U)CAM-B3LYP-D3/6-31G* level (0 kcal/mol for singlet diradical). Hydrogen atoms are omitted for clarity. [**1c**''$^{2+}$: Ar = 4-MeO-2-MeC$_6$H$_3$]

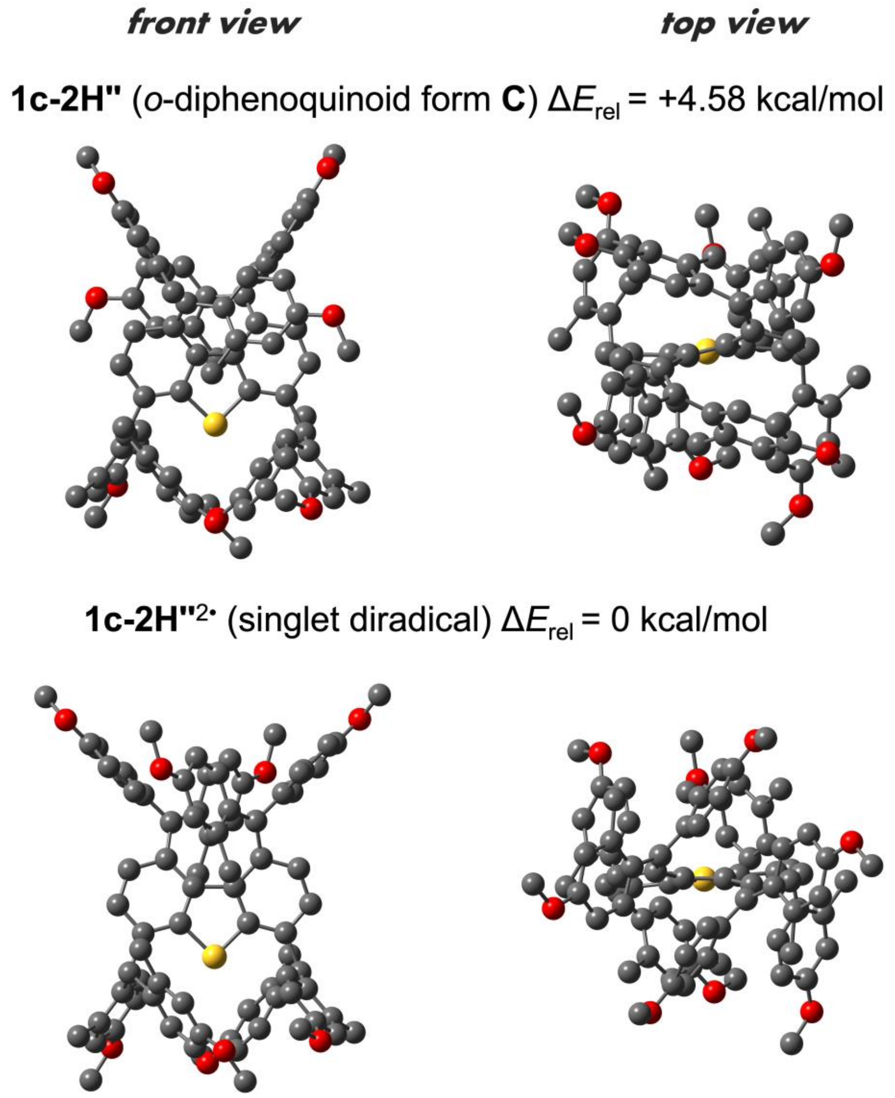

Figure S40 | Possible structures. Optimized structures and their relative energies of possible isomers for reference hydride adduct **1c-2H**'' obtained by DFT calculations at the (U)CAM-B3LYP-D3/6-31G* level (0 kcal/mol for singlet diradical). Hydrogen atoms are omitted for clarity. [**1c-2H**'': Ar = 4-MeO-2-MeC_6H_3]

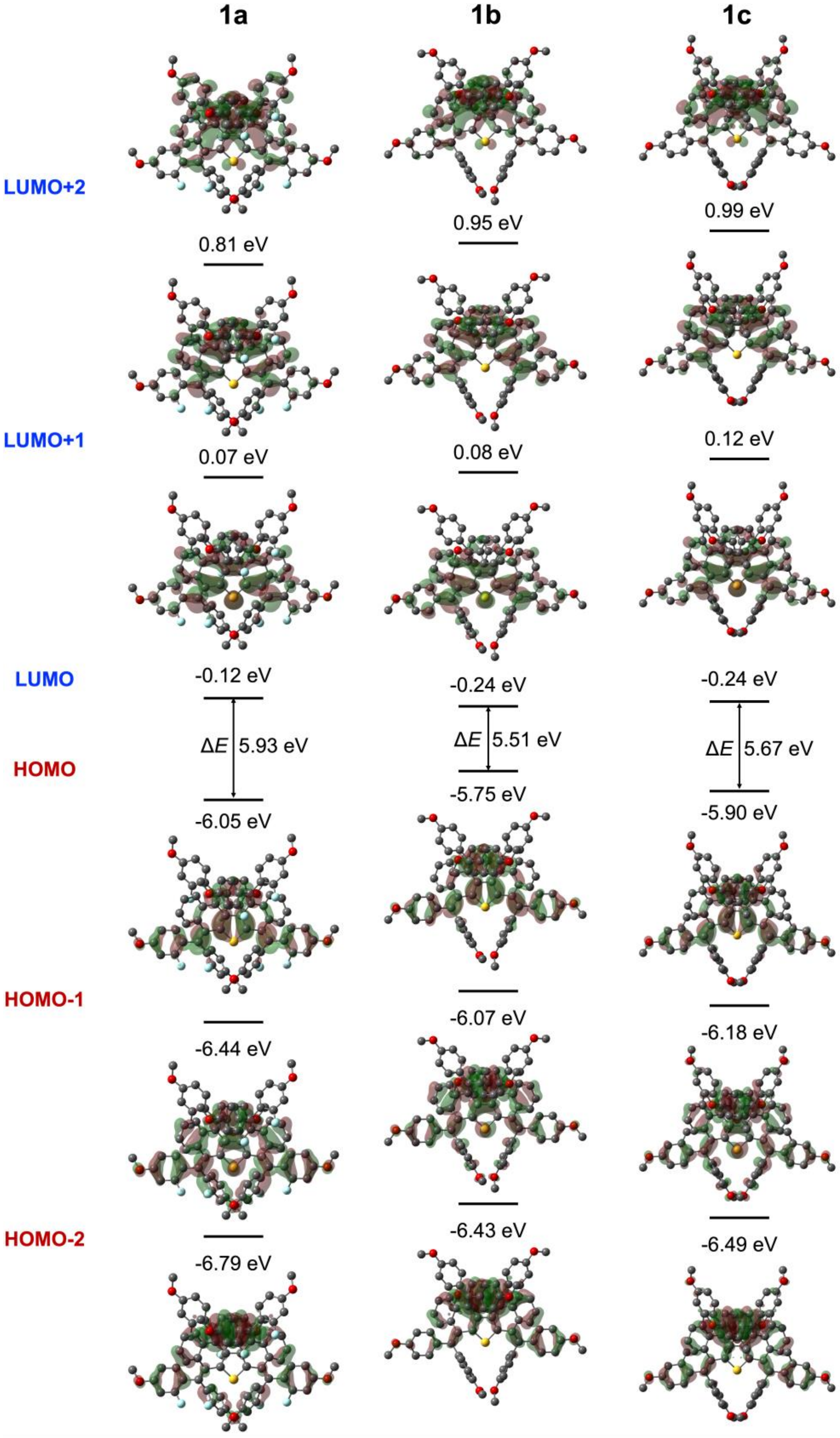

Figure S41 | Kohn–Sham orbitals. HOMO and LUMO levels calculated by the DFT method (CAM-B3LYP-D3/6-31G*) based on the optimized structures of neutral donors **1**. [**1a**: Ar = 2-F-4-MeOC_6H_3, **1b**: Ar = 4-MeOC_6H_4, **1c**: Ar = 4-MeO-2-MeC_6H_3]

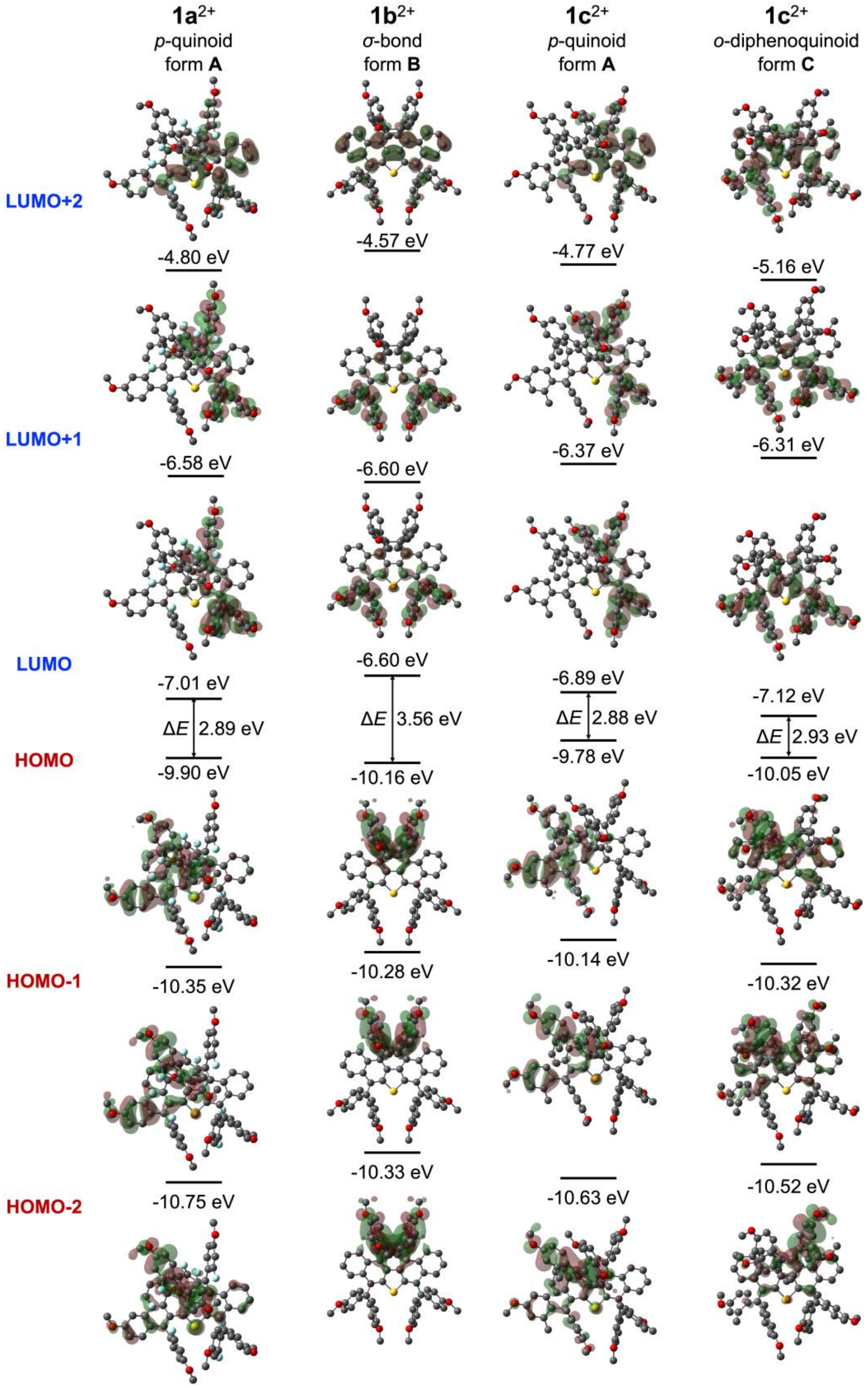

Figure S42 | Kohn–Sham orbitals. HOMO and LUMO levels calculated by the DFT method (CAM-B3LYP-D3/6-31G*) based on the optimized structures of dications $\mathbf{1}^{2+}$. [$\mathbf{1a}^{2+}$: Ar = 2-F-4-MeOC_6H_3, $\mathbf{1b}^{2+}$: Ar = 4-MeOC_6H_4, $\mathbf{1c}^{2+}$: Ar = 4-MeO-2-MeC_6H_3]

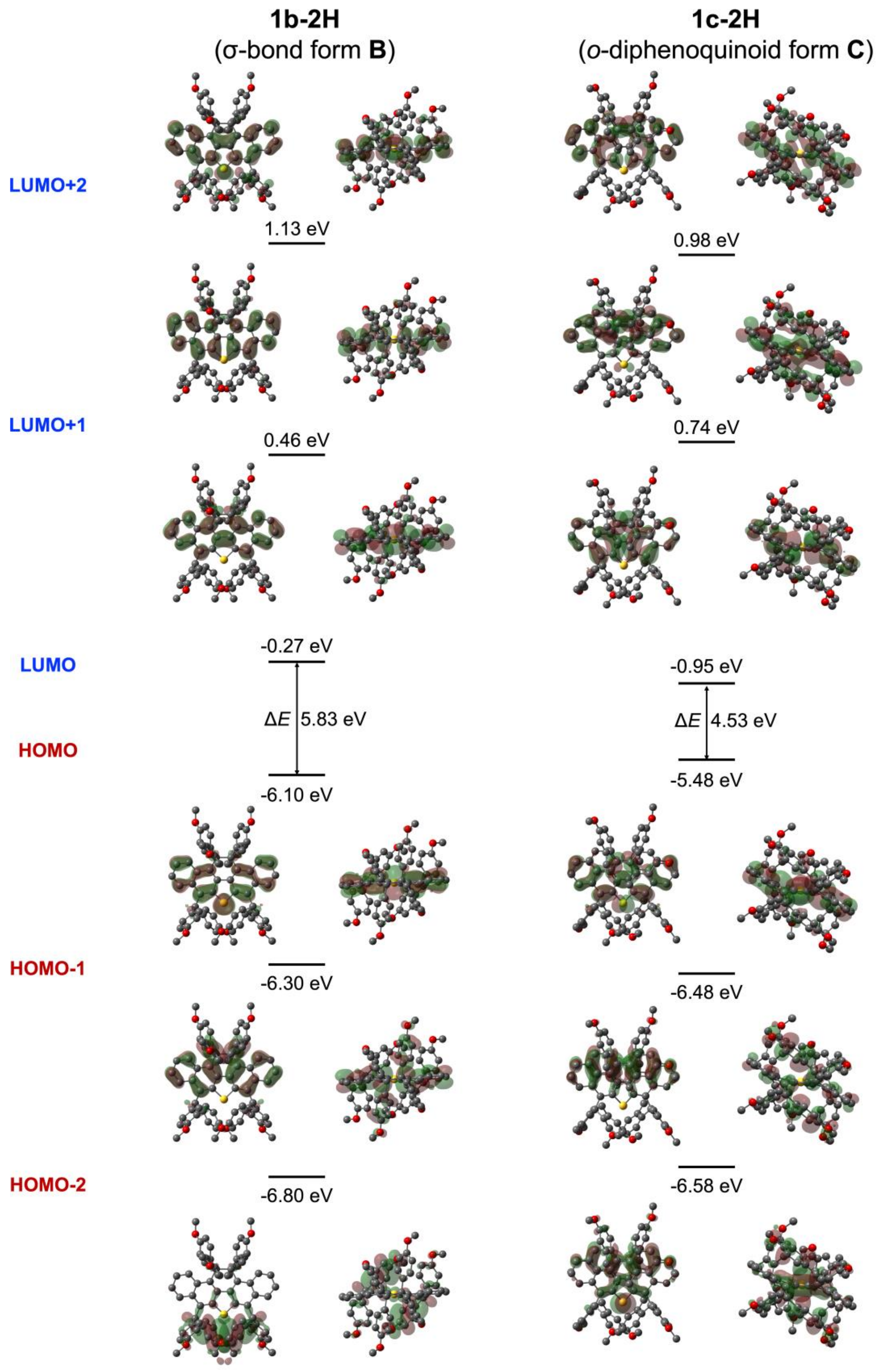

Figure S43 | Kohn–Sham orbitals. HOMO and LUMO levels calculated by the DFT method (CAM-B3LYP-D3/6-31G*) based on the optimized structures of hydride adducts **1-2H**. [**1b-2H**: Ar = 4-$MeOC_6H_4$, **1c-2H**: Ar = 4-MeO-2-MeC_6H_3]

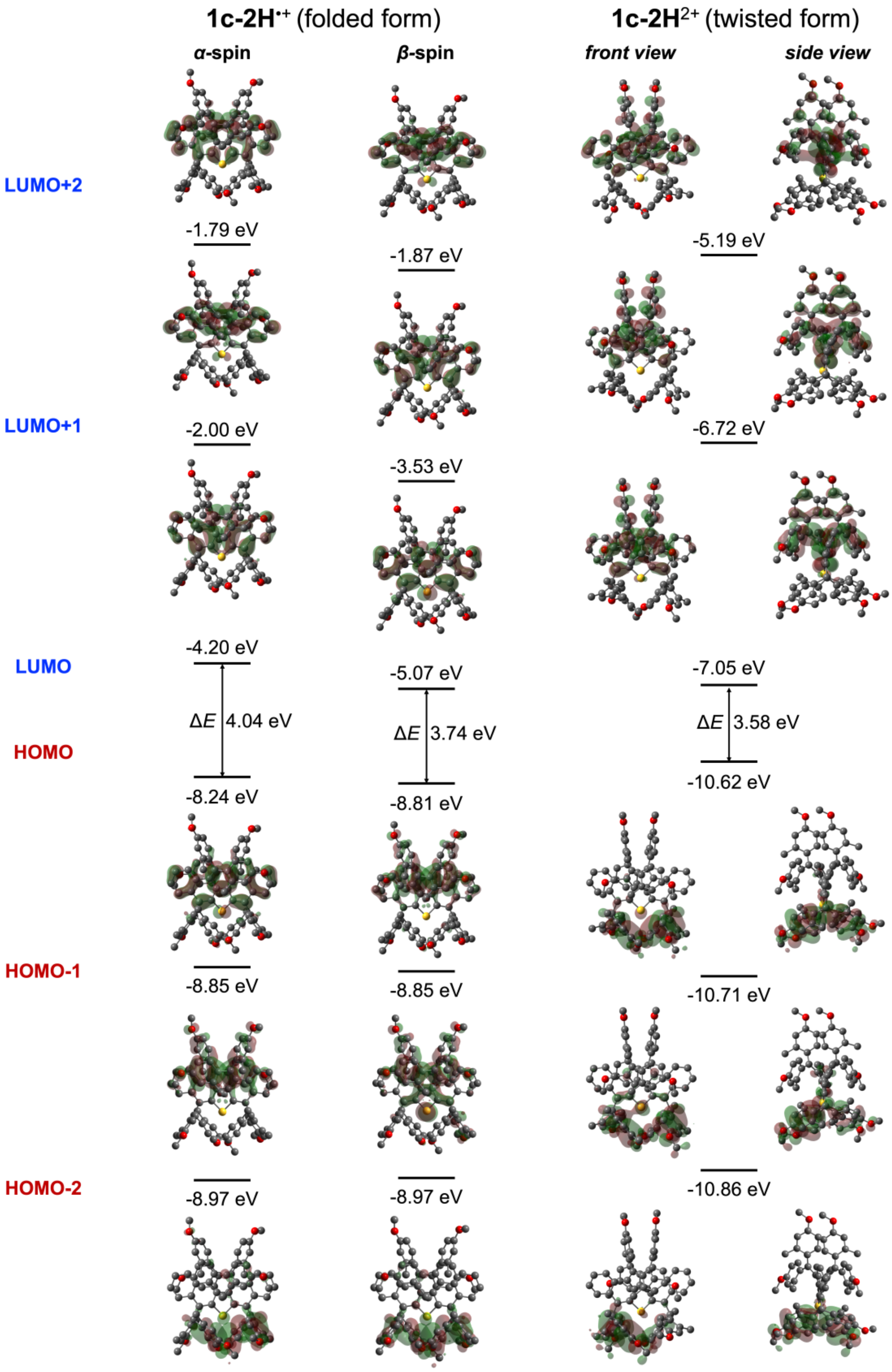

Figure S44 | Kohn–Sham orbitals. HOMO and LUMO levels calculated by the DFT method [(U)CAM-B3LYP-D3/6-31G*] based on the optimized structures of **1c-2H**$^{\bullet+}$ and **1c-2H**$^{2+}$. [**1c-2H**: Ar = 4-MeO-2-MeC_6H_3]

TD-DFT calculations (Figures S45-S47)

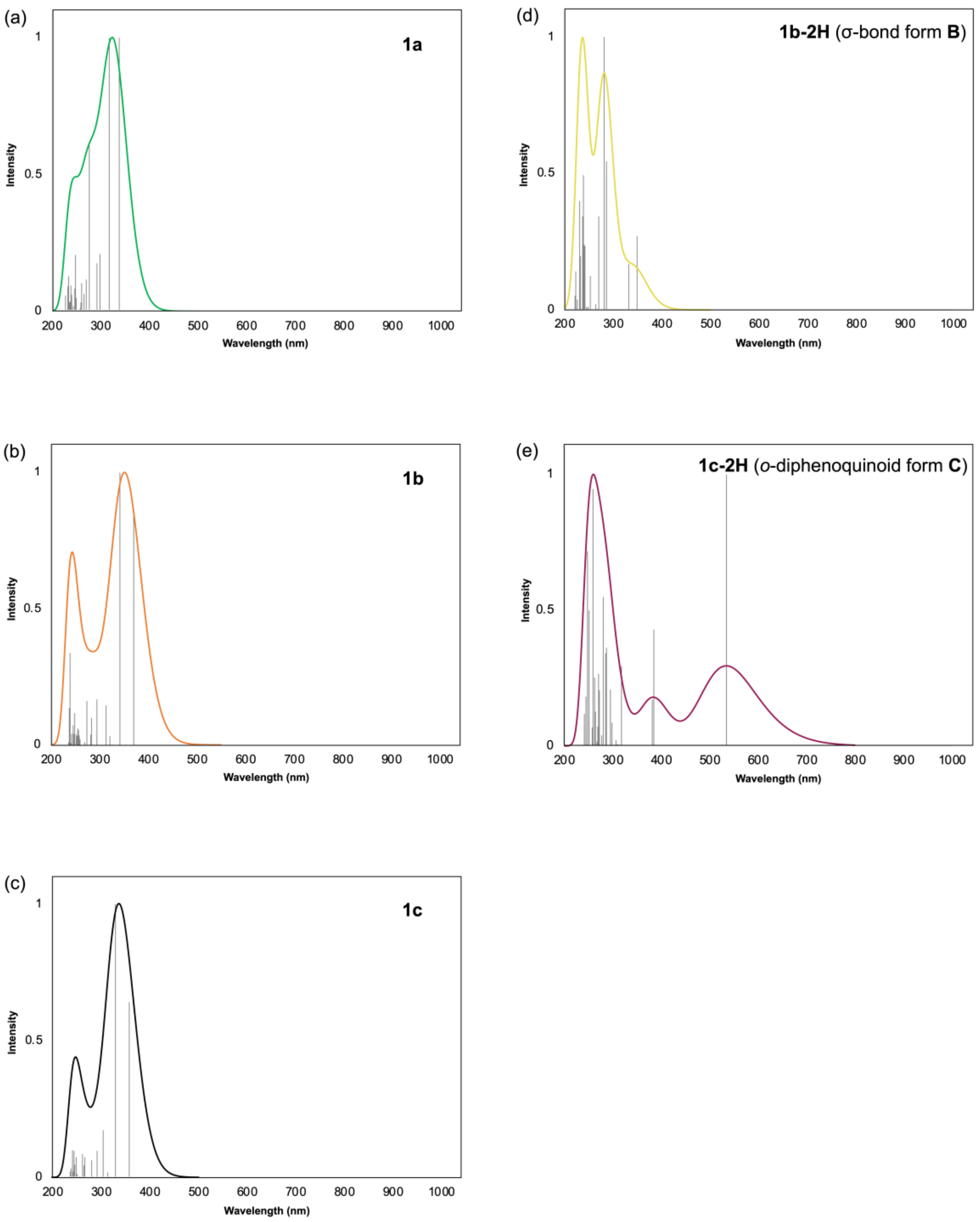

Figure S45 | Predicted electronic absorptions. Simulated UV−vis−NIR spectra by TD-DFT calculations (CAM-B3LYP-D3/6-31G*) for (a) **1a**, (b) **1b**, (c) **1c**, (d) **1b-2H** (σ-bond form **B**), (e) **1c-2H** (*o*-diphenoquinoid form **C**). [**1a**: Ar = 2-F-4-MeOC_6H_3, **1b**: Ar = 4-MeOC_6H_4, **1c**: Ar = 4-MeO-2-MeC_6H_3]

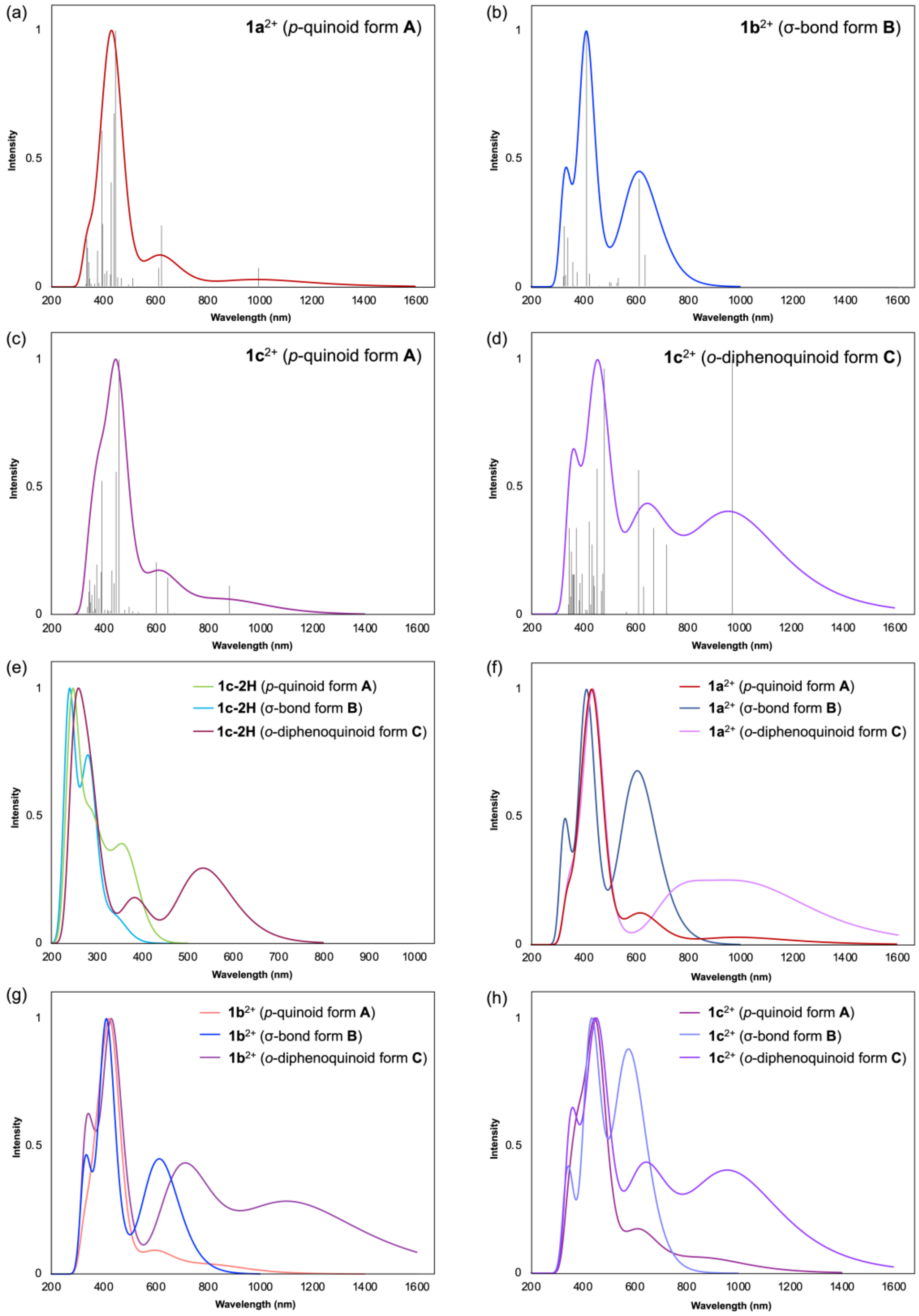

Figure S46 | Predicted electronic absorptions. Simulated UV−vis−NIR spectra by TD-DFT calculations (CAM-B3LYP-D3/6-31G*) for (a) **1a**$^{2+}$ (*p*-quinoid form **A**), (b) **1b**$^{2+}$ (σ-bond form **B**), (c) **1c**$^{2+}$ (*p*-quinoid form **A**), (d) **1c**$^{2+}$ (*o*-diphenoquinoid form **C**), and three forms **A**, **B**, and **C** of (e) **1c-2H**, (f) **1a**$^{2+}$, (g) **1b**$^{2+}$, and (h) **1c**$^{2+}$. [**1a**: Ar = 2-F-4-MeOC_6H_3, **1b**: Ar = 4-MeOC_6H_4, **1c**: Ar = 4-MeO-2-MeC_6H_3]

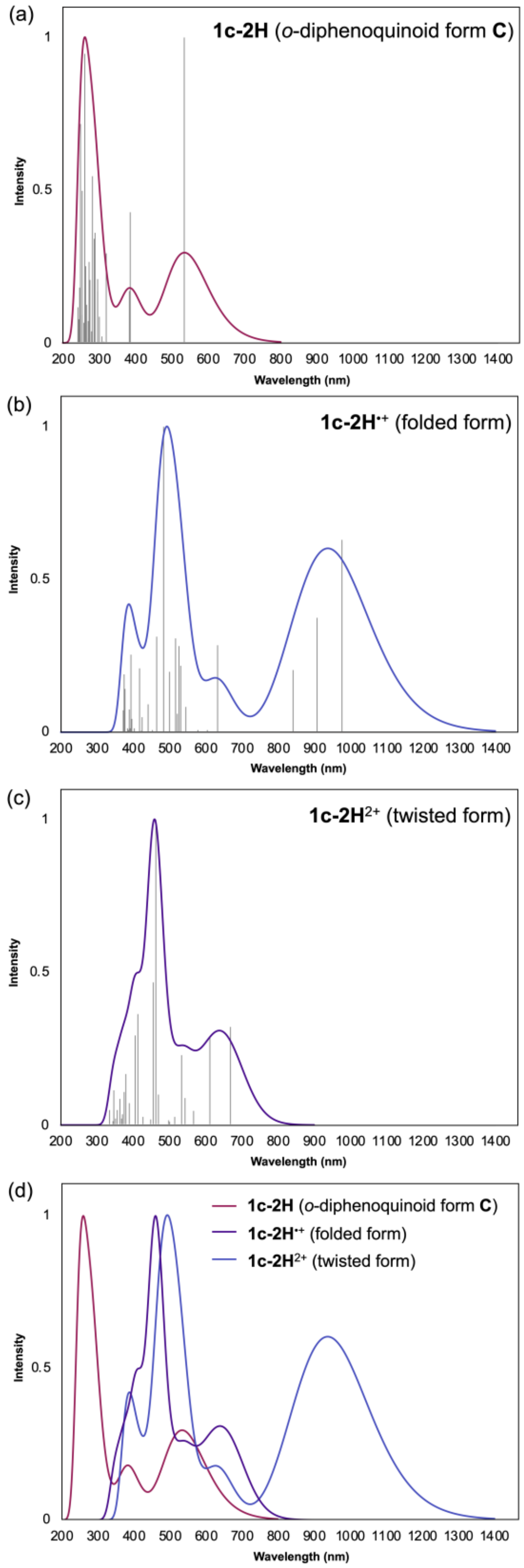

Figure S47 | Predicted electronic absorptions. Simulated UV−vis−NIR spectra by TD-DFT calculations [(U)CAM-B3LYP-D3/6-31G*] for (a) **1c-2H** (*o*-diphenoquinoid form **C**), (b) **1c-2H**$^{\bullet+}$ (folded form), (c) **1c-2H**$^{2+}$ (twisted form), and (d) three redox-states of **1c-2H**. [Ar = 4-MeO-2-MeC_6H_3]

CASSCF calculations (Figure S48)

Complete active space self-consistent field (CASSCF) is a wavefunction-based multi-reference method that explicitly considers electronic correlations within a chosen active space.[5] CASSCF accurately describes the electronic structure of systems in which static correlations are important by properly considering the multiple reference states involved. All CASSCF calculations for the singlet states of neutral donors **1a-1c** and several reference molecules were performed with ORCA 6.0.1[6] using the 6-31G* basis set. The calculation procedure is as follows: 1) Perform the structural optimizations at the CAM-B3LYP-D3/6-31G* level. 2) Calculate unrestricted natural orbitals and quasi-restricted orbitals based on the optimized structures, and examine the π-bonding orbitals distributed mainly in the parent skeletons and exomethylene moieties that can contribute to the open-shell character, which are reflected in the active space. 3) Conduct the CASSCF calculations at the 6-31G* basis set to analyze the occupation number for the LUMO (LUNO) and estimate the diradical index y_0.

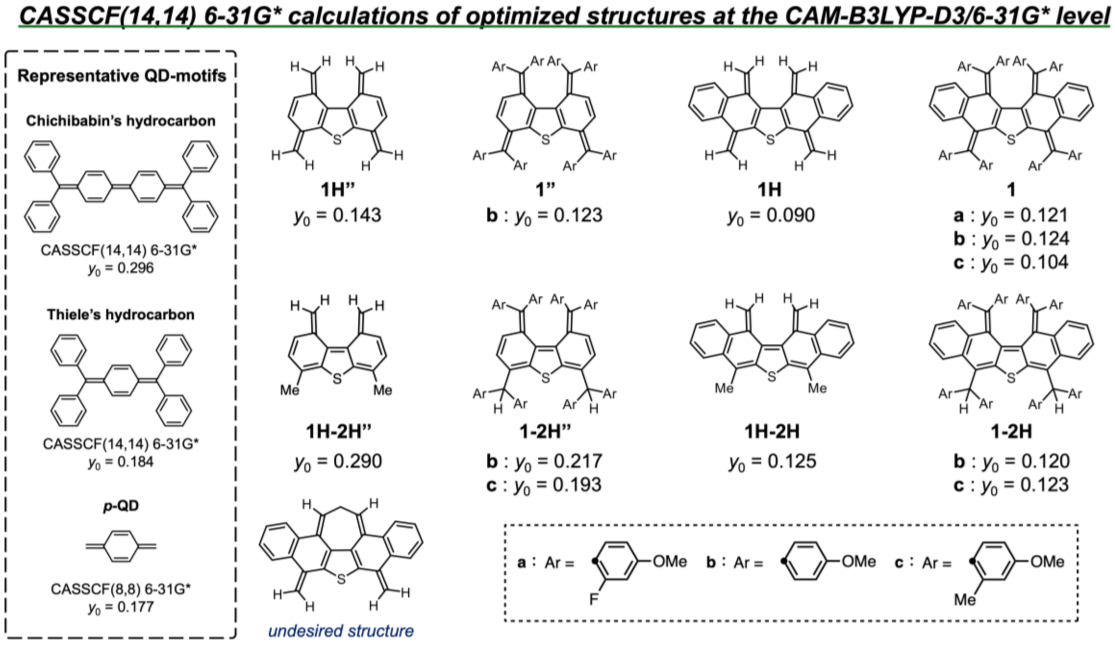

Figure S48 | Investigation of open-shell characters. Diradical index y_0 estimated by CASSCF calculations of neutral donors and their reference molecules.

(1) Neutral donors **1a-1c**

The diradical character y_0 of the experimentally isolated neutral donors **1a-1c** is about 0.104~0.124. There is no significant difference regardless of the aryl group, suggesting a closed-shell character, which is consistent with the experimental results. Comparison of reference molecules **1H** and **1H''**, which have less steric distortion, suggests that the presence or absence of a benzo-fused ring, which is crucial for the Clar's aromatic π-sextet rule discussed below, has a significant effect on the y_0 value. On the other hand, comparing **1b** with aryl groups (4-methoxyphenyl groups) and its reference molecule **1b''**, there is almost no difference in the y_0 value, suggesting that the steric hindrance and distortion caused by the introduction of aryl groups significantly reduces the diradical character. In any case, since even **1H''** (y_0=0.143) has a smaller value than Chichibabin's Hydrocarbon (y_0=0.296), which shows some contributions of open-shell character, we can conclude that, due to the slight distortion of the exomethylene moiety and the fusion of thiophene ring, the open-shell character is almost negligible in these systems.

(2) **1c-2H** with *o*-diphenoquinoid form

The diradical character y_0 of the experimentally isolated neutral *o*-diphenoquinoid **1c-2H** is about 0.123, suggesting that there is less contribution of open-shell character. Indeed, the experimental results indicate that **1c-2H** exhibits a closed-shell character. When calculated for the reference molecule **1H-2H** without aryl groups, its y_0 value was slightly higher than that of **1-2H**. This is presumably due to the reduced steric distortion (increased co-planarity) of the central skeleton. Furthermore, when **1H-2H''** and **1-2H''** without benzo-fused rings were calculated, the y_0 values were significantly larger, suggesting that the presence of benzo-fused rings in the *o*-diphenoquinoid form is an important factor for the decrease in the open-shell characters.

Therefore, these experimental and theoretical results suggest that the neutral donor **1a-1c** and **1c-2H** isolated in this study are experimentally and theoretically closed-shell species, which is common to previously reported benzofused quinodimethanes.[3,7–9] It is noted that in terms of diversification of redox-mediated molecular structures, the open-shell character of neutral species **1a-1c** does not affect the purpose and results of this paper.

ACID plots (Figure S49)

Calculations for anisotropy of the induced current density (ACID) plots[10,11] were performed. For ACID plots (isovalue: 0.02) of the three different forms (**A**, **B,** and **C**) in **1a**$^{2+}$, **1b**$^{2+}$, and **1c**$^{2+}$, the similar ring currents for each form were observed regardless of the steric effect of the *ortho* substituents. Here, we use the results of **1a**$^{2+}$ for explanation. In the *para*-quinoid form **A**, a local paramagnetic ring current was observed in the benzene ring of the central skeleton, and a global paramagnetic ring current was observed over the thienonaphthalene moiety. In the σ-bond form **B**, a global paramagnetic ring current was observed over the heteroacene core. In the *ortho*-diphenoquinoid form **C**, a local paramagnetic ring current was observed on the two benzene rings at both sides.

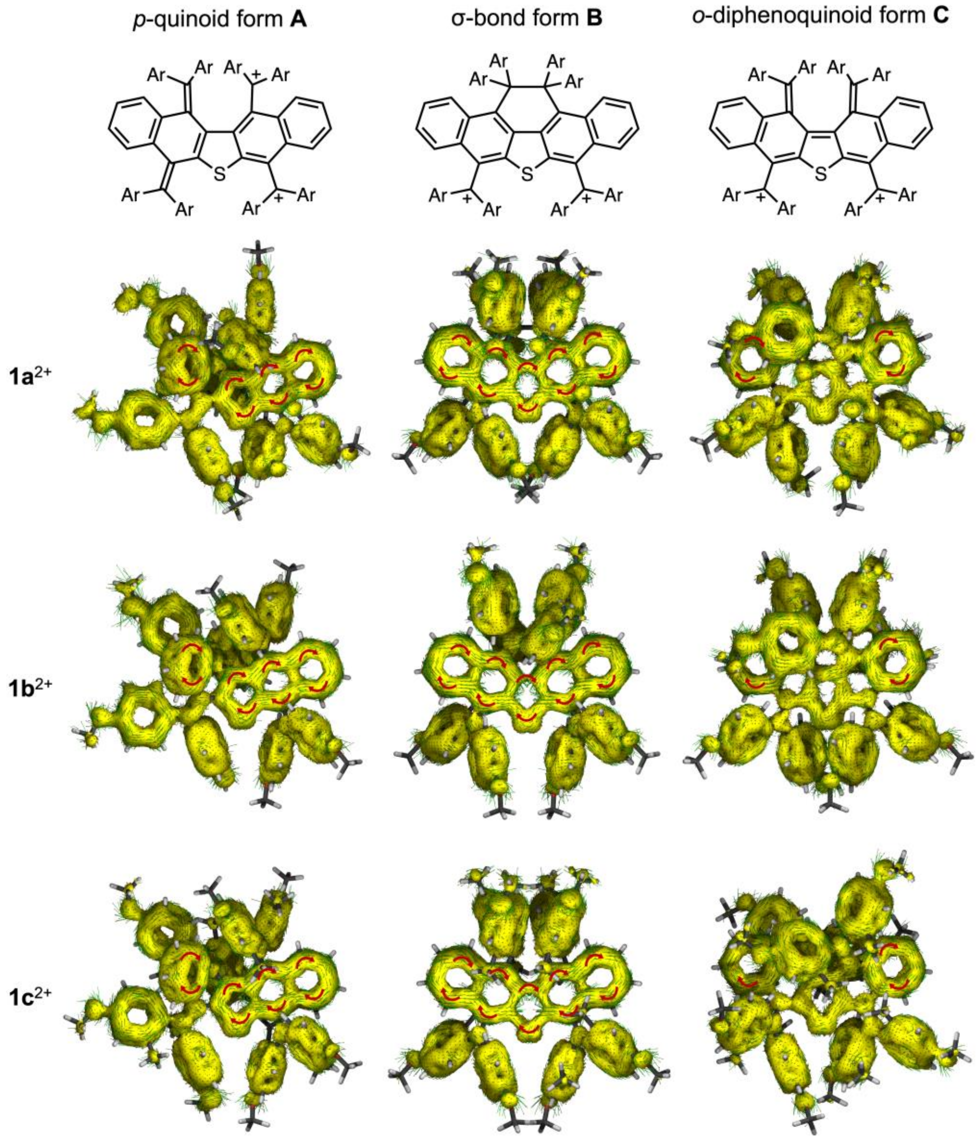

Figure S49 | Investigation of aromatic characters. ACID plots at the B3LYP/6-31G(d) level (isovalue of 0.02) of the optimized structures of neutral donors **1a-1c** by CAM-B3LYP-D3/6-31G(d) level.

NICS calculations (Figure S50)

To gain insight into aromaticity, nucleus-independent chemical shifts (NICS) calculations[12,13] were performed for dications. For NICS values of the three different forms (**A**, **B**, and **C**) in **1a**$^{2+}$, **1b**$^{2+}$, and **1c**$^{2+}$, the similar values for each form were observed regardless of the steric effect of the *ortho* substituents. In order to avoid the influence of the aryl groups, which overlap with the central skeleton, NICS(0) was adopted. In the *p*-quinoid form **A**, negative NICS values were observed for the benzene ring and thienonaphthalene skeleton, indicating that they have aromatic characters. On the other hand, the six-membered ring of the quinoid moiety showed a slightly positive NICS value, suggesting that the quinoid skeleton is non-aromatic. In the σ-bond form **B**, negative NICS values were observed for whole skeletons of heteroacene core, while strong aromaticity was observed in the two benzene rings at both sides. On the other hand, the six-membered ring containing the σ-bond showed a positive NICS value, suggesting that it is non-aromatic. In the *o*-diphenoquinoid form **C**, a negative NICS value was confirmed for the benzene rings, indicating that they are aromatic. In addition, the thiophene ring showed a slightly negative value, implying that it would be aromatic. Considering that the thienoquinoid ring is not an aromatic skeleton, this negative value would be caused by the overlap of congested aryl groups on the diarylmethylene moieties. Indeed, X-ray and theoretical analyses revealed that there are aryl groups on the thienoquinoid skeleton, by which a shielding effect could not be ignored. On the other hand, the six-membered ring of the quinoid moiety showed a slightly positive NICS value, suggesting that it is non-aromatic. As described above, aromatic and non-aromatic rings were characterized in each form.

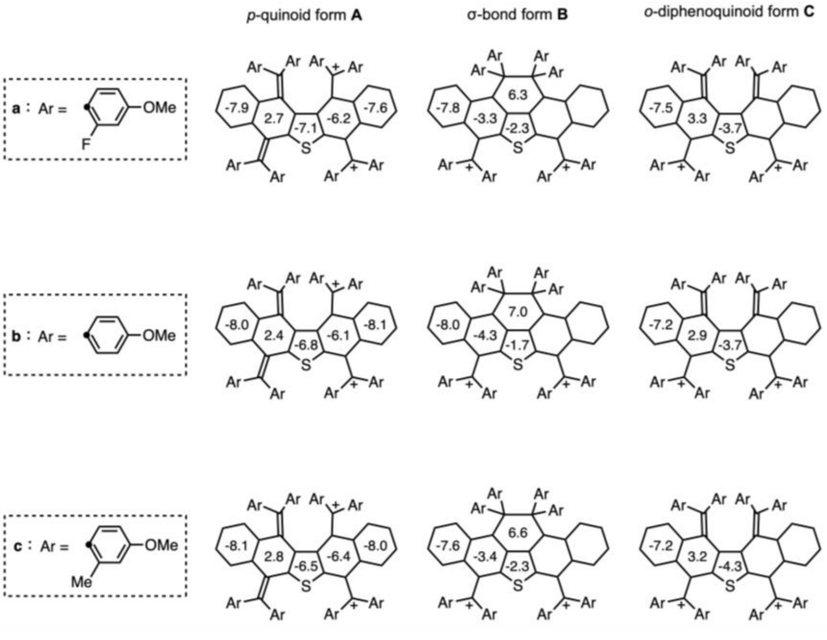

Figure S50 | Investigation of aromatic characters. Calculated NICS(0) [GIAO B3LYP/6-311+G(2d,p)//B3LYP/6-31G(d)] values (in parts per million) of the optimized structures of neutral donors **1a**-**1c** by CAM-B3LYP-D3/6-31G(d) level.

TD-DFT Excitation Energies

1a
HOMO : 344, LUMO : 345

Excitation energies and oscillator strengths:

```
 Excited State   1:      Singlet-A      3.6661 eV  338.19 nm  f=0.5153  <S**2>=0.000
     343 -> 346         0.17274
     344 -> 345         0.65525
 This state for optimization and/or second-order correction.
 Total Energy, E(TD-HF/TD-DFT) =  -4878.69073913
 Copying the excited state density for this state as the 1-particle RhoCI density.

 Excited State   2:      Singlet-A      3.9132 eV  316.84 nm  f=0.5148  <S**2>=0.000
     343 -> 345         0.25462
     344 -> 346         0.62431

 Excited State   3:      Singlet-A      4.1648 eV  297.69 nm  f=0.1074  <S**2>=0.000
     338 -> 346         0.11733
     340 -> 346         0.18933
     341 -> 345         0.14185
     343 -> 345         0.58407
     344 -> 346        -0.20571

 Excited State   4:      Singlet-A      4.2421 eV  292.27 nm  f=0.0900  <S**2>=0.000
     338 -> 345         0.14806
     340 -> 345         0.26098
     341 -> 346         0.17354
     343 -> 346         0.53673
     344 -> 347         0.15477

 Excited State   5:      Singlet-A      4.4823 eV  276.61 nm  f=0.0036  <S**2>=0.000
     335 -> 345         0.10146
     338 -> 346        -0.14868
     341 -> 345         0.53263
     342 -> 346         0.32528

 Excited State   6:      Singlet-A      4.4919 eV  276.01 nm  f=0.3111  <S**2>=0.000
     335 -> 346         0.10872
     338 -> 345        -0.18571
     340 -> 345        -0.31328
     341 -> 346         0.40920
     342 -> 345         0.27912
     343 -> 348        -0.11479
     344 -> 347         0.20049

 Excited State   7:      Singlet-A      4.5944 eV  269.86 nm  f=0.0026  <S**2>=0.000
     335 -> 345        -0.17331
     337 -> 345        -0.13064
     338 -> 346         0.20525
     339 -> 345        -0.19153
     340 -> 346         0.26788
     341 -> 345        -0.17873
     342 -> 346         0.44977

 Excited State   8:      Singlet-A      4.6028 eV  269.37 nm  f=0.0597  <S**2>=0.000
     336 -> 345         0.10564
     337 -> 346        -0.13992
     338 -> 345         0.18759
     340 -> 345         0.18591
     342 -> 345         0.50687
     343 -> 346        -0.17291
     344 -> 345         0.11820

 Excited State   9:      Singlet-A      4.6761 eV  265.14 nm  f=0.0318  <S**2>=0.000
     338 -> 345         0.10238
     341 -> 346        -0.25359
     343 -> 346        -0.12043
     344 -> 347         0.54568
```

344 -> 349 -0.14910

Excited State 10: Singlet-A 4.7684 eV 260.01 nm f=0.0522 <S**2>=0.000
338 -> 345 -0.35925
339 -> 346 0.26731
340 -> 345 0.40871
340 -> 347 -0.11085
343 -> 346 -0.12112
344 -> 347 0.14232

Excited State 11: Singlet-A 4.8018 eV 258.20 nm f=0.0169 <S**2>=0.000
339 -> 345 0.51262
339 -> 347 -0.10603
340 -> 346 0.26337
344 -> 348 -0.18477
344 -> 350 -0.15946

Excited State 12: Singlet-A 4.8260 eV 256.91 nm f=0.0054 <S**2>=0.000
332 -> 345 -0.10911
336 -> 346 -0.14426
337 -> 345 0.20056
338 -> 346 0.15941
339 -> 345 -0.22955
343 -> 347 0.30412
343 -> 349 -0.10200
344 -> 348 -0.22369
344 -> 350 -0.18724

Excited State 13: Singlet-A 4.9575 eV 250.09 nm f=0.0257 <S**2>=0.000
330 -> 345 0.10416
336 -> 345 -0.21415
336 -> 347 -0.11275
337 -> 346 0.32879
340 -> 345 0.11099
340 -> 347 0.10150
341 -> 352 0.10830
342 -> 345 0.15024
342 -> 351 0.16991
343 -> 350 -0.12397
344 -> 354 0.10697

Excited State 14: Singlet-A 5.0166 eV 247.15 nm f=0.1066 <S**2>=0.000
336 -> 346 -0.23432
337 -> 345 0.28212
337 -> 347 0.12656
341 -> 354 0.11204
342 -> 346 0.12800
342 -> 348 -0.10928
342 -> 350 -0.10391
343 -> 351 0.11619
343 -> 354 -0.10785
344 -> 348 0.15003
344 -> 352 0.20277
344 -> 353 -0.11522

Excited State 15: Singlet-A 5.0355 eV 246.22 nm f=0.0025 <S**2>=0.000
332 -> 345 -0.13199
336 -> 346 -0.15374
337 -> 345 0.14804
338 -> 346 0.11713
340 -> 346 0.10882
341 -> 345 0.11141
341 -> 347 -0.17132
342 -> 352 -0.12730
342 -> 353 -0.11634
343 -> 347 -0.20479
343 -> 351 -0.18371
344 -> 348 0.21013
344 -> 352 -0.13119

Excited State 16: Singlet-A 5.0387 eV 246.06 nm f=0.0430 <S**2>=0.000
330 -> 345 0.10816
334 -> 345 0.13698

337 -> 346 -0.16328
338 -> 345 -0.11706
341 -> 353 -0.12280
342 -> 354 -0.11817
343 -> 352 -0.13573
343 -> 353 0.17923
344 -> 351 -0.21893
344 -> 354 0.23746

Excited State 17: Singlet-A 5.0991 eV 243.15 nm f=0.0004 <S**2>=0.000
327 -> 347 -0.10538
336 -> 345 -0.14461
336 -> 347 -0.15157
337 -> 346 0.27906
337 -> 348 -0.10194
341 -> 352 -0.13270
342 -> 345 0.10694
342 -> 351 -0.15812
342 -> 359 0.11883
343 -> 348 0.13246
343 -> 352 -0.15241
344 -> 349 0.10751
344 -> 351 -0.18542

Excited State 18: Singlet-A 5.1003 eV 243.09 nm f=0.0091 <S**2>=0.000
326 -> 345 -0.11665
327 -> 346 0.10004
330 -> 346 0.10012
341 -> 351 0.13711
342 -> 352 0.22540
343 -> 347 -0.11058
343 -> 354 0.20294
344 -> 352 0.11044
344 -> 353 0.30382

Excited State 19: Singlet-A 5.1602 eV 240.27 nm f=0.0251 <S**2>=0.000
335 -> 345 0.13855
336 -> 346 0.23589
336 -> 348 -0.17372
337 -> 347 -0.17071
337 -> 349 -0.10087
338 -> 346 0.16388
340 -> 346 0.11813
341 -> 347 -0.16643
343 -> 345 -0.11158
343 -> 347 -0.11100
344 -> 352 0.12891

Excited State 20: Singlet-A 5.1811 eV 239.30 nm f=0.0304 <S**2>=0.000
335 -> 346 0.13187
338 -> 345 0.22511
338 -> 347 -0.12660
341 -> 346 0.15160
342 -> 345 -0.18046
343 -> 350 0.10352
343 -> 353 0.16193
344 -> 351 0.12469
344 -> 354 0.17644
344 -> 355 0.12586

Excited State 21: Singlet-A 5.1966 eV 238.59 nm f=0.0479 <S**2>=0.000
339 -> 358 0.10131
341 -> 347 0.18953
343 -> 347 0.32725
343 -> 349 -0.10010
344 -> 348 0.37352
344 -> 350 0.15539

Excited State 22: Singlet-A 5.2248 eV 237.30 nm f=0.0181 <S**2>=0.000
340 -> 346 0.10897
342 -> 346 -0.11197
342 -> 348 -0.13822
343 -> 347 0.12111

343 -> 349 0.23033
344 -> 348 -0.21035
344 -> 350 0.42068
344 -> 353 0.13049

Excited State 23: Singlet-A 5.2327 eV 236.94 nm f=0.0179 <S**2>=0.000
338 -> 345 -0.17278
341 -> 348 -0.12110
342 -> 347 0.11146
342 -> 349 0.10565
343 -> 348 -0.19701
343 -> 350 0.19326
344 -> 347 0.10822
344 -> 349 0.39232
344 -> 361 0.15965

Excited State 24: Singlet-A 5.2826 eV 234.70 nm f=0.0035 <S**2>=0.000
334 -> 346 -0.20188
335 -> 345 0.25997
336 -> 348 -0.13952
337 -> 345 0.13029
337 -> 349 -0.14248
338 -> 357 0.10474
339 -> 358 -0.18959
340 -> 357 -0.16542
341 -> 345 -0.14197
341 -> 347 0.13441
342 -> 346 0.12243
343 -> 358 -0.10748

Excited State 25: Singlet-A 5.2886 eV 234.44 nm f=0.0162 <S**2>=0.000
334 -> 345 0.15531
335 -> 346 -0.15715
336 -> 349 -0.14850
337 -> 348 -0.12031
338 -> 358 -0.15469
339 -> 357 0.24887
340 -> 358 0.23771
342 -> 358 -0.10918
343 -> 357 0.17483
344 -> 349 -0.11624
344 -> 351 0.15511

Excited State 26: Singlet-A 5.3125 eV 233.38 nm f=0.0659 <S**2>=0.000
334 -> 345 -0.15610
335 -> 346 0.18347
336 -> 349 0.16238
337 -> 346 0.11283
337 -> 348 0.10698
337 -> 350 -0.12330
338 -> 358 -0.13144
339 -> 357 0.20880
340 -> 358 0.20279
343 -> 357 0.13338
344 -> 351 -0.14734

Excited State 27: Singlet-A 5.3141 eV 233.31 nm f=0.0002 <S**2>=0.000
334 -> 346 -0.11230
335 -> 345 0.17536
336 -> 348 -0.10483
337 -> 345 0.12556
337 -> 349 -0.12382
338 -> 357 -0.15509
339 -> 358 0.27583
340 -> 357 0.23782
342 -> 357 -0.10276
343 -> 358 0.19301
344 -> 348 -0.12575

Excited State 28: Singlet-A 5.3449 eV 231.97 nm f=0.0470 <S**2>=0.000
334 -> 345 0.11686
336 -> 349 0.12414
337 -> 348 0.14966

340 -> 347 0.15868
341 -> 346 0.14673
342 -> 347 0.22094
343 -> 346 -0.10643
343 -> 348 -0.21224
344 -> 349 0.13489
344 -> 351 0.26285
344 -> 361 -0.15899

Excited State 29: Singlet-A 5.3948 eV 229.82 nm f=0.0005 <S**2>=0.000
332 -> 345 0.10568
334 -> 346 -0.17006
335 -> 345 0.35377
336 -> 348 0.13103
336 -> 350 -0.12347
337 -> 345 -0.12909
337 -> 349 0.17162
338 -> 346 0.12967
340 -> 346 0.17329
342 -> 346 -0.10712
343 -> 349 -0.10473
344 -> 348 0.11429
344 -> 350 -0.13177

Excited State 30: Singlet-A 5.4372 eV 228.03 nm f=0.0298 <S**2>=0.000
334 -> 345 0.29125
335 -> 346 -0.17048
339 -> 346 -0.19029
341 -> 346 0.25555
342 -> 345 -0.15807
343 -> 346 -0.20302
344 -> 351 -0.20682
344 -> 361 0.15452

1b
HOMO : 312, LUMO : 313

Excitation energies and oscillator strengths:

Excited State 1: Singlet-A 3.3553 eV 369.52 nm f=0.6237 <S**2>=0.000
310 -> 313 -0.11258
311 -> 314 0.19496
312 -> 313 0.64455
This state for optimization and/or second-order correction.
Total Energy, E(TD-HF/TD-DFT) = -4084.96150153
Copying the excited state density for this state as the 1-particle RhoCI density.

Excited State 2: Singlet-A 3.6392 eV 340.69 nm f=0.7375 <S**2>=0.000
311 -> 313 0.42098
312 -> 314 0.53369

Excited State 3: Singlet-A 3.8640 eV 320.87 nm f=0.0239 <S**2>=0.000
306 -> 314 -0.10380
309 -> 313 -0.13281
311 -> 313 0.50724
311 -> 314 -0.13152
312 -> 314 -0.37074

Excited State 4: Singlet-A 3.9640 eV 312.78 nm f=0.1078 <S**2>=0.000
306 -> 313 -0.13985
309 -> 314 -0.13629
310 -> 313 0.12091
311 -> 313 0.10845
311 -> 314 0.57798
312 -> 313 -0.10544
312 -> 314 -0.10533
312 -> 315 0.12707

Excited State 5: Singlet-A 4.2159 eV 294.08 nm f=0.0209 <S**2>=0.000

309 -> 313 0.47326
310 -> 313 0.17578
310 -> 314 -0.38893

Excited State 6: Singlet-A 4.2315 eV 293.00 nm f=0.1234 <S**2>=0.000
309 -> 313 -0.10886
309 -> 314 -0.12106
310 -> 313 0.58817
310 -> 314 0.14132
311 -> 314 -0.14232
312 -> 313 0.15557

Excited State 7: Singlet-A 4.3833 eV 282.85 nm f=0.0744 <S**2>=0.000
302 -> 314 -0.10013
304 -> 313 0.11300
305 -> 313 -0.17421
306 -> 313 0.33817
306 -> 314 -0.12996
307 -> 313 0.11663
309 -> 313 0.19168
309 -> 314 -0.32880
310 -> 314 0.27406

Excited State 8: Singlet-A 4.4223 eV 280.36 nm f=0.0279 <S**2>=0.000
302 -> 313 0.13889
305 -> 313 0.19386
305 -> 314 0.17564
306 -> 313 -0.14805
306 -> 314 -0.16992
307 -> 314 -0.13119
309 -> 313 0.24939
309 -> 314 0.27119
310 -> 314 0.34816

Excited State 9: Singlet-A 4.5398 eV 273.10 nm f=0.1190 <S**2>=0.000
308 -> 314 -0.11418
309 -> 314 0.10796
311 -> 314 -0.12167
311 -> 316 0.14929
312 -> 315 0.59691

Excited State 10: Singlet-A 4.6171 eV 268.54 nm f=0.0073 <S**2>=0.000
303 -> 313 -0.10634
308 -> 313 -0.16538
311 -> 315 0.41085
312 -> 316 0.37263
312 -> 317 0.10111

Excited State 11: Singlet-A 4.7743 eV 259.69 nm f=0.0154 <S**2>=0.000
299 -> 313 -0.12831
300 -> 314 0.10985
304 -> 313 -0.11215
305 -> 313 0.18471
305 -> 314 0.11731
306 -> 313 0.17196
307 -> 313 0.15706
308 -> 314 -0.17620
310 -> 313 0.11596
311 -> 316 0.14541
312 -> 315 -0.17242
312 -> 318 0.24710

Excited State 12: Singlet-A 4.8129 eV 257.61 nm f=0.0050 <S**2>=0.000
305 -> 314 -0.12187
309 -> 315 -0.15320
309 -> 318 -0.12339
311 -> 315 0.26442
311 -> 318 -0.23034
312 -> 317 0.27460

Excited State 13: Singlet-A 4.8252 eV 256.95 nm f=0.0265 <S**2>=0.000
305 -> 313 0.32712
306 -> 313 0.21992

306 -> 314 0.17826
307 -> 314 -0.13365
308 -> 313 0.16525
310 -> 314 -0.12253
311 -> 315 0.24176
312 -> 317 -0.12220
312 -> 318 -0.10030

Excited State 14: Singlet-A 4.8440 eV 255.95 nm f=0.0406 <S**2>=0.000
304 -> 313 0.10889
305 -> 313 -0.12018
305 -> 314 -0.16473
306 -> 313 -0.21286
307 -> 313 0.20085
307 -> 314 -0.15746
308 -> 313 0.22891
308 -> 314 -0.20596
311 -> 317 0.11159
312 -> 316 0.16049

Excited State 15: Singlet-A 4.8739 eV 254.38 nm f=0.0451 <S**2>=0.000
305 -> 313 -0.15718
307 -> 314 -0.13921
308 -> 313 0.19566
308 -> 314 0.21606
311 -> 317 -0.10995
311 -> 324 0.10643
312 -> 316 0.11976
312 -> 318 0.27845

Excited State 16: Singlet-A 4.8970 eV 253.18 nm f=0.0247 <S**2>=0.000
298 -> 315 -0.10230
304 -> 313 -0.24619
307 -> 313 0.13494
308 -> 314 -0.20271
311 -> 315 -0.10355
311 -> 316 -0.11142
311 -> 324 0.12170
312 -> 316 0.12898
312 -> 319 0.25356

Excited State 17: Singlet-A 4.9305 eV 251.46 nm f=0.0285 <S**2>=0.000
304 -> 313 0.26155
306 -> 313 -0.13662
306 -> 314 -0.10767
308 -> 314 -0.18087
310 -> 325 0.10967
311 -> 325 -0.10038
312 -> 316 -0.20898
312 -> 318 0.10286
312 -> 319 0.11254
312 -> 322 -0.11611
312 -> 323 -0.13466

Excited State 18: Singlet-A 4.9467 eV 250.64 nm f=0.0116 <S**2>=0.000
304 -> 313 0.13618
310 -> 323 0.16814
310 -> 324 0.13432
311 -> 315 0.15016
311 -> 319 0.22626
312 -> 316 -0.20637
312 -> 323 0.16215
312 -> 324 0.19353

Excited State 19: Singlet-A 4.9921 eV 248.36 nm f=0.0859 <S**2>=0.000
302 -> 313 -0.10133
304 -> 313 0.31217
304 -> 314 -0.11705
304 -> 316 0.12018
305 -> 313 0.14173
307 -> 314 0.15378
311 -> 321 -0.12168
312 -> 316 0.21722

312 -> 321 0.16399
312 -> 322 0.10367

Excited State 20: Singlet-A 5.0017 eV 247.89 nm f=0.0213 <S**2>=0.000
302 -> 313 -0.10888
304 -> 313 0.21136
304 -> 314 -0.10066
305 -> 313 0.19039
307 -> 314 -0.15380
309 -> 315 0.14589
311 -> 315 -0.18149
312 -> 317 0.36746
312 -> 319 -0.10652
312 -> 320 0.12032

Excited State 21: Singlet-A 5.0206 eV 246.95 nm f=0.0295 <S**2>=0.000
304 -> 313 0.10780
305 -> 313 -0.14088
306 -> 313 0.20278
306 -> 314 -0.11731
307 -> 314 -0.12864
308 -> 314 0.11573
309 -> 313 -0.10064
309 -> 314 0.20351
310 -> 313 0.11481
311 -> 316 -0.11611
312 -> 318 -0.10805
312 -> 319 0.24705
312 -> 321 0.10945
312 -> 322 -0.10957

Excited State 22: Singlet-A 5.0567 eV 245.19 nm f=0.0123 <S**2>=0.000
302 -> 313 0.25489
303 -> 313 0.14958
307 -> 314 0.22253
307 -> 316 0.10151
308 -> 315 -0.12106
312 -> 317 0.19650
312 -> 320 0.10521
312 -> 322 -0.13611

Excited State 23: Singlet-A 5.0795 eV 244.09 nm f=0.0534 <S**2>=0.000
302 -> 313 -0.12762
302 -> 314 0.11361
309 -> 314 0.13699
311 -> 316 0.21938
311 -> 317 0.15122
311 -> 322 0.11838
311 -> 327 -0.10516
312 -> 321 -0.13603
312 -> 323 0.10760
312 -> 328 -0.14578

Excited State 24: Singlet-A 5.1318 eV 241.60 nm f=0.0309 <S**2>=0.000
301 -> 313 -0.10335
305 -> 317 -0.12089
306 -> 313 -0.10440
306 -> 314 0.10340
309 -> 314 -0.12999
311 -> 317 0.21297
311 -> 320 0.10653
312 -> 325 -0.18497
312 -> 329 0.14991

Excited State 25: Singlet-A 5.1513 eV 240.68 nm f=0.0060 <S**2>=0.000
301 -> 314 0.20509
302 -> 313 -0.18353
303 -> 313 -0.13848
305 -> 314 -0.12120
306 -> 314 0.20540
309 -> 313 0.16545
309 -> 314 0.11577
310 -> 314 0.10987

```
     311 -> 317       -0.11440
     312 -> 320        0.12676
     312 -> 329       -0.11566

 Excited State  26:      Singlet-A      5.1966 eV  238.59 nm  f=0.0970  <S**2>=0.000
     301 -> 313        0.16039
     302 -> 314       -0.10177
     303 -> 314       -0.23118
     307 -> 315       -0.10032
     307 -> 319       -0.12972
     307 -> 321        0.12004
     307 -> 322       -0.10973
     308 -> 320       -0.16698
     310 -> 315        0.29171
     311 -> 316       -0.14682
     311 -> 317       -0.10783
     312 -> 323        0.10792

 Excited State  27:      Singlet-A      5.2087 eV  238.03 nm  f=0.2484  <S**2>=0.000
     307 -> 321        0.10697
     308 -> 320       -0.10925
     310 -> 315       -0.21861
     311 -> 323        0.16928
     312 -> 321        0.25383
     312 -> 322        0.12739
     312 -> 323       -0.20511
     312 -> 324        0.14359

 Excited State  28:      Singlet-A      5.2169 eV  237.66 nm  f=0.0738  <S**2>=0.000
     302 -> 313       -0.15403
     302 -> 314        0.14090
     305 -> 314        0.10280
     306 -> 315       -0.10942
     307 -> 320       -0.10675
     308 -> 313        0.10167
     310 -> 315        0.33672
     312 -> 321        0.10995
     312 -> 322        0.10635
     312 -> 323       -0.19576

 Excited State  29:      Singlet-A      5.2244 eV  237.32 nm  f=0.1009  <S**2>=0.000
     302 -> 313        0.25558
     304 -> 314        0.11596
     307 -> 320        0.19698
     308 -> 313       -0.15801
     308 -> 319        0.11416
     308 -> 322        0.10012
     310 -> 315        0.26804
     312 -> 322        0.10287

 Excited State  30:      Singlet-A      5.2654 eV  235.47 nm  f=0.0081  <S**2>=0.000
     303 -> 313       -0.12242
     304 -> 317        0.11669
     305 -> 318        0.11154
     306 -> 316        0.12225
     308 -> 313       -0.15505
     311 -> 316        0.12591
     311 -> 319        0.15826
     312 -> 316        0.17818
     312 -> 320       -0.11982
     312 -> 324        0.13198
     312 -> 326        0.18339
```

1c

HOMO : 344, LUMO : 345

Excitation energies and oscillator strengths:

```
 Excited State   1:      Singlet-A      3.4796 eV  356.32 nm  f=0.4826  <S**2>=0.000
     342 -> 345       -0.12457
     343 -> 346        0.17336
```

344 -> 345 0.64835
This state for optimization and/or second-order correction.
Total Energy, E(TD-HF/TD-DFT) = -4399.32223879
Copying the excited state density for this state as the 1-particle RhoCI density.

Excited State 2: Singlet-A 3.7698 eV 328.89 nm f=0.7530 <S**2>=0.000
343 -> 345 0.51958
344 -> 346 0.44029

Excited State 3: Singlet-A 3.9652 eV 312.68 nm f=0.0146 <S**2>=0.000
339 -> 345 -0.12881
340 -> 346 0.13261
341 -> 345 -0.13015
343 -> 345 -0.41179
344 -> 346 0.48514

Excited State 4: Singlet-A 4.0776 eV 304.06 nm f=0.1296 <S**2>=0.000
340 -> 345 -0.19259
341 -> 346 0.14216
342 -> 345 0.20197
343 -> 346 0.55933
344 -> 347 -0.14000

Excited State 5: Singlet-A 4.2684 eV 290.47 nm f=0.0728 <S**2>=0.000
341 -> 346 0.14061
342 -> 345 0.58592
343 -> 346 -0.21799
344 -> 345 0.17345

Excited State 6: Singlet-A 4.2849 eV 289.35 nm f=0.0015 <S**2>=0.000
341 -> 345 0.48262
342 -> 346 0.40838

Excited State 7: Singlet-A 4.4445 eV 278.96 nm f=0.0485 <S**2>=0.000
333 -> 345 -0.12065
334 -> 346 0.10096
335 -> 345 -0.17864
339 -> 346 0.10856
340 -> 345 0.46047
341 -> 346 0.35439
343 -> 346 0.11973
343 -> 348 -0.10870

Excited State 8: Singlet-A 4.4884 eV 276.23 nm f=0.0000 <S**2>=0.000
332 -> 345 -0.10083
333 -> 346 0.11529
334 -> 345 -0.15452
335 -> 346 0.15638
336 -> 345 0.15842
337 -> 345 -0.11537
339 -> 345 -0.19716
340 -> 346 -0.25998
341 -> 345 -0.25545
342 -> 346 0.40917

Excited State 9: Singlet-A 4.6746 eV 265.23 nm f=0.0567 <S**2>=0.000
335 -> 345 -0.15862
339 -> 346 0.16620
340 -> 345 -0.11886
341 -> 346 0.10072
343 -> 346 0.10222
343 -> 348 0.11845
344 -> 347 0.53376

Excited State 10: Singlet-A 4.6913 eV 264.29 nm f=0.0338 <S**2>=0.000
335 -> 346 -0.14662
337 -> 345 0.24249
339 -> 345 0.51851
340 -> 346 -0.16292
341 -> 345 -0.20409
342 -> 346 0.15648

Excited State 11: Singlet-A 4.7565 eV 260.66 nm f=0.0008 <S**2>=0.000

334 -> 345 -0.10095
335 -> 346 -0.13927
336 -> 345 0.21610
337 -> 345 -0.16985
338 -> 346 -0.15115
339 -> 345 0.12296
340 -> 346 0.10331
343 -> 347 0.34174
344 -> 348 0.24637
344 -> 349 0.12423

Excited State 12: Singlet-A 4.7580 eV 260.58 nm f=0.0650 <S**2>=0.000
335 -> 345 0.43552
337 -> 346 -0.14136
339 -> 346 -0.24494
340 -> 345 0.29705
344 -> 347 0.24068

Excited State 13: Singlet-A 4.8681 eV 254.69 nm f=0.0048 <S**2>=0.000
329 -> 345 0.12171
333 -> 345 0.20343
334 -> 346 -0.14870
336 -> 346 0.18431
337 -> 346 -0.13179
342 -> 345 -0.13080
342 -> 350 -0.18049
343 -> 349 0.17573
343 -> 355 -0.11464
344 -> 347 -0.11855
344 -> 352 -0.17004
344 -> 357 -0.11182

Excited State 14: Singlet-A 4.8990 eV 253.08 nm f=0.0018 <S**2>=0.000
333 -> 346 -0.11901
334 -> 345 0.17027
336 -> 345 -0.20317
338 -> 346 -0.11894
341 -> 347 0.10610
341 -> 350 0.10410
342 -> 349 -0.11406
342 -> 351 -0.10355
342 -> 355 0.19804
343 -> 347 0.21582
343 -> 350 0.11292
343 -> 352 0.13341
344 -> 349 -0.14206
344 -> 355 0.17052

Excited State 15: Singlet-A 4.9473 eV 250.61 nm f=0.0002 <S**2>=0.000
328 -> 345 0.15693
341 -> 352 0.10252
342 -> 354 -0.12209
343 -> 347 -0.17003
343 -> 350 0.21587
344 -> 348 -0.18403
344 -> 351 -0.13071
344 -> 354 0.21811

Excited State 16: Singlet-A 4.9474 eV 250.60 nm f=0.0019 <S**2>=0.000
337 -> 346 -0.19738
338 -> 345 -0.18034
338 -> 347 0.10423
341 -> 354 -0.11074
342 -> 352 0.13290
343 -> 351 -0.12618
343 -> 355 0.12060
344 -> 347 -0.14411
344 -> 350 0.26467

Excited State 17: Singlet-A 4.9695 eV 249.49 nm f=0.0103 <S**2>=0.000
325 -> 345 0.14058
337 -> 346 0.24845
338 -> 345 0.25378

338 -> 347 -0.12682
339 -> 346 -0.13384
341 -> 351 0.10616
341 -> 354 -0.12628
343 -> 354 0.14423
344 -> 347 0.12061
344 -> 350 0.18742
344 -> 352 -0.11574
344 -> 356 0.12304

Excited State 18: Singlet-A 4.9866 eV 248.64 nm f=0.0558 <S**2>=0.000
324 -> 345 0.10586
334 -> 345 0.12007
336 -> 345 -0.12877
337 -> 345 0.23913
337 -> 347 -0.14539
338 -> 346 0.23804
339 -> 345 -0.12628
342 -> 346 0.14910
343 -> 347 0.27409
344 -> 348 0.10428
344 -> 349 0.15254
344 -> 354 0.12211

Excited State 19: Singlet-A 5.0569 eV 245.18 nm f=0.0364 <S**2>=0.000
331 -> 345 0.14255
332 -> 345 -0.15753
333 -> 346 0.10595
334 -> 345 -0.12715
336 -> 345 0.17316
337 -> 345 0.16400
338 -> 346 0.26188
341 -> 345 0.11127
343 -> 352 0.11474
344 -> 348 0.13159
344 -> 349 -0.19299

Excited State 20: Singlet-A 5.0904 eV 243.56 nm f=0.0325 <S**2>=0.000
324 -> 345 0.10039
325 -> 346 0.10100
330 -> 346 0.10355
332 -> 345 -0.17370
333 -> 346 0.21507
337 -> 345 -0.10589
338 -> 351 -0.10093
341 -> 347 0.17378
342 -> 355 -0.10420
343 -> 347 0.14257
343 -> 353 -0.11158
344 -> 349 -0.12947
344 -> 351 -0.12672
344 -> 355 -0.12071

Excited State 21: Singlet-A 5.0941 eV 243.39 nm f=0.0736 <S**2>=0.000
329 -> 345 -0.10004
332 -> 346 -0.14741
333 -> 345 0.31840
335 -> 345 -0.13701
336 -> 346 0.23782
339 -> 346 -0.10145
339 -> 349 0.10444
341 -> 346 0.11790
343 -> 348 -0.15650
343 -> 354 -0.10525
344 -> 350 0.10392
344 -> 353 -0.13737

Excited State 22: Singlet-A 5.0996 eV 243.13 nm f=0.0494 <S**2>=0.000
325 -> 345 -0.11243
331 -> 358 0.10994
332 -> 346 0.16233
333 -> 345 -0.18290
335 -> 345 0.12415

335 -> 353 0.12043
336 -> 346 -0.16640
339 -> 348 -0.13277
339 -> 349 0.10254
339 -> 351 -0.12213
340 -> 345 -0.15199
340 -> 353 0.12070
343 -> 349 0.18637
344 -> 356 -0.10970

Excited State 23: Singlet-A 5.1031 eV 242.96 nm f=0.0234 <S**2>=0.000
332 -> 345 -0.13626
335 -> 349 -0.10053
336 -> 345 0.23402
338 -> 346 0.15956
340 -> 346 0.12742
342 -> 348 0.10062
343 -> 350 0.11435
343 -> 353 -0.12794
343 -> 356 -0.11388
344 -> 348 -0.23042
344 -> 354 -0.10326

Excited State 24: Singlet-A 5.1137 eV 242.45 nm f=0.0172 <S**2>=0.000
330 -> 345 -0.14408
336 -> 346 0.14445
337 -> 346 0.11815
338 -> 347 -0.11947
338 -> 357 0.11382
341 -> 346 0.10033
342 -> 361 0.13371
343 -> 351 0.10822
343 -> 355 0.19812
344 -> 353 0.18177
344 -> 356 -0.10197
344 -> 361 0.13884

Excited State 25: Singlet-A 5.1417 eV 241.13 nm f=0.0002 <S**2>=0.000
332 -> 345 -0.11766
333 -> 346 0.19172
337 -> 345 -0.10335
338 -> 351 0.10940
339 -> 345 0.11240
340 -> 346 -0.13937
341 -> 357 0.10713
342 -> 346 -0.10308
343 -> 352 -0.13195
343 -> 361 0.12219
344 -> 349 0.27156
344 -> 355 0.17845

Excited State 26: Singlet-A 5.1520 eV 240.65 nm f=0.0014 <S**2>=0.000
332 -> 346 0.14423
335 -> 345 0.23732
340 -> 345 -0.12282
341 -> 346 0.20082
341 -> 351 -0.10465
342 -> 345 -0.10589
343 -> 349 -0.19997
344 -> 350 0.21291
344 -> 352 0.17736
344 -> 357 0.11911
344 -> 361 -0.14508

Excited State 27: Singlet-A 5.1695 eV 239.84 nm f=0.0224 <S**2>=0.000
337 -> 345 0.16047
338 -> 351 -0.12162
341 -> 347 -0.10260
343 -> 347 -0.21935
343 -> 357 -0.20042
344 -> 348 0.30627
344 -> 349 0.11877
344 -> 351 -0.16237

344 -> 354 -0.10311

Excited State 28: Singlet-A 5.1718 eV 239.73 nm f=0.0766 <S**2>=0.000
336 -> 346 -0.10972
337 -> 346 -0.11433
337 -> 351 0.12919
338 -> 345 -0.19857
338 -> 352 -0.12808
338 -> 357 0.11286
341 -> 349 0.10130
342 -> 352 -0.11218
343 -> 348 -0.14267
343 -> 351 0.19587
344 -> 350 0.15396
344 -> 357 0.26414
344 -> 361 -0.11396

Excited State 29: Singlet-A 5.2404 eV 236.59 nm f=0.0250 <S**2>=0.000
332 -> 345 0.30221
334 -> 345 0.33221
335 -> 346 0.10360
336 -> 345 0.31129
336 -> 347 -0.11359
338 -> 346 0.12219
340 -> 346 -0.10909
343 -> 347 -0.14194

Excited State 30: Singlet-A 5.2607 eV 235.68 nm f=0.0171 <S**2>=0.000
325 -> 345 -0.12161
329 -> 345 0.11191
330 -> 345 -0.14509
332 -> 346 0.15919
333 -> 345 -0.16251
336 -> 346 0.27842
341 -> 346 -0.24018
342 -> 347 0.15796
342 -> 360 -0.10458
343 -> 348 -0.10444
343 -> 355 -0.11316
344 -> 353 -0.20732
344 -> 356 0.13226

1b-2H (σ-bond form **B**)
HOMO : 313, LUMO : 314

Excitation energies and oscillator strengths:

Excited State 1: Singlet-A 3.5629 eV 347.98 nm f=0.1179 <S**2>=0.000
303 -> 315 0.10047
313 -> 314 0.66892
This state for optimization and/or second-order correction.
Total Energy, E(TD-HF/TD-DFT) = -4086.19438154
Copying the excited state density for this state as the 1-particle RhoCI density.

Excited State 2: Singlet-A 3.7434 eV 331.21 nm f=0.0740 <S**2>=0.000
310 -> 314 -0.10031
312 -> 314 0.64196
313 -> 315 -0.22681

Excited State 3: Singlet-A 4.3401 eV 285.67 nm f=0.2147 <S**2>=0.000
299 -> 315 -0.10556
303 -> 314 0.41781
305 -> 314 -0.13762
310 -> 314 -0.21591
311 -> 314 0.12796
312 -> 316 -0.23129
313 -> 315 0.25609
313 -> 319 0.12080

Excited State 4: Singlet-A 4.3572 eV 284.55 nm f=0.2374 <S**2>=0.000
299 -> 314 -0.10353

302 -> 314 0.11041
310 -> 315 -0.12518
312 -> 315 0.54957
313 -> 314 -0.14128
313 -> 316 0.27157
313 -> 317 -0.10568

Excited State 5: Singlet-A 4.4154 eV 280.80 nm f=0.4371 <S**2>=0.000
310 -> 314 0.28673
312 -> 314 0.24676
313 -> 315 0.53437

Excited State 6: Singlet-A 4.5894 eV 270.16 nm f=0.1492 <S**2>=0.000
303 -> 314 0.18918
305 -> 314 -0.10201
307 -> 314 0.23812
310 -> 314 0.51339
312 -> 316 -0.15663
313 -> 315 -0.22549

Excited State 7: Singlet-A 4.6424 eV 267.07 nm f=0.0016 <S**2>=0.000
304 -> 314 -0.12068
309 -> 314 0.66085

Excited State 8: Singlet-A 4.7195 eV 262.70 nm f=0.0086 <S**2>=0.000
307 -> 314 0.62412
310 -> 314 -0.23493
311 -> 314 -0.10509

Excited State 9: Singlet-A 4.9146 eV 252.28 nm f=0.0536 <S**2>=0.000
308 -> 315 -0.11206
311 -> 314 0.61122
311 -> 317 0.10734
312 -> 316 0.12810

Excited State 10: Singlet-A 4.9711 eV 249.41 nm f=0.0011 <S**2>=0.000
299 -> 314 -0.15449
302 -> 314 0.13527
304 -> 314 0.42833
306 -> 314 0.10868
309 -> 314 0.12361
309 -> 320 -0.14454
310 -> 315 -0.25504
310 -> 318 0.10579
312 -> 315 -0.17738

Excited State 11: Singlet-A 5.0237 eV 246.80 nm f=0.0055 <S**2>=0.000
302 -> 314 0.12241
306 -> 314 0.15475
308 -> 314 0.53331
308 -> 317 0.13165
310 -> 315 0.12241
311 -> 315 -0.13324
311 -> 319 -0.12481

Excited State 12: Singlet-A 5.0417 eV 245.92 nm f=0.0015 <S**2>=0.000
302 -> 314 0.14294
304 -> 314 0.10168
308 -> 314 -0.10555
313 -> 316 0.15332
313 -> 317 0.45233
313 -> 324 0.11341
313 -> 327 -0.16193
313 -> 337 -0.16723

Excited State 13: Singlet-A 5.0702 eV 244.53 nm f=0.0038 <S**2>=0.000
301 -> 314 -0.18574
301 -> 322 0.10448
307 -> 332 -0.10509
309 -> 315 0.23816
309 -> 318 -0.21695
309 -> 319 -0.11739
310 -> 320 0.27728

312 -> 320 0.23925

Excited State 14: Singlet-A 5.0718 eV 244.46 nm f=0.0033 <S**2>=0.000
302 -> 314 0.20149
304 -> 314 0.27122
306 -> 314 0.10159
308 -> 314 -0.13750
309 -> 320 0.18244
310 -> 315 0.10613
310 -> 318 -0.12962
312 -> 318 -0.13692
313 -> 316 -0.15951
313 -> 317 -0.21720
313 -> 320 -0.10085
313 -> 327 0.12603

Excited State 15: Singlet-A 5.1571 eV 240.42 nm f=0.1020 <S**2>=0.000
298 -> 327 -0.11370
308 -> 315 0.11295
308 -> 318 0.12851
308 -> 319 -0.20061
308 -> 326 0.17070
311 -> 314 -0.14981
311 -> 316 0.15480
311 -> 317 0.33176
311 -> 325 0.15867
312 -> 316 -0.18509
312 -> 317 -0.10163
313 -> 319 0.10552

Excited State 16: Singlet-A 5.1757 eV 239.55 nm f=0.1046 <S**2>=0.000
299 -> 314 0.15418
302 -> 314 -0.17239
303 -> 315 0.12301
304 -> 314 0.25578
305 -> 315 -0.13730
306 -> 314 -0.20348
307 -> 315 0.10005
307 -> 318 -0.13894
308 -> 314 0.20723
312 -> 315 0.15881
313 -> 316 -0.21604
313 -> 317 0.14745

Excited State 17: Singlet-A 5.1966 eV 238.59 nm f=0.0521 <S**2>=0.000
300 -> 327 0.10310
308 -> 314 -0.16228
308 -> 316 0.14691
308 -> 317 0.26169
308 -> 325 0.11343
311 -> 315 0.17882
311 -> 318 0.13111
311 -> 319 -0.19530
311 -> 326 0.15554
313 -> 316 0.21650
313 -> 327 -0.12365

Excited State 18: Singlet-A 5.1994 eV 238.46 nm f=0.2154 <S**2>=0.000
302 -> 315 -0.10333
303 -> 314 0.27170
305 -> 314 -0.28793
306 -> 315 -0.13700
311 -> 314 -0.12015
312 -> 316 0.34861
312 -> 317 -0.17478

Excited State 19: Singlet-A 5.2306 eV 237.03 nm f=0.0788 <S**2>=0.000
303 -> 315 -0.11494
304 -> 314 -0.15722
305 -> 315 0.12061
306 -> 314 0.20949
307 -> 315 0.13095
307 -> 318 -0.16691

307 -> 319 -0.11117
308 -> 314 -0.15223
309 -> 320 -0.11018
310 -> 318 0.11925
312 -> 315 0.21270
312 -> 319 0.11317
313 -> 316 -0.18703

Excited State 20: Singlet-A 5.2368 eV 236.75 nm f=0.0410 <S**2>=0.000
304 -> 318 0.12255
305 -> 317 0.25623
305 -> 325 -0.11733
306 -> 318 -0.17186
306 -> 319 0.22170
307 -> 320 0.12506
311 -> 325 -0.13142
313 -> 319 0.13857

Excited State 21: Singlet-A 5.2442 eV 236.42 nm f=0.1491 <S**2>=0.000
294 -> 322 -0.10828
303 -> 314 0.11691
304 -> 315 -0.13157
304 -> 318 0.20407
304 -> 319 0.12547
305 -> 314 -0.12552
305 -> 317 -0.10691
306 -> 319 -0.10882
307 -> 320 0.22701
307 -> 332 0.11693
309 -> 318 0.16554
310 -> 332 -0.12687
312 -> 316 0.14126

Excited State 22: Singlet-A 5.2535 eV 236.00 nm f=0.0917 <S**2>=0.000
305 -> 318 0.12433
305 -> 319 -0.14066
306 -> 314 -0.10231
306 -> 317 -0.18716
307 -> 315 0.12134
307 -> 318 -0.15810
307 -> 319 -0.12431
308 -> 314 0.12552
308 -> 325 -0.12558
309 -> 332 -0.10150
312 -> 315 -0.14978
313 -> 316 0.25129

Excited State 23: Singlet-A 5.2678 eV 235.36 nm f=0.0008 <S**2>=0.000
302 -> 314 0.11334
304 -> 314 -0.13524
305 -> 318 -0.13030
305 -> 319 0.20459
306 -> 317 0.22510
306 -> 325 -0.11111
307 -> 318 -0.16443
312 -> 315 -0.13169
313 -> 316 0.18250
313 -> 317 -0.14252
313 -> 324 -0.11286

Excited State 24: Singlet-A 5.3576 eV 231.42 nm f=0.0861 <S**2>=0.000
303 -> 314 0.30877
305 -> 314 0.52258
306 -> 315 0.18355
312 -> 316 0.16881
313 -> 319 0.10401

Excited State 25: Singlet-A 5.3743 eV 230.70 nm f=0.1735 <S**2>=0.000
302 -> 314 0.45489
304 -> 314 -0.18328
306 -> 314 -0.17144
310 -> 315 -0.21225
311 -> 315 0.14020

313 -> 316 -0.24347
313 -> 317 0.14068

Excited State 26: Singlet-A 5.4008 eV 229.57 nm f=0.0350 <S**2>=0.000
303 -> 315 0.24048
306 -> 314 0.48325
310 -> 315 -0.17651
311 -> 315 0.10010
312 -> 319 -0.13809

Excited State 27: Singlet-A 5.4906 eV 225.81 nm f=0.0157 <S**2>=0.000
303 -> 314 -0.13749
305 -> 317 -0.10307
312 -> 317 -0.12978
313 -> 318 -0.28736
313 -> 319 0.50339

Excited State 28: Singlet-A 5.5741 eV 222.43 nm f=0.0043 <S**2>=0.000
299 -> 314 -0.16474
300 -> 314 0.16302
302 -> 314 -0.16687
303 -> 315 -0.10077
308 -> 314 0.17191
311 -> 315 0.51771
312 -> 318 -0.12100

Excited State 29: Singlet-A 5.5869 eV 221.92 nm f=0.0614 <S**2>=0.000
301 -> 314 0.53318
309 -> 315 0.21276
312 -> 320 0.13757
312 -> 324 -0.10109
313 -> 318 0.12767
313 -> 319 0.10835

Excited State 30: Singlet-A 5.6136 eV 220.87 nm f=0.0218 <S**2>=0.000
304 -> 315 -0.12843
308 -> 315 0.10037
309 -> 315 0.38471
312 -> 320 -0.29660
312 -> 324 0.20368
312 -> 325 -0.11654
313 -> 318 -0.13351
313 -> 321 0.11582

1c-2H (*p*-quinoid form **A**)
HOMO : 345, LUMO : 346

Excitation energies and oscillator strengths:

Excited State 1: Singlet-A 3.4165 eV 362.90 nm f=0.3585 <S**2>=0.000
345 -> 346 0.68105
This state for optimization and/or second-order correction.
Total Energy, E(TD-HF/TD-DFT) = -4400.53340110
Copying the excited state density for this state as the 1-particle RhoCI density.

Excited State 2: Singlet-A 3.8838 eV 319.23 nm f=0.2212 <S**2>=0.000
344 -> 346 0.58217
344 -> 347 -0.10375
345 -> 347 0.26455

Excited State 3: Singlet-A 4.0727 eV 304.42 nm f=0.0196 <S**2>=0.000
336 -> 346 0.16267
336 -> 347 0.11206
338 -> 346 -0.15187
343 -> 346 -0.12755
344 -> 349 -0.10516
345 -> 347 0.49904
345 -> 349 -0.22359

Excited State 4: Singlet-A 4.2375 eV 292.59 nm f=0.1084 <S**2>=0.000
336 -> 346 -0.22184

337 -> 346 -0.12117
338 -> 346 0.15478
340 -> 346 -0.10185
343 -> 346 -0.18549
343 -> 347 0.10863
344 -> 346 -0.16945
344 -> 347 0.14105
345 -> 347 0.25696
345 -> 348 0.34892
345 -> 349 0.20955

Excited State 5: Singlet-A 4.3343 eV 286.06 nm f=0.0365 <S**2>=0.000
342 -> 346 0.19497
343 -> 346 0.52041
344 -> 346 -0.14492
344 -> 347 -0.10500
345 -> 347 0.14270
345 -> 348 0.24133

Excited State 6: Singlet-A 4.3797 eV 283.09 nm f=0.2957 <S**2>=0.000
335 -> 346 0.13219
336 -> 346 0.18805
337 -> 346 0.15749
338 -> 346 -0.16463
340 -> 346 0.11691
342 -> 346 -0.10238
343 -> 346 -0.15432
344 -> 347 -0.17310
345 -> 347 -0.15730
345 -> 348 0.45545

Excited State 7: Singlet-A 4.5762 eV 270.93 nm f=0.0055 <S**2>=0.000
342 -> 346 0.57659
342 -> 347 0.14173
343 -> 346 -0.22658
343 -> 347 -0.14969
344 -> 346 0.10767

Excited State 8: Singlet-A 4.7060 eV 263.46 nm f=0.0714 <S**2>=0.000
336 -> 346 0.10184
337 -> 346 0.37856
337 -> 347 -0.13279
337 -> 348 0.10012
338 -> 346 0.41716
338 -> 347 -0.16280
339 -> 346 -0.13496
340 -> 346 -0.10292

Excited State 9: Singlet-A 4.8169 eV 257.39 nm f=0.0735 <S**2>=0.000
334 -> 346 -0.10520
335 -> 346 -0.21567
340 -> 346 -0.10894
342 -> 347 -0.17249
343 -> 347 -0.19130
343 -> 348 -0.11108
344 -> 346 0.14344
344 -> 347 0.28738
345 -> 347 -0.11016
345 -> 348 0.18321
345 -> 349 -0.21721
345 -> 351 -0.10249

Excited State 10: Singlet-A 4.8648 eV 254.86 nm f=0.0763 <S**2>=0.000
333 -> 346 0.18521
334 -> 346 -0.12424
335 -> 346 -0.21382
339 -> 346 0.21729
340 -> 346 -0.20864
341 -> 346 0.23359
343 -> 348 0.10082
344 -> 347 -0.15762
344 -> 348 -0.18859
345 -> 349 0.11007

```
Excited State  11:      Singlet-A      4.9498 eV  250.48 nm  f=0.1549  <S**2>=0.000
    333 -> 346       -0.22265
    333 -> 347        0.12158
    336 -> 346       -0.11376
    340 -> 346       -0.14599
    341 -> 346        0.27590
    343 -> 347        0.10367
    343 -> 348        0.13062
    345 -> 349       -0.13655
    345 -> 350        0.16596

Excited State  12:      Singlet-A      4.9618 eV  249.88 nm  f=0.0502  <S**2>=0.000
    338 -> 346       -0.10460
    341 -> 346        0.46785
    341 -> 347        0.11840
    343 -> 350       -0.11066
    344 -> 348        0.15720
    345 -> 349        0.17085
    345 -> 350       -0.14233

Excited State  13:      Singlet-A      4.9860 eV  248.67 nm  f=0.0509  <S**2>=0.000
    333 -> 346        0.12585
    334 -> 348        0.10158
    335 -> 346        0.15213
    341 -> 346        0.17794
    342 -> 350        0.11029
    343 -> 347       -0.12942
    343 -> 348       -0.10507
    343 -> 350        0.13388
    344 -> 347        0.10334
    344 -> 348        0.13452
    345 -> 350        0.19955
    345 -> 352       -0.12485

Excited State  14:      Singlet-A      4.9969 eV  248.12 nm  f=0.0625  <S**2>=0.000
    337 -> 346        0.23344
    338 -> 346       -0.12133
    339 -> 346        0.26043
    340 -> 346       -0.25109
    341 -> 346       -0.14993
    342 -> 346        0.12710
    344 -> 348        0.14802
    345 -> 349        0.23424

Excited State  15:      Singlet-A      5.0239 eV  246.79 nm  f=0.0929  <S**2>=0.000
    334 -> 346       -0.14404
    335 -> 346       -0.13483
    336 -> 346        0.19543
    340 -> 346        0.22785
    344 -> 351        0.11058
    345 -> 349        0.26083
    345 -> 350        0.13805
    345 -> 351       -0.10840

Excited State  16:      Singlet-A      5.0738 eV  244.36 nm  f=0.0883  <S**2>=0.000
    335 -> 346       -0.10266
    337 -> 346        0.10638
    337 -> 351        0.13460
    338 -> 346       -0.14442
    339 -> 354        0.10935
    340 -> 346        0.10363
    344 -> 347        0.13679
    345 -> 349        0.11590
    345 -> 351        0.26224
    345 -> 353        0.15054

Excited State  17:      Singlet-A      5.0814 eV  244.00 nm  f=0.0763  <S**2>=0.000
    338 -> 346        0.13871
    338 -> 351        0.10694
    339 -> 346        0.27445
    339 -> 351       -0.11834
    339 -> 354       -0.10544
```

340 -> 346 0.20326
340 -> 347 0.11780
342 -> 347 -0.11387

Excited State 18: Singlet-A 5.0929 eV 243.44 nm f=0.0168 <S**2>=0.000
338 -> 348 -0.10074
339 -> 352 -0.10180
342 -> 354 -0.14458
343 -> 352 0.11592
344 -> 347 0.11105
344 -> 352 -0.10086
345 -> 352 0.16237
345 -> 353 0.11727
345 -> 354 -0.14496

Excited State 19: Singlet-A 5.1056 eV 242.84 nm f=0.0347 <S**2>=0.000
334 -> 346 0.10834
337 -> 347 0.11395
338 -> 346 0.15564
339 -> 346 0.27617
339 -> 354 0.10325
342 -> 350 0.11738
342 -> 351 0.14161
342 -> 354 0.12722
343 -> 350 -0.11636

Excited State 20: Singlet-A 5.1409 eV 241.17 nm f=0.1083 <S**2>=0.000
335 -> 346 -0.10433
336 -> 346 -0.23570
337 -> 346 0.18432
338 -> 347 0.10404
338 -> 348 0.10622
339 -> 347 0.11414
340 -> 346 0.24622
343 -> 358 -0.10462
344 -> 348 -0.13410
345 -> 350 0.11923

Excited State 21: Singlet-A 5.1616 eV 240.21 nm f=0.0018 <S**2>=0.000
325 -> 346 -0.13096
333 -> 346 -0.16865
334 -> 346 -0.15989
335 -> 346 -0.24485
343 -> 348 0.18480
344 -> 347 -0.12844
344 -> 348 0.25769
345 -> 353 -0.10077

Excited State 22: Singlet-A 5.1767 eV 239.50 nm f=0.0193 <S**2>=0.000
340 -> 357 -0.10008
341 -> 350 0.13008
341 -> 351 -0.10722
341 -> 353 0.22084
345 -> 353 0.14567

Excited State 23: Singlet-A 5.2040 eV 238.25 nm f=0.0284 <S**2>=0.000
337 -> 346 0.11436
339 -> 346 0.20443
340 -> 346 0.18764
341 -> 353 -0.15756
342 -> 348 0.10547
344 -> 347 0.15407
344 -> 348 -0.10847

Excited State 24: Singlet-A 5.2251 eV 237.29 nm f=0.0344 <S**2>=0.000
336 -> 346 0.25992
336 -> 347 -0.11454
337 -> 346 -0.11050
339 -> 346 -0.11722
339 -> 347 -0.12206
343 -> 348 0.13176
344 -> 347 0.19824
345 -> 350 -0.14666

345 -> 356 -0.10118

Excited State 25: Singlet-A 5.2503 eV 236.15 nm f=0.0331 <S**2>=0.000
333 -> 346 -0.15944
339 -> 346 -0.10289
340 -> 353 0.21984
341 -> 357 -0.13067
341 -> 359 -0.10258
341 -> 361 0.11994
341 -> 363 0.10739
342 -> 347 -0.12808
343 -> 347 -0.12086
344 -> 347 -0.12492
344 -> 348 -0.14616
345 -> 353 0.16099

Excited State 26: Singlet-A 5.2570 eV 235.84 nm f=0.0479 <S**2>=0.000
333 -> 346 0.16040
340 -> 353 0.19932
341 -> 353 0.14024
341 -> 361 0.10257
342 -> 347 0.16173
343 -> 347 0.10541
343 -> 348 0.12654
344 -> 347 0.16325
344 -> 348 0.19060

Excited State 27: Singlet-A 5.2893 eV 234.41 nm f=0.0178 <S**2>=0.000
330 -> 346 0.11809
331 -> 346 0.11057
335 -> 347 0.11935
339 -> 346 0.12492
341 -> 353 -0.13264
342 -> 347 0.10501
344 -> 348 0.14635
345 -> 349 -0.12064
345 -> 350 0.13939
345 -> 351 0.20498
345 -> 353 0.17941
345 -> 358 -0.12100

Excited State 28: Singlet-A 5.3473 eV 231.86 nm f=0.0877 <S**2>=0.000
331 -> 346 -0.15142
333 -> 346 0.25379
334 -> 346 0.11110
336 -> 347 0.10014
343 -> 348 0.13475
344 -> 348 0.15973
345 -> 352 -0.15441
345 -> 353 0.15878
345 -> 355 -0.15224
345 -> 356 -0.11856

Excited State 29: Singlet-A 5.3779 eV 230.54 nm f=0.0130 <S**2>=0.000
333 -> 346 0.12324
336 -> 347 0.15494
339 -> 346 -0.10803
342 -> 347 -0.19165
343 -> 347 0.16716
343 -> 352 -0.13747
344 -> 352 -0.18418
344 -> 355 -0.12105
345 -> 352 0.30253
345 -> 355 0.14922
345 -> 356 -0.11465

Excited State 30: Singlet-A 5.3872 eV 230.15 nm f=0.0350 <S**2>=0.000
331 -> 346 -0.11997
336 -> 346 0.16174
339 -> 347 -0.11637
342 -> 347 -0.18086
342 -> 348 -0.15893
343 -> 347 0.25358

345 -> 350 0.24020
345 -> 356 0.14283

1c-2H (σ-bond form **B**)
HOMO : 345, LUMO : 346

Excitation energies and oscillator strengths:

Excited State 1: Singlet-A 3.6252 eV 342.01 nm f=0.1024 <S**2>=0.000
335 -> 347 -0.11059
345 -> 346 0.66312
This state for optimization and/or second-order correction.
Total Energy, E(TD-HF/TD-DFT) = -4400.48031078
Copying the excited state density for this state as the 1-particle RhoCI density.

Excited State 2: Singlet-A 3.7998 eV 326.29 nm f=0.0434 <S**2>=0.000
340 -> 346 0.10760
344 -> 346 0.63820
345 -> 347 -0.24374

Excited State 3: Singlet-A 4.2445 eV 292.10 nm f=0.0171 <S**2>=0.000
335 -> 346 0.17097
343 -> 346 0.62541
344 -> 348 0.10928

Excited State 4: Singlet-A 4.3447 eV 285.37 nm f=0.2296 <S**2>=0.000
330 -> 346 -0.15060
335 -> 346 0.37479
340 -> 346 -0.10464
343 -> 346 -0.26995
344 -> 348 0.22917
345 -> 347 -0.30181
345 -> 351 0.10458

Excited State 5: Singlet-A 4.3586 eV 284.46 nm f=0.2418 <S**2>=0.000
326 -> 346 -0.10971
329 -> 346 0.10683
344 -> 347 0.55457
345 -> 346 -0.14951
345 -> 348 0.27336

Excited State 6: Singlet-A 4.4816 eV 276.65 nm f=0.4767 <S**2>=0.000
335 -> 346 0.16825
339 -> 346 -0.11009
344 -> 346 0.23419
344 -> 348 0.18508
345 -> 347 0.54226

Excited State 7: Singlet-A 4.5951 eV 269.82 nm f=0.0039 <S**2>=0.000
341 -> 346 0.65870

Excited State 8: Singlet-A 4.6766 eV 265.12 nm f=0.0476 <S**2>=0.000
339 -> 346 0.64639
340 -> 346 0.10709

Excited State 9: Singlet-A 4.8007 eV 258.26 nm f=0.0172 <S**2>=0.000
340 -> 347 0.17257
341 -> 346 0.10968
342 -> 346 0.62612

Excited State 10: Singlet-A 4.8799 eV 254.07 nm f=0.0499 <S**2>=0.000
339 -> 346 -0.12145
340 -> 346 0.58606
342 -> 347 0.21564
344 -> 348 0.13594

Excited State 11: Singlet-A 4.9929 eV 248.32 nm f=0.0000 <S**2>=0.000
329 -> 346 0.10096
336 -> 346 0.44063
341 -> 346 0.15465
343 -> 347 -0.42139

```
Excited State  12:      Singlet-A      5.0040 eV  247.77 nm  f=0.0838  <S**2>=0.000
    333 -> 349       -0.12129
    333 -> 353        0.10729
    341 -> 354       -0.14651
    341 -> 355       -0.18780
    341 -> 358        0.12544
    343 -> 348       -0.19642
    343 -> 349        0.40549
    343 -> 353        0.22803
    343 -> 357        0.11889

Excited State  13:      Singlet-A      5.0400 eV  246.00 nm  f=0.0144  <S**2>=0.000
    345 -> 348       -0.19030
    345 -> 349       -0.27508
    345 -> 350        0.35547
    345 -> 353        0.17231
    345 -> 356       -0.25440
    345 -> 359       -0.13077
    345 -> 361        0.12226
    345 -> 368       -0.19711

Excited State  14:      Singlet-A      5.0546 eV  245.29 nm  f=0.0010  <S**2>=0.000
    331 -> 349        0.12628
    333 -> 354       -0.10042
    333 -> 365        0.11971
    336 -> 364        0.10145
    339 -> 355       -0.12031
    341 -> 348        0.12837
    341 -> 349       -0.17387
    341 -> 353       -0.20856
    341 -> 357       -0.11804
    343 -> 347       -0.16678
    343 -> 354        0.14592
    343 -> 355        0.31051
    343 -> 358       -0.18538

Excited State  15:      Singlet-A      5.1255 eV  241.90 nm  f=0.1015  <S**2>=0.000
    335 -> 346       -0.13842
    337 -> 352        0.11058
    338 -> 356       -0.10830
    340 -> 349       -0.10029
    340 -> 350        0.18896
    340 -> 356       -0.13427
    340 -> 361       -0.11223
    342 -> 351        0.24347
    342 -> 355        0.15001
    342 -> 358        0.14843
    344 -> 348        0.21208

Excited State  16:      Singlet-A      5.1320 eV  241.59 nm  f=0.0412  <S**2>=0.000
    332 -> 349       -0.16164
    334 -> 363       -0.13463
    336 -> 354        0.14286
    339 -> 348        0.10465
    339 -> 349       -0.15616
    339 -> 353       -0.10625
    339 -> 357       -0.10941
    339 -> 359       -0.10453
    341 -> 354        0.24640
    341 -> 360        0.11697
    343 -> 349       -0.10048
    343 -> 353        0.11504
    343 -> 356        0.11892
    343 -> 357       -0.14407
    343 -> 359       -0.18646
    343 -> 364        0.15981

Excited State  17:      Singlet-A      5.1361 eV  241.40 nm  f=0.0562  <S**2>=0.000
    332 -> 363       -0.14109
    334 -> 349       -0.17633
    339 -> 354        0.17891
    340 -> 351        0.10183
```

341 -> 357 -0.15730
341 -> 359 -0.16870
342 -> 350 0.11389
343 -> 354 0.27547
343 -> 360 0.10615

Excited State 18: Singlet-A 5.1373 eV 241.34 nm f=0.0190 <S**2>=0.000
337 -> 356 0.12663
337 -> 359 0.10462
338 -> 352 -0.16492
339 -> 354 -0.10463
340 -> 351 0.19244
340 -> 355 0.11144
340 -> 358 0.13794
342 -> 348 -0.13820
342 -> 349 -0.10473
342 -> 350 0.22298
342 -> 361 -0.12372
343 -> 354 -0.15042

Excited State 19: Singlet-A 5.1618 eV 240.20 nm f=0.0246 <S**2>=0.000
324 -> 346 -0.14650
326 -> 346 -0.15010
329 -> 346 0.20172
330 -> 347 -0.10354
331 -> 346 0.13423
335 -> 347 0.22913
337 -> 346 0.26929
338 -> 347 -0.15872
342 -> 346 0.12748
343 -> 347 0.22297
345 -> 348 0.14109

Excited State 20: Singlet-A 5.1632 eV 240.13 nm f=0.0863 <S**2>=0.000
335 -> 346 -0.13176
337 -> 351 0.21135
338 -> 346 0.18977
338 -> 350 -0.21753
338 -> 361 0.10300
340 -> 346 -0.13809
340 -> 356 0.14074
340 -> 359 0.11164
342 -> 352 0.23281
342 -> 355 -0.10464
344 -> 348 0.17734

Excited State 21: Singlet-A 5.1810 eV 239.31 nm f=0.0484 <S**2>=0.000
337 -> 350 0.20048
337 -> 361 -0.11908
338 -> 351 -0.21737
338 -> 352 -0.12497
340 -> 352 0.20811
340 -> 355 -0.12548
340 -> 358 -0.10327
342 -> 356 0.19579
342 -> 359 0.15143

Excited State 22: Singlet-A 5.1823 eV 239.25 nm f=0.1157 <S**2>=0.000
335 -> 346 -0.28056
337 -> 351 -0.10224
337 -> 352 -0.15389
338 -> 346 0.20686
338 -> 350 0.15109
340 -> 346 -0.13065
342 -> 351 -0.10269
344 -> 348 0.31956
344 -> 350 0.11476

Excited State 23: Singlet-A 5.2166 eV 237.67 nm f=0.2428 <S**2>=0.000
333 -> 349 0.12841
341 -> 355 0.10951
341 -> 363 0.14795
343 -> 349 0.43105

343 -> 350 0.19638
343 -> 353 -0.23392
343 -> 357 -0.15890
343 -> 359 -0.10184

Excited State 24: Singlet-A 5.2305 eV 237.04 nm f=0.2217 <S**2>=0.000
336 -> 346 -0.10990
340 -> 347 0.12343
343 -> 347 -0.10911
344 -> 347 -0.33286
345 -> 348 0.45371
345 -> 350 0.12961
345 -> 353 0.11857

Excited State 25: Singlet-A 5.2361 eV 236.79 nm f=0.0717 <S**2>=0.000
335 -> 346 0.26080
337 -> 347 -0.15706
338 -> 346 0.53508
342 -> 347 -0.20847
344 -> 348 -0.13645

Excited State 26: Singlet-A 5.3304 eV 232.60 nm f=0.0599 <S**2>=0.000
335 -> 347 -0.13430
336 -> 346 -0.22818
337 -> 346 0.50386
338 -> 347 -0.20874
343 -> 347 -0.20223

Excited State 27: Singlet-A 5.3717 eV 230.81 nm f=0.0533 <S**2>=0.000
339 -> 349 -0.24554
343 -> 348 0.12689
344 -> 349 0.54818
344 -> 350 0.11154

Excited State 28: Singlet-A 5.3814 eV 230.39 nm f=0.1210 <S**2>=0.000
326 -> 346 0.11550
329 -> 346 -0.14233
331 -> 346 -0.27757
334 -> 346 0.29068
335 -> 347 -0.10315
336 -> 346 0.31127
337 -> 346 0.13709
340 -> 347 -0.15574
343 -> 347 0.19984
345 -> 348 0.18400

Excited State 29: Singlet-A 5.4490 eV 227.54 nm f=0.0565 <S**2>=0.000
335 -> 346 0.10839
337 -> 347 -0.15480
338 -> 346 0.12834
340 -> 346 -0.19291
340 -> 348 -0.13605
341 -> 347 -0.10680
342 -> 347 0.52292
343 -> 348 0.10378
344 -> 349 -0.10099
345 -> 351 -0.10772

Excited State 30: Singlet-A 5.4662 eV 226.82 nm f=0.0001 <S**2>=0.000
334 -> 346 0.32663
335 -> 347 0.15850
336 -> 346 -0.16036
337 -> 346 -0.11581
339 -> 347 -0.15560
340 -> 347 -0.25931
341 -> 349 0.18634
343 -> 347 -0.24852
345 -> 348 0.13530

1c-2H (*o*-diphenoquinoid form **C**)
HOMO : 345, LUMO : 346

Excitation energies and oscillator strengths:

```
Excited State   1:      Singlet-A      2.3239 eV  533.52 nm  f=0.2153  <S**2>=0.000
     345 -> 346         0.69765
 This state for optimization and/or second-order correction.
 Total Energy, E(TD-HF/TD-DFT) =  -4400.59247469
 Copying the excited state density for this state as the 1-particle RhoCI density.

 Excited State   2:      Singlet-A      3.2251 eV  384.44 nm  f=0.0923  <S**2>=0.000
     325 -> 346         0.10658
     343 -> 346         0.66341
     344 -> 346        -0.10040

 Excited State   3:      Singlet-A      3.2515 eV  381.31 nm  f=0.0367  <S**2>=0.000
     344 -> 346         0.67958

 Excited State   4:      Singlet-A      3.9123 eV  316.91 nm  f=0.0635  <S**2>=0.000
     345 -> 347         0.65317

 Excited State   5:      Singlet-A      4.0033 eV  309.71 nm  f=0.0013  <S**2>=0.000
     333 -> 346         0.10833
     335 -> 346         0.35931
     337 -> 346         0.20723
     338 -> 346        -0.19882
     342 -> 346         0.45159
     345 -> 347        -0.14117

 Excited State   6:      Singlet-A      4.0447 eV  306.53 nm  f=0.0048  <S**2>=0.000
     336 -> 346         0.36182
     339 -> 346         0.41862
     341 -> 346         0.27408
     345 -> 348        -0.25508

 Excited State   7:      Singlet-A      4.1628 eV  297.84 nm  f=0.0183  <S**2>=0.000
     335 -> 346        -0.18518
     337 -> 346        -0.12285
     338 -> 346         0.34535
     340 -> 346         0.38617
     342 -> 346         0.32537

 Excited State   8:      Singlet-A      4.2151 eV  294.14 nm  f=0.0359  <S**2>=0.000
     334 -> 346         0.16252
     335 -> 346         0.26770
     339 -> 346         0.15909
     340 -> 346         0.34720
     341 -> 346         0.13374
     342 -> 346        -0.26506
     345 -> 348         0.32969

 Excited State   9:      Singlet-A      4.2167 eV  294.03 nm  f=0.0449  <S**2>=0.000
     334 -> 346         0.17610
     335 -> 346        -0.24914
     337 -> 346        -0.15543
     339 -> 346         0.16285
     340 -> 346        -0.20153
     341 -> 346         0.16570
     342 -> 346         0.27288
     343 -> 346         0.10078
     345 -> 348         0.37156

 Excited State  10:      Singlet-A      4.3160 eV  287.26 nm  f=0.0777  <S**2>=0.000
     334 -> 346         0.10355
     337 -> 346        -0.19174
     338 -> 346        -0.21560
     339 -> 346        -0.33269
     340 -> 346         0.12350
     341 -> 346         0.44958
     345 -> 348        -0.19829

 Excited State  11:      Singlet-A      4.3436 eV  285.44 nm  f=0.0736  <S**2>=0.000
     336 -> 346         0.56759
     339 -> 346        -0.24765
     341 -> 346        -0.11492
```

345 -> 348 0.21771

Excited State 12: Singlet-A 4.4283 eV 279.98 nm f=0.1177 <S**2>=0.000
334 -> 346 -0.34148
337 -> 346 0.20834
338 -> 346 0.30069
339 -> 346 -0.15500
340 -> 346 -0.13130
341 -> 346 0.34493
345 -> 348 0.20090

Excited State 13: Singlet-A 4.4756 eV 277.02 nm f=0.0084 <S**2>=0.000
331 -> 346 0.15159
337 -> 346 0.14182
338 -> 346 -0.11290
345 -> 349 0.24813
345 -> 350 0.49412
345 -> 351 -0.12230
345 -> 353 -0.20896

Excited State 14: Singlet-A 4.5529 eV 272.32 nm f=0.0443 <S**2>=0.000
333 -> 346 -0.28489
334 -> 346 0.32938
335 -> 346 0.28244
338 -> 346 0.27563
340 -> 346 -0.25726
345 -> 350 0.10681

Excited State 15: Singlet-A 4.5826 eV 270.56 nm f=0.0573 <S**2>=0.000
332 -> 346 0.11379
333 -> 346 0.51157
334 -> 346 0.24646
337 -> 346 0.13158
338 -> 346 0.21921

Excited State 16: Singlet-A 4.6232 eV 268.18 nm f=0.0155 <S**2>=0.000
331 -> 346 0.13357
333 -> 346 -0.21404
334 -> 346 0.24926
335 -> 346 -0.24548
337 -> 346 0.48337
345 -> 350 -0.10825

Excited State 17: Singlet-A 4.6329 eV 267.62 nm f=0.0033 <S**2>=0.000
343 -> 349 -0.12651
345 -> 349 0.54354
345 -> 350 -0.25382
345 -> 353 0.11941
345 -> 357 0.10234
345 -> 368 0.10105

Excited State 18: Singlet-A 4.7016 eV 263.71 nm f=0.0272 <S**2>=0.000
328 -> 346 -0.11081
331 -> 346 0.49267
337 -> 346 -0.15030
340 -> 346 -0.14385
343 -> 347 -0.18372
345 -> 349 -0.14299
345 -> 353 0.15716
345 -> 356 -0.11341
345 -> 365 -0.10487

Excited State 19: Singlet-A 4.7362 eV 261.78 nm f=0.0539 <S**2>=0.000
325 -> 346 0.46736
327 -> 346 -0.14403
329 -> 346 -0.11660
330 -> 346 -0.13702
343 -> 348 0.12483
345 -> 352 0.11911
345 -> 354 -0.16506
345 -> 364 -0.11083

Excited State 20: Singlet-A 4.7952 eV 258.56 nm f=0.2039 <S**2>=0.000

```
    325 -> 346        0.21131
    344 -> 347        0.32293
    345 -> 351        0.30401
    345 -> 352       -0.24146
    345 -> 353        0.14697
    345 -> 354        0.20511
    345 -> 364        0.12805
    345 -> 366       -0.11554

 Excited State  21:      Singlet-A      4.8354 eV  256.41 nm  f=0.0144  <S**2>=0.000
    325 -> 346        0.11165
    334 -> 346        0.15505
    343 -> 354        0.10845
    344 -> 347        0.26320
    345 -> 351       -0.16245
    345 -> 352        0.24722
    345 -> 354        0.16972
    345 -> 355        0.26828
    345 -> 356       -0.11022
    345 -> 358       -0.14469
    345 -> 359        0.10118
    345 -> 364       -0.18442

 Excited State  22:      Singlet-A      4.9346 eV  251.25 nm  f=0.0196  <S**2>=0.000
    325 -> 346       -0.15861
    345 -> 350        0.13075
    345 -> 351        0.42095
    345 -> 352        0.26436
    345 -> 364       -0.19376

 Excited State  23:      Singlet-A      4.9399 eV  250.98 nm  f=0.1073  <S**2>=0.000
    333 -> 346       -0.17209
    343 -> 347       -0.17064
    344 -> 348        0.22417
    344 -> 354       -0.17234
    344 -> 355       -0.12699
    345 -> 356        0.17113
    345 -> 357       -0.20192
    345 -> 358       -0.19776
    345 -> 359        0.12527
    345 -> 360       -0.15104

 Excited State  24:      Singlet-A      5.0037 eV  247.79 nm  f=0.0194  <S**2>=0.000
    331 -> 346        0.28512
    343 -> 347        0.19823
    345 -> 350       -0.19256
    345 -> 351        0.10624
    345 -> 353       -0.28321
    345 -> 356        0.28141
    345 -> 365        0.11861

 Excited State  25:      Singlet-A      5.0235 eV  246.81 nm  f=0.1541  <S**2>=0.000
    325 -> 346       -0.15031
    343 -> 348        0.12309
    344 -> 347        0.45279
    345 -> 352        0.12652
    345 -> 354       -0.26596
    345 -> 355       -0.19597
    345 -> 359       -0.12764

 Excited State  26:      Singlet-A      5.0657 eV  244.75 nm  f=0.0391  <S**2>=0.000
    332 -> 346        0.44358
    342 -> 349        0.24382
    343 -> 347        0.10910
    344 -> 347       -0.11191
    345 -> 354        0.12326
    345 -> 358       -0.10580

 Excited State  27:      Singlet-A      5.0831 eV  243.92 nm  f=0.0038  <S**2>=0.000
    323 -> 346        0.11083
    329 -> 346        0.17524
    330 -> 346       -0.12120
    332 -> 346       -0.12944
```

341 -> 349 0.12249
343 -> 347 0.42542
344 -> 348 0.19353
345 -> 353 0.10855

Excited State 28: Singlet-A 5.1389 eV 241.27 nm f=0.0169 <S**2>=0.000
329 -> 346 -0.22285
330 -> 346 0.16918
332 -> 346 0.26695
342 -> 349 -0.12586
343 -> 347 0.24475
345 -> 353 0.10044
345 -> 354 -0.24302
345 -> 355 0.23556

Excited State 29: Singlet-A 5.1425 eV 241.10 nm f=0.0096 <S**2>=0.000
329 -> 346 0.29298
330 -> 346 -0.21419
332 -> 346 0.21031
341 -> 349 0.10731
343 -> 347 -0.12525
345 -> 352 -0.16523
345 -> 353 -0.12067
345 -> 354 -0.11916
345 -> 355 0.29436
345 -> 358 0.11550

Excited State 30: Singlet-A 5.1588 eV 240.33 nm f=0.0253 <S**2>=0.000
332 -> 346 0.30754
342 -> 349 -0.21114
345 -> 352 0.11643
345 -> 353 -0.10776
345 -> 354 0.11433
345 -> 355 -0.24582

1a^{2+} (*p*-quinoid form **A**)
HOMO : 343, LUMO : 344

Excitation energies and oscillator strengths:

Excited State 1: Singlet-A 1.2473 eV 994.05 nm f=0.0327 <S**2>=0.000
340 -> 344 -0.12900
341 -> 344 0.19815
342 -> 344 -0.12842
343 -> 344 0.59192
343 -> 345 -0.24044
This state for optimization and/or second-order correction.
Total Energy, E(TD-HF/TD-DFT) = -4878.30989987
Copying the excited state density for this state as the 1-particle RhoCI density.

Excited State 2: Singlet-A 1.6874 eV 734.76 nm f=0.0021 <S**2>=0.000
340 -> 344 -0.18244
340 -> 345 -0.12501
341 -> 344 0.14630
341 -> 345 0.18389
342 -> 345 -0.14029
343 -> 344 0.18718
343 -> 345 0.57015

Excited State 3: Singlet-A 1.9931 eV 622.07 nm f=0.1050 <S**2>=0.000
334 -> 344 -0.12461
339 -> 344 0.12340
340 -> 344 0.48227
341 -> 344 -0.25704
342 -> 344 0.12084
343 -> 344 0.28660
343 -> 345 0.13691

Excited State 4: Singlet-A 2.0270 eV 611.65 nm f=0.0332 <S**2>=0.000
340 -> 344 -0.18893
340 -> 345 0.16816

```
    341 -> 345       -0.11225
    342 -> 344        0.59065
    342 -> 345       -0.18837

 Excited State   5:      Singlet-A      2.3298 eV  532.16 nm  f=0.0015  <S**2>=0.000
    340 -> 344        0.34237
    341 -> 344        0.48111
    341 -> 345       -0.28410
    342 -> 345       -0.22346

 Excited State   6:      Singlet-A      2.4263 eV  511.00 nm  f=0.0160  <S**2>=0.000
    339 -> 345        0.14350
    340 -> 345        0.54109
    341 -> 345       -0.17963
    342 -> 344       -0.18359
    343 -> 345        0.21300

 Excited State   7:      Singlet-A      2.4989 eV  496.16 nm  f=0.0046  <S**2>=0.000
    335 -> 345        0.10216
    339 -> 344        0.10668
    340 -> 345       -0.15156
    341 -> 344        0.14458
    341 -> 345       -0.22404
    342 -> 344        0.16666
    342 -> 345        0.54175
    343 -> 345        0.14350

 Excited State   8:      Singlet-A      2.6517 eV  467.57 nm  f=0.0154  <S**2>=0.000
    333 -> 344       -0.14979
    334 -> 344        0.10690
    335 -> 345       -0.12952
    337 -> 344        0.14965
    338 -> 344        0.45951
    339 -> 344       -0.31233
    339 -> 345        0.19958
    342 -> 344        0.12899
    342 -> 345        0.12628

 Excited State   9:      Singlet-A      2.7386 eV  452.73 nm  f=0.0163  <S**2>=0.000
    333 -> 344       -0.10308
    335 -> 344       -0.24936
    336 -> 344        0.14411
    337 -> 344        0.11045
    338 -> 344        0.29062
    338 -> 345        0.22580
    339 -> 344        0.42867
    339 -> 345       -0.10344
    340 -> 345        0.12718

 Excited State  10:      Singlet-A      2.7881 eV  444.69 nm  f=0.4392  <S**2>=0.000
    331 -> 344        0.14054
    331 -> 345        0.14509
    332 -> 344        0.49253
    332 -> 345        0.11024
    333 -> 344        0.11411
    334 -> 344       -0.27840
    335 -> 344        0.16369

 Excited State  11:      Singlet-A      2.8239 eV  439.05 nm  f=0.2976  <S**2>=0.000
    331 -> 344        0.22524
    332 -> 345        0.22772
    333 -> 344        0.39284
    333 -> 345       -0.33452
    335 -> 344       -0.12542
    338 -> 345        0.10153
    341 -> 345        0.14137

 Excited State  12:      Singlet-A      2.8890 eV  429.15 nm  f=0.1796  <S**2>=0.000
    330 -> 344       -0.13935
    331 -> 344        0.33229
    331 -> 345        0.13320
    332 -> 344        0.17612
    332 -> 345        0.10261
```

333 -> 344 -0.22800
334 -> 344 0.23299
338 -> 344 -0.20442
339 -> 345 -0.19311
340 -> 344 0.10425
340 -> 345 0.14305
342 -> 344 0.10606

Excited State 13: Singlet-A 2.9009 eV 427.39 nm f=0.0218 <S**2>=0.000
331 -> 344 -0.20011
332 -> 345 -0.11068
339 -> 345 -0.11160
340 -> 345 0.22009
341 -> 344 0.29669
341 -> 345 0.45277
342 -> 345 0.16826

Excited State 14: Singlet-A 3.0094 eV 411.99 nm f=0.0277 <S**2>=0.000
329 -> 344 0.19970
331 -> 344 -0.18321
332 -> 344 0.18416
333 -> 344 0.20367
334 -> 344 0.34985
337 -> 344 -0.30724
337 -> 345 0.20689
338 -> 344 0.18409

Excited State 15: Singlet-A 3.0636 eV 404.70 nm f=0.0227 <S**2>=0.000
329 -> 344 0.11259
331 -> 344 -0.10913
333 -> 344 0.14959
334 -> 344 0.27178
337 -> 344 0.44456
337 -> 345 -0.30192
338 -> 345 0.13752
343 -> 346 -0.15405

Excited State 16: Singlet-A 3.1289 eV 396.26 nm f=0.1082 <S**2>=0.000
331 -> 344 0.10708
331 -> 345 -0.14377
332 -> 345 0.20341
334 -> 344 0.21451
334 -> 345 -0.23499
335 -> 344 0.16925
338 -> 345 -0.20203
339 -> 344 0.20686
339 -> 345 0.14830
343 -> 346 0.30479

Excited State 17: Singlet-A 3.1433 eV 394.44 nm f=0.2682 <S**2>=0.000
332 -> 344 0.15637
332 -> 345 -0.12635
333 -> 345 -0.10539
335 -> 344 -0.15449
338 -> 345 0.10902
339 -> 344 -0.12040
339 -> 345 -0.10574
341 -> 346 0.11370
343 -> 346 0.50884
343 -> 347 0.13716

Excited State 18: Singlet-A 3.2546 eV 380.96 nm f=0.0065 <S**2>=0.000
327 -> 345 -0.11682
329 -> 344 -0.12005
331 -> 344 0.12435
332 -> 345 -0.10625
333 -> 345 -0.27119
334 -> 344 0.12160
334 -> 345 0.20168
335 -> 344 0.32805
335 -> 345 -0.28146
336 -> 344 0.15556
341 -> 345 -0.10454

342 -> 345 0.11925

Excited State 19: Singlet-A 3.2875 eV 377.14 nm f=0.0617 <S**2>=0.000
329 -> 344 0.31742
329 -> 345 -0.13463
330 -> 344 -0.21268
331 -> 345 0.17150
334 -> 345 0.12560
335 -> 345 -0.16560
339 -> 344 0.16637
339 -> 345 0.35221

Excited State 20: Singlet-A 3.2966 eV 376.10 nm f=0.0158 <S**2>=0.000
329 -> 344 0.29235
330 -> 344 -0.24501
332 -> 344 -0.19021
332 -> 345 0.13698
333 -> 345 0.18816
334 -> 344 -0.13638
335 -> 344 0.17736
336 -> 344 0.13844
338 -> 345 0.14465
339 -> 344 -0.13525
339 -> 345 -0.27988
343 -> 346 0.11246

Excited State 21: Singlet-A 3.3922 eV 365.50 nm f=0.0054 <S**2>=0.000
329 -> 345 -0.14546
333 -> 345 -0.10182
334 -> 345 -0.28398
336 -> 344 0.41890
337 -> 345 0.13479
338 -> 344 -0.21591
338 -> 345 0.19119
339 -> 344 -0.16983
339 -> 345 0.13317

Excited State 22: Singlet-A 3.4047 eV 364.16 nm f=0.0025 <S**2>=0.000
324 -> 344 -0.11084
329 -> 344 -0.20063
329 -> 345 0.22136
330 -> 344 -0.25480
330 -> 345 -0.14329
331 -> 344 -0.11706
331 -> 345 -0.11297
333 -> 344 0.12566
333 -> 345 0.14651
334 -> 345 0.27607
335 -> 344 -0.19664
336 -> 344 0.22856
338 -> 344 -0.11361

Excited State 23: Singlet-A 3.4739 eV 356.90 nm f=0.0018 <S**2>=0.000
329 -> 344 0.20097
330 -> 344 0.45789
330 -> 345 0.18323
331 -> 344 0.17600
333 -> 345 0.16328
334 -> 345 0.19472
335 -> 344 -0.12009
336 -> 344 0.21079

Excited State 24: Singlet-A 3.5380 eV 350.43 nm f=0.0061 <S**2>=0.000
329 -> 344 -0.14440
331 -> 345 -0.11303
332 -> 345 0.19670
333 -> 345 0.11568
334 -> 344 0.10554
334 -> 345 0.13109
335 -> 344 0.18321
336 -> 344 -0.17890
336 -> 345 0.11410
338 -> 345 0.41786

339 -> 345 0.20790

Excited State 25: Singlet-A 3.5871 eV 345.64 nm f=0.0137 <S**2>=0.000
324 -> 345 -0.11072
325 -> 344 0.25541
325 -> 345 -0.16004
327 -> 344 0.19233
327 -> 345 -0.23234
328 -> 344 0.12961
329 -> 345 0.20310
333 -> 345 0.11344
334 -> 345 -0.22432
335 -> 345 -0.15643
336 -> 344 -0.19014
338 -> 345 0.23325

Excited State 26: Singlet-A 3.6123 eV 343.23 nm f=0.0422 <S**2>=0.000
321 -> 344 0.15808
324 -> 344 0.41252
324 -> 345 0.20738
325 -> 344 -0.20119
326 -> 345 0.11038
333 -> 344 0.11973
333 -> 345 0.14738
335 -> 344 -0.11354
335 -> 345 -0.21978
337 -> 344 -0.11551
337 -> 345 -0.15601

Excited State 27: Singlet-A 3.6655 eV 338.25 nm f=0.0673 <S**2>=0.000
324 -> 344 0.16577
325 -> 344 0.14670
325 -> 345 -0.22877
326 -> 344 0.14879
326 -> 345 -0.11168
327 -> 344 0.15361
327 -> 345 -0.15103
334 -> 345 0.19274
335 -> 345 0.20933
341 -> 346 -0.15970
342 -> 346 0.26576
343 -> 347 0.19751

Excited State 28: Singlet-A 3.6730 eV 337.56 nm f=0.0042 <S**2>=0.000
333 -> 345 0.11283
336 -> 344 -0.13255
337 -> 344 0.34074
337 -> 345 0.52752
338 -> 344 -0.10781
338 -> 345 -0.11670

Excited State 29: Singlet-A 3.6855 eV 336.41 nm f=0.0823 <S**2>=0.000
325 -> 345 0.14207
326 -> 344 -0.16968
326 -> 345 0.15184
327 -> 344 -0.12647
327 -> 345 0.11144
328 -> 344 0.10174
334 -> 345 -0.15964
340 -> 346 0.13882
341 -> 346 -0.21033
342 -> 346 0.31572
343 -> 347 0.27189

Excited State 30: Singlet-A 3.7282 eV 332.56 nm f=0.0054 <S**2>=0.000
322 -> 344 -0.22570
322 -> 345 -0.12346
323 -> 344 0.36190
323 -> 345 0.19670
327 -> 344 0.12401
329 -> 345 -0.13953
331 -> 344 -0.14083
332 -> 345 0.26703

335 -> 344 -0.11771
335 -> 345 -0.17046

1a^{2+} (σ-bond form **B**)
HOMO : 343, LUMO : 344

Excitation energies and oscillator strengths:

Excited State 1: Singlet-A 1.9368 eV 640.15 nm f=0.1529 <S**2>=0.000
339 -> 345 -0.15748
340 -> 344 0.52208
341 -> 345 -0.37523
342 -> 344 -0.17396
This state for optimization and/or second-order correction.
Total Energy, E(TD-HF/TD-DFT) = -4878.27631795
Copying the excited state density for this state as the 1-particle RhoCI density.

Excited State 2: Singlet-A 2.0681 eV 599.50 nm f=0.4412 <S**2>=0.000
339 -> 344 -0.17281
340 -> 345 0.49673
341 -> 344 -0.41279
342 -> 345 -0.16136

Excited State 3: Singlet-A 2.3489 eV 527.84 nm f=0.0278 <S**2>=0.000
330 -> 344 0.16322
331 -> 345 0.10790
335 -> 345 -0.11183
339 -> 345 0.22357
340 -> 344 0.32227
341 -> 345 0.47386
342 -> 344 -0.17829
343 -> 345 -0.12253

Excited State 4: Singlet-A 2.3655 eV 524.15 nm f=0.0064 <S**2>=0.000
330 -> 345 0.13841
331 -> 344 0.10618
335 -> 344 -0.10011
339 -> 344 0.20782
340 -> 345 0.32338
341 -> 344 0.39231
342 -> 345 -0.20201
343 -> 344 -0.30681

Excited State 5: Singlet-A 2.4410 eV 507.93 nm f=0.0506 <S**2>=0.000
340 -> 345 0.19797
341 -> 344 0.23698
343 -> 344 0.61053

Excited State 6: Singlet-A 2.4627 eV 503.45 nm f=0.0021 <S**2>=0.000
338 -> 344 -0.11160
340 -> 344 0.11584
341 -> 345 0.11877
343 -> 345 0.65854

Excited State 7: Singlet-A 2.7212 eV 455.62 nm f=0.0037 <S**2>=0.000
339 -> 345 -0.25639
340 -> 344 0.21083
341 -> 345 0.15139
342 -> 344 0.57616
343 -> 345 -0.14601

Excited State 8: Singlet-A 2.7390 eV 452.66 nm f=0.0020 <S**2>=0.000
339 -> 344 -0.33979
340 -> 345 0.20331
341 -> 344 0.19325
342 -> 345 0.52371
343 -> 344 -0.11687

Excited State 9: Singlet-A 2.9485 eV 420.51 nm f=0.0026 <S**2>=0.000
335 -> 344 -0.10599
339 -> 344 0.51614

340 -> 345 0.11132
341 -> 344 -0.21560
342 -> 345 0.36879

Excited State 10: Singlet-A 2.9615 eV 418.66 nm f=0.0872 <S**2>=0.000
332 -> 344 0.14027
333 -> 345 0.14327
335 -> 345 -0.12871
339 -> 345 0.52984
341 -> 345 -0.23991
342 -> 344 0.27621

Excited State 11: Singlet-A 3.0139 eV 411.38 nm f=0.6982 <S**2>=0.000
331 -> 345 -0.22114
332 -> 344 0.44104
333 -> 345 0.35804
335 -> 345 -0.19340
339 -> 345 -0.19860
342 -> 344 -0.11113

Excited State 12: Singlet-A 3.0267 eV 409.64 nm f=0.0403 <S**2>=0.000
331 -> 344 -0.21126
332 -> 345 0.43477
333 -> 344 0.39745
335 -> 344 -0.23422
339 -> 344 -0.11212

Excited State 13: Singlet-A 3.2483 eV 381.69 nm f=0.0437 <S**2>=0.000
330 -> 344 0.12045
331 -> 345 -0.14979
335 -> 345 0.13403
338 -> 344 0.62279
343 -> 345 0.11375

Excited State 14: Singlet-A 3.2693 eV 379.24 nm f=0.0000 <S**2>=0.000
330 -> 345 0.12096
331 -> 344 -0.31071
335 -> 344 0.26407
338 -> 345 0.48084
340 -> 345 0.11970

Excited State 15: Singlet-A 3.3369 eV 371.56 nm f=0.0404 <S**2>=0.000
327 -> 345 -0.11155
331 -> 344 0.34423
333 -> 344 0.14515
335 -> 344 -0.26934
338 -> 345 0.45070
341 -> 344 -0.10507

Excited State 16: Singlet-A 3.3380 eV 371.43 nm f=0.0081 <S**2>=0.000
327 -> 344 -0.14968
331 -> 345 0.41053
333 -> 345 0.16678
335 -> 345 -0.36993
336 -> 344 -0.10208
338 -> 344 0.21857
339 -> 345 -0.11475
340 -> 344 -0.12479
341 -> 345 -0.10184

Excited State 17: Singlet-A 3.4561 eV 358.74 nm f=0.0077 <S**2>=0.000
330 -> 344 -0.12541
331 -> 345 0.16265
333 -> 345 0.13046
334 -> 344 0.16068
336 -> 344 0.55447
337 -> 345 0.27438

Excited State 18: Singlet-A 3.4579 eV 358.55 nm f=0.0001 <S**2>=0.000
331 -> 344 0.12083
333 -> 344 0.10790
334 -> 345 0.19926
336 -> 345 0.30912

337 -> 344 0.55615

Excited State 19: Singlet-A 3.5179 eV 352.44 nm f=0.0000 <S**2>=0.000
330 -> 345 -0.14895
335 -> 344 0.11418
336 -> 345 0.53886
337 -> 344 -0.36176

Excited State 20: Singlet-A 3.5199 eV 352.24 nm f=0.0018 <S**2>=0.000
330 -> 344 0.11439
334 -> 344 0.18324
336 -> 344 -0.30222
337 -> 345 0.58374

Excited State 21: Singlet-A 3.5608 eV 348.19 nm f=0.0427 <S**2>=0.000
324 -> 345 0.13382
325 -> 344 -0.10254
327 -> 344 0.12029
329 -> 345 0.13377
330 -> 344 0.55798
332 -> 344 -0.11716
333 -> 345 0.10236
336 -> 344 0.15089
338 -> 344 -0.15003
340 -> 344 -0.11909
341 -> 345 -0.10078

Excited State 22: Singlet-A 3.6087 eV 343.57 nm f=0.0076 <S**2>=0.000
324 -> 344 0.16045
325 -> 345 -0.17800
326 -> 344 0.14760
327 -> 345 0.11726
329 -> 344 0.13470
330 -> 345 0.52225
332 -> 345 -0.11669
333 -> 344 0.13996
336 -> 345 0.13064
338 -> 345 -0.14300
340 -> 345 -0.10281

Excited State 23: Singlet-A 3.6884 eV 336.14 nm f=0.0057 <S**2>=0.000
325 -> 344 0.45676
326 -> 345 -0.45062
328 -> 344 -0.14768
329 -> 345 -0.10955
330 -> 344 0.11121

Excited State 24: Singlet-A 3.6961 eV 335.45 nm f=0.0481 <S**2>=0.000
325 -> 345 -0.42191
326 -> 344 0.45474
328 -> 345 0.13412
330 -> 345 -0.19666

Excited State 25: Singlet-A 3.7806 eV 327.95 nm f=0.0986 <S**2>=0.000
324 -> 345 -0.16089
325 -> 344 0.11711
327 -> 344 -0.21261
328 -> 344 0.12091
329 -> 345 0.26047
330 -> 344 0.15083
331 -> 345 0.26431
332 -> 344 0.38430
333 -> 345 -0.20549
335 -> 345 0.11202

Excited State 26: Singlet-A 3.7918 eV 326.98 nm f=0.0128 <S**2>=0.000
324 -> 344 -0.12695
327 -> 345 -0.17791
329 -> 344 0.21504
330 -> 345 0.19247
331 -> 344 0.30190
332 -> 345 0.42301
333 -> 344 -0.21038

335 -> 344   0.15297

Excited State 27:   Singlet-A   3.8347 eV 323.32 nm f=0.1264 <S**2>=0.000
323 -> 345   0.11684
324 -> 345   -0.11603
325 -> 344   0.11066
327 -> 344   -0.17412
328 -> 344   0.38056
329 -> 345   0.26086
331 -> 345   -0.16580
332 -> 344   -0.24688
333 -> 345   0.28746

Excited State 28:   Singlet-A   3.8376 eV 323.07 nm f=0.0185 <S**2>=0.000
327 -> 345   -0.14798
328 -> 345   0.11260
332 -> 345   -0.13365
333 -> 344   0.43395
334 -> 345   0.10747
335 -> 344   0.38204
336 -> 345   -0.23569

Excited State 29:   Singlet-A   3.8621 eV 321.03 nm f=0.0051 <S**2>=0.000
331 -> 345   0.14717
333 -> 345   0.27560
334 -> 344   0.42145
335 -> 345   0.35653
336 -> 344   -0.17895
337 -> 345   -0.20916

Excited State 30:   Singlet-A   3.8662 eV 320.69 nm f=0.0903 <S**2>=0.000
323 -> 344   0.15955
324 -> 344   -0.13828
325 -> 345   0.10454
327 -> 345   -0.21362
328 -> 345   0.34375
329 -> 344   0.30287
331 -> 344   -0.18199
332 -> 345   -0.15833
334 -> 345   -0.14415
335 -> 344   -0.23500
336 -> 345   0.10437

1a^{2+} (*o*-diphenoquinoid form **C**)
HOMO : 343, LUMO : 344

Excitation energies and oscillator strengths:

Excited State   1:   Singlet-A   1.1671 eV 1062.37 nm f=0.1931 <S**2>=0.000
343 -> 344   0.67902
This state for optimization and/or second-order correction.
Total Energy, E(TD-HF/TD-DFT) = -4878.28726143
Copying the excited state density for this state as the 1-particle RhoCI density.

Excited State   2:   Singlet-A   1.5897 eV 779.90 nm f=0.1824 <S**2>=0.000
339 -> 345   0.10041
343 -> 345   0.66005

Excited State   3:   Singlet-A   1.7393 eV 712.85 nm f=0.0103 <S**2>=0.000
340 -> 344   0.28818
341 -> 344   -0.15144
342 -> 344   0.58569
342 -> 346   0.11235
343 -> 345   -0.11714

Excited State   4:   Singlet-A   1.9796 eV 626.29 nm f=0.0150 <S**2>=0.000
339 -> 344   -0.38212
341 -> 344   0.50448
343 -> 344   0.13570

Excited State   5:   Singlet-A   2.1881 eV 566.63 nm f=0.0080 <S**2>=0.000

337 -> 344 -0.18369
338 -> 344 0.14453
339 -> 344 -0.31122
340 -> 344 0.31373
340 -> 345 -0.15952
341 -> 344 -0.28198
341 -> 345 0.11830
342 -> 344 -0.27489

Excited State 6: Singlet-A 2.2217 eV 558.07 nm f=0.0039 <S**2>=0.000
337 -> 344 0.13929
338 -> 344 -0.11750
339 -> 344 0.26062
340 -> 344 0.50776
341 -> 344 0.17452
342 -> 344 -0.18485
342 -> 345 0.15940

Excited State 7: Singlet-A 2.3012 eV 538.77 nm f=0.0041 <S**2>=0.000
339 -> 344 -0.19242
340 -> 345 0.24705
341 -> 344 -0.25130
341 -> 345 -0.12594
342 -> 345 0.55056

Excited State 8: Singlet-A 2.5509 eV 486.04 nm f=0.0029 <S**2>=0.000
334 -> 344 -0.11902
339 -> 345 -0.38722
341 -> 345 0.47356
343 -> 345 0.18495

Excited State 9: Singlet-A 2.6819 eV 462.29 nm f=0.0643 <S**2>=0.000
334 -> 344 0.24163
334 -> 345 -0.10645
335 -> 344 -0.25495
337 -> 344 -0.21849
337 -> 345 0.14683
338 -> 344 0.13971
338 -> 345 -0.10269
339 -> 344 0.18343
341 -> 345 0.22933
342 -> 345 0.21400
343 -> 346 0.25682

Excited State 10: Singlet-A 2.7001 eV 459.19 nm f=0.0669 <S**2>=0.000
332 -> 344 0.14260
334 -> 344 -0.25111
335 -> 344 0.33732
336 -> 344 -0.11655
337 -> 344 -0.19242
338 -> 344 0.22629
339 -> 344 0.19006
340 -> 345 -0.23293
341 -> 345 -0.15093
342 -> 345 0.18366

Excited State 11: Singlet-A 2.7330 eV 453.65 nm f=0.1133 <S**2>=0.000
335 -> 344 0.14844
335 -> 345 0.10797
340 -> 345 0.25093
341 -> 346 -0.10462
342 -> 345 -0.15691
343 -> 346 0.56106

Excited State 12: Singlet-A 2.7728 eV 447.15 nm f=0.0077 <S**2>=0.000
335 -> 344 0.33421
337 -> 344 0.14263
337 -> 345 0.15755
338 -> 345 -0.13245
339 -> 345 0.36492
340 -> 344 -0.11777
341 -> 345 0.31335

Excited State 13: Singlet-A 2.8057 eV 441.90 nm f=0.0525 <S**2>=0.000
329 -> 344 0.12613
330 -> 344 -0.12307
331 -> 344 0.13188
332 -> 344 -0.15921
334 -> 345 0.11329
337 -> 344 -0.25616
338 -> 344 0.23866
339 -> 344 0.16045
340 -> 345 0.41615
342 -> 345 -0.12013
343 -> 346 -0.16485

Excited State 14: Singlet-A 2.8640 eV 432.90 nm f=0.4324 <S**2>=0.000
330 -> 345 0.10518
331 -> 344 -0.27448
331 -> 345 0.13071
332 -> 344 0.33781
332 -> 345 -0.11784
333 -> 344 0.10657
334 -> 344 0.21649
334 -> 345 -0.26431
335 -> 345 0.11100
340 -> 345 0.19087
342 -> 345 -0.11056
343 -> 346 -0.13354

Excited State 15: Singlet-A 2.8824 eV 430.15 nm f=0.1560 <S**2>=0.000
330 -> 344 0.43858
330 -> 345 0.13794
331 -> 344 -0.11008
331 -> 345 -0.13966
332 -> 345 0.18724
334 -> 344 -0.29153
335 -> 344 -0.22152
339 -> 345 0.12809
340 -> 345 0.10213
341 -> 345 0.11037

Excited State 16: Singlet-A 3.0670 eV 404.25 nm f=0.0072 <S**2>=0.000
332 -> 344 -0.10443
333 -> 344 0.47923
335 -> 345 0.13645
336 -> 344 0.28363
337 -> 344 0.13618
338 -> 344 0.25888

Excited State 17: Singlet-A 3.0846 eV 401.94 nm f=0.0053 <S**2>=0.000
330 -> 344 0.30478
331 -> 344 0.12958
332 -> 344 -0.12399
333 -> 344 0.11742
334 -> 344 0.17912
335 -> 344 0.11057
337 -> 345 0.15899
338 -> 345 -0.13678
339 -> 345 -0.27017
340 -> 346 -0.13155
341 -> 345 -0.11353
342 -> 346 -0.28737

Excited State 18: Singlet-A 3.1397 eV 394.90 nm f=0.0256 <S**2>=0.000
329 -> 344 0.20930
330 -> 344 -0.15663
331 -> 344 0.23068
332 -> 344 0.29679
334 -> 344 -0.11248
335 -> 344 -0.20086
337 -> 344 0.28199
338 -> 344 0.18329
338 -> 345 -0.16045
342 -> 346 -0.15342

Excited State 19: Singlet-A 3.1549 eV 392.99 nm f=0.0706 <S**2>=0.000
329 -> 344 0.56137
330 -> 345 0.11654
332 -> 344 -0.15479
334 -> 344 0.10203
335 -> 344 0.11126
335 -> 345 0.13179
338 -> 344 -0.11766
340 -> 345 -0.11899

Excited State 20: Singlet-A 3.1844 eV 389.35 nm f=0.0305 <S**2>=0.000
330 -> 344 0.17384
333 -> 344 -0.13321
334 -> 344 0.19442
335 -> 345 -0.10881
336 -> 344 -0.23999
337 -> 344 0.29070
337 -> 345 -0.13324
338 -> 344 0.35009
340 -> 346 0.11591
342 -> 346 0.24159

Excited State 21: Singlet-A 3.2119 eV 386.01 nm f=0.0412 <S**2>=0.000
328 -> 344 -0.11001
330 -> 345 0.12563
331 -> 344 -0.22513
333 -> 344 -0.24259
335 -> 345 -0.23678
336 -> 344 0.42380
338 -> 344 0.18502
342 -> 346 -0.16260

Excited State 22: Singlet-A 3.2261 eV 384.31 nm f=0.0165 <S**2>=0.000
329 -> 345 0.10780
330 -> 344 0.12109
331 -> 344 0.34373
332 -> 344 0.24194
334 -> 344 0.10412
336 -> 344 0.31838
337 -> 344 -0.16439
340 -> 346 0.12081
342 -> 346 0.27324

Excited State 23: Singlet-A 3.2743 eV 378.66 nm f=0.0797 <S**2>=0.000
328 -> 344 0.16204
330 -> 345 -0.19394
332 -> 345 0.19024
333 -> 344 -0.32486
334 -> 345 -0.19441
335 -> 345 0.35554
336 -> 344 0.16300
343 -> 346 -0.11652

Excited State 24: Singlet-A 3.3919 eV 365.53 nm f=0.0042 <S**2>=0.000
329 -> 345 -0.13766
331 -> 344 -0.17007
334 -> 344 -0.10548
337 -> 345 0.36362
338 -> 345 -0.32091
339 -> 345 -0.22154
342 -> 346 0.24930

Excited State 25: Singlet-A 3.4903 eV 355.23 nm f=0.0228 <S**2>=0.000
328 -> 344 0.17757
339 -> 346 -0.27368
341 -> 346 0.50152
342 -> 346 0.19076

Excited State 26: Singlet-A 3.5688 eV 347.41 nm f=0.0056 <S**2>=0.000
324 -> 344 -0.10614
327 -> 344 0.17449
328 -> 344 0.11985
329 -> 345 -0.15535

332 -> 344 0.18128
332 -> 345 0.14160
333 -> 345 0.11863
334 -> 344 0.18653
334 -> 345 0.34271
339 -> 346 -0.11254
340 -> 346 0.17274
341 -> 346 -0.13556
342 -> 346 -0.14191

Excited State 27: Singlet-A 3.5905 eV 345.31 nm f=0.0465 <S**2>=0.000
324 -> 344 0.17109
324 -> 345 0.11619
327 -> 344 -0.18032
327 -> 345 -0.16059
328 -> 344 -0.29660
328 -> 345 0.11080
329 -> 344 -0.11488
334 -> 345 0.22604
335 -> 345 0.34152
339 -> 346 -0.10879

Excited State 28: Singlet-A 3.6218 eV 342.33 nm f=0.0252 <S**2>=0.000
324 -> 344 -0.15853
325 -> 344 -0.14386
326 -> 344 0.25087
326 -> 345 -0.23308
327 -> 344 0.15609
328 -> 344 -0.23607
328 -> 345 0.22372
329 -> 345 -0.11735
331 -> 345 -0.11721
332 -> 345 0.11038
334 -> 345 -0.16050
341 -> 346 0.21466

Excited State 29: Singlet-A 3.6559 eV 339.13 nm f=0.0548 <S**2>=0.000
321 -> 344 -0.10198
323 -> 344 -0.10414
324 -> 344 0.14457
324 -> 345 0.10967
325 -> 344 -0.10300
325 -> 345 0.14882
327 -> 344 -0.22581
328 -> 344 0.11547
329 -> 345 -0.10260
330 -> 344 -0.11962
331 -> 345 -0.17885
333 -> 345 0.30494
336 -> 345 0.21370
338 -> 345 0.23385

Excited State 30: Singlet-A 3.6777 eV 337.12 nm f=0.0543 <S**2>=0.000
325 -> 344 0.18875
325 -> 345 -0.18574
326 -> 344 -0.11611
327 -> 344 0.17026
328 -> 344 -0.10334
331 -> 344 0.12473
332 -> 345 -0.12768
333 -> 345 0.20518
334 -> 345 -0.14662
336 -> 345 0.21815
337 -> 345 0.17776
338 -> 345 0.23290
340 -> 346 0.17138

1b^{2+} (*p*-quinoid form **A**)
HOMO : 311, LUMO : 312

Excitation energies and oscillator strengths:

Excited State 1: Singlet-A 1.5116 eV 820.22 nm f=0.0388 <S**2>=0.000
308 -> 312 -0.18799
309 -> 312 0.19240
309 -> 313 -0.12835
310 -> 312 -0.26500
310 -> 313 0.17998
311 -> 312 0.49542
311 -> 313 -0.22392
This state for optimization and/or second-order correction.
Total Energy, E(TD-HF/TD-DFT) = -4084.56535840
Copying the excited state density for this state as the 1-particle RhoCI density.

Excited State 2: Singlet-A 1.8670 eV 664.07 nm f=0.0050 <S**2>=0.000
308 -> 312 -0.27333
308 -> 313 -0.10831
309 -> 313 0.21319
310 -> 313 -0.30196
311 -> 312 0.30222
311 -> 313 0.40548

Excited State 3: Singlet-A 1.9898 eV 623.09 nm f=0.0435 <S**2>=0.000
308 -> 312 0.39111
309 -> 312 -0.14079
310 -> 312 0.36195
311 -> 312 0.37293

Excited State 4: Singlet-A 2.1154 eV 586.09 nm f=0.0575 <S**2>=0.000
307 -> 312 -0.12640
307 -> 313 0.13576
308 -> 312 0.33559
308 -> 313 -0.29166
309 -> 312 0.18585
310 -> 312 -0.26567
310 -> 313 0.11866
311 -> 313 0.33276

Excited State 5: Singlet-A 2.3222 eV 533.91 nm f=0.0017 <S**2>=0.000
308 -> 313 -0.14968
309 -> 312 0.43761
309 -> 313 -0.31893
310 -> 312 0.26797
310 -> 313 -0.28836

Excited State 6: Singlet-A 2.4306 eV 510.11 nm f=0.0162 <S**2>=0.000
307 -> 313 -0.10636
308 -> 312 -0.12847
308 -> 313 0.35158
309 -> 313 -0.24906
310 -> 312 0.18462
310 -> 313 0.28598
311 -> 313 0.35370

Excited State 7: Singlet-A 2.4558 eV 504.86 nm f=0.0071 <S**2>=0.000
306 -> 312 -0.14643
307 -> 312 0.49590
308 -> 312 0.18345
308 -> 313 0.27447
309 -> 312 0.16203
310 -> 312 -0.12326
310 -> 313 -0.18391

Excited State 8: Singlet-A 2.6119 eV 474.69 nm f=0.0040 <S**2>=0.000
307 -> 312 0.38368
307 -> 313 0.20011
308 -> 312 -0.10938
308 -> 313 -0.31281
309 -> 312 -0.14740
309 -> 313 -0.12524
310 -> 312 0.22648
310 -> 313 0.28439

Excited State 9: Singlet-A 2.8406 eV 436.47 nm f=0.2636 <S**2>=0.000

301 -> 312 0.51418
301 -> 313 0.14360
302 -> 312 0.13049
308 -> 312 0.10440
309 -> 312 0.20612
309 -> 313 0.25621
310 -> 312 0.10146
310 -> 313 0.15012

Excited State 10: Singlet-A 2.8737 eV 431.45 nm f=0.4208 <S**2>=0.000
301 -> 312 0.30608
302 -> 312 -0.20371
302 -> 313 0.22544
304 -> 312 -0.22665
304 -> 313 0.19399
309 -> 312 -0.22811
309 -> 313 -0.23594
310 -> 312 -0.13761
310 -> 313 -0.16682

Excited State 11: Singlet-A 2.8907 eV 428.91 nm f=0.1603 <S**2>=0.000
301 -> 312 -0.16297
302 -> 312 -0.26910
302 -> 313 0.28492
304 -> 312 -0.17900
304 -> 313 0.19266
309 -> 312 0.23798
309 -> 313 0.33580
310 -> 312 0.10417
310 -> 313 0.13205

Excited State 12: Singlet-A 2.9734 eV 416.98 nm f=0.0843 <S**2>=0.000
301 -> 312 -0.10034
302 -> 312 0.11349
302 -> 313 -0.18077
303 -> 312 0.12801
303 -> 313 -0.11080
305 -> 312 0.43259
305 -> 313 -0.30896
306 -> 312 0.28238
306 -> 313 -0.10392

Excited State 13: Singlet-A 3.0010 eV 413.14 nm f=0.0942 <S**2>=0.000
299 -> 312 -0.13828
300 -> 312 0.54375
301 -> 313 0.15460
302 -> 312 -0.19237
304 -> 312 0.11884
307 -> 313 -0.10988
308 -> 312 -0.12378
308 -> 313 -0.10410

Excited State 14: Singlet-A 3.0727 eV 403.50 nm f=0.0157 <S**2>=0.000
300 -> 312 -0.12075
302 -> 312 -0.15561
302 -> 313 0.15647
303 -> 312 -0.12513
303 -> 313 0.11964
305 -> 312 -0.13008
305 -> 313 0.11759
306 -> 312 0.50303
306 -> 313 -0.13555
307 -> 312 0.16471
307 -> 313 -0.17815

Excited State 15: Singlet-A 3.1902 eV 388.64 nm f=0.0378 <S**2>=0.000
299 -> 312 -0.12722
300 -> 313 0.25832
301 -> 313 -0.19019
304 -> 313 0.11258
306 -> 312 0.15196
307 -> 312 -0.10637
307 -> 313 0.45225

311 -> 314 -0.17178

Excited State 16: Singlet-A 3.2363 eV 383.10 nm f=0.3557 <S**2>=0.000
297 -> 312 0.10648
301 -> 313 -0.11937
307 -> 313 0.16212
308 -> 314 -0.10311
309 -> 314 0.13579
310 -> 314 -0.14566
311 -> 314 0.51585
311 -> 315 0.11897

Excited State 17: Singlet-A 3.2763 eV 378.43 nm f=0.0351 <S**2>=0.000
298 -> 312 -0.23100
298 -> 313 0.20533
299 -> 312 0.32349
299 -> 313 -0.24192
303 -> 312 0.19451
303 -> 313 -0.16446
304 -> 312 0.12063
304 -> 313 -0.10751
305 -> 312 -0.10377
307 -> 313 0.12848
311 -> 314 -0.22215

Excited State 18: Singlet-A 3.3864 eV 366.13 nm f=0.0314 <S**2>=0.000
297 -> 312 -0.20784
300 -> 313 -0.17156
301 -> 313 0.26560
302 -> 313 0.10842
303 -> 312 0.13169
303 -> 313 -0.11893
306 -> 312 0.28643
306 -> 313 0.32278
307 -> 313 0.21591
308 -> 313 0.12421

Excited State 19: Singlet-A 3.4136 eV 363.20 nm f=0.1083 <S**2>=0.000
297 -> 312 0.36091
299 -> 313 0.13816
300 -> 312 -0.10588
304 -> 313 -0.12628
305 -> 312 0.25733
305 -> 313 0.25574
306 -> 313 0.30346

Excited State 20: Singlet-A 3.4442 eV 359.98 nm f=0.0089 <S**2>=0.000
298 -> 313 -0.11352
299 -> 312 -0.24436
300 -> 312 -0.11961
300 -> 313 0.18008
301 -> 313 -0.12252
303 -> 312 0.37357
303 -> 313 -0.31236
304 -> 312 0.24509
304 -> 313 -0.12897

Excited State 21: Singlet-A 3.4766 eV 356.62 nm f=0.0003 <S**2>=0.000
298 -> 312 0.52867
298 -> 313 0.11045
299 -> 312 0.36208
299 -> 313 0.15175

Excited State 22: Singlet-A 3.4910 eV 355.15 nm f=0.0037 <S**2>=0.000
300 -> 313 0.14606
301 -> 313 -0.23270
302 -> 312 0.11275
302 -> 313 -0.23826
304 -> 312 -0.20252
304 -> 313 0.17682
305 -> 312 -0.25821
306 -> 313 0.30947
307 -> 312 0.12824

307 -> 313 -0.14328
308 -> 313 -0.10790

Excited State 23: Singlet-A 3.5421 eV 350.03 nm f=0.0248 <S**2>=0.000
296 -> 312 -0.11534
297 -> 312 -0.29270
298 -> 313 0.12279
299 -> 313 -0.14770
300 -> 313 0.12750
301 -> 313 -0.17429
303 -> 312 -0.10598
304 -> 312 0.18028
305 -> 312 0.30188
305 -> 313 0.23279
306 -> 313 0.21015
307 -> 313 -0.13646

Excited State 24: Singlet-A 3.5980 eV 344.59 nm f=0.0089 <S**2>=0.000
297 -> 312 0.15863
297 -> 313 -0.21108
302 -> 313 0.10782
303 -> 312 -0.24534
304 -> 312 0.38111
304 -> 313 0.15623
305 -> 313 -0.29885
306 -> 313 0.17496
307 -> 313 0.11126
310 -> 314 -0.10123

Excited State 25: Singlet-A 3.6734 eV 337.52 nm f=0.0789 <S**2>=0.000
302 -> 312 -0.16972
305 -> 313 -0.12902
306 -> 313 0.13399
308 -> 314 0.27095
309 -> 314 -0.22194
310 -> 314 0.40852
311 -> 314 0.13771
311 -> 315 0.13961
311 -> 318 -0.10773

Excited State 26: Singlet-A 3.7117 eV 334.03 nm f=0.0549 <S**2>=0.000
295 -> 312 -0.11118
297 -> 312 -0.12778
297 -> 313 0.32982
300 -> 312 -0.14009
302 -> 312 -0.32522
302 -> 313 -0.22877
303 -> 312 -0.12643
305 -> 313 -0.12115
308 -> 314 -0.12380
310 -> 314 -0.15072

Excited State 27: Singlet-A 3.8196 eV 324.60 nm f=0.0350 <S**2>=0.000
289 -> 312 0.13780
293 -> 312 0.13816
296 -> 313 0.15163
297 -> 313 0.20337
298 -> 312 -0.22296
298 -> 313 -0.14745
299 -> 312 0.20337
299 -> 313 0.15132
300 -> 313 0.13180
301 -> 313 0.10428
302 -> 312 0.11400
303 -> 312 0.14484
303 -> 313 0.13643
304 -> 312 0.17339
304 -> 313 0.23786

Excited State 28: Singlet-A 3.8339 eV 323.39 nm f=0.0390 <S**2>=0.000
289 -> 312 0.26547
289 -> 313 0.12535
291 -> 312 0.11094

292 -> 312 -0.15416
292 -> 313 -0.12631
293 -> 313 -0.15965
294 -> 312 -0.11711
295 -> 312 0.21357
296 -> 312 -0.23586
296 -> 313 0.11674
297 -> 312 -0.10398
297 -> 313 -0.11141
302 -> 312 -0.15073
304 -> 312 -0.12442
304 -> 313 -0.19787

Excited State 29: Singlet-A 3.8435 eV 322.58 nm f=0.0046 <S**2>=0.000
289 -> 312 0.37613
289 -> 313 0.17465
292 -> 312 -0.29080
293 -> 312 -0.10782
293 -> 313 0.21980
295 -> 313 0.14791
296 -> 312 0.16431
296 -> 313 -0.14280
299 -> 312 -0.15411

Excited State 30: Singlet-A 3.9137 eV 316.80 nm f=0.0411 <S**2>=0.000
307 -> 314 -0.14054
308 -> 314 0.48316
309 -> 314 0.24101
310 -> 314 -0.22991

1b^{2+} (σ-bond form **B**)
HOMO : 311, LUMO : 312

Excitation energies and oscillator strengths:

Excited State 1: Singlet-A 1.9550 eV 634.18 nm f=0.1025 <S**2>=0.000
305 -> 313 0.11787
306 -> 313 0.33114
307 -> 312 -0.30565
308 -> 313 -0.30866
309 -> 312 0.23283
310 -> 313 0.18036
311 -> 312 -0.27967
This state for optimization and/or second-order correction.
Total Energy, E(TD-HF/TD-DFT) = -4084.55214813
Copying the excited state density for this state as the 1-particle RhoCI density.

Excited State 2: Singlet-A 2.0276 eV 611.49 nm f=0.3458 <S**2>=0.000
305 -> 312 0.11750
306 -> 312 0.33505
307 -> 313 -0.28284
308 -> 312 -0.31946
309 -> 313 0.23460
310 -> 312 0.20219
311 -> 313 -0.27384

Excited State 3: Singlet-A 2.3283 eV 532.52 nm f=0.0288 <S**2>=0.000
306 -> 313 0.28349
308 -> 313 -0.17393
309 -> 312 -0.15867
310 -> 313 0.15939
311 -> 312 0.54984

Excited State 4: Singlet-A 2.3551 eV 526.46 nm f=0.0133 <S**2>=0.000
306 -> 312 0.25291
308 -> 312 -0.16725
309 -> 313 -0.15227
310 -> 312 0.20166
311 -> 313 0.56258

Excited State 5: Singlet-A 2.4627 eV 503.44 nm f=0.0017 <S**2>=0.000
306 -> 312 -0.23125
307 -> 313 0.25000
309 -> 313 0.29280
310 -> 312 0.52612

Excited State 6: Singlet-A 2.4636 eV 503.27 nm f=0.0143 <S**2>=0.000
306 -> 313 -0.18903
308 -> 313 0.11870
309 -> 312 0.45021
310 -> 313 0.43661
311 -> 312 0.15922

Excited State 7: Singlet-A 2.4875 eV 498.42 nm f=0.0164 <S**2>=0.000
298 -> 313 0.11742
307 -> 312 0.43208
308 -> 313 -0.23736
309 -> 312 -0.23761
310 -> 313 0.31428
311 -> 312 -0.24482

Excited State 8: Singlet-A 2.4965 eV 496.63 nm f=0.0033 <S**2>=0.000
298 -> 312 -0.11728
307 -> 313 -0.36385
308 -> 312 0.27545
309 -> 313 0.40433
311 -> 313 0.27945

Excited State 9: Singlet-A 2.6896 eV 460.97 nm f=0.0040 <S**2>=0.000
307 -> 312 0.35761
308 -> 313 -0.20336
309 -> 312 0.37868
310 -> 313 -0.37684

Excited State 10: Singlet-A 2.6980 eV 459.54 nm f=0.0055 <S**2>=0.000
307 -> 313 0.36092
308 -> 312 -0.22458
309 -> 313 0.40272
310 -> 312 -0.34156

Excited State 11: Singlet-A 2.9406 eV 421.63 nm f=0.0442 <S**2>=0.000
303 -> 312 0.14287
305 -> 313 0.10563
306 -> 313 0.40750
307 -> 312 0.16814
308 -> 313 0.48727
311 -> 312 -0.12523

Excited State 12: Singlet-A 2.9408 eV 421.61 nm f=0.0347 <S**2>=0.000
305 -> 312 0.11482
306 -> 312 0.42396
307 -> 313 0.20467
308 -> 312 0.46776
311 -> 313 -0.11591

Excited State 13: Singlet-A 3.0193 eV 410.63 nm f=0.0959 <S**2>=0.000
300 -> 312 0.46133
303 -> 313 0.49691

Excited State 14: Singlet-A 3.0238 eV 410.03 nm f=0.8165 <S**2>=0.000
299 -> 312 -0.11159
300 -> 313 0.45500
303 -> 312 0.48624

Excited State 15: Singlet-A 3.3015 eV 375.54 nm f=0.0005 <S**2>=0.000
298 -> 312 0.10499
301 -> 313 0.11955
302 -> 312 0.11343
305 -> 312 0.61641
306 -> 312 -0.20241

Excited State 16: Singlet-A 3.3155 eV 373.95 nm f=0.0478 <S**2>=0.000
298 -> 313 0.11768

301 -> 312 0.11860
302 -> 313 0.10416
304 -> 312 -0.10413
305 -> 313 0.60956
306 -> 313 -0.21666

Excited State 17: Singlet-A 3.4222 eV 362.29 nm f=0.0047 <S**2>=0.000
301 -> 312 0.17626
302 -> 313 0.20500
304 -> 312 0.61817

Excited State 18: Singlet-A 3.4396 eV 360.46 nm f=0.0005 <S**2>=0.000
298 -> 312 -0.10634
301 -> 313 0.15671
302 -> 312 0.21803
304 -> 313 0.62858

Excited State 19: Singlet-A 3.4630 eV 358.02 nm f=0.0810 <S**2>=0.000
294 -> 313 -0.10269
296 -> 313 0.14517
299 -> 312 0.52336
300 -> 313 -0.17756
301 -> 312 -0.13662
303 -> 312 0.22262
304 -> 312 0.10678
306 -> 313 -0.17221
307 -> 312 -0.12116

Excited State 20: Singlet-A 3.4682 eV 357.49 nm f=0.0048 <S**2>=0.000
294 -> 312 -0.10441
296 -> 312 0.14432
299 -> 313 0.52099
300 -> 312 -0.17294
301 -> 313 -0.15081
303 -> 313 0.22808
306 -> 312 -0.16879
307 -> 313 -0.12735

Excited State 21: Singlet-A 3.6704 eV 337.79 nm f=0.1571 <S**2>=0.000
307 -> 314 0.20676
308 -> 315 -0.12212
309 -> 314 -0.32696
311 -> 314 0.53425

Excited State 22: Singlet-A 3.7506 eV 330.57 nm f=0.0382 <S**2>=0.000
290 -> 313 0.14460
291 -> 312 0.10164
296 -> 313 -0.13932
297 -> 312 -0.21460
298 -> 313 0.47528
300 -> 313 -0.19410
303 -> 312 0.15449
304 -> 312 0.17223
305 -> 313 -0.12677
307 -> 312 -0.12206

Excited State 23: Singlet-A 3.7558 eV 330.11 nm f=0.0007 <S**2>=0.000
290 -> 312 0.13667
296 -> 312 -0.10055
297 -> 313 -0.18032
298 -> 312 0.48508
300 -> 312 -0.21235
303 -> 313 0.18576
304 -> 313 0.18149
305 -> 312 -0.10932
307 -> 313 -0.12917

Excited State 24: Singlet-A 3.8256 eV 324.09 nm f=0.0268 <S**2>=0.000
290 -> 313 -0.11031
291 -> 312 -0.22634
294 -> 313 0.26520
295 -> 312 0.18040
296 -> 313 0.27730

297 -> 312 0.26183
298 -> 313 0.16276
299 -> 312 -0.18931
301 -> 312 -0.26258

Excited State 25: Singlet-A 3.8265 eV 324.01 nm f=0.1937 <S**2>=0.000
291 -> 313 0.15821
294 -> 312 -0.16916
295 -> 313 -0.12690
296 -> 312 -0.19369
297 -> 313 -0.17283
301 -> 313 0.12054
302 -> 312 -0.20355
306 -> 314 0.11077
308 -> 314 -0.23851
310 -> 314 0.41225

Excited State 26: Singlet-A 3.8398 eV 322.89 nm f=0.0055 <S**2>=0.000
302 -> 312 0.59881
304 -> 313 -0.20033
305 -> 312 -0.14987
310 -> 314 0.18044

Excited State 27: Singlet-A 3.8555 eV 321.58 nm f=0.0039 <S**2>=0.000
302 -> 313 0.62689
304 -> 312 -0.20343
305 -> 313 -0.17465

Excited State 28: Singlet-A 3.8607 eV 321.15 nm f=0.0332 <S**2>=0.000
290 -> 312 -0.12584
291 -> 313 -0.23045
294 -> 312 0.22126
295 -> 313 0.12425
296 -> 312 0.20667
297 -> 313 0.17461
298 -> 312 0.15701
299 -> 313 -0.12366
301 -> 313 -0.20076
308 -> 314 -0.16277
310 -> 314 0.34299

Excited State 29: Singlet-A 3.8897 eV 318.75 nm f=0.0017 <S**2>=0.000
298 -> 313 0.16375
299 -> 312 0.15317
300 -> 313 0.36831
301 -> 312 -0.34762
303 -> 312 -0.31419
309 -> 314 0.12018
311 -> 314 0.11293

Excited State 30: Singlet-A 3.8964 eV 318.20 nm f=0.0003 <S**2>=0.000
297 -> 313 -0.10870
298 -> 312 0.20685
299 -> 313 0.21250
300 -> 312 0.39790
301 -> 313 -0.26760
303 -> 313 -0.33780
310 -> 314 -0.10003

1b^{2+} (*o*-diphenoquinoid form **C**)
HOMO : 311, LUMO : 312

Excitation energies and oscillator strengths:

Excited State 1: Singlet-A 1.0991 eV 1128.04 nm f=0.2097 <S**2>=0.000
309 -> 312 0.10444
311 -> 312 0.68843
This state for optimization and/or second-order correction.
Total Energy, E(TD-HF/TD-DFT) = -4084.55622329
Copying the excited state density for this state as the 1-particle RhoCI density.

```
Excited State   2:      Singlet-A      1.6069 eV  771.60 nm  f=0.1223  <S**2>=0.000
    308 -> 312        0.34604
    310 -> 312        0.57876
    310 -> 314       -0.11232
    311 -> 313        0.12570

Excited State   3:      Singlet-A      1.7635 eV  703.05 nm  f=0.1533  <S**2>=0.000
    307 -> 313       -0.10333
    309 -> 313        0.12672
    310 -> 312       -0.11633
    311 -> 313        0.66076

Excited State   4:      Singlet-A      1.8639 eV  665.17 nm  f=0.0890  <S**2>=0.000
    306 -> 312       -0.10050
    307 -> 312        0.52569
    309 -> 312       -0.38773
    311 -> 314       -0.11749

Excited State   5:      Singlet-A      2.1082 eV  588.11 nm  f=0.0054  <S**2>=0.000
    308 -> 312        0.58242
    308 -> 314       -0.10514
    310 -> 312       -0.35513

Excited State   6:      Singlet-A      2.1230 eV  584.02 nm  f=0.0164  <S**2>=0.000
    306 -> 312       -0.18085
    307 -> 312        0.34045
    309 -> 312        0.54822
    309 -> 314       -0.11557

Excited State   7:      Singlet-A      2.3862 eV  519.60 nm  f=0.0012  <S**2>=0.000
    306 -> 312        0.24745
    307 -> 312        0.12395
    308 -> 313        0.33919
    310 -> 313        0.52873

Excited State   8:      Singlet-A      2.5800 eV  480.56 nm  f=0.0829  <S**2>=0.000
    307 -> 312        0.15638
    310 -> 313       -0.10845
    311 -> 314        0.64216

Excited State   9:      Singlet-A      2.6387 eV  469.87 nm  f=0.0150  <S**2>=0.000
    298 -> 312        0.10020
    303 -> 312        0.38111
    306 -> 313       -0.12929
    307 -> 313        0.45015
    309 -> 313       -0.24376
    311 -> 313        0.18446

Excited State  10:      Singlet-A      2.6974 eV  459.64 nm  f=0.0074  <S**2>=0.000
    306 -> 312        0.57582
    307 -> 312        0.11217
    310 -> 313       -0.28928
    311 -> 314       -0.17280

Excited State  11:      Singlet-A      2.7442 eV  451.80 nm  f=0.0347  <S**2>=0.000
    301 -> 313       -0.15309
    302 -> 312        0.12038
    303 -> 312        0.53097
    307 -> 313       -0.28097
    309 -> 313        0.25347

Excited State  12:      Singlet-A      2.8578 eV  433.84 nm  f=0.5121  <S**2>=0.000
    297 -> 312        0.12539
    300 -> 312       -0.15519
    301 -> 312        0.54850
    302 -> 312       -0.16140
    303 -> 313       -0.20545
    307 -> 312       -0.14975
    308 -> 313        0.12173

Excited State  13:      Singlet-A      2.8875 eV  429.39 nm  f=0.0065  <S**2>=0.000
    298 -> 312        0.13738
    301 -> 312        0.15070
```

301 -> 313 0.13038
302 -> 312 0.49022
305 -> 312 0.17366
306 -> 313 0.16801
307 -> 313 -0.11470
308 -> 314 0.12479
309 -> 313 -0.27480

Excited State 14: Singlet-A 2.9543 eV 419.67 nm f=0.0104 <S**2>=0.000
298 -> 312 0.17390
299 -> 312 -0.28222
301 -> 313 0.17241
302 -> 312 0.19322
306 -> 313 -0.12484
307 -> 313 0.20611
308 -> 313 -0.12684
308 -> 314 -0.10771
309 -> 313 0.44034

Excited State 15: Singlet-A 2.9580 eV 419.14 nm f=0.0627 <S**2>=0.000
301 -> 312 -0.14088
306 -> 312 -0.10193
308 -> 313 0.54590
309 -> 313 0.11204
309 -> 314 -0.12542
310 -> 313 -0.30712

Excited State 16: Singlet-A 3.0322 eV 408.90 nm f=0.1031 <S**2>=0.000
297 -> 312 -0.23836
299 -> 313 -0.10594
300 -> 312 0.55864
301 -> 312 0.15051
303 -> 313 -0.12106
304 -> 312 0.10669
308 -> 313 0.12243

Excited State 17: Singlet-A 3.1115 eV 398.47 nm f=0.0001 <S**2>=0.000
299 -> 312 0.31607
305 -> 312 0.19355
306 -> 313 0.14829
307 -> 313 0.25620
308 -> 314 0.18375
309 -> 313 0.21337
310 -> 314 0.38419

Excited State 18: Singlet-A 3.1459 eV 394.11 nm f=0.0289 <S**2>=0.000
299 -> 312 -0.34024
302 -> 312 -0.11576
305 -> 312 -0.29602
306 -> 313 0.11292
308 -> 314 0.23872
310 -> 314 0.38389

Excited State 19: Singlet-A 3.2389 eV 382.79 nm f=0.0225 <S**2>=0.000
298 -> 312 0.52996
298 -> 314 -0.10687
299 -> 312 0.28161
305 -> 312 -0.28729

Excited State 20: Singlet-A 3.2451 eV 382.06 nm f=0.0035 <S**2>=0.000
297 -> 312 0.11604
298 -> 312 0.16755
304 -> 312 0.61726
304 -> 314 -0.11063
305 -> 312 0.17330
306 -> 312 0.10745

Excited State 21: Singlet-A 3.2881 eV 377.07 nm f=0.0109 <S**2>=0.000
298 -> 312 0.26898
299 -> 312 -0.18574
302 -> 312 -0.31375
304 -> 312 -0.21353
305 -> 312 0.45432

```
Excited State  22:      Singlet-A      3.3895 eV  365.79 nm  f=0.0891  <S**2>=0.000
     297 -> 312         0.41426
     297 -> 314        -0.12097
     298 -> 313        -0.15236
     299 -> 313         0.13541
     300 -> 312         0.21017
     301 -> 312        -0.15079
     303 -> 313        -0.24137
     307 -> 314        -0.25378
     309 -> 314         0.17756

Excited State  23:      Singlet-A      3.3949 eV  365.21 nm  f=0.1152  <S**2>=0.000
     296 -> 312         0.18502
     297 -> 312         0.37845
     300 -> 312         0.20973
     301 -> 312         0.10571
     302 -> 313         0.14997
     303 -> 313         0.38791
     307 -> 314         0.15779

Excited State  24:      Singlet-A      3.5431 eV  349.94 nm  f=0.0056  <S**2>=0.000
     291 -> 312        -0.11085
     296 -> 312         0.37742
     297 -> 312        -0.12285
     302 -> 313         0.16452
     307 -> 314        -0.29789
     309 -> 314         0.34993

Excited State  25:      Singlet-A      3.5465 eV  349.60 nm  f=0.0061  <S**2>=0.000
     306 -> 313         0.55740
     307 -> 313         0.15203
     310 -> 314        -0.27420

Excited State  26:      Singlet-A      3.6501 eV  339.67 nm  f=0.0810  <S**2>=0.000
     296 -> 312        -0.24615
     301 -> 312         0.11024
     301 -> 313         0.15557
     302 -> 313        -0.24731
     303 -> 313         0.27487
     308 -> 313         0.10534
     308 -> 314         0.12812
     309 -> 314         0.36316

Excited State  27:      Singlet-A      3.6684 eV  337.98 nm  f=0.0207  <S**2>=0.000
     296 -> 312         0.16050
     297 -> 313         0.11166
     300 -> 313        -0.14212
     301 -> 313         0.34206
     303 -> 313        -0.12800
     306 -> 313        -0.16161
     308 -> 314         0.35109
     309 -> 314        -0.17696
     310 -> 314        -0.14643

Excited State  28:      Singlet-A      3.6939 eV  335.65 nm  f=0.0009  <S**2>=0.000
     301 -> 313        -0.38234
     303 -> 312        -0.11090
     303 -> 314         0.11075
     308 -> 314         0.40434
     310 -> 314        -0.21783

Excited State  29:      Singlet-A      3.7099 eV  334.20 nm  f=0.1075  <S**2>=0.000
     293 -> 312         0.18964
     294 -> 312        -0.13127
     295 -> 312         0.12053
     295 -> 313         0.13227
     296 -> 312         0.23311
     299 -> 313        -0.16676
     302 -> 313        -0.21475
     303 -> 313        -0.20605
     305 -> 313        -0.12094
     306 -> 314        -0.17340
```

307 -> 314 0.29761
308 -> 313 0.10023
309 -> 314 0.13561

Excited State 30: Singlet-A 3.7749 eV 328.45 nm f=0.1871 <S**2>=0.000
292 -> 312 0.11436
293 -> 312 0.17258
293 -> 313 0.11231
295 -> 312 0.28903
296 -> 313 0.12135
298 -> 313 -0.16122
299 -> 313 0.12659
301 -> 312 0.13735
301 -> 313 -0.14100
302 -> 313 -0.20148
303 -> 313 0.16656
307 -> 314 -0.27776
309 -> 314 -0.18266

1c^{2+} (*p*-quinoid form **A**)
HOMO : 343, LUMO : 344

Excitation energies and oscillator strengths:

Excited State 1: Singlet-A 1.4092 eV 879.83 nm f=0.0498 <S**2>=0.000
340 -> 344 -0.10300
341 -> 344 0.19152
342 -> 344 -0.24657
342 -> 345 0.12630
343 -> 344 0.56234
343 -> 345 -0.17807
This state for optimization and/or second-order correction.
Total Energy, E(TD-HF/TD-DFT) = -4398.91836201
Copying the excited state density for this state as the 1-particle RhoCI density.

Excited State 2: Singlet-A 1.8531 eV 669.07 nm f=0.0023 <S**2>=0.000
340 -> 344 -0.20482
341 -> 345 0.13780
342 -> 344 0.36710
342 -> 345 -0.26849
343 -> 344 0.31476
343 -> 345 0.34793

Excited State 3: Singlet-A 1.9257 eV 643.84 nm f=0.0639 <S**2>=0.000
330 -> 344 -0.11466
338 -> 344 -0.13291
339 -> 344 0.33096
340 -> 344 0.26670
341 -> 344 -0.16738
342 -> 344 0.31816
343 -> 344 0.20674
343 -> 345 -0.25026

Excited State 4: Singlet-A 2.0680 eV 599.53 nm f=0.0908 <S**2>=0.000
338 -> 344 -0.15193
339 -> 344 0.23304
339 -> 345 -0.12348
340 -> 344 0.28159
340 -> 345 -0.19934
342 -> 344 -0.28441
343 -> 344 0.11036
343 -> 345 0.38351

Excited State 5: Singlet-A 2.3376 eV 530.39 nm f=0.0042 <S**2>=0.000
340 -> 344 0.16225
340 -> 345 -0.15476
341 -> 344 0.54756
341 -> 345 -0.26153
342 -> 344 0.19148
342 -> 345 -0.14690

```
Excited State   6:      Singlet-A      2.4399 eV  508.15 nm  f=0.0057  <S**2>=0.000
    338 -> 344       -0.16228
    338 -> 345       -0.11623
    339 -> 345        0.26776
    340 -> 344       -0.26825
    341 -> 344        0.11622
    341 -> 345       -0.13233
    342 -> 344        0.10316
    342 -> 345        0.42958
    343 -> 345        0.22740

Excited State   7:      Singlet-A      2.4987 eV  496.20 nm  f=0.0133  <S**2>=0.000
    330 -> 344       -0.10656
    338 -> 344        0.22871
    339 -> 344       -0.30343
    339 -> 345        0.17495
    340 -> 344        0.34757
    340 -> 345        0.25739
    341 -> 345       -0.16602
    343 -> 345        0.22865

Excited State   8:      Singlet-A      2.5911 eV  478.50 nm  f=0.0077  <S**2>=0.000
    330 -> 344        0.12043
    338 -> 344        0.12143
    338 -> 345        0.16601
    339 -> 344       -0.21525
    339 -> 345       -0.25443
    340 -> 345       -0.30164
    341 -> 344       -0.13023
    342 -> 344        0.22814
    342 -> 345        0.34572

Excited State   9:      Singlet-A      2.7184 eV  456.09 nm  f=0.4445  <S**2>=0.000
    330 -> 344       -0.14462
    330 -> 345       -0.12116
    331 -> 344        0.26861
    332 -> 344        0.51094
    332 -> 345        0.20037
    334 -> 344        0.14212
    335 -> 344       -0.13127

Excited State  10:      Singlet-A      2.7726 eV  447.18 nm  f=0.2488  <S**2>=0.000
    332 -> 344       -0.10747
    333 -> 344        0.30559
    333 -> 345       -0.20397
    334 -> 344        0.24155
    334 -> 345       -0.20461
    335 -> 344       -0.25588
    335 -> 345        0.22573
    336 -> 344       -0.24415
    336 -> 345        0.13483
    342 -> 345       -0.10248

Excited State  11:      Singlet-A      2.8283 eV  438.36 nm  f=0.0536  <S**2>=0.000
    330 -> 344        0.27519
    331 -> 344        0.10154
    333 -> 345       -0.11500
    334 -> 344        0.15825
    334 -> 345       -0.18318
    335 -> 344       -0.11263
    336 -> 344        0.41089
    336 -> 345       -0.12148
    337 -> 344       -0.12525
    338 -> 344       -0.16475
    339 -> 344       -0.14439
    339 -> 345        0.13478

Excited State  12:      Singlet-A      2.8785 eV  430.72 nm  f=0.0766  <S**2>=0.000
    330 -> 344        0.43329
    331 -> 344        0.10080
    333 -> 344       -0.15084
    334 -> 344       -0.20713
    335 -> 344       -0.16336
```

336 -> 344 -0.18386
336 -> 345 0.17101
337 -> 344 0.16041
339 -> 344 0.10753
340 -> 344 0.11422
340 -> 345 0.15390

Excited State 13: Singlet-A 2.9261 eV 423.71 nm f=0.0072 <S**2>=0.000
330 -> 344 0.16467
331 -> 344 0.11540
337 -> 344 -0.24686
337 -> 345 0.13519
338 -> 344 0.44716
339 -> 344 0.31717

Excited State 14: Singlet-A 2.9769 eV 416.49 nm f=0.0059 <S**2>=0.000
332 -> 344 0.22271
332 -> 345 -0.13132
333 -> 344 0.40318
333 -> 345 -0.22279
334 -> 344 -0.30209
334 -> 345 0.14444
341 -> 345 0.16216

Excited State 15: Singlet-A 2.9959 eV 413.84 nm f=0.0078 <S**2>=0.000
334 -> 344 0.11046
340 -> 344 0.12251
340 -> 345 0.18475
341 -> 344 0.27924
341 -> 345 0.52476
342 -> 345 0.17818

Excited State 16: Singlet-A 3.0795 eV 402.61 nm f=0.0074 <S**2>=0.000
331 -> 344 0.11839
335 -> 344 0.10931
336 -> 344 0.20431
337 -> 344 0.51840
337 -> 345 -0.24553
338 -> 344 0.20476

Excited State 17: Singlet-A 3.1641 eV 391.85 nm f=0.2327 <S**2>=0.000
331 -> 344 -0.16063
333 -> 344 -0.10066
334 -> 344 0.14189
341 -> 346 0.12845
343 -> 346 0.53007
343 -> 347 0.17784

Excited State 18: Singlet-A 3.1949 eV 388.07 nm f=0.0733 <S**2>=0.000
330 -> 344 -0.19003
331 -> 344 0.42010
331 -> 345 0.15268
332 -> 344 -0.23330
333 -> 345 0.10537
334 -> 344 -0.22999
335 -> 344 -0.12807
339 -> 345 0.12009
343 -> 346 0.22450

Excited State 19: Singlet-A 3.2644 eV 379.80 nm f=0.0279 <S**2>=0.000
331 -> 344 0.12992
333 -> 344 0.15157
335 -> 344 0.13210
338 -> 344 -0.20886
338 -> 345 0.18743
339 -> 345 -0.35398
340 -> 345 0.36997
341 -> 345 -0.13774
343 -> 346 0.13866

Excited State 20: Singlet-A 3.3250 eV 372.89 nm f=0.0870 <S**2>=0.000
329 -> 344 0.33292
330 -> 344 0.11296

330 -> 345 -0.17911
331 -> 344 0.18558
331 -> 345 -0.12816
332 -> 345 -0.14114
333 -> 345 0.16057
335 -> 344 0.19161
336 -> 344 -0.22896
336 -> 345 -0.14059
338 -> 344 -0.16077
340 -> 345 -0.13825
343 -> 346 -0.10422

Excited State 21: Singlet-A 3.3966 eV 365.02 nm f=0.0091 <S**2>=0.000
329 -> 344 0.14159
330 -> 345 -0.20129
331 -> 344 -0.11633
331 -> 345 -0.14459
332 -> 345 -0.14039
333 -> 345 0.18173
334 -> 345 0.12505
335 -> 344 -0.29975
335 -> 345 0.18440
336 -> 344 0.15324
336 -> 345 -0.15543
338 -> 345 0.30156

Excited State 22: Singlet-A 3.4117 eV 363.40 nm f=0.0509 <S**2>=0.000
327 -> 344 0.13034
328 -> 344 0.14600
329 -> 344 0.36783
329 -> 345 -0.10251
330 -> 345 0.34791
331 -> 345 0.13392
332 -> 345 0.21247
335 -> 344 -0.12496
335 -> 345 -0.13134
340 -> 345 0.12261

Excited State 23: Singlet-A 3.4567 eV 358.68 nm f=0.0056 <S**2>=0.000
329 -> 344 -0.16446
330 -> 345 0.11192
333 -> 345 -0.12361
335 -> 344 0.10704
336 -> 344 -0.22486
336 -> 345 -0.32791
338 -> 345 0.35277
339 -> 345 0.29821

Excited State 24: Singlet-A 3.5208 eV 352.14 nm f=0.0348 <S**2>=0.000
322 -> 344 -0.16161
324 -> 344 -0.27237
324 -> 345 -0.15070
325 -> 344 0.23060
325 -> 345 0.15589
326 -> 344 0.35215
326 -> 345 0.13043
328 -> 344 0.15091
331 -> 344 0.12691
336 -> 345 0.13850
337 -> 345 -0.12273

Excited State 25: Singlet-A 3.5303 eV 351.20 nm f=0.0107 <S**2>=0.000
324 -> 344 0.16386
327 -> 344 0.13867
327 -> 345 -0.14848
328 -> 344 -0.10461
335 -> 344 0.24607
335 -> 345 0.17608
336 -> 345 0.27255
337 -> 344 -0.16887
337 -> 345 -0.25328
338 -> 345 0.24809
339 -> 345 0.10144

```
Excited State  26:      Singlet-A      3.5651 eV  347.77 nm  f=0.0208  <S**2>=0.000
    321 -> 344       -0.17149
    321 -> 345       -0.10594
    325 -> 344        0.19898
    326 -> 344        0.21800
    326 -> 345        0.12657
    327 -> 344        0.22907
    327 -> 345       -0.21432
    328 -> 344       -0.21396
    328 -> 345        0.17158
    329 -> 344       -0.11975
    331 -> 344       -0.12715
    332 -> 344        0.11956
    333 -> 344        0.12981
    333 -> 345        0.11846
    334 -> 344       -0.13606
    334 -> 345       -0.10391
    336 -> 345       -0.10217

Excited State  27:      Singlet-A      3.5992 eV  344.48 nm  f=0.0600  <S**2>=0.000
    321 -> 344        0.23965
    321 -> 345        0.13074
    324 -> 344       -0.26650
    324 -> 345       -0.15244
    326 -> 344       -0.15284
    327 -> 344        0.16057
    327 -> 345       -0.14981
    328 -> 344       -0.10819
    328 -> 345        0.12247
    339 -> 346        0.12307
    341 -> 346       -0.13381
    342 -> 346        0.30051
    343 -> 347        0.12085

Excited State  28:      Singlet-A      3.6199 eV  342.51 nm  f=0.0391  <S**2>=0.000
    321 -> 344       -0.20378
    321 -> 345       -0.10462
    324 -> 344        0.16175
    327 -> 344       -0.10988
    327 -> 345        0.12482
    333 -> 345        0.16063
    334 -> 344        0.12736
    337 -> 345       -0.10175
    339 -> 346        0.14795
    341 -> 346       -0.16352
    342 -> 346        0.37341
    343 -> 347        0.14290

Excited State  29:      Singlet-A      3.6391 eV  340.70 nm  f=0.0282  <S**2>=0.000
    323 -> 344       -0.18964
    323 -> 345        0.21728
    324 -> 344        0.14763
    325 -> 344       -0.17069
    325 -> 345        0.12817
    327 -> 344        0.26437
    327 -> 345       -0.25217
    328 -> 344        0.23640
    328 -> 345       -0.20625
    329 -> 344       -0.10069
    332 -> 345       -0.11961
    334 -> 344        0.11251
    336 -> 345       -0.12364

Excited State  30:      Singlet-A      3.6880 eV  336.18 nm  f=0.0128  <S**2>=0.000
    329 -> 344        0.12166
    329 -> 345       -0.15797
    333 -> 344       -0.20400
    333 -> 345       -0.19775
    334 -> 345       -0.16452
    335 -> 344        0.15472
    335 -> 345        0.19340
    337 -> 344        0.18695
```

337 -> 345 0.38496
342 -> 346 0.13054

1c^{2+} (σ-bond form **B**)
HOMO : 343, LUMO : 344

Excitation energies and oscillator strengths:

Excited State 1: Singlet-A 1.9889 eV 623.39 nm f=0.0014 <S**2>=0.000
343 -> 344 0.68591
This state for optimization and/or second-order correction.
Total Energy, E(TD-HF/TD-DFT) = -4398.85644266
Copying the excited state density for this state as the 1-particle RhoCI density.

Excited State 2: Singlet-A 2.0079 eV 617.47 nm f=0.0028 <S**2>=0.000
343 -> 345 0.67188
343 -> 346 0.12158

Excited State 3: Singlet-A 2.0532 eV 603.87 nm f=0.1907 <S**2>=0.000
337 -> 344 0.10145
338 -> 344 0.47748
339 -> 345 -0.38052
340 -> 344 0.19234
341 -> 345 0.18642

Excited State 4: Singlet-A 2.1789 eV 569.03 nm f=0.4938 <S**2>=0.000
338 -> 345 0.44432
339 -> 344 -0.41440
340 -> 345 0.16533
341 -> 344 0.23595

Excited State 5: Singlet-A 2.3006 eV 538.91 nm f=0.0051 <S**2>=0.000
341 -> 345 -0.27495
342 -> 344 0.62063

Excited State 6: Singlet-A 2.3132 eV 535.99 nm f=0.0038 <S**2>=0.000
338 -> 345 0.11641
339 -> 344 -0.14073
341 -> 344 -0.28374
342 -> 345 0.59565
342 -> 346 0.11791

Excited State 7: Singlet-A 2.4814 eV 499.66 nm f=0.0243 <S**2>=0.000
330 -> 344 -0.11727
331 -> 345 -0.11708
338 -> 344 -0.29286
339 -> 345 -0.25729
340 -> 344 -0.11999
341 -> 345 0.47181
342 -> 344 0.23430

Excited State 8: Singlet-A 2.5059 eV 494.76 nm f=0.0060 <S**2>=0.000
331 -> 344 -0.10339
338 -> 345 -0.30047
339 -> 344 -0.15473
340 -> 345 -0.12578
341 -> 344 0.50866
342 -> 345 0.26591

Excited State 9: Singlet-A 2.5975 eV 477.32 nm f=0.0084 <S**2>=0.000
330 -> 345 0.13289
331 -> 344 0.12727
338 -> 345 0.24010
339 -> 344 0.45993
340 -> 345 0.20885
341 -> 344 0.29604
342 -> 345 0.20390

Excited State 10: Singlet-A 2.5993 eV 476.99 nm f=0.0250 <S**2>=0.000
330 -> 344 0.13482
331 -> 345 0.11031

338 -> 344 0.16932
339 -> 345 0.45390
340 -> 344 0.17771
341 -> 345 0.36706
342 -> 344 0.20603

Excited State 11: Singlet-A 2.8578 eV 433.85 nm f=0.5868 <S**2>=0.000
332 -> 344 -0.44129
333 -> 345 0.47709
338 -> 344 -0.11354
340 -> 344 0.15304

Excited State 12: Singlet-A 2.9101 eV 426.05 nm f=0.1104 <S**2>=0.000
332 -> 345 -0.42935
333 -> 344 0.50922
340 -> 345 0.11088

Excited State 13: Singlet-A 2.9298 eV 423.19 nm f=0.0461 <S**2>=0.000
332 -> 344 0.14382
333 -> 345 -0.12563
338 -> 344 -0.24448
340 -> 344 0.59474
343 -> 345 -0.10458

Excited State 14: Singlet-A 2.9567 eV 419.33 nm f=0.0103 <S**2>=0.000
332 -> 345 0.10808
338 -> 345 -0.26250
340 -> 345 0.59231

Excited State 15: Singlet-A 3.0972 eV 400.31 nm f=0.0117 <S**2>=0.000
334 -> 345 -0.32002
335 -> 344 -0.19464
336 -> 344 0.51446
337 -> 345 -0.27469

Excited State 16: Singlet-A 3.1005 eV 399.88 nm f=0.0007 <S**2>=0.000
334 -> 344 -0.34701
335 -> 345 -0.17544
336 -> 345 0.47037
337 -> 344 -0.32298

Excited State 17: Singlet-A 3.1942 eV 388.15 nm f=0.0014 <S**2>=0.000
334 -> 344 -0.32049
335 -> 345 -0.41774
337 -> 344 0.43065
340 -> 344 -0.10891

Excited State 18: Singlet-A 3.1970 eV 387.81 nm f=0.0001 <S**2>=0.000
334 -> 345 0.27328
335 -> 344 0.48688
336 -> 344 0.14564
337 -> 345 -0.36188
340 -> 345 0.12575

Excited State 19: Singlet-A 3.3300 eV 372.33 nm f=0.0027 <S**2>=0.000
335 -> 344 0.25742
336 -> 344 0.37349
337 -> 345 0.50077
338 -> 345 -0.11675

Excited State 20: Singlet-A 3.3308 eV 372.23 nm f=0.0004 <S**2>=0.000
335 -> 345 0.30997
336 -> 345 0.42602
337 -> 344 0.42120
338 -> 344 -0.10976
339 -> 345 0.10364

Excited State 21: Singlet-A 3.4376 eV 360.67 nm f=0.0004 <S**2>=0.000
331 -> 345 0.20631
334 -> 344 0.46742
335 -> 345 -0.36915
336 -> 345 0.22474
338 -> 344 -0.10328

Excited State 22:   Singlet-A   3.4419 eV 360.22 nm f=0.0005 <S**2>=0.000
327 -> 345   -0.11140
331 -> 344   0.33161
334 -> 345   0.44965
335 -> 344   -0.30150
336 -> 344   0.16568
338 -> 345   -0.11421
339 -> 344   -0.10242

Excited State 23:   Singlet-A   3.4673 eV 357.58 nm f=0.0229 <S**2>=0.000
327 -> 345   -0.20432
330 -> 345   -0.13430
331 -> 344   0.46037
334 -> 345   -0.30112
335 -> 344   0.19137
336 -> 344   -0.16485

Excited State 24:   Singlet-A   3.4826 eV 356.01 nm f=0.0008 <S**2>=0.000
321 -> 345   -0.10707
325 -> 344   0.11356
327 -> 344   -0.24431
331 -> 345   0.52778
333 -> 345   -0.12985
334 -> 344   -0.16823
335 -> 345   0.12764
336 -> 345   -0.11804

Excited State 25:   Singlet-A   3.5453 eV 349.72 nm f=0.0576 <S**2>=0.000
324 -> 345   -0.11283
325 -> 344   0.35902
326 -> 345   -0.35078
328 -> 344   0.23888
329 -> 345   -0.25551
330 -> 344   0.26919

Excited State 26:   Singlet-A   3.5514 eV 349.11 nm f=0.0230 <S**2>=0.000
324 -> 344   0.11401
325 -> 345   -0.38323
326 -> 344   0.40099
328 -> 345   -0.21510
329 -> 344   0.24754
330 -> 345   -0.20662

Excited State 27:   Singlet-A   3.5895 eV 345.41 nm f=0.0295 <S**2>=0.000
321 -> 345   0.14356
324 -> 345   -0.15286
327 -> 344   0.21792
328 -> 344   -0.24649
329 -> 345   0.22580
330 -> 344   0.34676
332 -> 344   -0.24553
333 -> 345   -0.16841
334 -> 344   -0.11363
335 -> 345   0.11620
338 -> 344   -0.10747
339 -> 345   -0.12276

Excited State 28:   Singlet-A   3.6206 eV 342.44 nm f=0.0538 <S**2>=0.000
321 -> 344   0.16648
324 -> 344   -0.16528
327 -> 345   0.24012
328 -> 345   -0.22392
329 -> 344   0.23838
330 -> 345   0.30688
332 -> 345   -0.26717
333 -> 344   -0.21097
338 -> 345   -0.12029
339 -> 344   -0.11196

Excited State 29:   Singlet-A   3.6344 eV 341.14 nm f=0.0003 <S**2>=0.000
343 -> 345   -0.11952
343 -> 346   0.67251

Excited State 30:   Singlet-A   3.6962 eV 335.44 nm f=0.1559 <S**2>=0.000
325 -> 344   -0.29006
326 -> 345   0.28113
328 -> 344   0.14570
329 -> 345   -0.26329
330 -> 344   0.36917
331 -> 345   0.10488
332 -> 344   0.14849

1c^{2+} (*o*-diphenoquinoid form **C**)
HOMO : 343, LUMO : 344

Excitation energies and oscillator strengths:

Excited State   1:   Singlet-A   1.2745 eV 972.83 nm f=0.2591 <S**2>=0.000
341 -> 344   0.12653
342 -> 344   -0.23433
343 -> 344   0.63525
This state for optimization and/or second-order correction.
Total Energy, E(TD-HF/TD-DFT) = -4398.91806301
Copying the excited state density for this state as the 1-particle RhoCI density.

Excited State   2:   Singlet-A   1.7251 eV 718.70 nm f=0.0710 <S**2>=0.000
341 -> 344   -0.19311
342 -> 344   0.59403
343 -> 344   0.25121

Excited State   3:   Singlet-A   1.8522 eV 669.37 nm f=0.0878 <S**2>=0.000
339 -> 344   0.30865
340 -> 344   0.33894
341 -> 344   0.44544
342 -> 344   0.18744
342 -> 345   -0.10973
343 -> 345   0.10272

Excited State   4:   Singlet-A   1.9729 eV 628.44 nm f=0.0284 <S**2>=0.000
337 -> 344   -0.15630
339 -> 344   -0.36305
340 -> 344   0.50585
343 -> 345   -0.16561

Excited State   5:   Singlet-A   2.0320 eV 610.17 nm f=0.1469 <S**2>=0.000
339 -> 344   -0.11800
339 -> 345   0.10697
341 -> 344   -0.11217
341 -> 345   0.18147
342 -> 344   -0.10036
342 -> 345   -0.24572
343 -> 345   0.56766

Excited State   6:   Singlet-A   2.2008 eV 563.36 nm f=0.0026 <S**2>=0.000
333 -> 344   0.13384
337 -> 344   -0.12656
339 -> 344   -0.37347
340 -> 344   -0.26923
341 -> 344   0.45294

Excited State   7:   Singlet-A   2.5906 eV 478.59 nm f=0.2498 <S**2>=0.000
332 -> 344   -0.32253
333 -> 344   0.11218
334 -> 344   0.25718
334 -> 345   0.13859
336 -> 344   0.41905
336 -> 345   0.13291
339 -> 344   0.13328

Excited State   8:   Singlet-A   2.6263 eV 472.09 nm f=0.0417 <S**2>=0.000
333 -> 344   0.11886
334 -> 344   -0.13001
336 -> 344   -0.15734

337 -> 345 -0.10129
340 -> 345 -0.15748
341 -> 345 -0.22687
342 -> 345 0.45116
343 -> 345 0.30723

Excited State 9: Singlet-A 2.6513 eV 467.64 nm f=0.0237 <S**2>=0.000
333 -> 344 -0.13757
335 -> 344 0.12823
336 -> 344 0.10972
337 -> 344 0.46590
338 -> 344 -0.10924
339 -> 344 -0.17954
339 -> 345 0.17903
340 -> 345 0.20548
341 -> 345 0.17684
342 -> 344 0.11064
342 -> 345 0.15416

Excited State 10: Singlet-A 2.7540 eV 450.20 nm f=0.0734 <S**2>=0.000
329 -> 344 -0.10103
332 -> 344 0.40497
336 -> 344 0.33098
337 -> 344 -0.22900
338 -> 344 -0.15653
339 -> 345 0.12960
342 -> 345 0.23385

Excited State 11: Singlet-A 2.7619 eV 448.91 nm f=0.1481 <S**2>=0.000
331 -> 344 -0.14136
332 -> 344 0.15458
332 -> 345 -0.12185
333 -> 344 -0.23552
334 -> 344 0.39442
335 -> 344 -0.26660
338 -> 344 0.25077
339 -> 344 -0.10309
342 -> 345 0.16177

Excited State 12: Singlet-A 2.8161 eV 440.27 nm f=0.0293 <S**2>=0.000
330 -> 344 0.19792
333 -> 344 0.42291
333 -> 345 -0.13597
334 -> 344 0.17862
335 -> 344 -0.13325
336 -> 344 -0.13092
338 -> 344 0.12723
339 -> 344 0.13525
339 -> 345 0.12093
340 -> 345 0.12548
341 -> 345 0.21258
342 -> 345 0.13867
343 -> 346 -0.14491

Excited State 13: Singlet-A 2.8443 eV 435.90 nm f=0.0393 <S**2>=0.000
332 -> 344 0.30429
333 -> 344 0.30893
337 -> 344 0.22882
338 -> 344 -0.10420
339 -> 345 -0.15401
341 -> 345 -0.26062
342 -> 345 -0.22328
343 -> 346 0.18668

Excited State 14: Singlet-A 2.8868 eV 429.48 nm f=0.0712 <S**2>=0.000
330 -> 344 -0.25902
331 -> 344 0.31240
332 -> 345 -0.12267
334 -> 344 0.17385
335 -> 344 -0.18746
336 -> 344 -0.22275
338 -> 344 -0.23661
339 -> 345 -0.14493

340 -> 345 0.28523

Excited State 15: Singlet-A 2.9074 eV 426.44 nm f=0.0098 <S**2>=0.000
331 -> 344 -0.27574
335 -> 344 0.10812
337 -> 345 -0.10532
338 -> 344 0.17623
339 -> 345 -0.26069
340 -> 345 0.42191
343 -> 346 -0.20619

Excited State 16: Singlet-A 2.9595 eV 418.93 nm f=0.0943 <S**2>=0.000
337 -> 344 -0.16463
338 -> 344 0.10796
340 -> 345 0.15519
341 -> 345 0.18574
341 -> 346 0.13473
342 -> 346 -0.21716
343 -> 346 0.52571

Excited State 17: Singlet-A 3.0142 eV 411.33 nm f=0.0041 <S**2>=0.000
331 -> 344 0.40246
338 -> 344 0.41023
339 -> 345 0.13246
340 -> 345 0.13191
341 -> 345 -0.24982

Excited State 18: Singlet-A 3.0496 eV 406.56 nm f=0.0051 <S**2>=0.000
330 -> 344 -0.30983
331 -> 344 0.12964
333 -> 344 0.15604
335 -> 344 0.15224
336 -> 344 0.12224
338 -> 344 0.26680
339 -> 345 -0.19652
340 -> 345 -0.18961
341 -> 345 0.31381

Excited State 19: Singlet-A 3.1698 eV 391.14 nm f=0.0415 <S**2>=0.000
330 -> 344 0.32668
331 -> 344 0.12386
334 -> 344 -0.18102
335 -> 344 -0.32171
336 -> 344 0.10059
337 -> 344 0.10284
339 -> 345 -0.32187
340 -> 345 -0.10427
341 -> 345 0.15177

Excited State 20: Singlet-A 3.2329 eV 383.51 nm f=0.0316 <S**2>=0.000
330 -> 344 0.30255
331 -> 344 0.16336
334 -> 344 0.25851
335 -> 344 0.42851
336 -> 344 -0.16756
337 -> 344 -0.13418
339 -> 345 -0.14635

Excited State 21: Singlet-A 3.2583 eV 380.52 nm f=0.0143 <S**2>=0.000
325 -> 344 0.11009
329 -> 344 0.58614
331 -> 344 0.13916

Excited State 22: Singlet-A 3.3432 eV 370.85 nm f=0.0880 <S**2>=0.000
327 -> 344 -0.24872
327 -> 345 0.10447
328 -> 344 0.50335
328 -> 345 -0.21300
330 -> 345 -0.10470
331 -> 345 0.11423
342 -> 346 0.14989

Excited State 23: Singlet-A 3.4268 eV 361.81 nm f=0.0403 <S**2>=0.000

327 -> 344 0.13560
328 -> 344 -0.12969
342 -> 346 0.52223
343 -> 346 0.21387

Excited State 24: Singlet-A 3.4617 eV 358.16 nm f=0.0407 <S**2>=0.000
323 -> 345 -0.11473
325 -> 344 0.31840
326 -> 344 0.13185
327 -> 344 -0.12084
332 -> 345 -0.12338
334 -> 344 -0.13772
334 -> 345 0.20533
336 -> 345 0.37799
339 -> 346 0.12091
342 -> 346 0.10648

Excited State 25: Singlet-A 3.4794 eV 356.34 nm f=0.0403 <S**2>=0.000
323 -> 344 -0.18546
323 -> 345 -0.11129
324 -> 344 -0.12142
325 -> 344 0.42200
325 -> 345 0.16571
326 -> 344 -0.21323
329 -> 345 0.10652
334 -> 345 -0.19264
336 -> 345 -0.16189
337 -> 345 0.13175

Excited State 26: Singlet-A 3.5318 eV 351.05 nm f=0.0637 <S**2>=0.000
324 -> 344 -0.11978
325 -> 344 -0.11407
326 -> 344 -0.20084
328 -> 344 0.11263
330 -> 345 0.15441
331 -> 345 -0.10477
332 -> 345 0.22491
333 -> 345 -0.11462
336 -> 345 0.13265
339 -> 346 0.21439
340 -> 346 0.21582
341 -> 346 0.32451

Excited State 27: Singlet-A 3.5569 eV 348.57 nm f=0.0182 <S**2>=0.000
323 -> 344 -0.10209
324 -> 344 0.39851
324 -> 345 0.24229
326 -> 344 0.22204
333 -> 345 -0.10221
336 -> 345 -0.13633
337 -> 345 0.21717
340 -> 346 0.13426
341 -> 346 0.18671

Excited State 28: Singlet-A 3.6198 eV 342.52 nm f=0.0881 <S**2>=0.000
323 -> 344 -0.31153
325 -> 345 0.11035
327 -> 344 0.40768
327 -> 345 -0.16551
328 -> 344 0.23579
330 -> 345 -0.14206
336 -> 345 0.11376

Excited State 29: Singlet-A 3.6234 eV 342.18 nm f=0.0454 <S**2>=0.000
323 -> 344 0.22461
324 -> 344 -0.15261
324 -> 345 -0.12414
325 -> 344 0.20746
325 -> 345 -0.11350
326 -> 344 0.29454
327 -> 344 0.12740
328 -> 344 0.12028
332 -> 345 0.11990

333 -> 345 -0.10951
335 -> 345 -0.16374
336 -> 345 -0.25882
338 -> 345 0.10775
339 -> 346 0.11444

Excited State 30: Singlet-A 3.6427 eV 340.37 nm f=0.0099 <S**2>=0.000
323 -> 344 -0.20679
324 -> 344 0.22870
324 -> 345 0.15394
326 -> 344 -0.14450
331 -> 345 -0.15332
332 -> 344 0.11334
332 -> 345 0.18541
335 -> 345 -0.17198
337 -> 345 -0.16705
338 -> 345 0.20000
340 -> 346 -0.20300
342 -> 346 0.16965

1c-2H^{+} (folded form)
αHOMO : 345, αLUMO : 346
βHOMO : 344, βLUMO : 345

Excitation energies and oscillator strengths:

Excited State 1: 2.189-A 1.2718 eV 974.84 nm f=0.0714 <S**2>=0.948
335A -> 346A -0.10375
345A -> 346A 0.94538
345A <- 346A 0.10768
This state for optimization and/or second-order correction.
Total Energy, E(TD-HF/TD-DFT) = -4400.43697236
Copying the excited state density for this state as the 1-particle RhoCI density.

Excited State 2: 2.148-A 1.3680 eV 906.35 nm f=0.0425 <S**2>=0.903
325B -> 345B 0.12000
336B -> 345B -0.19117
337B -> 345B -0.21886
338B -> 345B -0.17004
341B -> 345B -0.15190
342B -> 345B -0.11515
343B -> 345B 0.87320

Excited State 3: 2.170-A 1.4747 eV 840.75 nm f=0.0232 <S**2>=0.928
326B -> 345B -0.10743
337B -> 345B -0.11212
338B -> 345B 0.17298
339B -> 345B -0.10427
342B -> 345B -0.13346
343B -> 346B 0.11905
344B -> 345B 0.90160

Excited State 4: 2.450-A 1.9622 eV 631.87 nm f=0.0323 <S**2>=1.250
335A -> 346A 0.13056
337A -> 346A 0.19880
342A -> 346A -0.20858
345A -> 348A -0.11938
326B -> 345B -0.16144
327B -> 345B 0.15892
329B -> 345B -0.19410
334B -> 345B 0.32641
335B -> 345B -0.28145
337B -> 345B 0.10331
338B -> 345B -0.11401
339B -> 345B -0.17838
340B -> 345B -0.26381
342B -> 345B 0.54731
343B -> 346B 0.21496

Excited State 5: 3.006-A 2.0572 eV 602.67 nm f=0.0009 <S**2>=2.010
323A -> 346A -0.10378
326A -> 346A 0.12338
327A -> 346A 0.10225

338A -> 346A 0.16982
339A -> 346A -0.11114
344A -> 346A 0.58815
345A -> 347A -0.16172
325B -> 345B 0.19086
326B -> 346B 0.13650
332B -> 345B -0.10266
336B -> 345B 0.42726
338B -> 346B -0.10830
343B -> 345B 0.21417
344B -> 346B -0.29760

Excited State 6: 2.421-A 2.1520 eV 576.12 nm f=0.0008 <S**2>=1.215
335A -> 346A -0.11197
337A -> 346A -0.19491
342A -> 346A 0.21176
345A -> 348A 0.10123
326B -> 345B 0.12853
329B -> 345B 0.10539
334B -> 345B -0.21256
335B -> 345B 0.17208
341B -> 346B 0.12396
342B -> 345B 0.75873
343B -> 345B 0.13096
343B -> 346B -0.17006
344B -> 345B 0.18814

Excited State 7: 2.137-A 2.2768 eV 544.57 nm f=0.0094 <S**2>=0.892
344A -> 346A -0.10284
336B -> 345B 0.25463
338B -> 345B 0.14284
339B -> 345B 0.36823
340B -> 345B -0.32847
341B -> 345B 0.74210
343B -> 345B 0.22740

Excited State 8: 2.405-A 2.3406 eV 529.70 nm f=0.0248 <S**2>=1.197
344A -> 346A -0.45961
336B -> 345B 0.46873
337B -> 345B 0.44211
338B -> 345B 0.26646
339B -> 345B -0.10351
340B -> 345B 0.33776
341B -> 345B -0.13346
343B -> 345B 0.17580

Excited State 9: 2.367-A 2.3660 eV 524.03 nm f=0.0321 <S**2>=1.151
337A -> 346A 0.14648
342A -> 346A -0.17929
343A -> 346A -0.10536
344A -> 346A 0.19067
336B -> 345B -0.24838
337B -> 345B -0.13570
340B -> 345B 0.66155
341B -> 345B 0.38995
342B -> 345B 0.16454
343B -> 346B 0.17217
344B -> 345B -0.13102

Excited State 10: 2.205-A 2.3781 eV 521.35 nm f=0.0070 <S**2>=0.965
326B -> 345B -0.18482
327B -> 345B -0.14958
334B -> 345B -0.16568
335B -> 345B 0.13217
336B -> 346B -0.11677
337B -> 345B -0.34387
337B -> 346B -0.14995
338B -> 345B 0.59062
338B -> 346B -0.11352
339B -> 345B -0.33842
340B -> 345B -0.29757
341B -> 345B -0.10541
344B -> 345B -0.28513

Excited State 11: 2.342-A   2.4067 eV 515.16 nm f=0.0348 <S**2>=1.121
337A -> 346A   0.15594
342A -> 346A   -0.20650
334B -> 345B   -0.14148
337B -> 345B   -0.22726
339B -> 345B   0.70211
341B -> 345B   -0.41166
342B -> 345B   0.10626
343B -> 346B   0.17680

Excited State 12: 2.149-A   2.4853 eV 498.88 nm f=0.0225 <S**2>=0.905
344A -> 346A   0.24266
332B -> 345B   0.13119
336B -> 345B   -0.47054
337B -> 345B   0.58153
338B -> 345B   0.42768
339B -> 345B   0.20423
340B -> 345B   -0.11038
343B -> 345B   0.15640
344B -> 346B   0.16008

Excited State 13: 2.267-A   2.5758 eV 481.35 nm f=0.1134 <S**2>=1.035
337A -> 346A   -0.10410
342A -> 346A   0.15579
327B -> 345B   0.13839
328B -> 345B   0.15369
329B -> 345B   -0.15821
334B -> 345B   0.44788
335B -> 345B   -0.38422
336B -> 346B   0.10113
337B -> 345B   -0.32615
338B -> 345B   0.41872
339B -> 345B   0.17584
340B -> 345B   0.30479
343B -> 346B   -0.13451

Excited State 14: 2.499-A   2.6815 eV 462.36 nm f=0.0355 <S**2>=1.311
325A -> 346A   0.10464
335A -> 346A   0.20489
337A -> 346A   -0.28336
340A -> 346A   -0.16515
341A -> 346A   -0.20422
342A -> 346A   0.45808
343A -> 346A   0.18502
326B -> 345B   -0.40812
328B -> 345B   -0.11781
331B -> 345B   -0.11735
336B -> 346B   -0.24002
338B -> 345B   -0.13235
339B -> 345B   0.14740
343B -> 346B   0.32125

Excited State 15: 2.186-A   2.7490 eV 451.02 nm f=0.0009 <S**2>=0.945
344A -> 346A   0.25845
325B -> 345B   -0.28522
329B -> 345B   -0.12062
330B -> 345B   -0.33539
331B -> 345B   0.23110
332B -> 345B   0.56377
334B -> 345B   -0.10305
335B -> 345B   -0.12514
336B -> 345B   0.27366
338B -> 346B   0.11467
339B -> 345B   -0.14837
340B -> 345B   0.10429
344B -> 346B   0.29492

Excited State 16: 2.613-A   2.8144 eV 440.53 nm f=0.0103 <S**2>=1.457
326A -> 348A   -0.10010
327A -> 346A   -0.24020
333A -> 346A   0.16915
344A -> 346A   0.36342

345A -> 347A 0.18887
345A -> 349A 0.14140
324B -> 345B -0.19472
330B -> 345B 0.16061
331B -> 345B -0.17256
332B -> 345B -0.33216
334B -> 346B -0.17989
335B -> 346B 0.14530
336B -> 345B 0.14992
341B -> 345B -0.12028
342B -> 346B -0.10442
344B -> 346B 0.41429
344B -> 348B -0.10319

Excited State 17: 2.248-A 2.9217 eV 424.35 nm f=0.0055 <S**2>=1.014
326B -> 345B 0.29747
327B -> 345B 0.45058
328B -> 345B -0.44684
329B -> 345B -0.25629
330B -> 345B -0.27257
331B -> 345B -0.27959
332B -> 345B -0.13160
333B -> 345B 0.28822
336B -> 346B 0.10519
338B -> 345B 0.12033

Excited State 18: 2.947-A 2.9462 eV 420.83 nm f=0.0006 <S**2>=1.921
327A -> 346A -0.30403
333A -> 346A 0.19225
338A -> 346A -0.13081
340A -> 346A 0.17136
342A -> 346A -0.15429
343A -> 346A 0.44271
345A -> 349A 0.16985
324B -> 345B -0.10716
325B -> 345B -0.15749
330B -> 345B -0.16569
332B -> 345B 0.19149
334B -> 346B -0.13816
335B -> 346B 0.11522
336B -> 345B -0.11903
343B -> 345B 0.10803
343B -> 347B -0.11199
344B -> 346B -0.41527

Excited State 19: 2.440-A 2.9739 eV 416.91 nm f=0.0237 <S**2>=1.238
342A -> 346A 0.15607
344A -> 347A 0.10257
325B -> 346B 0.10240
326B -> 345B 0.43270
328B -> 345B 0.28717
329B -> 345B 0.14801
331B -> 345B 0.29753
333B -> 345B -0.17406
336B -> 346B 0.10418
337B -> 346B -0.14022
343B -> 346B 0.55491
343B -> 348B -0.10553
344B -> 347B 0.13470

Excited State 20: 2.497-A 3.0603 eV 405.13 nm f=0.0000 <S**2>=1.309
342A -> 346A -0.17312
343A -> 346A 0.53507
324B -> 345B 0.13886
333B -> 345B 0.11034
334B -> 345B 0.44177
334B -> 346B 0.10533
335B -> 345B 0.56428
344B -> 346B 0.12367

Excited State 21: 2.522-A 3.0901 eV 401.23 nm f=0.0016 <S**2>=1.340
327A -> 346A 0.13171
333A -> 346A -0.12120

342A -> 346A -0.17002
343A -> 346A 0.53326
324B -> 345B 0.12391
325B -> 345B 0.24725
332B -> 345B -0.13972
334B -> 345B -0.40281
335B -> 345B -0.48665
344B -> 346B 0.16677

Excited State 22: 2.539-A 3.1315 eV 395.93 nm f=0.0050 <S**2>=1.361
327A -> 346A -0.11674
338A -> 346A -0.37447
339A -> 346A 0.26673
344A -> 346A 0.15540
345A -> 347A 0.10588
325B -> 345B 0.62788
330B -> 345B -0.19494
332B -> 345B 0.29561
333B -> 345B -0.16097

Excited State 23: 2.938-A 3.1531 eV 393.22 nm f=0.0288 <S**2>=1.908
335A -> 346A 0.34788
337A -> 346A 0.50790
339A -> 346A 0.10508
341A -> 346A -0.26738
342A -> 346A 0.30893
345A -> 346A 0.12759
326B -> 345B 0.24035
331B -> 345B 0.16966
336B -> 346B -0.10307
343B -> 346B -0.30039

Excited State 24: 2.743-A 3.1698 eV 391.14 nm f=0.0014 <S**2>=1.631
326A -> 346A 0.10574
338A -> 346A 0.51256
339A -> 346A -0.47223
340A -> 346A 0.10693
341A -> 346A -0.12368
344A -> 346A -0.19310
324B -> 345B -0.12214
325B -> 345B 0.33768
330B -> 345B -0.16505
331B -> 345B -0.11300
332B -> 345B 0.20041
333B -> 345B -0.23202
344B -> 346B 0.12267

Excited State 25: 2.822-A 3.1898 eV 388.68 nm f=0.0084 <S**2>=1.741
335A -> 346A -0.17511
337A -> 346A 0.27787
338A -> 346A 0.26092
339A -> 346A 0.21959
340A -> 346A 0.31224
342A -> 346A 0.40341
344A -> 347A -0.12608
326B -> 345B -0.10134
328B -> 345B 0.27524
329B -> 345B -0.15954
331B -> 345B -0.24287
332B -> 345B 0.10085
334B -> 345B -0.18962
335B -> 345B 0.13619
343B -> 346B 0.17902

Excited State 26: 2.325-A 3.2167 eV 385.44 nm f=0.0008 <S**2>=1.101
335A -> 346A 0.14259
338A -> 346A -0.11997
339A -> 346A -0.19754
340A -> 346A -0.23328
342A -> 346A -0.18613
327B -> 345B 0.18512
328B -> 345B 0.67070
329B -> 345B -0.29131

331B -> 345B -0.23195
333B -> 345B 0.16444
334B -> 345B -0.19774
335B -> 345B 0.12510
343B -> 346B -0.13060

Excited State 27: 2.978-A 3.2456 eV 382.01 nm f=0.0014 <S**2>=1.967
325A -> 346A 0.11175
335A -> 346A -0.28389
337A -> 346A 0.51970
338A -> 346A -0.10941
339A -> 346A -0.30422
340A -> 346A -0.42194
323B -> 345B 0.10868
326B -> 345B -0.17766
327B -> 345B -0.11296
328B -> 345B -0.10562
331B -> 345B -0.11690

Excited State 28: 2.302-A 3.2883 eV 377.05 nm f=0.0162 <S**2>=1.075
327A -> 346A -0.14743
339A -> 346A -0.14885
340A -> 346A 0.20905
341A -> 346A -0.18928
343A -> 346A -0.12480
345A -> 347A 0.10954
324B -> 345B 0.14012
325B -> 345B 0.24684
330B -> 345B 0.14602
331B -> 345B 0.29779
333B -> 345B 0.72895
335B -> 345B -0.12426

Excited State 29: 3.029-A 3.3118 eV 374.37 nm f=0.0216 <S**2>=2.043
325A -> 346A 0.13761
330A -> 346A -0.10235
332A -> 346A -0.12455
340A -> 346A 0.15547
341A -> 346A 0.60514
342A -> 346A 0.24544
343A -> 346A 0.13061
344A -> 347A 0.20972
325B -> 346B -0.11515
328B -> 345B 0.10356
329B -> 345B 0.11330
331B -> 345B -0.10088
333B -> 345B 0.15510
343B -> 346B -0.17950
344B -> 347B 0.20134

Excited State 30: 2.388-A 3.3458 eV 370.56 nm f=0.0083 <S**2>=1.176
327A -> 346A 0.11109
339A -> 346A 0.18221
340A -> 346A -0.20864
324B -> 345B -0.17371
326B -> 345B 0.20685
327B -> 345B -0.22264
329B -> 345B 0.46052
330B -> 345B -0.17513
331B -> 345B -0.42458
332B -> 345B 0.10471
333B -> 345B 0.30700
334B -> 345B 0.18955
335B -> 345B -0.11227

1c-2H^{2+} (twisted form)
HOMO : 344, LUMO : 345

Excitation energies and oscillator strengths:

Excited State 1: Singlet-A 1.8545 eV 668.56 nm f=0.1116 <S**2>=0.000
340 -> 345 0.44107

341 -> 345 0.31067
343 -> 345 -0.34773
344 -> 345 0.22644
This state for optimization and/or second-order correction.
Total Energy, E(TD-HF/TD-DFT) = -4400.13353075
Copying the excited state density for this state as the 1-particle RhoCI density.

Excited State 2: Singlet-A 2.0266 eV 611.80 nm f=0.0987 <S**2>=0.000
339 -> 345 0.48868
340 -> 345 -0.10447
340 -> 346 -0.26186
341 -> 345 -0.12376
341 -> 346 -0.17659
343 -> 346 0.20717
344 -> 345 0.23663

Excited State 3: Singlet-A 2.1884 eV 566.56 nm f=0.0160 <S**2>=0.000
339 -> 345 -0.14943
340 -> 345 -0.14410
340 -> 346 0.20174
341 -> 345 -0.20511
341 -> 346 0.17425
344 -> 345 0.56235

Excited State 4: Singlet-A 2.2844 eV 542.74 nm f=0.0309 <S**2>=0.000
339 -> 345 0.29926
340 -> 345 0.14380
340 -> 346 0.22205
341 -> 345 0.16462
341 -> 346 0.12105
342 -> 345 -0.21104
343 -> 345 0.33154
343 -> 346 -0.23585
344 -> 346 0.25155

Excited State 5: Singlet-A 2.3215 eV 534.07 nm f=0.0796 <S**2>=0.000
339 -> 345 0.34500
339 -> 346 -0.14788
340 -> 346 0.23259
341 -> 345 -0.12565
341 -> 346 0.21385
342 -> 345 0.22422
343 -> 345 -0.32102
343 -> 346 -0.16362
344 -> 345 -0.17779

Excited State 6: Singlet-A 2.4137 eV 513.66 nm f=0.0095 <S**2>=0.000
339 -> 346 0.42999
342 -> 345 -0.30006
343 -> 345 -0.35592
344 -> 346 0.22527

Excited State 7: Singlet-A 2.4771 eV 500.52 nm f=0.0038 <S**2>=0.000
339 -> 346 0.42484
340 -> 345 0.18464
342 -> 345 0.49115
343 -> 345 0.10326

Excited State 8: Singlet-A 2.4964 eV 496.64 nm f=0.0058 <S**2>=0.000
340 -> 345 -0.42453
341 -> 345 0.51336
342 -> 345 0.13019

Excited State 9: Singlet-A 2.6411 eV 469.44 nm f=0.0351 <S**2>=0.000
333 -> 345 0.11313
337 -> 346 0.12013
339 -> 346 -0.21456
342 -> 345 0.18325
342 -> 346 -0.13662
343 -> 346 0.12328
344 -> 346 0.54455

Excited State 10: Singlet-A 2.6879 eV 461.28 nm f=0.3459 <S**2>=0.000

333 -> 346 0.13436
334 -> 345 0.11463
335 -> 345 -0.32582
335 -> 346 -0.14623
336 -> 345 -0.11571
337 -> 345 0.35911
338 -> 345 0.31672
339 -> 346 0.14287
343 -> 346 -0.13459
344 -> 346 -0.11886

Excited State 11: Singlet-A 2.7268 eV 454.68 nm f=0.1613 <S**2>=0.000
333 -> 345 0.37927
334 -> 345 -0.24739
334 -> 346 0.15253
335 -> 346 -0.20049
337 -> 345 -0.16871
337 -> 346 0.24868
338 -> 345 -0.13118
338 -> 346 0.19930
344 -> 346 -0.15350

Excited State 12: Singlet-A 2.7742 eV 446.91 nm f=0.0070 <S**2>=0.000
340 -> 346 0.29062
341 -> 346 0.19515
342 -> 346 -0.15495
343 -> 346 0.54398

Excited State 13: Singlet-A 2.8549 eV 434.29 nm f=0.0006 <S**2>=0.000
341 -> 346 0.13537
342 -> 346 0.63946
343 -> 346 0.16111
344 -> 346 0.13725

Excited State 14: Singlet-A 2.9017 eV 427.28 nm f=0.0093 <S**2>=0.000
340 -> 346 -0.40935
341 -> 346 0.52887
342 -> 346 -0.11172

Excited State 15: Singlet-A 3.0063 eV 412.41 nm f=0.1254 <S**2>=0.000
332 -> 345 0.57876
333 -> 345 -0.10722
335 -> 345 -0.19045
336 -> 345 0.13152
337 -> 345 -0.14828

Excited State 16: Singlet-A 3.0623 eV 404.87 nm f=0.1014 <S**2>=0.000
333 -> 345 0.31908
333 -> 346 -0.21016
334 -> 345 -0.16395
335 -> 345 -0.21081
335 -> 346 0.30268
336 -> 345 -0.12967
336 -> 346 0.14330
337 -> 346 -0.24034
338 -> 345 0.15015
338 -> 346 -0.21375

Excited State 17: Singlet-A 3.1010 eV 399.82 nm f=0.0007 <S**2>=0.000
335 -> 345 0.27942
335 -> 346 -0.12191
337 -> 345 -0.28583
338 -> 345 0.53816

Excited State 18: Singlet-A 3.1805 eV 389.82 nm f=0.0254 <S**2>=0.000
331 -> 345 -0.17361
332 -> 345 -0.23217
332 -> 346 -0.13423
335 -> 345 -0.17482
335 -> 346 0.10291
336 -> 345 0.50446
338 -> 346 0.16182

Excited State 19: Singlet-A 3.2622 eV 380.06 nm f=0.0580 <S**2>=0.000
331 -> 345 0.25320
332 -> 346 0.29001
333 -> 346 0.25773
334 -> 346 -0.21845
335 -> 345 -0.19953
336 -> 345 0.11339
337 -> 345 -0.23997
337 -> 346 -0.22408

Excited State 20: Singlet-A 3.3167 eV 373.81 nm f=0.0377 <S**2>=0.000
331 -> 345 -0.20697
332 -> 346 -0.34427
333 -> 345 0.14808
333 -> 346 0.37950
334 -> 346 -0.17959
337 -> 345 -0.20347
338 -> 345 -0.10103
338 -> 346 -0.12645

Excited State 21: Singlet-A 3.3429 eV 370.89 nm f=0.0125 <S**2>=0.000
332 -> 345 -0.10466
332 -> 346 0.11303
333 -> 345 -0.10415
333 -> 346 -0.17747
334 -> 345 0.38450
335 -> 345 -0.29192
336 -> 345 -0.17758
337 -> 345 -0.30364
338 -> 345 -0.10461

Excited State 22: Singlet-A 3.3794 eV 366.88 nm f=0.0078 <S**2>=0.000
331 -> 345 -0.20912
332 -> 345 0.13873
332 -> 346 0.25827
333 -> 345 0.34447
334 -> 345 0.31097
335 -> 345 0.13921
336 -> 345 0.26171
338 -> 346 -0.11506

Excited State 23: Singlet-A 3.4137 eV 363.20 nm f=0.0296 <S**2>=0.000
330 -> 345 -0.13061
331 -> 345 0.45629
331 -> 346 -0.15279
332 -> 346 -0.22730
333 -> 345 0.16522
334 -> 345 0.27004
336 -> 345 0.12558

Excited State 24: Singlet-A 3.4758 eV 356.70 nm f=0.0176 <S**2>=0.000
326 -> 345 -0.11780
326 -> 346 -0.15010
327 -> 345 0.26443
328 -> 345 0.14066
329 -> 346 0.14388
331 -> 345 0.10477
331 -> 346 0.35054
336 -> 345 0.13790
336 -> 346 -0.19437
337 -> 346 0.18992
338 -> 346 -0.16206

Excited State 25: Singlet-A 3.5302 eV 351.21 nm f=0.0072 <S**2>=0.000
326 -> 345 0.17630
326 -> 346 -0.11860
327 -> 345 0.34664
327 -> 346 -0.17342
328 -> 345 0.17235
330 -> 345 -0.26083
330 -> 346 0.12226
336 -> 346 0.12475
337 -> 345 0.10411

337 -> 346 -0.12644
338 -> 346 0.27444
340 -> 347 -0.11458

Excited State 26: Singlet-A 3.5761 eV 346.71 nm f=0.0165 <S**2>=0.000
325 -> 345 0.10234
330 -> 345 0.32205
330 -> 346 -0.11122
331 -> 346 0.19905
332 -> 346 -0.15573
334 -> 345 0.13153
337 -> 346 -0.22041
338 -> 346 0.39660

Excited State 27: Singlet-A 3.5819 eV 346.14 nm f=0.0400 <S**2>=0.000
328 -> 345 0.24176
328 -> 346 0.19380
330 -> 345 -0.11942
331 -> 346 -0.17736
334 -> 346 -0.10950
340 -> 347 0.34248
341 -> 347 0.23399
343 -> 347 -0.24282
344 -> 347 0.13909

Excited State 28: Singlet-A 3.6061 eV 343.82 nm f=0.0043 <S**2>=0.000
324 -> 346 -0.11385
325 -> 345 -0.10123
325 -> 346 -0.20668
328 -> 345 0.33482
328 -> 346 0.15044
329 -> 345 -0.27851
329 -> 346 -0.14505
330 -> 345 0.20226
340 -> 347 -0.19117
341 -> 347 -0.13083
343 -> 347 0.13932

Excited State 29: Singlet-A 3.6490 eV 339.78 nm f=0.0002 <S**2>=0.000
326 -> 345 -0.21724
327 -> 345 -0.10471
327 -> 346 0.10035
328 -> 346 0.17109
329 -> 345 0.28451
330 -> 345 -0.17163
331 -> 345 -0.16494
335 -> 346 0.15907
336 -> 346 -0.23631
338 -> 346 0.17857
340 -> 347 -0.17520
341 -> 347 -0.11990
343 -> 347 0.13339

Excited State 30: Singlet-A 3.7132 eV 333.91 nm f=0.0171 <S**2>=0.000
326 -> 345 -0.13773
328 -> 345 0.17743
329 -> 345 0.21258
329 -> 346 0.17752
332 -> 346 -0.13341
335 -> 346 -0.14887
336 -> 346 0.47580

References

1. Harimoto, T., Tadokoro, T., Sugiyama, S., Suzuki, T. & Ishigaki, Y. Domino-Redox Reaction Induced by An Electrochemically Triggered Conformational Change. *Angew. Chem. Int. Ed.* **63**, e202316753 (2024).
2. Neidlein, R. & Winter, M. Synthesis of Geminal Enediynes with Saturated and Unsaturated Carbocyclic Backbones by Palladium-Catalyzed Alkynylation of Dibromoolefins. *Synthesis* **1998**, 1362–1366 (1998).
3. Harimoto, T., Suzuki, T. & Ishigaki, Y. Enhancement of NIR-Absorbing Ability of Bis(diarylmethylium)-Type Dicationic Dyes Based on an Ortho -Substitution Strategy. *Chem. Eur. J.* **29**, e202203899 (2023).
4. Ishigaki, Y., Fukagawa, R., Sugawara, K., Harimoto, T. & Suzuki, T. Geometrical and Electronic Structure of Cation Radical Species of Tetraarylanthraquinodimethane: An Intermediate for Unique Electrochromic Behavior. *Chem. Asian J.* **17**, e202200914 (2022).
5. Roos, B. O., Taylor, P. R. & Sigbahn, P. E. M. A complete active space SCF method (CASSCF) using a density matrix formulated super-CI approach. *Chem. Phys.* **48**, 157–173 (1980).
6. Neese, F. Software update: The ORCA program system—Version 5.0. *WIREs Comput. Mol. Sci.* **12**, e1606 (2022).
7. Ishigaki, Y. *et al.* Redox-active tetraaryldibenzoquinodimethanes. *Chem. Commun.* **57**, 7201–7214 (2021).
8. Ishigaki, Y., Harimoto, T., Sugawara, K. & Suzuki, T. Hysteretic Three-State Redox Interconversion among Zigzag Bisquinodimethanes with Non-fused Benzene Rings and Twisted Tetra-/Dications with [5]/[3]Acenes Exhibiting Near-Infrared Absorptions. *J. Am. Chem. Soc.* **143**, 3306–3311 (2021).
9. Harimoto, T., Sugai, Y., Sugawara, K., Suzuki, T. & Ishigaki, Y. Double Dynamic Structural Change Enabling Tricolor Chromism by the Realization of Apparent Two-Electron Transfer to Skip the Open-Shell State. *Chem. Eur. J.* **29**, e202301476 (2023).
10. Herges, R. & Geuenich, D. Delocalization of Electrons in Molecules. *J. Phys. Chem. A* **105**, 3214–3220 (2001).
11. Geuenich, D., Hess, K., Köhler, F. & Herges, R. Anisotropy of the Induced Current Density (ACID), a General Method To Quantify and Visualize Electronic Delocalization. *Chem. Rev.* **105**, 3758–3772 (2005).
12. Schleyer, P. von R., Maerker, C., Dransfeld, A., Jiao, H. & van Eikema Hommes, N. J. R. Nucleus-Independent Chemical Shifts: A Simple and Efficient Aromaticity Probe. *J. Am. Chem. Soc.* **118**, 6317–6318 (1996).
13. Chen, Z., Wannere, C. S., Corminboeuf, C., Puchta, R. & von Ragué Schleyer, P. Nucleus-independent chemical shifts (NICS) as an aromaticity criterion. *Chem. Rev.* **105**, 3842–3888 (2005).
